# A *Rhodnius prolixus* Insulin Receptor and Its Conserved Intracellular Signaling Pathway and Regulation of Metabolism

**DOI:** 10.3389/fendo.2018.00745

**Published:** 2018-12-06

**Authors:** Marina S. Defferrari, Sara R. Da Silva, Ian Orchard, Angela B. Lange

**Affiliations:** Department of Biology, University of Toronto Mississauga, Mississauga, ON, Canada

**Keywords:** Rhopr-IR, Akt, Gsk3b, FOXO, lipid, insect, RNAi, Western blot

## Abstract

The insulin signaling pathway is a modulator of metabolism in insects and can regulate functions associated with growth and development, as well as lipid and carbohydrate balance. We have previously reported the presence of an insulin-like peptide and an insulin-like growth factor in *Rhodnius prolixus*, which are involved in the homeostasis of lipids and carbohydrates in post-feeding and non-feeding periods. In the present study, we have characterized the first insulin receptor (IR) to be discovered in *R. prolixus*, Rhopr-IR, and investigated its intracellular signaling cascade and its role in nutrient control. We identified a candidate protein sequence within *R. prolixus* putative peptidome and predicted its conserved features using bioinformatics. Tissue-specific expression analyses indicated that the Rhopr-IR transcript is differentially-expressed in all tissues tested, with the highest values observed in the central nervous system (CNS). Treatment of insects with the IR kinase activator BpV(phen), glucose, or porcine insulin resulted in the activation of protein phosphorylation in the fat body, and stimulated the phosphorylation of protein kinase Akt, an evolutionarily conserved key regulator of the intracellular insulin signaling cascade. We also observed activation of Akt and phosphorylation of its downstream targets glycogen synthase kinase 3 β (GSK3β) and the transcription factor FOXO for several days following a blood meal. We used dsRNA to knockdown transcript expression and examined the resulting effects on metabolism and intracellular signaling. Furthermore, knockdown of the Rhopr-IR transcript increased lipid levels in the hemolymph, while reducing lipid content in the fat body. Interestingly, the levels of carbohydrates in the hemolymph and in the fat body did not show any alterations. The activation of Akt and phosphorylation of FOXO were also reduced in knockdown insects, while the phosphorylation pattern of GSK3β did not change. Our results support the identification of the first IR in *R. prolixus* and suggest that Rhopr-IR signaling is involved in hemolymph nutrient homeostasis and fat body storage both in post-feeding and in non-feeding stages. These metabolic effects are likely regulated by the activation of Akt and downstream cascades similar to mammalian insulin signaling pathways.

## Introduction

The insulin signaling pathway is an evolutionarily conserved regulator of physiological functions related to metabolism and is well-known for balancing glucose uptake and storage in mammals. However, the pathway is found in all metazoans and has a broad pleiotropic nature, playing additional roles in reproduction, development, growth, and life span (1, 2). The signaling cascade is initiated by the activation of an insulin receptor (IR) upon binding of insulin, insulin-like peptides (ILPs), or insulin-like growth factors (IGFs). IRs are part of the receptor tyrosine kinase (RTK) family, which contain tyrosine kinase domains within their cytoplasmic portion. In humans, the insulin receptor is expressed in two isoforms, denoted IR isoform A (IR-A) and IR isoform B (IR-B), due to the alternative splicing of exon 11 found within the IR gene (3, 4). Whereas, the shorter IR-A is mainly expressed in cells of the central nervous system, hematopoietic cells, and several fetal tissues (4, 5), the longer IR-B isoform is primarily expressed in muscle, liver and adipose tissue (4). Although previous work suggests that human IR-A displays a higher binding affinity to insulin than IR-B (6), it has also been found that IR-A can bind with high affinity to insulin growth factor II (IGF II) as well (5). These isoforms also display differential signaling in response to insulin. Whereas, IR-A stimulation in mammalian pancreatic β cells leads to the increased transcription of the insulin gene through the involvement of the PI3K class Ia, p70 s6 and Ca^2+^/calmodulin kinases, insulin signaling at IR-B results in the activation of PI3K class II, the downstream kinase Akt, and resulting transcription of the glucokinase (βGK) gene, resulting in the regulation of metabolic events (7).

Upon receptor activation, the intracellular kinase domains of both IR isoforms initiate the auto-phosphorylation of the IR, which recruits adaptor proteins termed insulin response substrates (IRS) that facilitate a cascade of tyrosine phosphorylation (8–10). Once activated, the IRS can trigger different downstream signaling pathways by interacting with either Grb2 (growth factor receptor bound protein-2) or PI3K (phosphatidylinositol-3 kinase). The activated Grb2 protein initiates the Ras-MAPK (mitogen-activated protein kinase) pathway by associating with the son-of-sevenless (SOS) protein leading to the recruitment of Ras and the activation of its GTPase function. The activated Ras protein then initiates the serine/threonine kinase activity of the MAPK pathway, leading to mitogenic responses within target cells (11). Stimulation of MAPK and its downstream response elements results in cell growth and division by activating the MEK/ERK complex via the Ras and Raf proteins (12). In contrast, PI3K activates the PI3K/PKB (protein kinase B) pathway (12, 13), leading to the production of PIP_3_ (phosphatidyl-inositol triphosphate), which in turn promotes the activation of downstream kinases such as Akt 2 (PKB) and PKC (protein kinase C) (13). Activated Akt phosphorylates different downstream targets such as GSK3β (glycogen synthase kinase 3β), as well as select isoforms of the FOXO (Forkhead box) transcription factor family that control cell cycle arrest, apoptosis, and nutrient metabolism, among other functions (14). Phosphorylation of GSK3β stimulates the storage of glucose by facilitating the formation of glycogen (15), while the phosphorylation of FOXO relieves cell cycle arrest, inhibits apoptosis, and promotes cell growth (16).

In vertebrates, there are three known receptors in the IR subfamily: the insulin receptor, which binds to insulin; the type 1 IGF receptor, which binds to IGF I and II; and the orphan IR-related receptor (17). In contrast, only one or two IRs have been identified in most invertebrate species investigated, including insects. Single IRs were described in *Drosophila melanogaster* (18), in the mosquitoes *Culex quinquefasciatus* and *Aedes aegypti* (19, 20), and in the dung beetle *Onthophagus nigriventris* (21). The presence of two IRs was reported in the honey bee *Apis mellifera* (22), in the fire ant *Solenopsis invicta* (23), in the brown plant hopper *Nilaparvata lugens* (24), in the beetle *Tribolium castaneum* (25), and in the citrus aphid *Aphis citricidus* (26). Unlike IRs, the number and sequences of ILPs and IGFs can vary among insect species, ranging from one single insulin-related peptide in *Locusta migratoria* (27), to eight ILPs identified in *D. melanogaster* (DILPs) (18), to more than 30 ILP genes discovered in *Bombyx mori* (28). Much like in other animals, insulin signaling controls a variety of processes in insects, including lipid and carbohydrate homeostasis (29–31), wing size (26), caste differentiation (22, 23), fecundity (20), and reproduction (25).

We have previously reported the presence of one ILP and one IGF in the blood-gorging insect *R. prolixus*. We found that the ILP, namely Rhopr-ILP, is only produced in neurosecretory cells in the brain and is involved in lipid and carbohydrate homeostasis during post-feeding and non-feeding periods (32). In contrast, Rhopr-IGF is expressed in a variety of tissues, with the highest transcript levels found in the fat body. We found that Rhopr-IGF also contributes to hemolymph nutrient balance in addition to regulating wing and body size (33). Here, we identify and characterize an IR in *R. prolixus*. The Rhopr-IR transcript is expressed in all tissues investigated, with highest expression levels identified in the central nervous system (CNS). We discovered that receptor activation is responsive to insulin stimulation and initiates the activity of Akt, resulting in the coordinated phosphorylation of downstream targets GSK3β and FOXO. Finally, knockdown of Rhopr-IR transcript renders an imbalance in hemolymph and fat body lipid homeostasis.

## Materials and Methods

### Identification of a Candidate Insulin Receptor Protein Sequence

The predicted peptidome of *R. prolixus* (available at rprolixus.vectorbase.org) was scanned using different insulin receptor protein sequences (*Homo sapiens* XP_011526290.2, *D. melanogaster* AAC47458.1, *Onthophagus nigriventris* AFQ20827.1) using the program Geneious 8.1.7 (34). The correspondent candidate mRNA sequence was identified by BLASTing the putative protein sequence against *R. prolixus* transcriptome (available at rprolixus.vectorbase.org) on Geneious 8.1.7. The conserved features and domains of the Rhopr-IR were identified using the online service InterPro: protein sequence analysis and classification (www.ebi.ac.uk/interpro). The tyrosine kinase active site and signature were predicted using the online database Prosite (https://prosite.expasy.org/). The transmembrane region was confirmed using the TMHMM Server v. 2.0 (www.cbs.dtu.dk/services/TMHMM/) and Phobius predictor (phobius.sbc.su.se), which was also used for predicting the signal peptide. Glycosylation and phosphorylation sites were predicted using the NetNGlyc 1.0 Server (www.cbs.dtu.dk/services/NetNGlyc/) and the NetPhos 3.1 Server (www.cbs.dtu.dk/services/NetPhos/), respectively.

### Phylogenetic Analysis of Insulin Receptor Protein Sequences

The predicted insulin receptor protein sequence from *R. prolixus* was aligned with 57 other sequences from 54 different vertebrate and invertebrate species using the program MUSCLE 3.8 (Multiple Sequence Comparison by Log-Expectation—www.ebi.ac.uk/Tools/msa/muscle).

The evolutionary history of insulin receptors was inferred by analyzing the tyrosine kinase domain within the alignment described above using the maximum likelihood method (35) on the program Molecular Evolutionary Genetics Analysis version 7.0 [MEGA7, (36)]. The bootstrap consensus tree was inferred from 500 replicates and branches corresponding to partitions reproduced in < 50% bootstrap replicates were collapsed. The percentage of replicate trees in which the associated taxa clustered together in the bootstrap test (500 replicates) are shown next to the branches. The distances were estimated using a JTT model with a discrete Gamma distribution, 4 categories (+G, parameter = 0.4310 for the tyrosine kinase tree, parameter = 1.0151 for the extracellular domains tree).

### Insects

Fifth instar *R. prolixus* were used throughout the study. The colony was kept at 25°C, under 50% humidity, and insects were fed on defibrinated rabbit blood (Cedarlane Laboratories Inc., Burlington, ON, Canada) once per instar, or as indicated.

### Analysis of Rhopr-IR Relative Transcript Expression in 5th Instar *R. prolixus*

The relative expression of Rhopr-IR was quantified using real-time quantitative PCR (qPCR) in 10 different tissues from unfed 5th instars. RNA was extracted using a Total RNA mini kit (BioBasic, Markham, ON, Canada), followed by cDNA synthesis using the High Capacity cDNA Reverse Transcription Kit (Applied-Biosystems, Fisher Scientific, Toronto, ON, Canada). Primers for the amplification of Rhopr-IR (Supplementary Table 1) were designed to amplify fragments of similar size across all experimental and reference genes (β-actin, α-tubulin, rp49) (37, 38). All the qPCR reactions were performed using a CFX384 Touch^TM^ Real-Time PCR Detection System (Bio-Rad, Mississauga, ON, Canada). Relative expression was calculated using the ΔΔCt method (39).

Alternatively, semi-quantitative reverse transcriptase-PCR (RT-PCR) was performed to confirm the presence of the transcript in tissues with low relative expression. The same Rhopr-IR primers used for real-time PCR (Supplementary Table 1) were used and actin was amplified as a transcript reference. RNA from unfed 5th instars was extracted, as mentioned above. The result of the RT-PCR was visualized on 1.2% agarose gels.

### Fat Body Collection

Fat bodies were collected from both unfed and recently fed 5th instars to investigate the signaling of the Rhopr-IR pathway under endogenous or exogenous stimulation. For unfed insects, the ventral and dorsal fat bodies were removed 4–5 weeks after feeding as 4th instars (5 pairs combined per trial) under *R. prolixus* glucose-free physiological saline (NaCl 150 mM, KCl 8.6 mM, CaCl_2_ 2.0 mM, MgCl_2_ 8.5 mM, NaHCO_3_ 4.0 mM, HEPES 5.0 mM, pH 7.0). For post-feeding signaling analysis, ventral and dorsal fat bodies were removed 1 day before feeding, and 2–4 h and every day for 5 days after feeding (5 pairs combined per time point per trial). Once removed from the insects, tissues were immediately stored in cold phosphate-buffered saline (PBS) and frozen at −20°C until sample preparation for Western blotting (see below).

For unfed insects treated with BpV(phen) (Millipore-Sigma, Milwaukee, WI, USA) or porcine insulin (Millipore-Sigma, Oakville, ON, Canada), the ventral and dorsal fat bodies (5 pairs combined per trial) were removed under *R. prolixus* glucose-free saline 30 min post-injection with either 1 μL of *R. prolixus* saline (with or without 34.0 mM glucose), BpV(phen) (10^−3^M), or porcine insulin (0.1 or 1 μg). Tissues were stored immediately in cold PBS and frozen at −20°C until lysed and used for Western blotting.

### Tissue Lysis and Protein Quantification

Fat body tissues collected from unfed and recently fed insects were thawed from storage at −20°C (in PBS) and were immediately submerged in cold, freshly-made lysis buffer (aprotinin, 0.31 nM; leupeptin, 21 nM; pepstatin A, 1.5 nM; phenylmethylsulfonyl fluoride, 1 mM, EDTA, 5 mM; EGTA, 1 mM; sodium fluoride, 10 mM; sodium orthovanadate, 1 mM; in RIPA buffer [150 mM NaCl, 1% Triton X-100, 0.5% sodium deoxycholate, 0.1% SDS, 50 mM Tris, pH 8.0 in double-distilled or MilliQ water]). For post-feeding analysis of fat bodies, tissues were collected 1 day before feeding, and 2–4 h and every day for 5 days post-feeding and were stored −20°C (in PBS) until use. After thawing, tissues were first weighed collectively (per trial per day), followed by incubation in cold, freshly-made lysis buffer. Once in lysis buffer, samples were sonicated 5 × for 3 s each, followed by a 2-h incubation at 4°C with constant agitation. Samples were then centrifuged for 25 min at 4°C and 17,000 *g*. The resulting infranatant was collected and used for Western blotting. Protein quantification was done on all lysed tissue samples prior to gel electrophoresis using the BCA protein quantification assay (Pierce™ BCA Protein Assay Kit, ThermoFischer Mississauga, ON, Canada).

### Gel Electrophoresis and Western Blotting

After protein quantification, lysed fat body samples from unfed and recently fed insects were subjected to gel electrophoresis. Protein bands were separated under reducing conditions on hand-cast (TGX Stain-Free^TM^ FastCast^TM^ acrylamide solution, Bio-Rad, Mississauga, ON, Canada; prepared according to protocol specifications) or pre-made (Mini-PROTEAN® Stain-Free^TM^ gels, Bio-Rad) 12% stain-free SDS-polyacrylamide gels and loaded in equal amounts across all wells (amounts loaded specified per experiment). All gels were run for 60–90 min at a constant voltage (120 V) in Tris/Glycine/SDS running buffer (25 mM Tris, 192 mM glycine, 0.1% SDS, pH 8.3; Bio-Rad). Proteins were then transferred to a low-fluorescence PVDF (LF-PVDF) membrane in Transfer buffer over 3 min, using a Trans-Blot® Turbo™ Transfer System (all reagents/materials: Bio-Rad). Membranes were then blocked overnight in PBS-T (1xPBS containing 0.1% Tween-20) and 5% bovine serum albumin (BSA). Blots were incubated overnight in primary antibody (1:1000 dilution in PBS-T with 3% BSA) at 4°C, against the following antigens: anti-Akt [Akt (pan) (C67E7) rabbit monoclonal antibody, Cell Signaling Technology, Beverly, MA, USA]; anti-pAkt (phospho-Akt (Ser473) (D9E) XP® rabbit monoclonal antibody, Cell Signaling Technology); anti-GSK3β (phospho-GSK-3Beta (Ser9) (D85E12) XP® rabbit monoclonal antibody, Cell Signaling Technology); anti-FOXO (phospho-FOXO (Ser256) rabbit polyclonal antibody, Cell Signaling Technology); anti-actin (rabbit polyclonal antibody, Millipore-Sigma, Oakville, ON, Canada); or anti-tubulin (mouse monoclonal antibody, Life Technologies, Burlington, ON, Canada). After incubation, primary antibodies were washed-off with PBS-T followed by incubation in secondary antibody (1:5000, horseradish peroxidase (HRP)-conjugated anti-mouse or anti-rabbit antibodies, Cell Signaling Technology) for 1–2 h at room temperature with constant agitation. Blots were then washed with PBS-T and visualized using enhanced chemiluminescence (Clarity^TM^ Western ECL Substrate, Bio-Rad). Blots were imaged on a ChemiDoc XRS system and analyzed using Image Lab 5.0 (Bio-Rad software and systems) using the “intense band” automatic exposure setting in the “high resolution” and/or “high sensitivity” default blot protocol options, in order to optimize the exposure time for intense bands and prevent overexposure of the blot/minimize detection of background noise. For the complete blots corresponding to those used in subsequent figures please see Supplementary Data.

### Double-Stranded RNA Design and Synthesis

A 474-base pair template, designed in the 5′ region of the open reading frame of Rhopr-IR transcript, was used to synthesize a double stranded RNA molecule (dsIR) using the T7 Ribomax Express RNAi System (Promega, Madison, WI, USA), according to the manufacturer protocol. Gene specific primers (GSP) were combined with GSP containing the T7 RNA polymerase promoter sequence (Supplementary Table 1). As an experimental control, a dsRNA molecule based on the Ampicillin Resistance Gene (dsARG) from the pGEM-T Easy Vector system (Promega, Madison, WI, USA) was used throughout the study (38, 40).

### Knockdown of Rhopr-IR Transcript Expression Using Double Stranded RNA

To knockdown the expression of Rhopr-IR in unfed 5th instar *R. prolixus*, 1 μg of dsARG or dsIR in 1 μL of ultrapure water was injected into the insect hemocoel using a Hamilton micro syringe (Hamilton Company, Reno, NV, USA). Insects were dissected at 3 and 10 days post-injection and Rhopr-IR transcript expression was measured using quantitative PCR. The knockdown percentage in dsIR-injected insects was calculated relative to the expression of the transcript in dsARG-injected insects. In another experiment, at 3 days after injection, insects were fed on defibrinated rabbit blood, separated in groups of 40 individuals of dsARG or dsIR- injected insects. Each group was allowed to feed for 25 min.

### Hemolymph Collection From Rhopr-IR Knockdown Insects

For unfed insects, hemolymph was collected at 7 days post-dsRNA injection, and for the recently fed insects, hemolymph was collected at 4 days post-feeding (7 days post-injection). Hemolymph samples were obtained upon immobilizing insects, after which 5 μL were collected from the cut end of a leg of each insect using a micro-pipette. Samples were immediately placed in 50 μL of 10% trichloroacetic acid (TCA) and centrifuged for 5 min at 20°C, 8000 *g*. Supernatants were then transferred to new microcentrifuge tubes and subsequently used for carbohydrate level measurements. Pellets containing lipids associated with lipoproteins were resuspended in 200 μL isopropanol and used for lipid level measurements.

### Fat Body Collection of DSRNA-Injected Insects

For unfed insects, fat bodies were collected at 7 days post-dsRNA injection, and for recently fed insects, fat bodies were collected at 4 days post-feeding (7 days post-injection). The ventral fat body sheet covering the abdominal segments was removed under *R. prolixus* physiological saline (containing 34.0 mM glucose) and placed in 200 μL isopropanol or 200 μL 10% TCA. Samples were sonicated for 5 s each and centrifuged for 10 min at 4°C, 8000 *g*. Following centrifugation, 20 μL of each isopropanol supernatant were transferred to new tubes containing 180 μL isopropanol for total fat body lipid content measurements, while 50 μL of each TCA supernatant were transferred to new tubes for measuring total fat body carbohydrate content.

To evaluate the effects of Rhopr-IR knockdown on fat body growth and IR signaling, both unfed and recently fed insects were injected with 1 μg of dsARG or dsIR in 1 μL of ultrapure water. For unfed insects, the ventral and dorsal fat bodies were removed (5 pairs per trial) at 4 days post-injection. In another experiment, insects injected with dsARG or dsIR were fed 4 days post-injection and ventral and dorsal fat bodies were collected between 2 and 4 h and every day for 5 days after feeding (5 pairs per time point per trial). Once removed, all tissues were immediately stored in PBS and frozen at −20°C until undergoing the same preparation for Western blotting, as described above.

### Lipid and Carbohydrate Measurements From Hemolymph and Fat Body Samples

Lipid (41) and carbohydrate (32) measurements were carried out using Lipid and Anthrone-based assays, as previously described.

### Statistical Analyses

Results are shown as means ± standard errors. The statistical significance of the data was calculated using either a Student's *t*-test or a one-way ANOVA followed by Dunnett's multiple comparisons test, where specified. Results were considered statistically different when *p* < 0.05. All analyses were carried out using the programs SigmaPlot (Systat Software, San Jose, California, USA) or GraphPad Prism 7 (GraphPad Software, La Jolla, California, USA, www.graphpad.com).

## Results

### Analysis of Rhopr-IR Amino Acid Sequence and Prediction of Conserved Features and Domains

**Figure 1:**
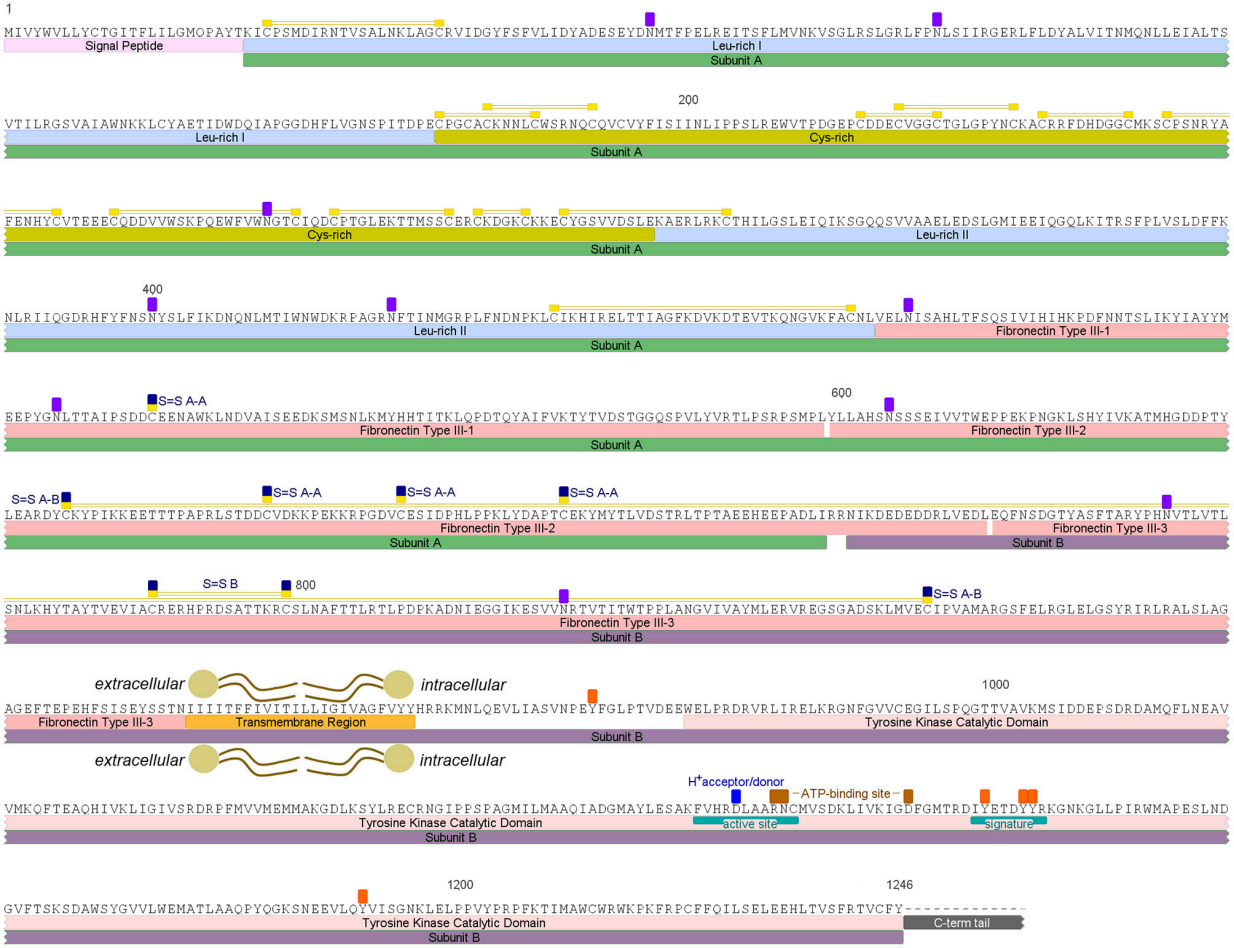
Rhopr-IR putative amino acid sequence and predicted conserved features and domains. Rhopr-IR monomer subunits A and B are highlighted by green and purple boxes, respectively. Signal peptide is highlighted in light pink, followed by Leu-rich domains highlighted in blue and a Cys-rich domain highlighted in dark yellow. Disulfide bonds are identified by yellow boxes over Cys residues, connected by two yellow lines. FnIII domains are indicated by pink boxes. The Cys residues in the subunit A that form disulfide bonds with Cys residues in the other subunit A of the active receptor dimer are indicated by yellow/dark blue boxes over the residues (S = S A-A). The two Cys residues that form the disulfide bond between subunits A and B of the monomer, are indicated by yellow/dark blue boxes over the residues (S-S A-B) connected by two yellow lines. The transmembrane region is highlighted by an orange box, and the Tyr kinase catalytic domain by a pink box. The active site, F-V-H-R-D-L-A-A-R-N-C (1097–1107), containing a proton donor/acceptor, and the signature that characterizes receptors Tyr kinase class II, D-I-Y-E-T-D-Y-Y-R (1125–1133), are indicated with teal boxes within the catalytic domain. ATP-binding sites between the active site and the receptor signature are indicated with brown boxes over the residues. Potentially phosphorylated Tyr residues in the intracellular region and potentially glycosylated Asn residues in the extracellular portion, are indicated by orange and purple boxes, respectively.

An insulin receptor candidate sequence was identified within the *R. prolixus* peptidome (RPRC006251-PA) along with its coding mRNA sequence (RPRC006251-RA), which was used for designing the primers for Rhopr-IR quantification using qPCR and for dsRNA production. The protein sequence was previously annotated on the UniprotKB database under the general classification of “Receptor Protein-Tyrosine Kinase” (T1HQC7_RHOPR). The Rhopr-IR monomer is composed of two subunits (A and B) that are connected and stabilized by disulfide bonds, and its candidate sequence was found to be 1,246 amino acids long (Figure 1; Supplementary Figure 1). According to the multiple sequence alignment performed (Supplementary Figure 2), the predicted sequence of Rhopr-IR is missing the intracellular C-terminal tail that follows the tyrosine kinase catalytic domain and further investigation is needed in order to characterize this portion of the receptor that is likely to be present in the protein. The Rhopr-IR sequence also contains a signal peptide with a cleavage site between residues 25 and 26, followed by a leucine-rich domain, a cysteine-rich domain, and a second leucine-rich domain, which together incorporate the12 predicted disulfide bonds likely involved in establishing the structure of the ligand binding region (10). Three fibronectin type III (FnIII) domains follow the second leucine-rich domain and contain the cysteine residues (Cys) that are likely involved in the dimerization of the active receptor and in the connection of subunits A and B in the monomer. FnIII-1 is located within subunit A and contains one Cys (528) that likely forms a disulfide bond with the equivalent residue located in the other subunit A of the Rhopr-IR dimer. FnIII-2 starts in subunit A, ends in subunit B, and contains three Cys (668, 682, 699) that likely form disulfide bonds with their equivalent residues in the other subunit A of the active receptor dimer. FnIII-3 is located in subunit B and is predicted to contain one disulfide bond between Cys784 and Cys798, which is probably involved in stabilizing the structure of the monomer. The disulfide bond that connects subunits A and B is predicted to be formed by Cys647 in FnIII-2 and Cys865 in FnIII-3.

The transmembrane domain follows FnIII-3 in subunit B and is 24 amino acids long. The tyrosine kinase catalytic domain is located in the intracellular portion of subunit B and contains a tyrosine kinase-specific active site, defined by the sequence F-V-H-R-D-L-A-A-R-N-C (1097–1107), a proton donor/acceptor site (Asp1101), and two ATP-binding sites (Arg1105–Asn1106 and Asp1119). A receptor tyrosine kinase class II signature is also present in subunit B and is defined by the sequence D-I-Y-E-T-D-Y-Y-R (1125–1133). Furthermore, there are five tyrosine residues that are potentially phosphorylated in the intracellular portion of the receptor, and ten asparagine residues in the extracellular portion that are potentially glycosylated in the mature receptor.

### Phylogenetic Analysis of Rhopr-IR

**Figure 2:**
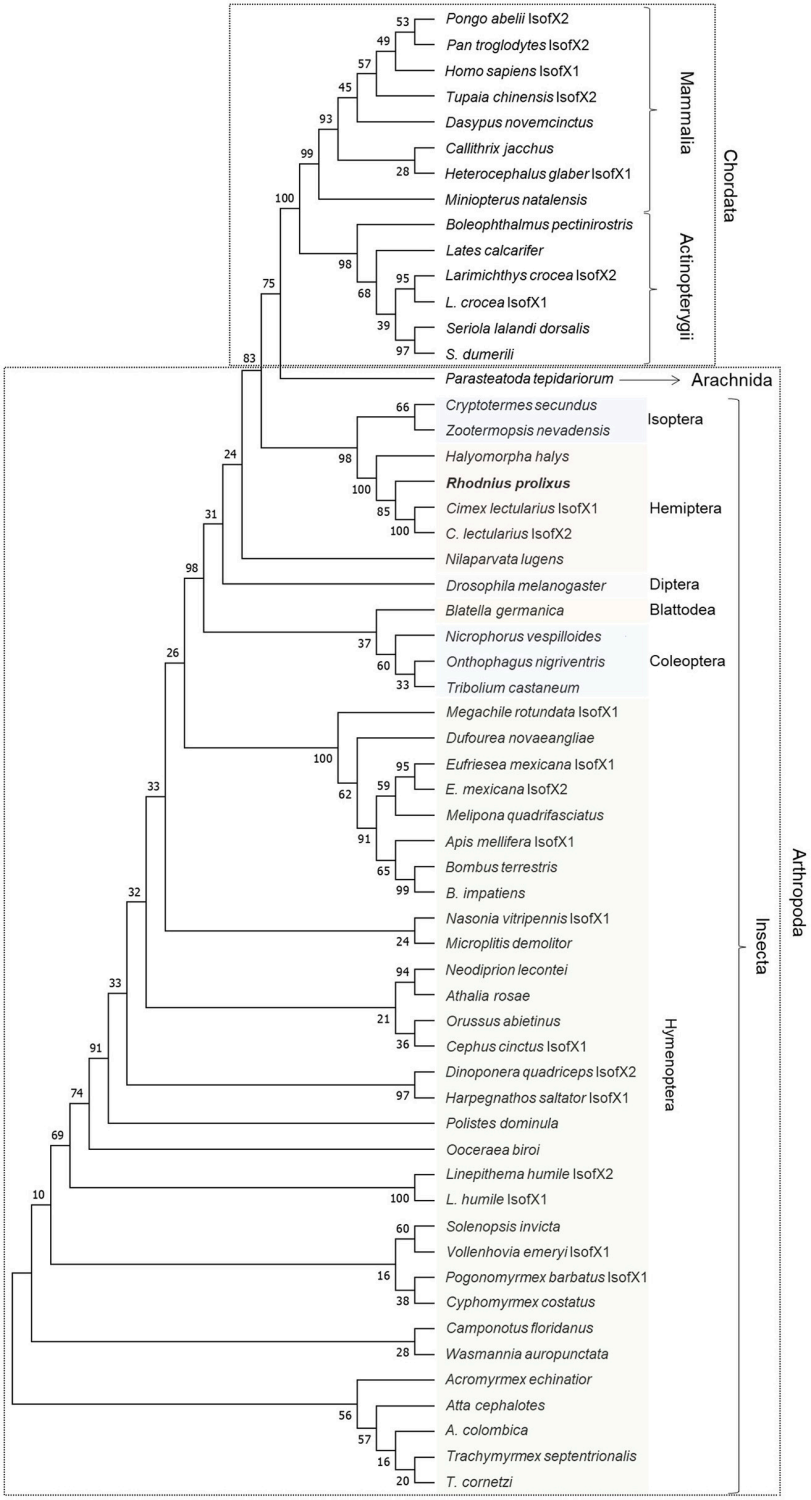
Maximum likelihood inference for phylogenetic analysis of insulin receptor sequences from vertebrates and invertebrates using the tyrosine kinase domain. The phylogenetic tree shows the relationships among 58 tyrosine kinase domain sequences of insulin receptors from 54 different species of vertebrates (Chordata) and invertebrates (Arthropoda), including Rhopr-IR sequence. The tree was generated by maximum likelihood inference using the JTT + G model and the bootstrap consensus tree was inferred from 500 replicates. The analyses were conducted in MEGA7 (36).

The alignment of the insulin receptor protein sequences was constructed using the online tool MUSCLE and a highly conservative region was selected to generate a maximum likelihood phylogenetic tree (Supplementary Figure 2). The tree was generated using the intracellular portion of the sequence containing the tyrosine kinase catalytic domain (Figure 2). *R. prolixus* Rhopr-IR grouped with other hemipterans, suggesting the existence of close relationships to both the bed bug (*C. lectularius*) and to the brown marmorated stink bug (*H. halys*).

### Characterization of Tissue-Specific Expression of Rhopr-IR Transcript in 5th Instar *R. prolixus*

**Figure 3:**
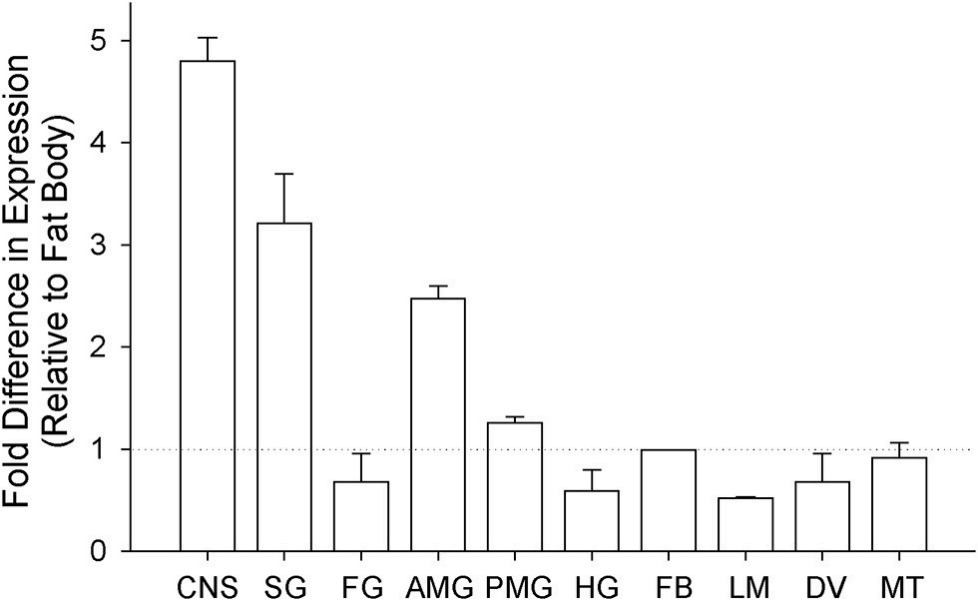
Analysis of tissue-specific expression of Rhopr-IR transcript in 5th instars. Unfed 5th instar *R. prolixus* were dissected and tissues were pooled into 10 different groups (CNS, central nervous system; SG, salivary glands; FG, foregut; AMG, anterior midgut; PMG, posterior midgut; HG, hindgut; FB, fat body; LM, leg muscles; DV, dorsal vessel; MT, Malpighian tubules). The expression of Rhopr-IR transcript in each tissue was quantified relative to the expression in the fat body using qPCR and the ΔΔCt method (39). The experiment was repeated 3 times and results are shown as means + std error.

The tissue-specific relative transcript expression of Rhopr-IR was investigated using qPCR (Figure 3) and the presence of the transcript in tissues with the lowest relative expression was confirmed using semi-quantitative RT-PCR (Supplementary Figure 3). Rhopr-IR transcript was present in all tissues analyzed, with the highest relative expression identified in the CNS, followed by the salivary glands, the anterior midgut, and the posterior midgut. The remaining tissues (foregut, hindgut, fat body, leg muscles, dorsal vessel, and Malpighian tubules) displayed very similar and lower levels of Rhopr-IR transcript expression.

### The fat Body of *R. prolixus* Demonstrates IR Signaling Through a Phosphorylation Cascade

**Figure 4:**
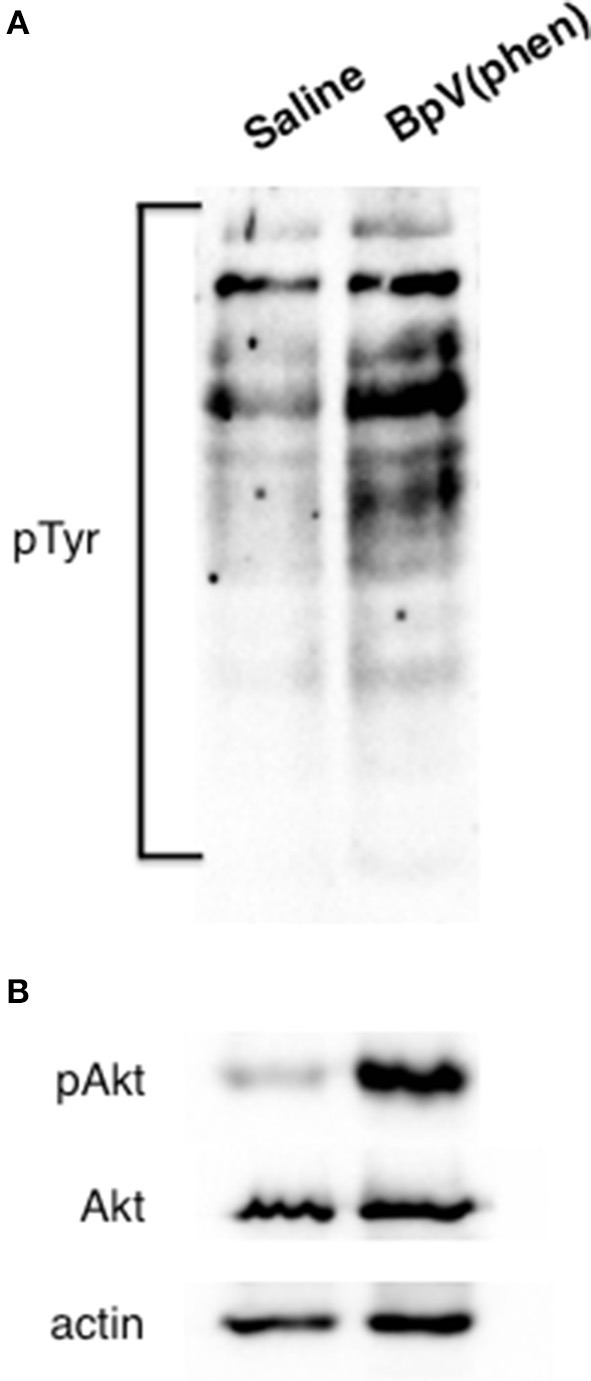
Activation of Rhopr-IR signaling triggers a phosphorylation cascade. The mammalian IR-specific activator BpV(phen) also appears to activate Rhopr-IR in the fat body of 5th instar *R. prolixus*. **(A)** The injection of unfed insects with BpV(phen) (10^−3^ M) results in a general increase in the phosphorylation of tyrosine residues within the fat body, as compared to injection with glucose-free saline alone. Phosphorylation was detected on a Western blot evaluating anti-phosphotyrosine (primary anti-phosphotyrosine antibody, 1:1000, visualized using Chemiluminescence; *n* = 5 insects per blot). **(B)** Injecting unfed insects with BpV(phen) stimulated the phosphorylation of the IR-responsive kinase Akt, while no changes in the relative amounts of unphosphorylated Akt or actin were noted between control insects and those treated with BpV(phen). Phosphorylation of Akt was detected on a Western blot probing for anti-phospho-Akt (primary anti-phospho-Akt antibody, 1:1000; *n* = 5 insects per blot).

To investigate the signaling activity of Rhopr-IR, insects were first injected with either *R. prolixus* glucose-free saline or 10^−3^ M BpV(phen) (in glucose-free saline). This activator is well characterized as a specific activator of mammalian IRs, which is achieved through the BpV(phen)-mediated inhibition of the protein-tyrosine phosphatase 1B (PTP1B) responsible for negatively regulating basal IR activity (42). Once inhibited, PTP1B is incapable of catalyzing the dephosphorylation of mammalian IR proteins, leading to an increase in the levels of basal auto-phosphorylated receptor. In mammals, this leads to an increase in the intracellular levels of phosphorylated tyrosine species (43). In the present study, fat bodies were harvested from injected insects after 30 min, and the general phosphorylation of cellular tyrosine (Tyr) residues was assessed. As seen in Figure 4A, treatment with BpV(phen) induced a global increase in intracellular Tyr phosphorylation, as indicated by an increase in the intensity of the anti-pTyr bands relative to those in saline controls. The activation of Rhopr-IR by BpV(phen) was further supported by the observed increase in the phosphorylation of the IR downstream target Akt (pAkt; Figure 4B), compared with low apparent pAkt detection in saline controls. Thus, it appears that the mammalian IR activator BpV(phen) can also activate an IR-like receptor in *R. prolixus*, and that the activation of Rhopr-IR subsequently induces a tyrosine phosphorylation cascade typical of other IR signaling cascades in mammals and insects alike (19, 44, 45). Furthermore, it appears that BpV(phen) treatment also induces the phosphorylation of the potential Rhopr-IR downstream protein target Akt, and thus likely stimulates the coordinated phosphorylation of other proteins in the Rhopr-IR pathway.

### Endogenous Rhopr-IR Activation Exhibits a Distinct Pattern Following Feeding

**Figure 5:**
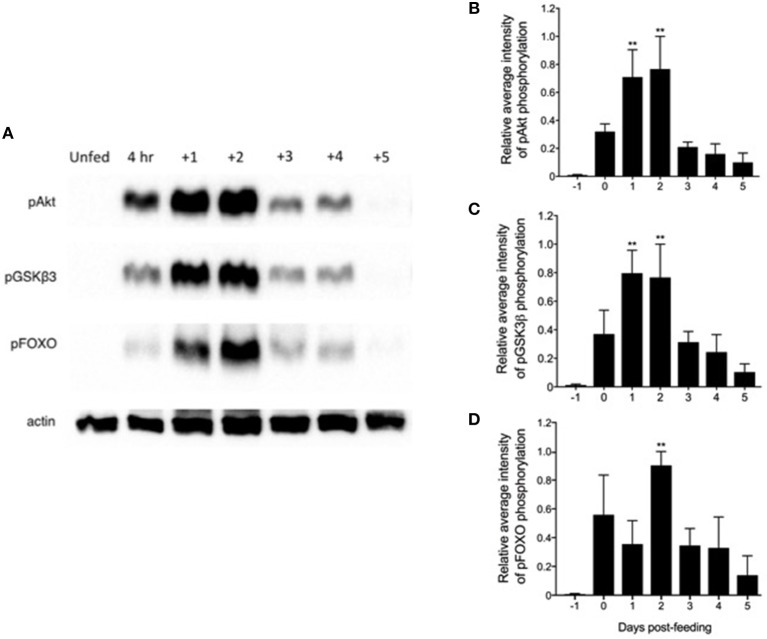
Feeding triggers a coordinated phosphorylation cascade within a Rhopr-IR associated pathway**. (A)** Fat bodies were dissected from unfed 5th instar *R. prolixus* 1 day prior to feeding (“Unfed”), between 2 and 4 h immediately after feeding (“4 h”), and up to 5 days after feeding on a rabbit blood meal (*n* = 5 insects per day per trial; 3 trials completed in total). Western blots were conducted to probe for anti-phospho-Akt (pAkt), anti-pGSKβ3, and anti-pFOXO (primary antibodies, 1:1000; visualized using Chemiluminescence), which form part of a downstream pathway stimulated by Rhopr-IR signaling. The phosphorylation of all three proteins increased significantly post-feeding, with pAkt **(B)** and pGSKβ3 **(C)** reaching peak phosphorylation as soon as 1 day post-feeding, and with pFOXO **(D)** reaching peak relative phosphorylation levels 2 days post-feeding. The experiment was repeated 3 times (*n* = 5 insects per trial) and results are shown as means + std error. **(*p* < 0.01) indicates statistically significant difference, inferred using one-way ANOVA with Dunnett's multiple comparisons test.

Previous reports indicated that an ILP released from neurosecretory cells in the CNS peaked within 4 days post-feeding, implying the stimulation of whole-body insulin-like signaling in this time frame (46). Therefore, we investigated the activation of the Rhopr-IR pathway within the fat body of 5th instar insects after a blood meal, compared with unfed controls. As seen in Figure 5A, there is a distinct pattern of pathway stimulation as soon as 4 h after feeding, as indicated by the increased intensity of phospho-Akt, -GSK3β, and –FOXO bands, compared to relatively no detection of phosphorylation in unfed insects. The most significant phosphorylation of Akt and GSK3β occurred between 1 and 2 days post-feeding (Figures 5B,C), while the phosphorylation of FOXO, located downstream in the IR pathway, peaked at 2 days after feeding (Figure 5D). The phosphorylation of all pathway components decreased markedly at 3–4 days post-feeding and remained at low levels for up to 7 days after ingestion of the blood meal (data not shown). The coordinated phosphorylation of these pathway components suggests the stimulation of Rhopr-IR signaling in the fat body by Rhopr-ILP after a blood meal.

**Figure 6:**
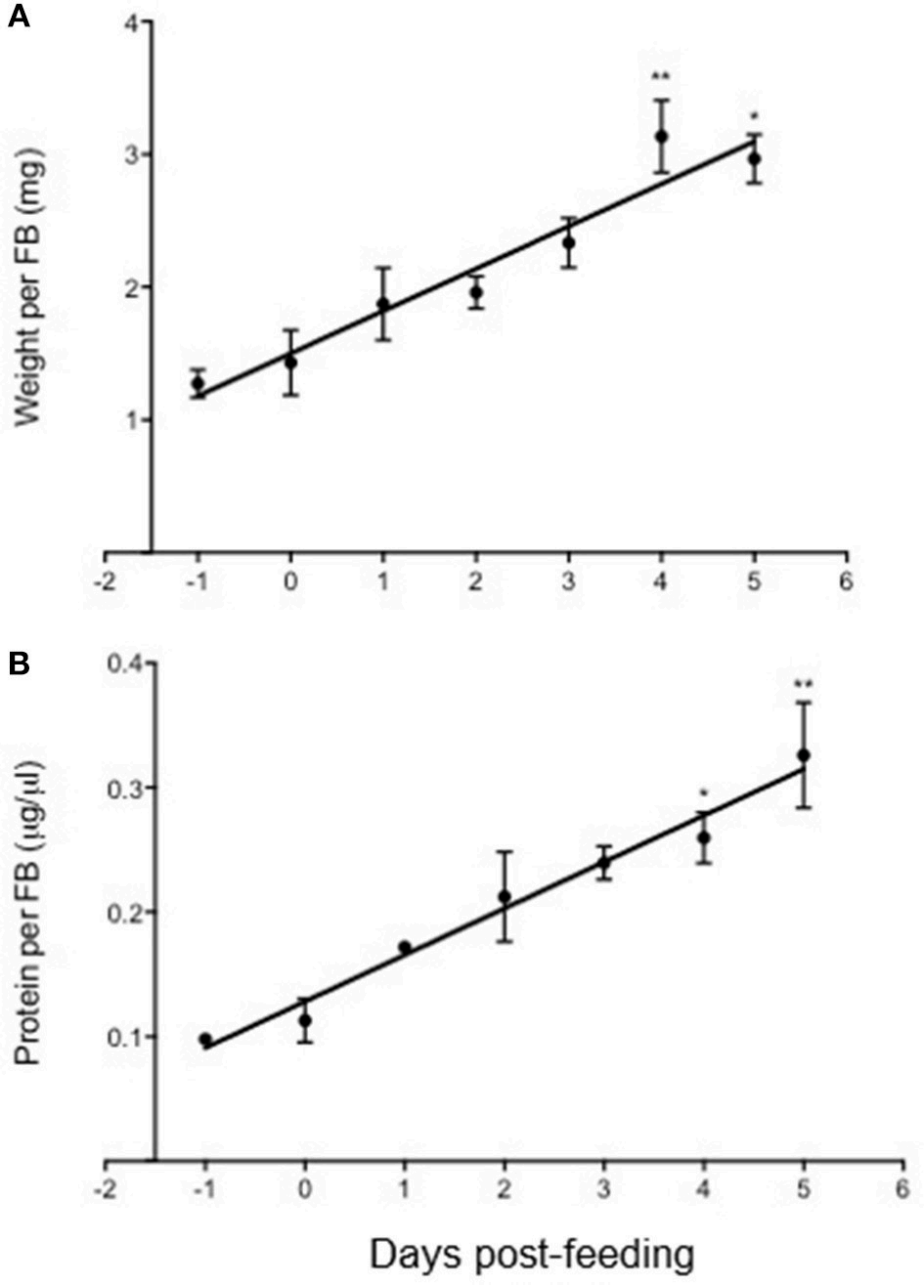
Changes in fat body weight and protein content in *Rhodnius prolixus* after feeding on a blood meal. The weight (mg) and protein content (μg/μl) of fat bodies isolated from 5th instar *R. prolixus* were measured prior to and up to 5 days after feeding on a blood meal (*n* = 5 insects per day per trial; 3 trials completed). Both the weight **(A)** and protein content **(B)** of fat bodies increased significantly post-feeding, and the accumulation of weight and protein amount correlated linearly up until 5 days after feeding. The experiment was repeated 3 times (*n* = 5 insects per trial) and results are shown as means + std error. *p < 0.05) and **p < 0.01) indicate statistically significant difference, inferred using one-way ANOVA with Dunnett's multiple comparisons test.

The weight (mg) and protein concentration (μg/μl) of 5th instar fat bodies were also determined for 5 days post-feeding compared to unfed (Figure 6). It was observed that fat bodies significantly increased in weight and protein concentration as early as 4 days post-feeding, and there was a phenotypic observation of increased lipid accumulation. However, the involvement of the Rhopr-IR pathway activation in regulating nutrient uptake post-feeding is yet to be investigated.

### Exogenous Activation of Rhopr-IR by Mammalian Insulin

**Figure 7:**
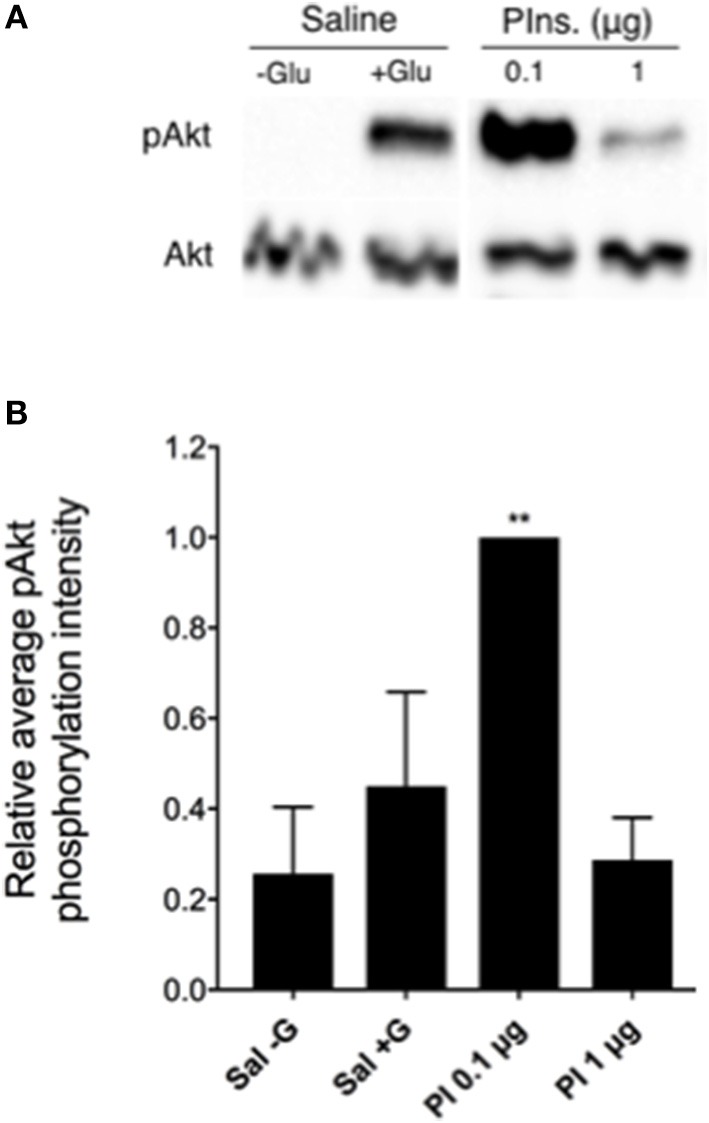
The Rhopr-IR pathway is activated by mammalian insulins. **(A)** After injecting unfed 5th instar insects with either 0.1 or 1 μg porcine insulin (Pins or PI), an increase in the phosphorylation of Akt was detected within fat bodies as observed through Western blot analysis (probing for phospho-Akt and visualized using Chemiluminescence), compared to injection with glucose-free saline. **(B)** Porcine insulin (0.1 μg) significantly stimulated the phosphorylation of Akt relative to saline (with or without glucose) following injection of unfed 5th instar insects and a 30 min incubation with all treatments (*n* = 5 insects per trial). The experiment was repeated 3 times (*n* = 5 insects per trial) and results are shown as means + std error. **p < 0.01 indicates statistically significant difference, inferred using one-way ANOVA with Dunnett's multiple comparisons test.

To further characterize the activity of Rhopr-IR, we investigated the stimulatory effects of mammalian insulin on the IR pathway within *R. prolixus* fat bodies. As seen in Figures 7A,B, injecting 5th instar *R. prolixus* with 0.1 μg porcine insulin led to a significant increase in Akt phosphorylation within 30 min (*n* = 3 trials, with 5 insects per trial), relative to control insects injected with *R. prolixus* glucose-free saline. This increase in pAkt levels would suggest an increase in the activity of other downstream components of the Rhopr-IR pathway, although the phosphorylation of these proteins was not investigated. Higher concentrations of porcine insulin did not appear to stimulate phosphorylation as compared to controls. As expected, injecting insects with glucose-containing saline led to a slight but non-significant increase in fat body Akt phosphorylation (Figures 7A,B), indicating that the IR pathway is also likely responsive to hemolymph glucose fluctuations. However, the full extent of the effects of carbohydrate levels on Rhopr-IR expression and signaling is yet to be investigated.

### Knockdown of Rhopr-IR Expression

RNA interference was used to knockdown the expression of Rhopr-IR (Supplementary Figure 4). Insects were injected with dsIR or control dsARG, and transcript expression was quantified in four different tissues at 3 and 10 days after injections. The CNS and midgut showed a reduction in expression of around 70% by day 3, which was also observed at day 10 post-injection. In the fat body, the expression was knocked down by 40% at day 3 and by 80% at day 10. In contrast to other tissues, the knockdown of expression in the leg muscles at day 3 post-injection was higher than that observed at day 10, which were 50 and 40%, respectively.

Insects injected with dsRNAs were fed on rabbit blood at 3 days post injections in two separate groups, dsARG and dsIR, and were weighed before and after feeding to investigate the effects of Rhopr-IR knockdown on the amount of ingested blood. Although the post-feeding average body weight of Rhopr-IR knockdown insects was slightly lower than that of the control group, no significant difference was observed between the groups (Supplementary Figure 5).

### Metabolic Regulation of Lipids and Carbohydrates in Rhopr-IR Knockdown Insects

**Figure 8:**
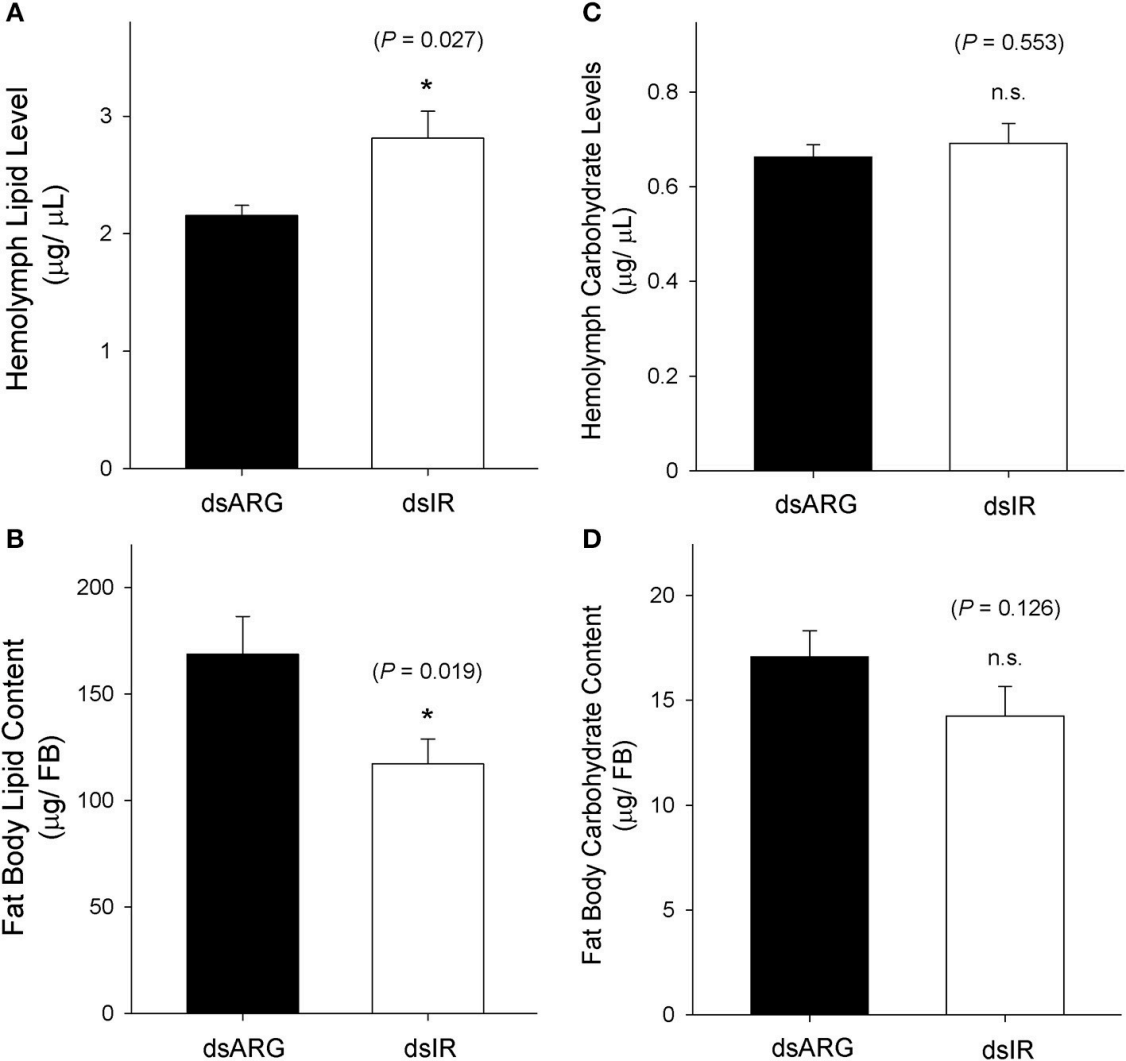
Lipid and carbohydrate measurements of unfed Rhopr-IR knockdown insects. 5th instar *R. prolixus* were injected with 1 μg of dsARG or dsIR in 1 μL of ultrapure water and hemolymph and fat body samples were collected 7 days later. Significance of results is inferred comparing dsIR-injected insects to dsARG-injected insects. **(A)** Increase in hemolymph lipid level in dsIR-injected insects (*n* = 21 dsARG, *n* = 20 dsIR). **(B)** Decrease in fat body lipid content in dsIR-injected insects (*n* = 12 dsARG, *n* = 14 dsIR). **(C)** Hemolymph carbohydrate levels (*n* = 18 dsARG, *n* = 17 dsILP). **(D)** Fat body carbohydrate content (*n* = 16 dsARG, *n* = 17 dsIR). Results are shown as means + std errors. An asterisk (*) indicates statistically significant differences between dsARG- and dsIR-injected insects.

**Figure 9:**
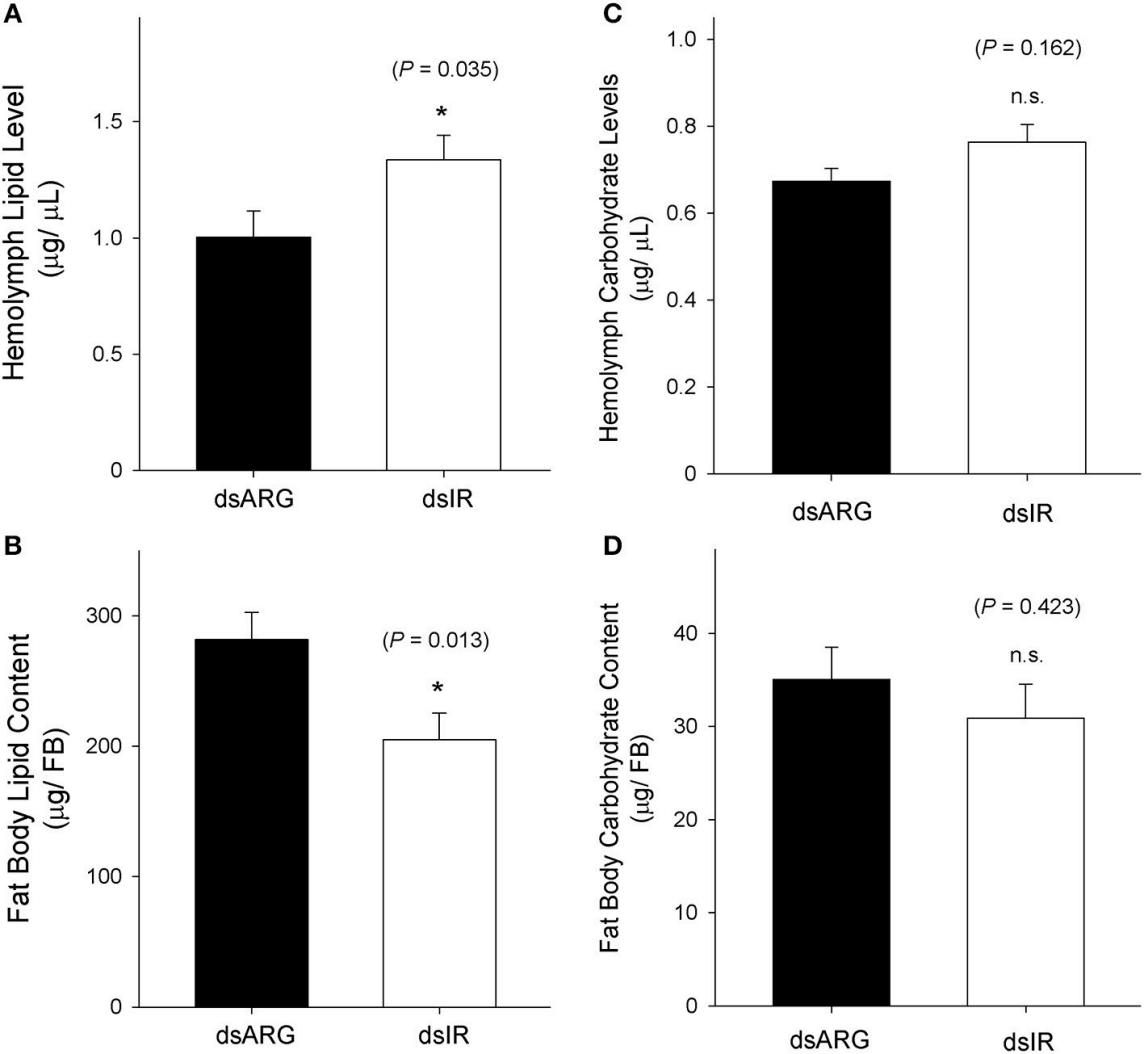
Lipid and carbohydrate measurements in recently fed Rhopr-IR knockdown insects. 5th instar *R. prolixus* were injected with 1 μg of dsARG or dsIR in 1 μL of ultrapure water and fed on defibrinated rabbit blood 3 days after injections. Hemolymph and fat body samples were collected 4 days post-feeding. Significance of results is inferred comparing dsIR-injected insects to dsARG-injected insects. **(A)** Increase in hemolymph lipid level in dsIR-injected insects (*n* = 26 dsARG, *n* = 27 dsIR). **(B)** Decrease in fat body lipid content in dsIR-injected insects (*n* = 25 dsARG, *n* = 20 dsIR). **(C)** Hemolymph carbohydrate levels (*n* = 22 dsARG, *n* = 22 dsIR). **(D)** Fat body carbohydrate content (*n* = 10 dsARG, *n* = 9 dsIR). Results are shown as means + std errors. An asterisk (*) indicates statistically significant differences between dsIR and dsARG injected insects.

The levels of lipids and carbohydrates were measured at 7 days post dsRNA injection in unfed insects and in dsRNA injected insects at 4 days post-feeding. In unfed insects, the hemolymph lipid level increased following the knockdown of Rhopr-IR transcript (Figure 8A), while there was a reduction in the fat body lipid content in knockdown insects compared to the dsARG-injected controls (Figure 8B). In contrast, no differences in carbohydrate levels were observed in either the hemolymph or fat bodies of unfed insects following dsIR or dsARG injections (Figures 8C,D). A similar scenario was observed for recently fed insects, where hemolymph lipid levels increased (Figure 9A) and fat body lipid content decreased in Rhopr-IR knockdowns relative to dsARG controls (Figure 9B). Similar to experiments on dsRNA injected unfed insects, no difference was seen in the hemolymph and fat body carbohydrate content between Rhopr-IR knockdowns and dsARG controls (Figures 9C,D). Our results suggest that Rhopr-IR is involved in fat body lipid storage and hemolymph lipid homeostasis in post-feeding and non-feeding periods. Interestingly, we did not see differences in either stored or circulating carbohydrates between knockdown and control insects.

### Silencing of Rhopr-IR Using DSRNA Suppresses IR Pathway Activation

**Figure 10:**
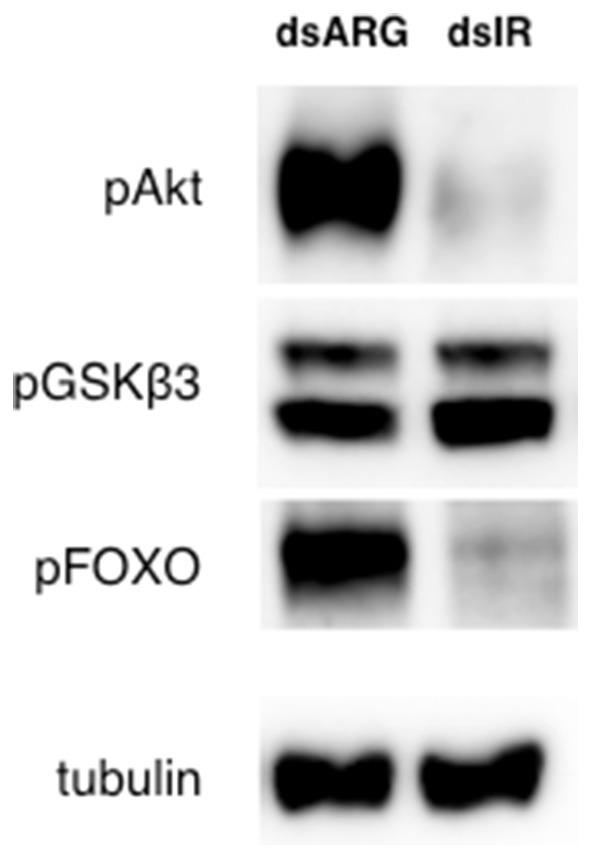
Rhopr-IR knockdown suppresses an IR-associated phosphorylation signaling cascade. The fat bodies of unfed dsIR-injected 5th instar insects displayed decreased Akt and FOXO phosphorylation compared with insects injected with dsARG, when measured 4 days post-injection (*n* = 7 insects per trial). Protein phosphorylation in 5th instar fat bodies was visualized through Western blot analysis, probing for anti-phospho-Akt (pAkt), anti-pGSKβ3 and anti-pFOXO (primary antibodies, 1:1000), and visualized using Chemiluminescence.

Considering the transient knockdown of Rhopr-IR expression observed after treating insects with dsRNA, the effects of dsIR injection on the Rhopr-IR signaling cascade was also investigated. Unfed 5th instars were injected with 1 μg of either dsARG or dsIR and after 4 days, fat bodies were removed and analyzed for the phosphorylation of IR pathway components. As seen in Figure 10, the fat bodies of animals injected with dsIR displayed lower levels of Akt and FOXO phosphorylation compared with dsARG controls, indicating that the transient knockdown of Rhopr-IR likely resulted in reduced IR-related signaling for at least 4 days post-treatment. Interestingly, the phosphorylation state of GSK3β remained unchanged. Overall, these results support the ability of dsRNA to reduce IR expression and related signaling in *R. prolixus* and support the coordinated activity of different upstream and downstream pathway components in mediating IR signaling. Further work should be conducted to determine the prolonged effects of dsIR treatment on fat body IR protein content and associated physiological effects in unfed and fed insects.

## Discussion

The insulin pathway is an evolutionarily conserved cellular signaling system found in all metazoans. The activation of all identified IRs is achieved through the binding of insulin or an ILP, whose sequences can differ markedly between animal classes. However, despite variances in sequence and structure, both vertebrate and insect IRs have been shown to regulate similar phosphorylation cascades that control physiological functions related to macronutrient homeostasis, storage, and mobilization. In insects, the various pathways controlled by IR also influence other processes such as ecdysteroid production (47, 48), ovarian maturation and egg production (19), senescence, and age-related changes in locomotor behavior (49). Although ILPs and IRs have been identified in a variety of invertebrate species, a comprehensive characterization of each pathway component and their role in physiological functions is far from complete.

We have identified an IR in *R. prolixus*, namely Rhopr-IR, which activates conserved intracellular kinases and modulates lipid homeostasis in both post-feeding and non-feeding states. Through the analysis of its sequence, it appears that Rhopr-IR is a glycoprotein composed of two subunits (A and B), and contains the conserved ligand-binding, leucine-rich, cysteine-rich, and kinase domains found in vertebrate and *D. melanogaster* IRs (10, 50). When compared to human IR, one of the most studied IRs (51), Rhopr-IR displays highly conserved structural features, including the extracellular portion that contains cysteine residues that form disulfide bonds in positions homologous to those in human IR (Supplementary Figure 1). Within subunit A, there are 12 predicted disulfide bonds that stabilize each monomer and contribute to the structure of the classical binding surface of insulin. Other predicted disulfide bonds located in the Fibronectin Type III domains connect subunits A and B of each monomer and subunits A of the monomers to form the active dimeric receptor (52). The 1,248-amino acid predicted sequence of Rhopr-IR was compared to IR sequences from 54 other species, including vertebrates and invertebrates. We found that the region with the highest sequence similarity was located in the intracellular tyrosine kinase domain, therefore we analyzed the evolutionary history of Rhopr-IR using this domain, which suggested a close relationship to other hemipteran IRs. It has been previously shown that the intracellular components of the insulin signaling pathway are very conserved throughout evolution in regards to function and sequence (10, 53).

In our investigation, we found that the Rhopr-IR transcript is present in all tissues tested, supporting the idea that the insulin signaling pathway is involved in a variety of physiological processes, as seen in other insects. Previous studies used Northern blot analysis and *in situ* hybridization to identify the expression of an IR within the CNS and ovaries of larval and adult *D. melanogaster*, as well as to localize the receptor within imaginal disks in growing embryos (54). Transcript expression of IR was seen in new and maturing ovary follicles and nurse cells of mosquitoes (19), as well as in ovaries of the honey bee *A. mellifera* (55). Interestingly, there has been very little investigation into the localization of the IR within fat body and skeletal muscles in other insects, although the signaling of ILPs on these and other tissues has been thoroughly characterized, suggesting the presence of both IR transcript and protein. Surprisingly, we have identified a relatively low level of Rhopr-IR transcript expression in the fat body relative to other tissues in unfed 5th instars. It is possible that receptor expression is modulated by nutrient status and intake, and as a result there may be a differential expression pattern observed within the fat body after feeding. It should also be noted that the tissue localization of Rhopr-IR transcript proposed in this study relates to the last nymphal stage of *R. prolixus* and not the adult insect. It is possible that IR transcript expression may differ markedly between these life stages, considering the reproductive activity and multiple feeding cycles associated with the adult stage.

The pharmacological activation of Rhopr-IR was subsequently investigated to classify its function compared with previously identified insect IRs. It is important to reiterate that IR stimulation typically triggers the activation of two main intracellular pathways: the PI3K/Akt/FOXO cascade, and the Ras-MAPK pathway. However, while the PI3K/Akt pathway regulates processes involving glucose uptake and metabolism (56), the IR-dependent MAPK pathway is involved in mediating mitogenic and cell cycle responses in mammals (11), among other functions. For the purposes of the present study, we chose to investigate the PI3K/Akt branch of the IR signaling network overseeing nutrient storage and metabolism, as there is great interest in studying the post-feeding physiological responses of the blood-feeding *R. prolixus* disease vector. However, it would be of interest to further explore the identification of a MAPK-like pathway downstream of the Rhopr-IR and its role in regulating cell growth and proliferation in future research.

In the present work, we chose to first investigate basal IR activation by stimulating receptor activity using the mammalian IR-specific activator BpV(phen), as well as a mammalian insulin. BpV(phen) relieves the negative regulation of basal autophosphorylation of IR by inhibiting PTP1B, a phosphatase that catalyzes the dephosphorylation of the autophosphorylated receptor (42, 57). Consequently, by inhibiting PTP1B, an expected increase in IR-mediated tyrosine phosphorylation is typically observed, as well as the increased phosphorylation of proteins containing pTyr sites downstream of the IR receptor (43). We observed both an increase in general tyrosine phosphorylation as well as phosphorylation of the kinase Akt, which is a conserved component of IR signaling in mammals, *C. elegans*, and *D. melanogaster* (58). The coordinated increase in tyrosine and Akt phosphorylation reinforce our characterization of Rhopr-IR as a receptor tyrosine kinase, similar to the IRs identified for vertebrate insulins and other insect ILPs. Stimulation of insect IR-like pathways by other pervanadate compounds [similar to BpV(phen)] have also been reported in the mosquito *A. aegypti* (47), where an increase in ecdysteroid production, normally controlled by the insulin pathway, was observed. Thus, as a result of the stimulation of IR-like signaling induced by the injection of insects with BpV(phen), we believe that the mechanisms controlling basal IR activity in insects is similar to what is seen in mammals, and that bisperoxovanadium compounds are capable of stimulating IR-like receptors in *R. prolixus* as well as other insect species.

It should be noted that BpV(phen) activates IR through an indirect mechanism and does not act on the receptor itself, as previous studies identified molecular iterations of BpV compounds as potent inhibitors of the tumor suppressor phosphatase phosphatidylinositol 3,4,5-triphosphate 3-phosphatase (PTEN) (59). Hence, in order to support the observed increase in the phosphorylation of downstream IR proteins (such as Akt) when treated with BpV(phen), we furthered our investigation of Rhopr-IR activity by injecting insects with porcine insulin and evaluated the responsiveness of its potential downstream pathway components. We previously reported the effects of porcine insulin on the modulation of nutrient balance in the locust *L. migratoria* (60). Furthermore, porcine insulin was also found to dose-dependently stimulate the phosphorylation of embryo-specific proteins in *D. melanogaster* (50) and to directly compete with an ILP from *Manduca sexta* in binding to an insulin antibody (61). Other studies have investigated the ability of bovine insulin to stimulate ecdysteroid synthesis in *B. mori* (48), and to stimulate a decrease in circulating carbohydrates in the cockroach *Periplaneta americana* (62). Here, we observed that treatment with porcine insulin led to an increase in the phosphorylation of Akt in the fat body of *R. prolixus* after a half-hour incubation. Interestingly, injecting insects with larger amounts of insulin resulted in a decrease in the detected phosphorylation of Rhopr-IR pathway components. It is possible that Rhopr-IR experienced desensitization when stimulated by higher concentrations of porcine insulin, leading to an observed decrease in pathway activation. This hypothesis is supported by a previous study conducted in isolated rat adipocytes, where an apparent time-dependent and dose-dependent decrease in IR proteins was observed after treatment with large insulin concentrations for 4 h, coupled with a measurable decrease in the insulin-dependent glucose transport (63).

Alternatively, it is possible that larger concentrations of insulin stimulated the maximal activation of pathway phosphorylation in a shorter timeframe, which may not have been captured by the 30-min time point used in this study. Through kinetic analysis, it has been found that insulin induced 50% maximum stimulation of IR autophosphorylation within 30 s of incubation with partially purified IR β-subunit, with near-maximal activation achieved after a 5-min incubation (64). This study further observed a dose-dependent inhibition of the autophosphorylation of the IR β-subunit by concentrations of insulin < 100 nM. Therefore, it is likely that porcine insulin was capable of activating Rhopr-IR and stimulating the ILP-responsive pathway as seen in other insects but induced either receptor sensitization or rapid kinetic effects not detectable by the methodology used in the present study. The receptor-ligand specificity of insect IRs, and specifically Rhopr-IR, for vertebrate insulins and insulin-like ligands should be further investigated.

We further sought to map a potential signaling cascade triggered by Rhopr-IR by investigating the timing and coordination in phosphorylation of components downstream of Akt. The phosphorylation of Akt and its substrates GSK3β and FOXO was barely detectable using Western blot analysis in unfed 5th instars, whereas feeding on a blood meal triggered a coordinated increase in the phosphorylation of all three proteins. As FOXO is the most downstream target of the pathway, it was unsurprising that peak levels of phosphorylation appeared to be reached 2 days post-feeding, while maximum phosphorylation of Akt and GSK3β was observed 1 day after feeding. Previously, it was observed that the maximum release of ILPs into circulating hemolymph may be reached at 3 to 4 days post-feeding in *R. prolixus*, as monitored through immunofluorescence (46). Thus, the coordinated phosphorylation of Akt, GSK3β, and FOXO in the present study implies an increase in the activity of the IR within the same time frame as putative endogenous ILP release following feeding. These three protein targets are part of the PI3K/Akt/PKB pathway, which is stimulated by vertebrate IRs and other insect IRs such as within the plant hopper *Nilaparvata lugens* (24), the mosquito *A. aegypti* (65), the moth *B. mori* (66), and *D. melanogaster* (67–69). Along these lines, we believe that Rhopr-IR shares the conserved PI3K/Akt signaling cascade observed in other insulin-mediated pathways.

However, it should be noted that Akt activation and signaling is not exclusive to the PI3K/Akt axis of the IR pathway. In mammals, the activity of Akt has been implicated in several diverse cellular processes including cell survival (due to the inhibition of the apoptosis-inducing FOXO transcription factor), cell growth, proliferation, metabolism, cell migration, and cancer proliferation (70, 71). Aside from IR-mediated stimulation, the phosphorylation and activation of Akt is also regulated by G-protein coupled receptors (GPCRs), as observed in mammals through a muscarinic acetylcholine GPCR whose Akt-related signaling mediates mammalian cell survival (72). It has also been found that ecdysteroids and ecdysteroid receptor signaling also regulate Akt activation and overall IR pathway signaling in *Drosophila* (73), in relation to coordinating physiological responses to insect growth and development. Stimulation of ecdysteroidogenesis in *B. mori* is also partially accomplished by Akt activation mediated by the prothoracicotropic hormone (PTTH) signaling at its receptor, PTTH-R (74). Furthermore, IGF signaling in both mammals and invertebrates also triggers the activation of the PI3K/Akt pathway (18, 75). Similar to IR stimulation, the activity of IGF at its cognate tyrosine kinase receptor (IGF-1R) also regulates metabolism and cell survival, as well as development-related growth, aging, and skeletal muscle atrophy and growth (76–79). Thus, it is possible that the observed activation of pAkt levels in the present study could have been influenced by one or several of these competing pathways. Moving forwards, we believe that the signaling induced by Rhopr-ILP and -IGF at the Rhopr-IR within the fat body, and particularly the effects of these signaling molecules on Akt activation and pathway response to nutrient sensing and storage in *R. prolixus*, should be investigated.

Physiologically, the coordinated phosphorylation of Akt, GSK3β, and FOXO mediated by the activation of IR signaling results in cell growth, glucose uptake and increased nutrient storage, as observed in mammalian cells and in *Drosophila* (69, 80–84). The serine/threonine kinase Akt is activated upon its phosphorylation, resulting in the pAkt-mediated phosphorylation and inactivation of GSK3β (15, 85) and FOXO (86). The inhibition of GSK3β stimulates glycogen synthesis, lipid and glucose storage, while the inhibition of FOXO prevents the transcriptional promotion of apoptosis and allows for cell growth, as observed in *D. melanogaster* (69). We observed that the post-feeding increase in Rhopr-IR pathway activity also coincides with an increase in fat body weight and apparent lipid accumulation. By silencing the expression of Rhopr-IR using dsRNA, we detected a decrease in Akt and FOXO phosphorylation in unfed insects 4 days post-injection, which also correlated with a decrease in fat body lipid content and increase in hemolymph triglyceride levels. This effect of Rhopr-IR knockdown on circulating and stored lipids was repeated in recently fed insects, and it was observed that dsIR-injected animals had significantly less lipid stored in the fat body and more lipids in their hemolymph when compared to dsARG-injected controls.

As there was no apparent effect of dsRNA injection on the amount of blood ingested by insects, it is likely that dsIR knockdown partially silenced the Rhopr-IR signaling leading to the depressed activation of Akt and resulting increased activity of FOXO. Without the insulin signal, FOXO remains active in the nucleus and stimulates the expression of a variety of genes such as PEPCK (phosphoenolpyruvate carboxykinase), a key enzyme involved in propagating the gluconeogenesis signal in the absence of stored nutrients (87). In *D. melanogaster*, FOXO stimulates the expression of the triacylglycerol (TAG) lipase gene *dLip4* (88) and the mitochondrial acyl-CoA synthetase gene *pudgy* (89), which are directly involved in lipid metabolism. In *D. melanogaster*, it was also observed that the expression of a constitutively active FOXO homolog lacking its regulatory Akt phosphorylation sites resulted in the suppression of IR-induced triglyceride storage (69), rendering similar results to our observations of reduced lipid storage in Rhopr-IR knockdown insects. Furthermore, it was observed that treatment of diapausing *A. aegypti* females with dsFOXO resulted in significantly lower lipid accumulation compared with untreated controls, and there was a noticeable reduction in the number of fat body cells in dsFOXO animals (90). The cellular apoptotic and anti-proliferative activities mediated by FOXO most likely counteract fat body cell growth and ultimately work to reduce lipid storage. Therefore, arresting FOXO function by activating Rhopr-IR and Akt results in fat body cell growth and lipid accumulation. Future work should focus on simultaneously monitoring FOXO phosphorylation and transcriptional activity along with cell growth and lipid storage, in order to corroborate this hypothesis.

Contrasting the lipid imbalance observed between fat body and hemolymph, we did not see any changes in carbohydrate distribution post Rhopr-IR knockdown, either in unfed or in recently fed insects. Additionally, GSKβ3 phosphorylation levels in the fat body of unfed insects were not drastically altered by dsRNA injections, suggesting that the reduction in transcript expression did not interfere in the signaling that regulates carbohydrate homeostasis. Although GSKβ3 is a substrate for Akt and therefore has its activity modulated by this kinase, it has been shown that PKA (protein kinase A) and other kinases in the Wnt pathway can also phosphorylate and inactivate this enzyme (91, 92). GSKβ3 was proven to be essential for glycogen metabolism but not lipogenesis during oogenesis and embryogenesis in *R. prolixus*, in response to both insulin and Wnt receptors (93). Also, triatomine insects, such as *R. prolixus*, mainly rely on lipids as their motor energy supply, having a remarkably low concentration of sugars in the hemolymph when compared to other insects (94). Thus, it is interesting yet unsurprising that silencing Rhopr-IR yields a more sensitive response in regulating the homeostasis of lipid levels and related signaling cascades, with a smaller effect observed on pathway components that regulate carbohydrate nutrient balance. We have recently reported the presence of two ILPs in *R. prolixus*, Rhopr-ILP and Rhopr-IGF, and their involvement in energy homeostasis. We observed that both hormones are involved in the control of lipid and carbohydrate levels in the hemolymph, but only Rhopr-ILP seems to be involved in the regulation of lipid and carbohydrate content in the fat body (32, 33). It has been shown that insulin signaling in *D. melanogaster* adult adipocytes can activate GSKβ3 independently from FOXO depending on developmental stage (95). We could speculate that knocking down the expression of Rhopr-IR, but not its ligands, is reducing lipid uptake while increasing lipolysis through FOXO activation. At the same time, the lower Rhopr-IR expression levels could be promoting carbohydrate homeostasis by preventing GSKβ3 activation.

In conclusion, we have identified a candidate IR sequence in *R. prolixus* genome, namely Rhopr-IR, that is expressed in all tissues tested, suggesting that this receptor is likely involved in a variety of physiological processes. This work also demonstrates that a mammalian insulin and an IR activator have the ability to trigger an intracellular phosphorylation cascade, indicating that the conserved domains of Rhopr-IR can interact with mammalian activators. These observations further support the likely conserved nature of IR signaling between mammals and insects. Furthermore, Rhopr-IR regulates lipid homeostasis in hemolymph and fat body likely through the modulation of pathway protein activity including the kinase Akt and the transcription factor FOXO, as seen in vertebrate and invertebrate models. Our results contribute to the understanding of energy homeostasis control in *R. prolixus* as well as to the characterization of the evolutionarily conserved insulin signaling system in insects.

## Author Contributions

MD and SD designed the study, conducted the experiments and wrote the manuscript. IO and AL aided in the design of the study, revised the manuscript, and supervised the work.

### Conflict of Interest Statement

The authors declare that the research was conducted in the absence of any commercial or financial relationships that could be construed as a potential conflict of interest.
