# Supplementary material for: A Rhodnius prolixus Insulin Receptor and Its Conserved Intracellular Signaling Pathway and Regulation of Metabolism
Source: Front Endocrinol (Lausanne). 2018 Dec 6;9:745. doi: 10.3389/fendo.2018.00745 (PMC6291494; doi:10.3389/fendo.2018.00745)
Supplement: Supplementary file 1 [file Data_Sheet_1.PDF]

Supplementary Table 1. Gene specific primers.

**quantitative PCR – 5'-3' 157 bp amplicon**	
IR qPCR Forward	AGCTCCCAGATTGTCTACGG
IR qPCR Reverse	CCGGGTCGAATCAACTAGG
**dsRNA – 5'-3' 475 bp amplicon**	
IR Forward	GAGATGATTGTTTATTGGGTGC
T7-IR Forward	**TAATACGACTCACTATAGGGAGA**GAGATGATTGTTTATTGGGTGC*
IR Reverse	GGGTGCAATTTGATCCCAGT
T7-IR Reverse	**TAATACGACTCACTATAGGGAGA**GGGTGCAATTTGATCCCAGT*

* **TAATACGACTCACTATAGGGAGA** = T7 RNA polymerase promoter

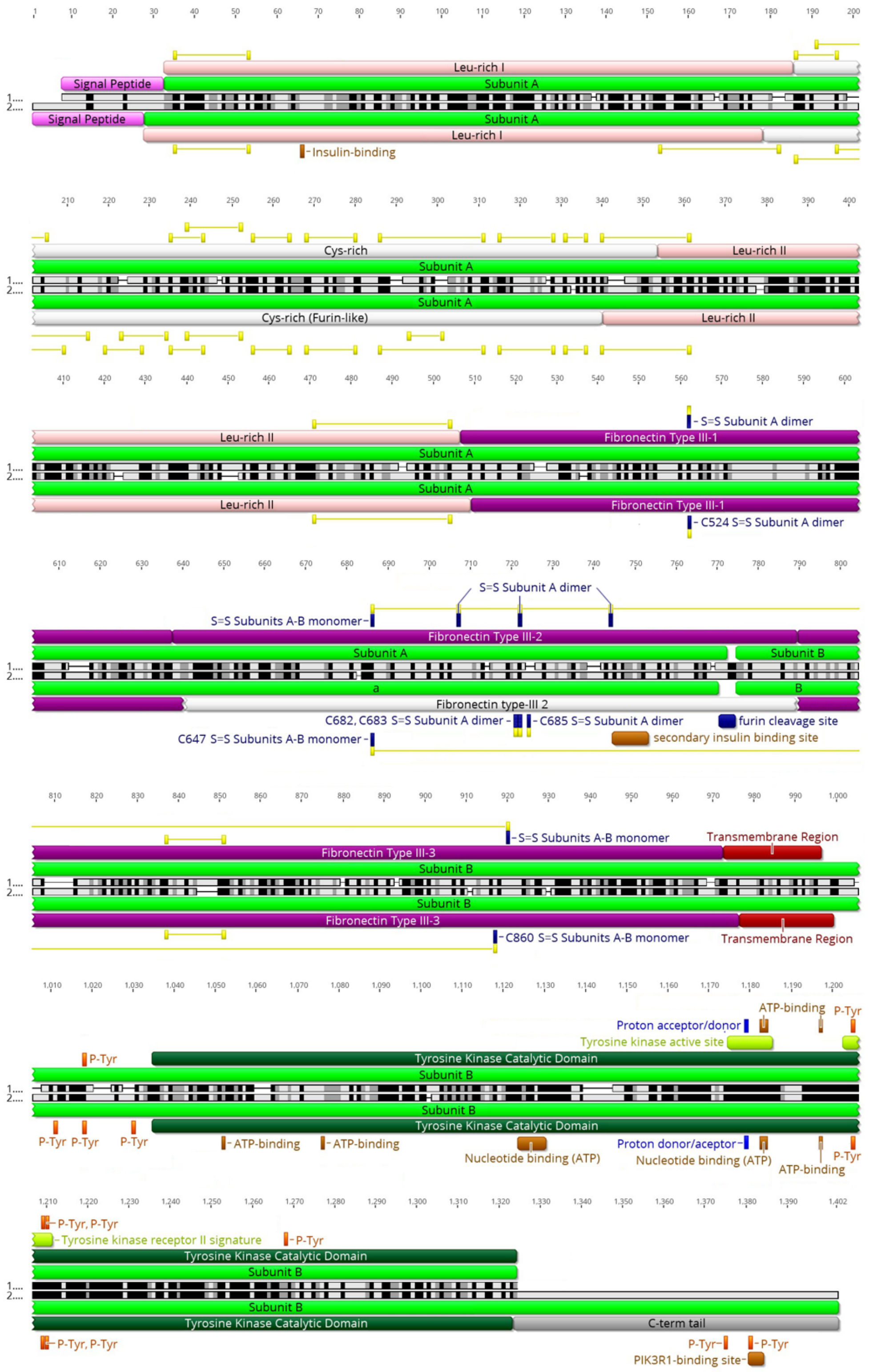

Supplementary Figure 1. Graphic alignment highlighting the structural similarities between Rhopr-IR and human IR. The IR monomers were aligned, and subunits A and B are highlighted by green and purple boxes, respectively. Signal peptides are highlighted in bright pink, followed by Leu-rich domains highlighted in pale pink and Cys-rich domains in light grey. Disulfide bonds are identified by yellow boxes over Cys residues, connected by yellow lines when the bonds are formed within the monomer. Fibronectin Type-III domains are

indicated by purple and grey boxes. The Cys residues that form disulfide bonds involved in the dimerization of the receptor are indicated by yellow/dark blue boxes over said residues. The Cys residues that form the disulfide bonds between subunits A and B of each monomer, are indicated by yellow/dark blue boxes over the residues and connected by yellow lines. The transmembrane regions are highlighted by red boxes, and the Tyr kinase catalytic domain by dark green boxes. Tyr residues that are phosphorylated upon activation of the receptor are indicated with small orange boxes. ATP-binding sites between the active site and the receptor signature are indicated with brown boxes over the residues.

CLUSTAL multiple sequence alignment by MUSCLE (3.8)

```
Drosophila_melanogaster            -MFNMPRGVTKSKSKRGKIKMENDMAAAAATTAKTTACTLGHICVLCRQEMLLDTCCCRQ
Halyomorpha_halys                  ------------------------------------------------------------
Rhodnius_prolixus                  ------------------------------------------------------------
Cimex_lectularius_X1               MNGMRPRNSMEGGAWWGVGGGHGGPSLLAVHQCTLLPES---------------------
C._lectularius_X2                  ------------------------------------------------------------
Cryptotermes_secundus              ------------------------------------------------------------
Miniopterus_natalensis             ------------------------------------------------------------
Callithrix_jacchus                 ------------------------------------------------------------
Heterocephalus_glaber_X1           ------------------------------------------------------------
Dasypus_novemcinctus               ------------------------------------------------------------
Tupaia_chinensis_X2                ------------------------------------------------------------
Homo_sapiens_X1                    ------------------------------------------------------------
Pongo_abelii_2                     ------------------------------------------------------------
Pan_troglodytes_X2                 ------------------------------------------------------------
Boleophthalmus_pectinirostris      ------------------------------------------------------------
Larimichthys_crocea_X2             ------------------------------------------------------------
Larimichthys_crocea                ------------------------------------------------------------
Lates_calcarifer                   ------------------------------------------------------------
Seriola_lalandi_dorsalis           ------------------------------------------------------------
Seriola_dumerili                   ------------------------------------------------------------
Zootermopsis_nevadensis            ------------------------------------------------------------
Parasteatoda_tepidariorum          ------------------------------------------------------------
Onthophagus_nigriventris           ------DACSERKA----------------------------------------------
Nicrophorus_vespilloides           ------------------------------------------------------------
Tribolium_castaneum                ------------------------------------------------------------
Nilaparvata_lugens                 ------------------------------------------------------------
Blattella_germanica                ------------------------------------------------------------
Nasonia_vitripennis_X1             ----------MSNLHRSTMTTTTSGCIET-------------------------------
Microplitis_demolitor              ----------MKSSVISKKKEKDKKEIML-------------------------------
Neodiprion_lecontei                MGRRTVNNMRWREHQR----NENDDGSVATTTTKTANESSTSI-----------------
Athalia_rosae                      -MMKNSRRLQRRNSKNCSTSSSTDDDRVNGVGSVMTRNFNAIKKSNGINDDDEDCNNDVN
Orussus_abietinus                  -------MKPCESIGSPVAVNWDACSAPNYDT------PIKPADDGE-------------
Dufourea_novaeangliae              ---MKSAAWCTRRLGSASSVDDSADASPPEVYTTTSVRCSNIAAATTRISCGVTAIVDAA
Megachile_rotundata_X1             ------------------------------------------------------------
Melipona_quadrifasciata            ------------------------------------------------------------
Apis_mellifera_X1                  --MEPSTWCTRRLGGGVATSAESTIAVTLAKLGATTTSSRLSSDASAIIPQPD-------
Eufriesea_mexicana_1               --MESPTWCTRRLGGSSSAIEESSSSRLSIASNPVNDRSSETDTTPC-------------
Eufriesea_mexicana_2               ------------------------------------------------------------
Bombus_terrestris                  --MEPLTWCTRRLGGSVAASEESTIAVTLAKVAATTSSRLSTNANPVNDCSSTTTWICET
Bombus_impatiens                   --MEPLTWCTRRLGGSVAASEESTIAVTLAKVAATTSSRLSTNANPVNDCSSTTTWICGT
Polistes_dominula_X1               -------MYFSRRMRRMEIHEELNRNSSSSNSSSSSSSSSSSSRKSTRSTTRSISSTLDD
Cephus_cinctus_X1                  ---MTLTDCSSLLTGNCLEYS---------------------------------------
Dinoponera_quadriceps_X2           MGKMKSAACCTRRLAGTATFDEDE-PSAV--P------SSSEARSNT-------------
Harpegnathos_saltator_X1           MVKMKSAACCTRRLRGTATFDEDESPRAVSPS------PSPKIRSNT-------------
Linepithema_humile_X2              ------------------------------------------------------------
Linepithema_humile_X1              ------------------------------------------------------------
Ooceraea_biroi                     --MKSSAARYRRK-SRGATFDEDEPSTTT--PSSCSCSSSSEAHSTN-------------
Camponotus_floridanus              --MKSPA--FACRKNRSATFDVDDEPYAS--A------ASSSSSETLLVTR---------
Solenopsis_invicta                 --MKSSAACYARR-FGGVTFDENESSIVA--P------FLSEANSTT-------------
Pogonomyrmex_barbatus_X1           --MKSSAACR----LCGATFDEDESSIAS--T------SFPEADHAI-------------
Vollenhovia_emeryi_X1              --MNFSGACHARR----LGSVDESSTVAA--L------SSSEANFST-------------
Wasmannia_auropunctata             --MKSSAACYTARRLRGATFDEDVPSIVA--S------SFSEANSTT-------------
Cyphomyrmex_costatus               ------------------------------------------------------------
Trachymyrmex_septentrionalis       --MKSSAACYTSRGLRDAMFDEGERSVVA--S------SFWEVDSTT-------------
Atta_colombica                     --------------------------------------MISETKEAS-------------
Trachymyrmex_cornetzi              --MKFSATCYTRR-LRGVTFDEDERSIVA--S------SFSEVNSTT-------------
Acromyrmex_echinatior              --MKSSATCYTRR-LRGATFDEDKQNIVA--T------SFWEVNSTT-------------
Atta_cephalotes                    --MKSSATCYTRR-LRGATFDEDEQNIVA--T------SFWEVNSTT-------------


Drosophila_melanogaster            AVEAVDSPASSEEAYSSSNS-------SSCQASSEISAEEVWFLSHDDIVLCRRPKFDEV
Halyomorpha_halys                  ------------------------------------------------------------
Rhodnius_prolixus                  ------------------------------------------------------------
Cimex_lectularius_X1               ------------------------------------------------------------
C._lectularius_X2                  ------------------------------------------------------------
Cryptotermes_secundus              ------------------------------------------------------------
Miniopterus_natalensis             ------------------------------------------------------------
Callithrix_jacchus                 ------------------------------------------------------------
```

```
Heterocephalus_glaber_X1          ------------------------------------------------------------
Dasypus_novemcinctus              ------------------------------------------------------------
Tupaia_chinensis_X2               ------------------------------------------------------------
Homo_sapiens_X1                   ------------------------------------------------------------
Pongo_abelii_2                    ------------------------------------------------------------
Pan_troglodytes_X2                ------------------------------------------------------------
Boleophthalmus_pectinirostris     ------------------------------------------------------------
Larimichthys_crocea_X2            ------------------------------------------------------------
Larimichthys_crocea               ------------------------------------------------------------
Lates_calcarifer                  ------------------------------------------------------------
Seriola_lalandi_dorsalis          ------------------------------------------------------------
Seriola_dumerili                  ------------------------------------------------------------
Zootermopsis_nevadensis           ------------------------------------------------------------
Parasteatoda_tepidariorum         ------------------------------------------------------------
Onthophagus_nigriventris          ------------------------------------------------------------
Nicrophorus_vespilloides          ------------------------------------------------------------
Tribolium_castaneum               ------------------------------------------------------------
Nilaparvata_lugens                ------------------------------------------------------------
Blattella_germanica               ------------------------------------------------------------
Nasonia_vitripennis_X1            ---------------SSCRC-------CCCSCG-----------------RELSD-----
Microplitis_demolitor             ---------------------------GCPNCLVHL--------------ELSTT-----
Neodiprion_lecontei               ---------------LNSLC------YSCRIC----------QADEAGRDKNENT-----
Athalia_rosae                     TKVVTSSSTSASEDDVENFCDKGAKVFQCRNCSPGTALGEELDNEEYGRAIDGSSPLTGR
Orussus_abietinus                 ------------------NY-------SCSNCLALQ--------------VKVTEDFVDS
Dufourea_novaeangliae             TTGCAVTTTNTSTTTCHCHCPSGRDTLECADPSHFLSSSSTMSSASTGPNRTTT------
Megachile_rotundata_X1            ------------------------------------------------------------
Melipona_quadrifasciata           ------------------------------------------------------------
Apis_mellifera_X1                 ---------------------------PCPSSSPQH------------------------
Eufriesea_mexicana_1              ---LADTSADASSASCHCHCSSGCVATRSHSWSSRL------------------------
Eufriesea_mexicana_2              ------------------------------------------------------------
Bombus_terrestris                 TCRLTDTSEDANSVLRHCHC--------------------------------WSGSATPG
Bombus_impatiens                  TCRLTDTSEDANSVLRHCHC--------------------------------WSSSATPG
Polistes_dominula_X1              QQSTTANFCCPSGGYCFCCC-------CCRCSNGVTMNNKECKMNNMEEHENKDDCIINN
Cephus_cinctus_X1                 ------------------------------------------------------------
Dinoponera_quadriceps_X2          ---------------CHCHC-------P-WSRGAFE--------------EALA------
Harpegnathos_saltator_X1          ---------------CHCHC-------P-WSRGAFE--------------DALA------
Linepithema_humile_X2             ------------------------------------------------------------
Linepithema_humile_X1             ------------------------------------------------------------
Ooceraea_biroi                    ---------------CHCHC-------PLSSRGVFE--------------DVDGVPAAWT
Camponotus_floridanus             ---------------RYCHC-------P-SICGAFE--------------DTSNDASSQS
Solenopsis_invicta                ---------------CHCHC-------P---CDAFE--------------RDDGASSL--
Pogonomyrmex_barbatus_X1          ---------------RHCHC-------LSSSSREFE--------------KTDGASF-PS
Vollenhovia_emeryi_X1             ---------------CHCHC-------PSSSCGAFE--------------KDDGVSSLPS
Wasmannia_auropunctata            ---------------CHCQC-------PSSSCGAFE--------------KDDGVSFLPS
Cyphomyrmex_costatus              ------------------------------------------------------------
Trachymyrmex_septentrionalis      ---------------CHCHC-------PSSSCGAFG--------------KDDGVSFPPS
Atta_colombica                    ---------------CSLMH-------RLNCC----------------------TLLLP-
Trachymyrmex_cornetzi             ---------------CHCHC-------PSSSCGAFE--------------KDDGISFLPS
Acromyrmex_echinatior             ---------------CHYHY-------PSSSCGAFE--------------KDDGVSFLPS
Atta_cephalotes                   ---------------CHYHY-------PSSNCGAFE--------------KDDGISFLPS


Drosophila_melanogaster           ETTGKKRDVKCSGHQCSNECDDGSTKNNRQQRENFNIFSNCHNI----------------
Halyomorpha_halys                 ------------------------------------------------------------
Rhodnius_prolixus                 ------------------------------------------------------------
Cimex_lectularius_X1              ---------------LLHLAQVRDRVAALR------------------------------
C._lectularius_X2                 ------------------------------------------------------------
Cryptotermes_secundus             ------------------------------------------------------------
Miniopterus_natalensis            -------------MGTLGICEGENRLAEKPLPLPGESVGP--------------------
Callithrix_jacchus                ------------------------------------------------------------
Heterocephalus_glaber_X1          ------------------------------------------------------------
Dasypus_novemcinctus              ------------------------------------------------------------
Tupaia_chinensis_X2               ------------------------------------------------------------
Homo_sapiens_X1                   ------------------------------------------------------------
Pongo_abelii_2                    ------------------------------------------------------------
Pan_troglodytes_X2                ------------------------------------------------------------
Boleophthalmus_pectinirostris     ------------------------------------------------------------
Larimichthys_crocea_X2            ------------------------------------------------------------
Larimichthys_crocea               ------------------------------------------------------------
Lates_calcarifer                  ------------------------------------------------------------
Seriola_lalandi_dorsalis          ------------------------------------------------------------
```

```
Seriola_dumerili                ------------------------------------------------------------
Zootermopsis_nevadensis         ------------------------------------------------------------
Parasteatoda_tepidariorum       -------------------MVVIVIFKGEAAMG---------------------------
Onthophagus_nigriventris        ---------------GPLLCRISHPMASKSICG---------------------------
Nicrophorus_vespilloides        ------------------------------------------------------------
Tribolium_castaneum             ------------------------------------------------------------
Nilaparvata_lugens              -----------MEDSACPGCSLRPTNVGRR------------------------------
Blattella_germanica             ------------------------------------------------------------
Nasonia_vitripennis_X1          ---------------AARLCPLCQRLQHDS--------KACGDE----------------
Microplitis_demolitor           ---------------FTDQCQLCDRLCNES-----KSVTFIIQK----------------
Neodiprion_lecontei             VG---------YR------CEICERLQGSVGDDDVDDLSTNEE-----------------
Athalia_rosae                   LG---------HRQKPQQQCELCERLQGCESKDKSSDVDGNERRGAGGGAGAGAGGGECC
Orussus_abietinus               ---------------EFLQCDICKRLRNSA----ENHEEGCNEV----------------
Dufourea_novaeangliae           ---------------ISNSCELCERLRGRC------------------------------
Megachile_rotundata_X1          ----------------MTSCGTSNRVTDPRTLS---------------------------
Melipona_quadrifasciata         ------------------------------------------------------------
Apis_mellifera_X1               ----------HDHGPFPSLCDLCERLRGGRLVSGGRSETDEEEE----------------
Eufriesea_mexicana_1            --------------NRSNVCDLCERLRGRS------------------------------
Eufriesea_mexicana_2            ------------------------------------------------------------
Bombus_terrestris               HDGPPLSLFLPNRQNQSNVCDLCERLRGRS------------------------------
Bombus_impatiens                HDGPPLSLFLPNRQNQSNVCDLCERLRGRS------------------------------
Polistes_dominula_X1            NN--------NHHQFATRTCVLCERLHGSSLNSSDHRFDEIDTNYYNN------------
Cephus_cinctus_X1               ---------------SRLRCEICKRLCPTE------------------------------
Dinoponera_quadriceps_X2        ---------------SPASTSMCDRLRDRT--D----RDLLDVI----------------
Harpegnathos_saltator_X1        ---------------SPSTTSVCERLRHRT--D----RDLLGAI----------------
Linepithema_humile_X2           -----------------MACDLCERLRGRT--D--GHFDLVDAI----------------
Linepithema_humile_X1           -----------------MACDLCERLRGRT--D--GHFDLVDAI----------------
Ooceraea_biroi                  ---------------SAVTCDLCERLRGRT--G--RRSDPVDAT----------------
Camponotus_floridanus           PS--------ESTSMSTTTCDLCERLRGQT--D--GCSDTMDAI----------------
Solenopsis_invicta              ----------------STACDLCERLRGQT--G--RCYDSVDRVDRVD------------
Pogonomyrmex_barbatus_X1        TS-------------ASTTCDLCERLRGRT--GGSNASSDVSAI----------------
Vollenhovia_emeryi_X1           PA-------------PATACDLCERLRG----C----YDSVDAT----------------
Wasmannia_auropunctata          PV-------------TTTACDLCVRLRGQT--S----YDSVDAI----------------
Cyphomyrmex_costatus            -----------ISKTKEASCSLMHRL----------------------------------
Trachymyrmex_septentrionalis    SA-------------STTACDLCERLRGQT--G----YEPVNAT----------------
Atta_colombica                  ------------------------------------------------------------
Trachymyrmex_cornetzi           PA-------------STITCDLCERLRGQT--G----YESVNAI----------------
Acromyrmex_echinatior           SA-------------STTACDLCERLRGQT--G----YESVNAI----------------
Atta_cephalotes                 PA-------------STTACDLCERLRGQT--G----YESVNAV----------------


Drosophila_melanogaster         ----LRTLHSLLLLMFNCGIFNKRRRRQHQQQHHHHYQHHHQQH-------HQQHLQRQQ
Halyomorpha_halys               ------------------------------------------------------------
Rhodnius_prolixus               ------------------------------------------------------------
Cimex_lectularius_X1            -----------------------RRAQHARVNSLLRDSAPTKQN---------------S
C._lectularius_X2               ------------------------------------------------------------
Cryptotermes_secundus           ------------------------------------------------------------
Miniopterus_natalensis          -----------------------RSSVHEAGPATNSSNPPIACE----------------
Callithrix_jacchus              ------------------------------------------------------------
Heterocephalus_glaber_X1        ------------------------------------------------------------
Dasypus_novemcinctus            ------------------------------------------------------------
Tupaia_chinensis_X2             ------------------------------------------------------------
Homo_sapiens_X1                 ------------------------------------------------------------
Pongo_abelii_2                  ------------------------------------------------------------
Pan_troglodytes_X2              ------------------------------------------------------------
Boleophthalmus_pectinirostris   ------------------------------------------------------------
Larimichthys_crocea_X2          ------------------------------------------------------------
Larimichthys_crocea             ------------------------------------------------------------
Lates_calcarifer                ------------------------------------------------------------
Seriola_lalandi_dorsalis        ------------------------------------------------------------
Seriola_dumerili                ------------------------------------------------------------
Zootermopsis_nevadensis         ------------------------------------------------------------
Parasteatoda_tepidariorum       -----------------------RRSMPP-------------------------------
Onthophagus_nigriventris        ------------------------------------------------------------
Nicrophorus_vespilloides        ------------------------------------------------------------
Tribolium_castaneum             ------------------------------------------------------------
Nilaparvata_lugens              ------------------------------------------------------------
Blattella_germanica             ------------------------------------------------------------
Nasonia_vitripennis_X1          -----------------------RRGSRRKNHHQREDSGRRR---------KYDDNTEKD
Microplitis_demolitor           ------------------------------NKSSMNDKSRVKTD-------WRSDKVTLI
Neodiprion_lecontei             -EEKKKKDEEDHRG---------KRRSRLFSRGFFNASSKLEPL-------AMDCGRRQS
```

```
Athalia_rosae                   YTDGRRNRNNGH-----------QTKRGSKEYGDVNDGCTGHPMDPVSPPSPMDCGRRRC
Orussus_abietinus               ------------------------SYRQGYLQRLFPAFSSGKIG-------ST------C
Dufourea_novaeangliae           -------------------DSPERRTIGDRETIRRGEHVSRDPDHAMTLSRTFEERDRFC
Megachile_rotundata_X1          ------------------------------------------------------------
Melipona_quadrifasciata         ------------------------------------------------------------
Apis_mellifera_X1               ------------------G----GRGRMTRRGRSVADRGHASPAGVVSAKDRR-------
Eufriesea_mexicana_1            -----------------------RNCSTLDGTAEEGRFDRAPRREQRRPLVPHADHAVAT
Eufriesea_mexicana_2            ------------------------------------------------------------
Bombus_terrestris               -----------------------CDYSTLDEKSTEGRFDRITRRGQRQVVNRDVNHTMIS
Bombus_impatiens                -----------------------CDYSTLDEKSTEGRFDRITRRGQRQVVNRDVNHTMIS
Polistes_dominula_X1            -SSSTSSSSSSSTS----TSTSSSTSSSPKYCRSFYEVSSKSIR-------RKDDKDKWI
Cephus_cinctus_X1               ---------------------------------EPEEEHPTKSSRQIPCKHKKILASISC
Dinoponera_quadriceps_X2        ----RT--EDGLVV----GRS--RRKRDE-NDTLTNH-SEATDQ-------KR-DRTSKC
Harpegnathos_saltator_X1        ----RT--EAGLALGRRYGRP--RWKRDERDDSSTNHSSEATDE-------ER-DRTTKC
Linepithema_humile_X2           ----RT--EDSRMA----DWPS-RRRRDE-NNSLTSD-SKASGE-------KR-DRTSRC
Linepithema_humile_X1           ----RT--EDSRMA----DWPS-RRRRDE-NNSLTSD-SKASGE-------KR-DRTSRC
Ooceraea_biroi                  ----RT--EDVLAA----DRRPLRRKQHD-ENGSSKSDLSAAIE-------KR-DRTLRC
Camponotus_floridanus           ----RT---EEHFG----QRD--RRKRDE-SANSRAK----IVE-------KR-DRTLRC
Solenopsis_invicta              -ATEWT--GDSHAA----DRSS-RRKSDE-NDLSVND-SRVTVE-------KR-DRTLKC
Pogonomyrmex_barbatus_X1        ----WT--RDSHAA----DHQSPRRKRDE-NDLSACDSAEPIVG-------RR-DRTLRC
Vollenhovia_emeryi_X1           ----WT--GDSHVA----HRSS-RRKRDK-NDSLAND-SGAIVG-------KR-DRTLRR
Wasmannia_auropunctata          ----WTPGDGSHVA----DRSS-RRKRDK-NDSPANDSSEATVG-------KRGNRTLRC
Cyphomyrmex_costatus            ------------------------------------------------------------
Trachymyrmex_septentrionalis    ----RT--GDSHVA----DR-S-RRKREK-NDLLTND-SKTTV-----------------
Atta_colombica                  -----------------------RARRDL-ALSIVN------------------SRKLCC
Trachymyrmex_cornetzi           ----WT--GDGHVA----DR-S-RRKRDK-NDLLTND-SKTTVG-------KR-DRTLRR
Acromyrmex_echinatior           ----WT--GDSHAA----DR-S-RRKRDK-NDLLTND-SKTTVG-------KR-DRTLRC
Atta_cephalotes                 ----WT--GDNHVA----DR-S-RRKRDK-NDLLTND-SKTTVG-------KR-DRTLRC


Drosophila_melanogaster         ANVSYTKFLLLLQTLAAATTRLSLSPKNY-------------------------------
Halyomorpha_halys               ------------------MQAALGLY----------------------------------
Rhodnius_prolixus               ------------------HQEMIVYW----------------------------------
Cimex_lectularius_X1            WRTGPSPISTCERTDLTFCVMLLAFW----------------------------------
C._lectularius_X2               --------------------MLLAFW----------------------------------
Cryptotermes_secundus           ------------------------MWNML-------------------CA----------
Miniopterus_natalensis          ------------------------------------------------------------
Callithrix_jacchus              ------------------------------------------------------------
Heterocephalus_glaber_X1        ----------------------MGAW----------------------------------
Dasypus_novemcinctus            ------------------------------------------------------------
Tupaia_chinensis_X2             ------------------------------------------------------------
Homo_sapiens_X1                 ------------------------------------------------------------
Pongo_abelii_2                  ------------------------------------------------------------
Pan_troglodytes_X2              ------------------------------------------------------------
Boleophthalmus_pectinirostris   ------------------------MWLRT-------------------------------
Larimichthys_crocea_X2          ----------------MVSYPESVMWLRT-------------------------------
Larimichthys_crocea             ------------------------------------------------------------
Lates_calcarifer                --------------MVSYPESV--------------------------------------
Seriola_lalandi_dorsalis        --------------MVSYPESV--------------------------------------
Seriola_dumerili                --------------MVSYPESV--------------------------------------
Zootermopsis_nevadensis         ------------------------MW----------------------------------
Parasteatoda_tepidariorum       ------------------------------------------------------------
Onthophagus_nigriventris        -----------LADTGRLCSWLLVAW----------------------------------
Nicrophorus_vespilloides        ------------------------------------------------------------
Tribolium_castaneum             ------------------------------------------------------------
Nilaparvata_lugens              --------TRRGGGGLRGGYGDDVSW----------------------------------
Blattella_germanica             ------------------MDTDWWRW----------------------------------
Nasonia_vitripennis_X1          FR-----STTTSTRRRRSASSV-VRWVSA-------------------KC----------
Microplitis_demolitor           NRQCFM-KPMYRCGIVKSAASV-VRW----------------------------------
Neodiprion_lecontei             DVASSLSSTTTT-RRPSCCSSLSVLFRNL-------------------STINVSTIFFIV
Athalia_rosae                   DV--------PLQCRLRCASSV-VRWRSVPTSHNDRDGVYVDNYTGDKTSNEETNNNKHL
Orussus_abietinus               RR-----DSVDEGRWLRCASSV-VRWTQG-------------------KR----------
Dufourea_novaeangliae           HA-----TTSTSPFTSRSCGPSDVRW----------------------------------
Megachile_rotundata_X1          -------SSSSSSSSSSNSVSSVVRW----------------------------------
Melipona_quadrifasciata         ------------------------------------------------------------
Apis_mellifera_X1               -------RPDDGPSSSGCVSSV-VRWNST-------------------------------
Eufriesea_mexicana_1            SRITECDRLRPDASSSSYVSSV-VRW----------------------------------
Eufriesea_mexicana_2            ------------------------------------------------------------
Bombus_terrestris               SKISQRDRLVSDASSTSYVSSV-VRW----------------------------------
Bombus_impatiens                SKISQRDRLVSDASSTSYVSSV-VRW----------------------------------
Polistes_dominula_X1            TRENNYSSMQNRGENSRYASSV-VRW----------------------------------
```

```
Cephus_cinctus_X1                  RR-----NRVDQGRWLRSASSV-VRW----------------------------------
Dinoponera_quadriceps_X2           DR-----VPLCE-SSARCASSV-VRW-GS-------------------VA----------
Harpegnathos_saltator_X1           DR-----VPLCE-SSVRCASSV-VRW-SS-------------------VA----------
Linepithema_humile_X2              DR-----IPLDE-SSARCASNV-VRW-KL-------------------AS----------
Linepithema_humile_X1              DR-----IPLDE-SSARCASNV-VRW-KL-------------------AS----------
Ooceraea_biroi                     DR-----VPAVE-SSARCASSV-VRW-NL-------------------AV----------
Camponotus_floridanus              DR-----LSRYESSSARCASSV-VRWNHL-------------------AA----------
Solenopsis_invicta                 DR-----TPLRE-SSARCASSV-VRW-IF-------------------AA----------
Pogonomyrmex_barbatus_X1           DR-----TPIRE-SSARCASSV-VRW-IF-------------------AV----------
Vollenhovia_emeryi_X1              DR-----TPLRE-SSARCASSV-VRW-IF-------------------AV----------
Wasmannia_auropunctata             DR-----TLLRE-SSARCASSV-VRW-IF-------------------AG----------
Cyphomyrmex_costatus               ----------------NCCTLMLLRA----------------------------------
Trachymyrmex_septentrionalis       ------------------------------------------------------------
Atta_colombica                     -----------------VINSV-TIW-FS-------------------SV----------
Trachymyrmex_cornetzi              DR-----TPLRE-SSAKCASNV-VRW-MF-------------------SI----------
Acromyrmex_echinatior              DR-----TPLHE-SSAKCASSV-VRW-IF-------------------SI----------
Atta_cephalotes                    DR-----TPLHE-SSAKCASSV-VRW-IF-------------------SI----------


Drosophila_melanogaster            --------------------------KQQQQLQHNQQLPRATPQQKQQEKDRHKCFHYKH
Halyomorpha_halys                  ------------------------------------------------------------
Rhodnius_prolixus                  ------------------------------------------------------------
Cimex_lectularius_X1               ------------------------------------------------------------
C._lectularius_X2                  ------------------------------------------------------------
Cryptotermes_secundus              --------------------------AARKGSLFRLRPGRGVKA----------------
Miniopterus_natalensis             ------------------------------------------------------------
Callithrix_jacchus                 --------------------------MGAGSRRG--------------------------
Heterocephalus_glaber_X1           --------------------------GARG------------------------------
Dasypus_novemcinctus               --------------------------XMGAGGRRG-------------------------
Tupaia_chinensis_X2                --------------------------MGAGDRRG--------------------------
Homo_sapiens_X1                    --------------------------MATGGRRG--------------------------
Pongo_abelii_2                     --------------------------MGTGGRRG--------------------------
Pan_troglodytes_X2                 --------------------------MGTGGRRG--------------------------
Boleophthalmus_pectinirostris      ------------------------------------------------------------
Larimichthys_crocea_X2             ------------------------------------------------------------
Larimichthys_crocea                ------------------------------------------------------------
Lates_calcarifer                   ------------------------------------------------------------
Seriola_lalandi_dorsalis           ------------------------------------------------------------
Seriola_dumerili                   ------------------------------------------------------------
Zootermopsis_nevadensis            ---------------------------HTGGLDMCLV-----------------------
Parasteatoda_tepidariorum          ------------------------------------------------------------
Onthophagus_nigriventris           ------------------------------------------------------------
Nicrophorus_vespilloides           --------------------------MAATGLTTGGALLFA-------------------
Tribolium_castaneum                ------------------------------------------------------------
Nilaparvata_lugens                 --------------------------GVVSVLAA--------------------------
Blattella_germanica                ------------------------------------RWQS--------------------
Nasonia_vitripennis_X1             --------------------------SRTKIARTTGFWPG--------------------
Microplitis_demolitor              --------------------------SFEGGTF---SWFG--------------------
Neodiprion_lecontei                LFIANLNYASTTSAHGVY------DTSDNRYYNSNGRHTQH-------------------
Athalia_rosae                      QYYSKSSPPTTPQRSTSSSSSSCSSSSSSSSSSSSRRLPDNKQTSCLSKYFLNKSASIYS
Orussus_abietinus                  --------------------------RM--------PWQI--------------------
Dufourea_novaeangliae              ----------------------------HSWTERSFRWTRKGPS----------------
Megachile_rotundata_X1             ------------------------------------SWNSRRT-----------------
Melipona_quadrifasciata            ------------------------------------------------------------
Apis_mellifera_X1                  --------------------------RSARSLPATSHEQQT-------------------
Eufriesea_mexicana_1               ----------------------------ISRLERSIRWTRQKQ-----------------
Eufriesea_mexicana_2               ------------------------------------------------------------
Bombus_terrestris                  ----------------------------SLRPERSFRWTRQKHA----------------
Bombus_impatiens                   ----------------------------SLRPERSFRWTRQKHA----------------
Polistes_dominula_X1               -------------------------------KNLC-YWRL--------------------
Cephus_cinctus_X1                  ----------------------------WCTQNFLISWQR--------------------
Dinoponera_quadriceps_X2           --------------------------RFAGR-SSK-SWRR--------------------
Harpegnathos_saltator_X1           --------------------------RLVGR--SK-SWRR--------------------
Linepithema_humile_X2              --------------------------RLQA--KST-EWRR--------------------
Linepithema_humile_X1              --------------------------RLQA--KST-EWRR--------------------
Ooceraea_biroi                     --------------------------RFQD--KST-KWQRI-------------------
Camponotus_floridanus              --------------------------GFH---KST-EWRR--------------------
Solenopsis_invicta                 --------------------------RFYADFKSI-EWRR--------------------
Pogonomyrmex_barbatus_X1           --------------------------RFYSDLKSA-EWRR--------------------
Vollenhovia_emeryi_X1              --------------------------RFHRDFKLT-EWRK--------------------
Wasmannia_auropunctata             --------------------------RLYGDFKLT-EWRR--------------------
```

```
Cyphomyrmex_costatus               -----------------------------RRDLALVEHTR--------------------
Trachymyrmex_septentrionalis       ------------------------------------------------------------
Atta_colombica                     --------------------------T---------------------------------
Trachymyrmex_cornetzi              --------------------------RFYKDFKSI-EWRR--------------------
Acromyrmex_echinatior              --------------------------RFYRDFKST-EWRR--------------------
Atta_cephalotes                    --------------------------RFYRDFKST-EWRR--------------------


Drosophila_melanogaster            NYSYSPGISLLLFILLANTLAIQAVVLPAHQQHLLHNDIADGLDKTALSVSGTQTRWPRS
Halyomorpha_halys                  ------VLWLAYFGAAR-------------------------------------------
Rhodnius_prolixus                  ------VL--LYCTGITFL--------------ILGMQPAYTK-----------------
Cimex_lectularius_X1               ------RIWWLLTIALSST--------------VMGR-----------------------
C._lectularius_X2                  ------RIWWLLTIALSST--------------VMGR-----------------------
Cryptotermes_secundus              ------YMYVYLCIGLLVT--------------CSVTQVAKEGGK---------------
Miniopterus_natalensis             ------LTCHAFCIFSLV------------------------------------------
Callithrix_jacchus                 ------AAAAPLLVAVAAL--------------LLGAAGQMYPGE---------------
Heterocephalus_glaber_X1           ------AATAPLLVAAAAL--------------LVGVTGHLYPGE---------------
Dasypus_novemcinctus               ------AAAAPLLLAVAAL--------------LVGAAGHLYPGE---------------
Tupaia_chinensis_X2                ------AAAAPLLVAVAAL--------------LVGAAGHLYPGE---------------
Homo_sapiens_X1                    ------AAAAPLLVAVAAL--------------LLGAAGHLYPGE---------------
Pongo_abelii_2                     ------AAAAPLLVAVAAL--------------LLGAAGHLYPGE---------------
Pan_troglodytes_X2                 ------AAAAPLLVAVAAL--------------LLGAAGHLYPGE---------------
Boleophthalmus_pectinirostris      ------LCFVVVAVCILSC--------------------HRANGE---------------
Larimichthys_crocea_X2             ------LCFVLTACCLMMS--------------C---HRASGE-----------------
Larimichthys_crocea                ------MCLRGESLQTCVG--------------ETLPNL---------------------
Lates_calcarifer                   ------MCLRTLCFVLTAV--------------CFLMSCHRATGE---------------
Seriola_lalandi_dorsalis           ------MCLRTLCFILTAV--------------CIVMSCHRVTGE---------------
Seriola_dumerili                   ------MCLRTLCFILTAV--------------CIVMSCHRVTGE---------------
Zootermopsis_nevadensis            ------FILVLLLLGLPLS-------RVDGTVTISLSNPATGGNATTQMTSER-------
Parasteatoda_tepidariorum          ------LLHLVLLLVFLLS--------------TGEAQRPDRYEK---------------
Onthophagus_nigriventris           ------VVFGFYCNLPVTH--------------AGYITEYNGFTPYNKTSG---------
Nicrophorus_vespilloides           ------GALLAFISSTPTT--------------SEKMPITAGLSPKAHAYIHS-------
Tribolium_castaneum                ------MASNPVGLHKHSV--------------SQIPNVKLHSGRSHS------------
Nilaparvata_lugens                 ------VCCLLLCLANPVE-------AFASLGTELRPRVDLSTDRSSPDARG--------
Blattella_germanica                ------VIIIFFFVWMVPA--------------QLIPQNDIKVVQKSG------------
Nasonia_vitripennis_X1             ------ALSTGIVLLMLLL--------------TLGRAVAENAKTAVEGTAPN-------
Microplitis_demolitor              ------LIWIILILCPIVT-----------------ASFTERSTQTFHPTEGE-------
Neodiprion_lecontei                ------FDHAVRDISSSSS--------------SSSAVAAAASTSSSSTSSSA-------
Athalia_rosae                      SVRLLKFSWTFAALIILLT--------------ANVNVANAQVNRVNTEISTAVTKRD--
Orussus_abietinus                  ------LIWLTAMVLLTLA--------------A--PCVIADSQNSILANTSV-------
Dufourea_novaeangliae              ------LAWTILFLLVLVF--------------PALAGLQKGISKSSSKEDG--------
Megachile_rotundata_X1             ------LLWTFLFLLVLVF--------------PALADPKKETSRSLSKEDG--------
Melipona_quadrifasciata            ------MSSLACGGFDPPG--------------PDRPAMMEKKGEGRKEQLGG-------
Apis_mellifera_X1                  ------LTWTILFLFLVLV-------------FSASADLQKPTSRERSSKEEG-------
Eufriesea_mexicana_1               ------FAWMILFLLVLVF--------------PASADLQKGTSSGSSKEEG--------
Eufriesea_mexicana_2               ------MV----------------------------------------------------
Bombus_terrestris                  ------LAWTILFLLVLVP--------------STSANLQKGISRGSSKEEG--------
Bombus_impatiens                   ------LAWTILFLLVLVP--------------STSANLQKGMSRGSSKEEG--------
Polistes_dominula_X1               ------AFSIALFL---AL--------------TFPPNAVASLQRK-HSVGYS-------
Cephus_cinctus_X1                  ------LIAIILLIIASFV--------LPASAEFLSPPITEVTASSSKTE----------
Dinoponera_quadriceps_X2           ------LFSIILLIL--AL-----------------SGLVAAQRR--TAIGPS-------
Harpegnathos_saltator_X1           ------LFSVIFLLL--AL-----------------PGLVTAQRH--TAMGPS-------
Linepithema_humile_X2              ------LMWIVFLLAMVSM--------------VIHPGLAAAQKN-ISTVKSF-------
Linepithema_humile_X1              ------LMWIVFLLAMVSM--------------VIHPGLAAAQKN-ISTVKSF-------
Ooceraea_biroi                     ------LLWIAVLIL-ASV-----------------PNLAMAQQSKFGAIGPL-------
Camponotus_floridanus              ------LLWIVFLVFLASS-----------------PDLAAAQQN--STDG---------
Solenopsis_invicta                 ------FLWIVLFIF--AI-----------------CNLAAANNI--AIGPSR-------
Pogonomyrmex_barbatus_X1           ------LFWIVLLML--AV-----------------PDLAAAKEN--KTADDK-------
Vollenhovia_emeryi_X1              ------LLWIVLLIL--AV-----------------PDLAAAHDA--ANSGPS-------
Wasmannia_auropunctata             ------LLWIVFLIL--AV-----------------PDFAAAHDT--A-IGRS-------
Cyphomyrmex_costatus               ------NHCIVNVVGVSVA--------------LLIPSRYVSFVA---------------
Trachymyrmex_septentrionalis       ------------------------------------------------------------
Atta_colombica                     ------------------------------------------------------------
Trachymyrmex_cornetzi              ------LLWIVLLIL--TV-----------------PDLAAAHDT--G-LDNG-------
Acromyrmex_echinatior              ------LLWIVLLIL--TI-----------------PDLAAAHDT--D-LDNG-------
Atta_cephalotes                    ------LLWIVLLIL--TV-----------------PDLAAAHDT--G-LDNG-------


Drosophila_melanogaster            ESNPTMRLSQNVK-PCKSMDIRNMVSHF------NQLENCTVIEGFLLIDLINDASP---
Halyomorpha_halys                  --------------VCPSMDIRNSASAL------LNLEPCSRIQGYLQIVLMENETESNF
Rhodnius_prolixus                  --------------ICPSMDIRNTVSAL------NKLAGCRVIDGYFSFVLIDYADESEY
```

```
Cimex_lectularius_X1                --------------TCPSMDIRNNVTNF------NLLRGCSVIDGFLHIVLIDKSSQQDF
C._lectularius_X2                   --------------TCPSMDIRNNVTNF------NLLRGCSVIDGFLHIVLIDKSSQQDF
Cryptotermes_secundus               --------------ICRSMDIRNRVQAL------QRLSGCRVVEGFVRVVLIDRANETDY
Miniopterus_natalensis              --------------VCPSMDIRNNLTRL------HELANCSVIEGHLQILLMFKTRPEDF
Callithrix_jacchus                  --------------VCPGMDIRNNLSRL------HELENCSVIEGHLQILLMFKTRPEDF
Heterocephalus_glaber_X1            --------------VCPGMDIRNNLTRL------HRLENCSIIEGHLQILLMFKTKPEDF
Dasypus_novemcinctus                --------------VCPGMDIRNNLTRL------HELENCSVIEGHLQILLMFKTRPEDF
Tupaia_chinensis_X2                 --------------VCPGMDIRNNLTRL------RELENCSVIEGHLQILLMFKTRPEDF
Homo_sapiens_X1                     --------------VCPGMDIRNNLTRL------HELENCSVIEGHLQILLMFKTRPEDF
Pongo_abelii_2                      --------------VCPGMDIRNNLTRL------HELENCSVIEGHLQILLMFKTRPEDF
Pan_troglodytes_X2                  --------------VCPGMDIRNNLTRL------HELENCSVIEGHLQILLMFKTRPEDF
Boleophthalmus_pectinirostris       --------------ICSSKDIRNNVTNL------QVLENCTIIEGHLKILLMFRTKAEDF
Larimichthys_crocea_X2              --------------VCPSKDIRNNVTNL------QTLENCTVIEGHLKILLMFRTKPEDF
Larimichthys_crocea                 --------------VCPSKDIRNNVTNL------QTLENCTVIEGHLKILLMFRTKPEDF
Lates_calcarifer                    --------------VCPSKDIRNNVTNL------QTLENCTVIEGHLKILLMFRTKPEDF
Seriola_lalandi_dorsalis            --------------ICPNKDIRNNVTNL------QSLENCTVIEGHLKILLMFRTKPEDF
Seriola_dumerili                    --------------ICPNKDIRNNVTNL------QSLENCTVIEGHLKILLMFRTKPEDF
Zootermopsis_nevadensis             --------------ICVSVDIRNSVREF------EQLRGCTVVEGFVRIVLIDRANESDF
Parasteatoda_tepidariorum           --------------VCRSIDIRNKVENF------KKLENCTVIEGSLQILLIDHGKAQDY
Onthophagus_nigriventris            --------------ICKSVDIRNSGEMF------ENLRGCRVVEGYIQILLFDHENKSTF
Nicrophorus_vespilloides            -NTSG---------ICKSIDVRNSMQAL------LNLRGCRVVEGFVQISLFDAVEDTEF
Tribolium_castaneum                 --------------FCESVDIRNTLDSF------NRLKGCHVVEGFVQILLFDNVNETEL
Nilaparvata_lugens                  --------------VCQSLDIRNAAREF------HQLQGCQVVEGFVQIVLMDKAVESEF
Blattella_germanica                 --------------VCPSIDIRNRVEAF------KRLNGCKVVEGFVQIVLIDQASESSF
Nasonia_vitripennis_X1              -HASGR--------VCQSIDIRNKLSEF------SKLDGCRVVEGFLQILLLENTNEAHF
Microplitis_demolitor               --------------VCQSIDIRNSVTQF------SRLKGCRVVEGFVQILLIDHADDSAY
Neodiprion_lecontei                 -KRT----------VCQSVDVRNSVNQF------SRLEGCRVVEGFVQILLIDHANDTSY
Athalia_rosae                       --------------VCQSVDVRNSVHQF------SRLEGCRVVEGFVQILLIDHADDSHY
Orussus_abietinus                   -ANTFNKT------VCQSVDIRNNVNQF------SRLDGCRVVEGFVQILLIDNADDQSY
Dufourea_novaeangliae               --------------VCQSIDVRNSVSSF------SKLNGCRIVEGFVQILLIDSADPSEF
Megachile_rotundata_X1              --------------VCQSIDIRNYVHHF------SRLKGCRLVEGFVQILLIDNAEPSEF
Melipona_quadrifasciata             --------------LHAVKHARRYVIGFRAAWTAATLSGCRLVEGFVQILLIDNAEPAEF
Apis_mellifera_X1                   --------------VCQSIDIRNTVRQF------SRLKGCRLVEGFVQILLIDNAEPSEY
Eufriesea_mexicana_1                --------------VCQSIDIRNSVRHF------SRLKGCRLVQGFVQILLIDNAEPSEY
Eufriesea_mexicana_2                --------------VCQSIDIRNSVRHF------SRLKGCRLVQGFVQILLIDNAEPSEY
Bombus_terrestris                   --------------VCQSIDIRNSVRYF------SRLKGCRLVEGFVQILLIDNAEPSEY
Bombus_impatiens                    --------------VCQSIDIRNSVRYF------SRLKGCRLVEGFVQILLIDNAEPSEY
Polistes_dominula_X1                -KKDG---------VCQSIDIRNSVNQF------SRLQGCRVVEGFVQILLIDHADDSAF
Cephus_cinctus_X1                   --------------VCQSVDIRNSVSEF------SRLEGCRVVEGFVQILLIDRADDLAY
Dinoponera_quadriceps_X2            -KKGG---------VCQSIDIRNSVDQF------SRLEGCQVVEGFVQILLIDKANQASY
Harpegnathos_saltator_X1            -IKDG---------VCQSIDIRNSVDQF------SRLEGCRVVEGFVQILLIDRANHTAY
Linepithema_humile_X2               -NTDG---------VCQSIDIRNSVNEL------SKLEGCRVVEGFVQILLIDRAEPSNY
Linepithema_humile_X1               -NTDG---------VCQSIDIRNSVNEL------SKLEGCRVVEGFVQILLIDRAEPSNY
Ooceraea_biroi                      -RKNG---------VCQSIDIRNNVNQF------SKLEGCRVVEGFVQILLIDRAEQSAY
Camponotus_floridanus               --------------VCQSIDIRNSVDQF------LRLNGCRVVEGFVQILLIDRAEQSAY
Solenopsis_invicta                  -NK-----------VCQSIDIRNNVNEF------SKLKGCQVVEGFVQISLIDRAEPLDY
Pogonomyrmex_barbatus_X1            -GI-----------VCESIDIRNSVDQFH-----PKLNNCRVVEGFVQILLIDRAEPSDF
Vollenhovia_emeryi_X1               -NRDG---------VCQSIDIRNSVSQF------SKLEGCRVVEGFIQILLIDRAEQSAY
Wasmannia_auropunctata              -NKDG---------VCQSIDIRNSVSQF------SKLEGCRVVEGFVQILLIDRAEQSAY
Cyphomyrmex_costatus                --------------VCQSIDIRNSVSDF------SKLEGCRVVEGFVQILLIDRAEQSAY
Trachymyrmex_septentrionalis        --------------VCQSIDIRNSVSEF------SKLEGCRVVEGFVQILLIDRAEQSAY
Atta_colombica                      --------------VCQSIDIRNSVSEF------SKLEGCRVVEGFVQILLIDRAEQSAY
Trachymyrmex_cornetzi               -GRNLCIVNVSSVAVCQSIDIRNSVSEF------SKLEGCRVVEGFVQILLIDRAEQSAY
Acromyrmex_echinatior               --------------VCQSIDIRNSVSEF------SKLEGCRVVEGFVQILLIDRAEQSAY
Atta_cephalotes                     --------------VCQSIDIRNSVSEF------SKLEGCRVVEGFVQILLIDRAEQSAY
                                                           *.     :        *  *  ::* . . *:

Drosophila_melanogaster             LNRSFPKLTEVTDYIIIY----------------------RVTGLHSLSKIFPNLSVIRG
Halyomorpha_halys                   ANLSFPKLSEVTSYVIFY----------------------RVHGLTSIGKLFPNLTIIGG
Rhodnius_prolixus                   DNMTFPELREITSFLMVN----------------------KVSGLRSLGRLFPNLSIIRG
Cimex_lectularius_X1                ENVTFPDLREVTEYVLFY----------------------QVNGLTSLGEVFPNLAVIRG
C._lectularius_X2                   ENVTFPDLREVTEYVLFY----------------------QVNGLTSLGEVFPNLAVIRG
Cryptotermes_secundus               YRFSFPELREITDYLLVY----------------------RVAGLTSLGKLFPNLTVIRG
Miniopterus_natalensis              RDLSFPNLIMITDYLLLF----------------------RVYGLESLKDLFPNLTVIRG
Callithrix_jacchus                  RDLSFPKLIMITDYLLLF----------------------RVYGLESLKDLFPNLTVIRG
Heterocephalus_glaber_X1            RDLSFPKLVMITDYLLLF----------------------RVYGLESLKDLFPNLTVIRG
Dasypus_novemcinctus                RDLSFPKLIMITDYLLLF----------------------RVYGLESLKDLFPNLTVIRG
Tupaia_chinensis_X2                 RDLSFPKLIMITDYLLLF----------------------RVYGLESLKDLFPNLTVIRG
Homo_sapiens_X1                     RDLSFPKLIMITDYLLLF----------------------RVYGLESLKDLFPNLTVIRG
Pongo_abelii_2                      RDLSFPKLIMITDYLLLF----------------------RVYGLESLKDLFPNLTVIRG
Pan_troglodytes_X2                  RDLSFPKLIMITDYLLLF----------------------RVYGLESLKDLFPNLTVIRG
```

```
Boleophthalmus_pectinirostris      RGVSFPKLTVITDYLLMF----------------------RVYGLESLSDLFPNLTLIRG
Larimichthys_crocea_X2             RGLSFPKLIVVTDYLLLF----------------------RVYGLESLSDLFPNLTVIRG
Larimichthys_crocea                RGLSFPKLIVVTDYLLLF----------------------RVYGLESLSDLFPNLTVIRG
Lates_calcarifer                   RGLSFPKLTVVTDYLLLF----------------------RVYGLESLSDLFPNLTVIRG
Seriola_lalandi_dorsalis           RGLSFPKLRVVTDYLLLF----------------------RVYGLESLSDLFPNLTVIRG
Seriola_dumerili                   RGLSFPKLRVVTDYLLLF----------------------RVYGLESLSDLFPNLTVIRG
Zootermopsis_nevadensis            ENVTFPELREITCYLMLY----------------------RVRGLKNLGFLFPNLAVIRG
Parasteatoda_tepidariorum          DYLSFPNLVEITDYLLVY----------------------RAFGLNSLGKLFPNLAVIRG
Onthophagus_nigriventris           ENISFPELREITGHLLLY----------------------RVNGLTSLGKLFPNLAVIRG
Nicrophorus_vespilloides           EGISFPELTEITDFLLLY----------------------RVHGLRSVGQLFPNLAVIRG
Tribolium_castaneum                SLLSFPNLTEITDYLLLY----------------------RVNGLRSIGQLFPNLSVIRG
Nilaparvata_lugens                 ANFSFPELREITGYLLLY----------------------RIDGLRTLSNLFPNLAVIRG
Blattella_germanica                ANLPFPELREITDYLLLY----------------------RVNGLRSIGSLFPNLTVIRG
Nasonia_vitripennis_X1             ENMSYPDLVEITGYFMVY----------------------RVQGLQSLGHLFPNLAVIRG
Microplitis_demolitor              TNLTFPDLVEITGYLLLY----------------------RVNGLRSVGQLFPNLTVIRG
Neodiprion_lecontei                ANLTFPDLVEITGYLLLY----------------------RVNGLRSVGHLFPNLTVIRG
Athalia_rosae                      ANLTFPDLVEITGYLLLY----------------------RVNGLRSVGHLFPNLTVIRG
Orussus_abietinus                  ANLTFPNLVEITGYLILY----------------------RVYGLQSIGHLFPNLAVIRG
Dufourea_novaeangliae              ANITFPELREITGYLLFY----------------------RVKGLRSVGRLFPNLTVIRG
Megachile_rotundata_X1             VNISFPELKEITGYLLLY----------------------RVKGLRSIGRLFPNLTVIRG
Melipona_quadrifasciata            ANISFPELKEITGYLLFY----------------------RVKGLRSIGRLFPNLSVIRG
Apis_mellifera_X1                  TNISFPELREITGYLLLYSRLLPLLPLSFTALPSLSVSVLRVKGLRSIGRLFPNLTVIRG
Eufriesea_mexicana_1               ANVSFPELKEITGYLLLY----------------------RVKGLRSIGRLFPNLTVIRG
Eufriesea_mexicana_2               ANVSFPELKEITGYLLLY----------------------RVKGLRSIGRLFPNLTVIRG
Bombus_terrestris                  ANISFPELKEITGYLLLY----------------------RVKGLRSIGRLFPNLTVIRG
Bombus_impatiens                   ANISFPELKEITGYLLLY----------------------RVKGLRSIGRLFPNLTVIRG
Polistes_dominula_X1               ANLTFPDLVEITEYLILY----------------------RVNGLRSVGRLFPNLTLIRG
Cephus_cinctus_X1                  ANLTFPDLVEITGYLVLY----------------------RVNGLKSVGKLFPNLTVIRG
Dinoponera_quadriceps_X2           ANISFPELVEITGYLILF----------------------RVNGLRSIGHLFPNLTIIRG
Harpegnathos_saltator_X1           ANISFPQLVEITGYLILY----------------------RVSGLRSIGHLFPNLTIIRG
Linepithema_humile_X2              TDHVYPELVEITGYLILY----------------------RVNGLRSIGHIFPNLTVIRG
Linepithema_humile_X1              TDHVYPELVEITGYLILY----------------------RVNGLRSIGHIFPNLTVIRG
Ooceraea_biroi                     ANLSFPDLVEITGYLVLY----------------------RVSGLRSVGRLFPNLAVIRG
Camponotus_floridanus              ANLSFPDLVEITGYLILY----------------------RVSGLRSIGRLFPNLTVIRG
Solenopsis_invicta                 ANFSFPELVEITDFLLLY----------------------RVNGLKTIGQLFPNLAVIRG
Pogonomyrmex_barbatus_X1           ANISFPKLVEITGYLILY----------------------RVSGLKSVGHLFPNLAVIRG
Vollenhovia_emeryi_X1              ANLSFPDLVEITGYLILY----------------------RVNGLRAVGHLFPNLTVIRG
Wasmannia_auropunctata             DNISFPELVEITGYLILF----------------------RVSGLRTVGHLFPNLAVIRG
Cyphomyrmex_costatus               ANLSFPDLVEITGYLILY----------------------RVSGLRTVGHLFPNLTVIRG
Trachymyrmex_septentrionalis       ANLSFPDLVEITGYLILY----------------------RVSGLRTIGRLFPNLTVIRG
Atta_colombica                     ANLSFPDLVEITGYLILY----------------------RVSGLRTVGRLFPNLTVIRG
Trachymyrmex_cornetzi              ANLSFPDLVEITGYLILY----------------------RVSGLRTVGRLFPNLMIIRG
Acromyrmex_echinatior              ANLSFPELVEITGYLILY----------------------RVSGLRTVGHLFPNLTVIRG
Atta_cephalotes                    ANLSFPDLVEITGYLILY----------------------RVSGLRTVGRLFPNLTVIRG
                                       :*.*  :* ..:.                       .  **  :  :**** :* *

Drosophila_melanogaster            NK-LFDGYALVVYSNFDLMDLGLHKLRSITRGGVRIEKNHKLCYDRTIDWLEIL--AENE
Halyomorpha_halys                  DL-LSVDYALIITQMPHLKEIDLPELRLILRGSVAIFKNPKLCYVDTVDWDLIA----TT
Rhodnius_prolixus                  ER-LFLDYALVITNMQNLLEIALTSVT-ILRGSVAIAWNKKLCYAETIDWDQIA--PGG-
Cimex_lectularius_X1               EK-LFLDYSFIVFQMPDLKTLKFKSFI-ILRGSVVIANNKNLCYDNTIDWDKVA--VSGV
C._lectularius_X2                  EK-LFLDYSFIVFQMPDLKTLKFKSFI-ILRGSVVIANNKNLCYDNTIDWDKVA--VSGV
Cryptotermes_secundus              NS-LFVDYALVVYEMFQLQEIGLRSLTAVLRGSVRFDKNPELCYVDTVDWRSIL--RN--
Miniopterus_natalensis             SR-LFFNYALVIFEMVHLKELGLYSLMNITRGSVRIEKNNELCYLATIDWSRIL--DSVE
Callithrix_jacchus                 SR-LFFNYALVVFEMVHLKELGLYSLMNITRGSVRIEKNNELCYLATIDWSRIL--DSVE
Heterocephalus_glaber_X1           SR-LFFNYALVIFEMVHLKELGLYSLMNITRGSVRIEKNNELCYLATIDWSRIL--DSVE
Dasypus_novemcinctus               SR-LFFNYALVIFEMVHLKELGLYSLMNITRGSVRIEKNNELCYLATIDWSRIL--DSVE
Tupaia_chinensis_X2                SR-LFFNYALVIFEMVHLKELGLYNLMSITRGSVRIEKNNELCYLATIDWSRIL--DSVE
Homo_sapiens_X1                    SR-LFFNYALVIFEMVHLKELGLYNLMNITRGSVRIEKNNELCYLATIDWSRIL--DSVE
Pongo_abelii_2                     SR-LFFNYALVIFEMVHLKELGLYNLMNITRGSVRIEKNNELCYLATIDWSRIL--DSVE
Pan_troglodytes_X2                 SR-LFFNYALVIFEMVHLKELGLYNLMNITRGSVRIEKNNELCYLATIDWSRIL--DSVE
Boleophthalmus_pectinirostris      SN-LFFNYALVIFEMIQLREIGLHSLMNITRGAVRIEKNPDLCYLSTLDWSKIL--DSVA
Larimichthys_crocea_X2             NN-LFFNYALVMFEMLQLREIGLHSLMNITRGAVRIEKNPDLCYLSTLDWSKIL--DSVE
Larimichthys_crocea                NN-LFFNYALVMFEMLQLREIGLHSLMNITRGAVRIEKNPDLCYLSTLDWSKIL--DSVE
Lates_calcarifer                   NN-LFFNYALVVFEMLQLREIGLHSLMNITRGAVRIEKNPDLCYLSTLDWSKIL--DSVE
Seriola_lalandi_dorsalis           NN-LFFNYALVVFEMLQLREIGLHSLMNITRGAVRIEKNPDLCYLSTLDWSKIL--DSVE
Seriola_dumerili                   NN-LFFNYALVVFEMLQLREIGLHSLMNITRGAVRIEKNPDLCYLSTLDWSKIL--DSVE
Zootermopsis_nevadensis            NT-LFQDYALVVYEMVELQELGLWGLAAILRGSVRIEKNYALCYYNTVNWDAVT--NG--
Parasteatoda_tepidariorum          NE-LFHNYALVVFELYQLQEIGLSNLTDILRGSVRIEKNPGLCYVETIEWDLIA--KEGK
Onthophagus_nigriventris           QQPLLLGYALIIFEMPSLQEIGLYSLTDIMNGGIRIDKNPQLCFANTINWNRIMHKKNKD
Nicrophorus_vespilloides           QD-TVLGYSFIIYELSGLQEVGLYSLTDIQAGLIRIDKNPELCFVNSIDWDKIA--HE-K
Tribolium_castaneum                RS-TFYTYSFVVFEMSSLQEIGLYSLTDITRGLIRIDKNPSLCFVNSIDWEAIA--HE-K
```

```
Nilaparvata_lugens                  NT-LFFNYALVVFEMLHLQEMGLKSLTDIVRGAVHISKNPMLCYVDTIDWDKIA--RSSQ
Blattella_germanica                 ET-LFLNYALVAFEMIHLQEIGLYSLTDILRGSVRIEKNPLLCFVDTVEWDLIA--KKGR
Nasonia_vitripennis_X1              HS-LFMNYAFVAFEMTSLQEIGLASLTDIMRGSVRFEKNPVLCYANTVDWDLIA--HAGK
Microplitis_demolitor               HS-LFINYALVAFEMMNLQEVGLYSLTDILRGSVRFEKNPMLCYVDTVDWDIIA--KAGN
Neodiprion_lecontei                 HS-LFINYALVAFEMMHLQEIGLHSLTNIGRGSVRFEKNPALCYVDTIDWDLII--GVGK
Athalia_rosae                       HS-LFINYALVAFEMVHLQEIGLHSLTNIVRGTVRFEKNPALCYVDTIDWDLIT--GVGK
Orussus_abietinus                   HS-LFFNYALVVFEMMHLQEIGLYSLTDILRGSVRFEKNPVLCNADTIDWDIIA--KAGK
Dufourea_novaeangliae               HS-LFINYALVAFEMMSLQEIGLHSLTTIVRGSVRFEKNHALCYVDTIDWDLIG--KAGK
Megachile_rotundata_X1              HS-LFINYALVAFEMMNLQEIGLHSLTTIVRGSVRFEKNPALCYVDTIDWDLIA--KAGK
Melipona_quadrifasciata             HS-LFINYALVVFEMMSLQEVGLHSLTTVVRGSVRFEKNPALCYVDTIDWDLIA--KSGK
Apis_mellifera_X1                   HS-LFINYALVAFEMMNLQEIGLHSLTTIVRGSVRFEKNPALCYVDTIDWDLIA--KAGK
Eufriesea_mexicana_1                HS-LFINYALVAFEMMNLQEIGLHHLTTIVRGSVRFEKNPTLCYVDTIDWDLIA--KAGK
Eufriesea_mexicana_2                HS-LFINYALVAFEMMNLQEIGLHHLTTIVRGSVRFEKNPTLCYVDTIDWDLIA--KAGK
Bombus_terrestris                   HS-LFINYALVAFEMMNLQEIGLHSLTTIVRGSVRFEKNPALCYVDTIDWDLIA--KAGK
Bombus_impatiens                    HS-LFINYALVAFEMMNLQEIGLHSLTTIVRGSVRFEKNPALCYVDTIDWDLIA--KAGK
Polistes_dominula_X1                HS-LFINYALVAFEMMHLQEIGLHSLTDILRGSVRFEKNPALCYVDTIDWDIIA--KAGK
Cephus_cinctus_X1                   HS-LFINYALVMFEMMHLQEIGLHSLTDILRGSVRFEKNPALCYVDTIDWDNIA--KAGK
Dinoponera_quadriceps_X2            HS-LFVNYALVAFEMMQLQEIGLHSLTTILRGSVRFEKNPSLCYVDTIDWDIIA--KAGK
Harpegnathos_saltator_X1            RS-LFTNYALVAFEMMQLQEIGLHSLTTIQRGSVRFEKNPSLCYVDTIDWDIIA--RAGK
Linepithema_humile_X2               HS-LFINYALVAFEMMHLQEIGLHSLTNILRGSVRFEKNPTLCYVNSIDWDIIA--KAGK
Linepithema_humile_X1               HS-LFINYALVAFEMMHLQEIGLHSLTNILRGSVRFEKNPTLCYVNSIDWDIIA--KAGK
Ooceraea_biroi                      HS-LFINYALVAFEMMHLQELGLHSLTNILRGSVRFEKNPTLCYVDTIDWDIIA--KAGK
Camponotus_floridanus               HS-LFINYALVAFEMMHLQEIGLHSLTNILRGSVRFEKNPTLCYVDTIDWDIIA--KAGK
Solenopsis_invicta                  NS-LIMNYALMAFEMMHLQEIGLHSLTNILRGSVHFEKNPMLCFVDTIDWDIIA--KAGN
Pogonomyrmex_barbatus_X1            NS-LFINYALVAFEMMNLQEIGLHSLTNILRGFVRFEKNPTLCYVDTIDWDIIA--KAGK
Vollenhovia_emeryi_X1               HS-LFINYALVAFEMMHLQEIGLHSLTNILRGSVRFEKNPTLCYVDTIDWDMIA--KAGK
Wasmannia_auropunctata              HS-LFINYALVAFEMMHLQEIGLFSLTNILRGSVRFEKNPTLCYVDTIDWDIIA--PAGK
Cyphomyrmex_costatus                HS-LFINYALVAFEMMHLQEIGLHSLTSILRGSVRFEKNPTLCYIDTIDWDVIA--KAGK
Trachymyrmex_septentrionalis        HS-LFINYALVAFEMMHLQEIGLHSLTSILRGSVRFEKNPTLCYVDTIDWDIIA--KAGK
Atta_colombica                      HS-LFINYALVAFEMMHLQEIGLHSLTSILRGSVRFEKNPTLCYVDTIDWDIIA--KAGK
Trachymyrmex_cornetzi               HS-LFINYALVAFEMMHLQEIGLHSLTSILRGSVRFEKNPTLCYVDTIDWDIIA--KAGK
Acromyrmex_echinatior               HS-LFINYALVAFEMMHLQEIGLHSLTSILRGSVRFEKNPTLCYVDTIDWDIIA--KAGK
Atta_cephalotes                     HS-LFINYALVAFEMMHLQEIGLHSLTSILRGSVRFEKNPTLCYVDTIDWDIIA--KAGK
                                           *:::  .   *  : :  .  :  * : : .*  **   :::*  :

Drosophila_melanogaster             TQLVVLTENGKEKECR-LS----------------KCPGEIRIEEGHDNTAIEGELNASC
Halyomorpha_halys                   GSNFIVPAKDR---S--MCPGCG------------YCPP---------------------
Rhodnius_prolixus                   -DHFLVGNSPITD-P--------------------ECPG---------------------
Cimex_lectularius_X1                REYLFPNDTEKN--C--------------------SCPS---------------------
C._lectularius_X2                   REYLFPNDTEKN--C--------------------SCPS---------------------
Cryptotermes_secundus               GEHFAASNKEVPL-CP-------------------SCPA---------------------
Miniopterus_natalensis              DNYIVLNKDDNEE-CGDICPGTAKGKT--------NCPA------------------TVI
Callithrix_jacchus                  DNYIVLNKDDNEE-CGDICPGTSKGKT--------NCPA------------------TVI
Heterocephalus_glaber_X1            DNYIVLNKDDNEE-CGDICPGTTKGKT--------NCPA------------------TVI
Dasypus_novemcinctus                DNYIVLNKDDNEE-CGDICPGTAKGKT--------NCPA------------------TVI
Tupaia_chinensis_X2                 DNYIVLNKDDNEE-CGDICPGTAKGKT--------SCPA------------------TVI
Homo_sapiens_X1                     DNYIVLNKDDNEE-CGDICPGTAKGKT--------NCPA------------------TVI
Pongo_abelii_2                      DNYIVLNKDDNEE-CGDICPGTAKGKT--------NCPA------------------TVI
Pan_troglodytes_X2                  DNYIVLNKDDNEE-CGDICPGTAKGKT--------NCPA------------------TVI
Boleophthalmus_pectinirostris       DNYIVFNKNERE--CGDVCPGAAVGKT--------TCQT------------------TTI
Larimichthys_crocea_X2              DNYIVANKNDRE--CGDVCPGAAVGKT--------TCQT------------------TTI
Larimichthys_crocea                 DNYIVANKNDRE--CGDVCPGAAVGKT--------TCQT------------------TTI
Lates_calcarifer                    DNYIMANKNDRE--CGDVCPGAAVGKT--------TCLT------------------TTI
Seriola_lalandi_dorsalis            DNYIMANKNDRE--CGDVCPGAAIGKT--------TCQT------------------TTI
Seriola_dumerili                    DNYIMANKNDRE--CGDVCPGAAIGKT--------TCQT------------------TTI
Zootermopsis_nevadensis             GEHVIALNKDSAS-CP--GQG--------------MCDV---------------------
Parasteatoda_tepidariorum           GGHFIKGNKKSAD-CPNTCPGPPT-----------VCPK---------------------
Onthophagus_nigriventris            AELFIRSSKKENE-CP-------------------MCP-------------------RMD
Nicrophorus_vespilloides            GEHYIKNIKAENE-CP-LCPGEDEESSS-------LCPA------------------SKN
Tribolium_castaneum                 GEHFIKNLKSPNE-CP-ICPGDEKEDESGNSVAHIHCHK-------------APHRSDSE
Nilaparvata_lugens                  GGHFITGNKKKNE-CP-ICQTNA------------VCPT---------------------
Blattella_germanica                 GEHFITGNRSPNE-CP-MCANDLM-----------HC--------------------R--
Nasonia_vitripennis_X1              GGNVISSNRPKNE-CP-VCEK--------------DCPR------------------R--
Microplitis_demolitor               GEHVIADNKLKNL-CP-VCEK--------------HCPL------------------R--
Neodiprion_lecontei                 GEHVISGNKPRNG-CP-VCEK--------------HCPT------------------R--
Athalia_rosae                       GEHVISGNKPKNG-CP-VCEK--------------NCPT------------------R--
Orussus_abietinus                   GEHVISSNKPKNV-CP-VCEK--------------HCPT------------------R--
Dufourea_novaeangliae               GEHVIAGNNPKNG-CP-VCEK--------------HCPQ------------------R--
Megachile_rotundata_X1              GENVISGNNPKNG-CP-VCEK--------------HCPQ------------------S--
Melipona_quadrifasciata             GEHIIVGNNPRNG-CP-VCEK--------------HCPQ------------------R--
Apis_mellifera_X1                   GEHVISGNNPKNA-CP-VCEK--------------HCPQ------------------R--
```

```
Eufriesea_mexicana_1               GEHVIAANNRKNG-CP-VCEK--------------RCPQ------------------R--
Eufriesea_mexicana_2               GEHVIAANNRKNG-CP-VCEK--------------RCPQ------------------R--
Bombus_terrestris                  GEHVIAGNNPRNG-CP-VCEK--------------HCPQ------------------R--
Bombus_impatiens                   GEHVIAGNNPRNG-CP-VCEK--------------HCPQ------------------R--
Polistes_dominula_X1               GEHVISGNKPKND-CP-ICEK--------------NCTT------------------RLI
Cephus_cinctus_X1                  GEHVISGNKPKNG-CP-VCEK--------------NCPP------------------R--
Dinoponera_quadriceps_X2           GEYVISGNKLTNG-CP-MCEK--------------SCPE------------------K--
Harpegnathos_saltator_X1           GENVILGNKLTNG-CP-VCEK--------------SCPE------------------K--
Linepithema_humile_X2              GEHVISGNKPTNG-CP-ICDR--------------NCPK------------------K--
Linepithema_humile_X1              GEHVISGNKPTNG-CP-ICDR--------------NCPK------------------K--
Ooceraea_biroi                     GEHVISGNKLTNG-CP-VCDK--------------KCPA------------------R--
Camponotus_floridanus              GEHVISGNKITNG-CP-VCDK--------------SCPS------------------R--
Solenopsis_invicta                 GEHFIKDNKPSNG-CP-MCPR--------------KCPT------------------R--
Pogonomyrmex_barbatus_X1           GGHVIMDNKPTNG-CP-VCDK--------------KCPT------------------R--
Vollenhovia_emeryi_X1              GGHFIKDNKPMNG-CP-MCDK--------------RCPT------------------R--
Wasmannia_auropunctata             GGHVIMDNKPTNG-CP-VCDKFL------------RCPI------------------R--
Cyphomyrmex_costatus               GGHFIKDNKPTNG-CP-VCDK--------------KCPT------------------R--
Trachymyrmex_septentrionalis       GGHFIKDNKPTNG-CP-VCDK--------------KCPT------------------R--
Atta_colombica                     GGHFIKDNKPTNG-CP-VCDK--------------KCPT------------------R--
Trachymyrmex_cornetzi              GGHFIKDNKPTNG-CP-VCDK--------------KCPT------------------R--
Acromyrmex_echinatior              GGHFIKDNKPTNG-CP-VCDK--------------KCPT------------------R--
Atta_cephalotes                    GGHFIKDNKPTNG-CP-VCDK--------------KCPT------------------R--
                                                                       *

Drosophila_melanogaster            QLHNNRRLCWNSKLCQTKC----PEKCR--NNCIDEHT-------CCSQDCLGGCVIDKN
Halyomorpha_halys                  -----RGNCWSRSHCQVL-----EW---------------GT---DCHSECLGGC--SGP
Rhodnius_prolixus                  -CACKNNLCWSRNQCQ-VCVYFISIINLIPPSLREWVTPDGE---PCDDECVGGC--TGL
Cimex_lectularius_X1               ---CSDGLCWSEKNCQL-------------DLAES----------SCHQECVGGC--TGS
C._lectularius_X2                  ---CSDGLCWSEKNCQL-------------DLAES----------SCHQECVGGC--TGS
Cryptotermes_secundus              --SCESANCWGPGVCHSLR-----------------KN--ET---NCHRLCLGGC--TGL
Miniopterus_natalensis             NGQFVER-CWTHSHCQKVC----PTSCK--SHGCT-AE--GL---CCHSECLGNC--SEP
Callithrix_jacchus                 NGQFVER-CWTHSHCQKVC----PTICK--SHGCT-AE--GL---CCHSECLGNC--SEP
Heterocephalus_glaber_X1           NGQFIER-CWTHSHCQKVC----PTMCK--SHGCT-TE--GL---CCHSECLGNC--SEP
Dasypus_novemcinctus               NGQFVER-CWTHSHCQKVC----PTVCK--SHGCT-AE--GL---CCHSECLGNC--SAP
Tupaia_chinensis_X2                NGQFVER-CWTHSHCQKVC----PTICK--SHGCT-AE--GL---CCHSECLGNC--SEP
Homo_sapiens_X1                    NGQFVER-CWTHSHCQKVC----PTICK--SHGCT-AE--GL---CCHSECLGNC--SQP
Pongo_abelii_2                     NGQFVER-CWTHSHCQKVC----PTICK--SHGCT-AE--GL---CCHSECLGNC--SEP
Pan_troglodytes_X2                 NGQFVER-CWTHSHCQKVC----PTICK--SHGCT-AE--GL---CCHSECLGNC--SQP
Boleophthalmus_pectinirostris      NGLFNER-CWNQKHCQRMC----PAHCK--HRACT-KD--GQ---CCHEQCLGGC--SKP
Larimichthys_crocea_X2             NGHFSER-CWTQNQCQRMC----PVQCK--HRACT-KD--DQ---CCHDQCLGGC--LKP
Larimichthys_crocea                NGHFSER-CWTQNQCQRMC----PVQCK--HRACT-KD--DQ---CCHDQCLGGC--LKP
Lates_calcarifer                   NGHFSER-CWTQNHCQRMC----PVQCK--HRACT-KD--DQ---CCHDQCLGGC--LKP
Seriola_lalandi_dorsalis           NGHFSER-CWTQNHCQRMC----PVQCK--HRACT-KE--DQ---CCHDQCLGGC--LKP
Seriola_dumerili                   NGHFSER-CWTQNHCQRMC----PVQCK--HRACT-KE--DQ---CCHDQCLGGC--LKP
Zootermopsis_nevadensis            AEGCNKTMCWGSH----------THNCH--HRRRL-QS-------ECHELCLGGC--SGP
Parasteatoda_tepidariorum          STEKPTYLCWNSQKCQKIS-PCIKKECDLQNSSCY-NS--TH---CCHPECLGGC--TGP
Onthophagus_nigriventris           NGKPLCEYCWSSKHCQKKC----KSNCT----SCN-DL--GE---CCNNTCIGGC--SNN
Nicrophorus_vespilloides           KNGIMRFLCWNRVHCQKVC----PSSCK---TTCD-AK--GK---CCDEKCLGGC--RED
Tribolium_castaneum                NFDRDVHLCWNRQHCQKIC----PTKCK--H-SCN-AN--LE---CCDESCLGGC--SIN
Nilaparvata_lugens                 -SATNDNLCWNIQHCQKFC-----SKCEE-GTACM-DD--GT---CCDKNCLGGC--NGT
Blattella_germanica                NASSTKPLCWNLTKCQKIC----PTECG--SRSCT-DS--GM---CCHELCLGGC--TGP
Nasonia_vitripennis_X1             PTNLDERLCWNKQFCQKVCTSCQDKDGR--DRTCTVGSRPDNKTICCHENCLGGC--TGP
Microplitis_demolitor              LTKLDDNLCWNGQNCQKIC------KCG--NGACN-PS--GE---CCNPLCLGGC--TGQ
Neodiprion_lecontei                STKKDETLCWNRQYCQHVC----DKSCG--ISACN-NSMPGK---CCHSSCLGSC--TGT
Athalia_rosae                      VTKQDETLCWNRQHCQKVC----NTNCG--DAACS-NTVPVK---CCHPTCLGGC--TGP
Orussus_abietinus                  LTKPDETLCWNRQHCQRLC----DRKCG--DKVCS-KT--NE---CCHPSCIGGC--TGK
Dufourea_novaeangliae              SPKSQETLCWNQQHCQQIC----ERKCE--NNACD-IA--GK---CCHPSCLGRC--TGP
Megachile_rotundata_X1             STNPDEFLCWNQQHCQRTC----DGKCE--NNACD-DT--GK---CCHKSCLGTC--TGP
Melipona_quadrifasciata            QTKSDEYLCWNQQHCQRIC----DQKCE--SDACD-EF--GG---CCHPSCLGAC--TGP
Apis_mellifera_X1                  LTKQDEYLCWNQQHCQRIC----DRKCE--NNACD-EA--GN---CCHSSCLGTC--SGP
Eufriesea_mexicana_1               STKSDEYLCWNQQHCQRIC----DRKCE--NNACY-ESELER---CCHPSCLGTC--TGP
Eufriesea_mexicana_2               STKSDEYLCWNQQHCQRIC----DRKCE--NNACY-ESELER---CCHPSCLGTC--TGP
Bombus_terrestris                  QTKSDEYLCWNQQHCQQIC----DRKCE--NNACD-ES--GT---CCHPSCLGTC--TGP
Bombus_impatiens                   QTKSDEYLCWNQQHCQQIC----DRKCE--NNACD-ES--GT---CCHPSCLGTC--TGP
Polistes_dominula_X1               SNKPDETFCWNRQHCQRIC----D-RCL--EGACD-AS--GQ---CCHPSCLGGC--TGR
Cephus_cinctus_X1                  LTKPDETLCWNKQHCQKIC----DSKCG--DYACS-DS--GT---CCHPSCLGGC--TGL
Dinoponera_quadriceps_X2           QTKLGETMCWNRQHCQQIC----D-KCG--DGACD-NT--GA---CCHPSCLGGC--NGP
Harpegnathos_saltator_X1           QTKPDETMCWNRQHCQRIC----D-KCG--DNACD-DT--GA---CCHSSCLGGCT-TGP
Linepithema_humile_X2              QTKRDETLCWNRLHCQRIC----DPKCG--DGACD-DT--GA---CCHSSCLGGC--TGP
Linepithema_humile_X1              QTKRDETLCWNRLHCQRIC----DPKCG--DGACD-DT--GA---CCHSSCLGGC--TGP
Ooceraea_biroi                     QTKPDETLCWNRQHCQRIC----DRKCE--DCACD-DG--GR---NCHRSCLGGC--TGL
```

```
Camponotus_floridanus              QTKPDETLCWNRQHCQRVC----DHKCG--DNACDINT--GK---CCNSSCLGGC--TGL
Solenopsis_invicta                 QTKPDQNLCWNVQHCQRIC----DRKCE--DRACN-ST--GQ---CCHPFCLGGC--TGP
Pogonomyrmex_barbatus_X1           QMKHDQTLCWNRHHCQRIC----Q-DCG--DRACN-DK--GE---CCHPSCLGGC--TGS
Vollenhovia_emeryi_X1              QTKPDQTLCWNSHNCQQIC----DRTCE--NRACN-KT--GE---CCHQSCLGGC--TGS
Wasmannia_auropunctata             QTKPDQTLCWNRTHCQQIC----Q-KCE--DGACN-NK--GE---CCHASCLGGC--TEV
Cyphomyrmex_costatus               QTKPDQTLCWNRQHCQQIC----NWKCE--NRGCS-NT--GE---CCHPFCLGGC--TGP
Trachymyrmex_septentrionalis       QTKPDQSLCWNRQHCQQIC----DWKCD--NRACN-ST--GE---CCHPFCLGGC--TGP
Atta_colombica                     QTKFDQSLCWNRQHCQQIC----DWKCE--NRACN-ST--GE---CCHPFCLGGC--TGP
Trachymyrmex_cornetzi              QTKPDQTLCWNRQHCQQIC----DWKCE--NRACN-ST--GE---CCHPFCLGGC--TGS
Acromyrmex_echinatior              QTKLDQSLCWNRQYCQKIC----DWKCE--NRACN-ST--GV---CCHPFCLGGC--SGL
Atta_cephalotes                    QTKFDQSLCWNRQHCQQIC----DWKCE--NRACN-ST--GE---CCHPFCLGGC--TGP
                                           **                                    *   *:* *

Drosophila_melanogaster            -GNESCISCRNVSF-----NNICMDSCPKGYYQF-DSRCVTANECITLTKF-------ET
Halyomorpha_halys                  -SPKECTACKNFIH-----GNKCVSSCPPFTYEFETKRCVTKDECLAMNNTLLRPGK-TE
Rhodnius_prolixus                  -GPYNCKACRRFDH-----DGGCMKSCPSNRYAFENHYCVTEEECQD----------DV
Cimex_lectularius_X1               -GPSNCVSCRHLYF-----RGKCVPECPDGYYSFNSRHCISKEDCINSSSTYHKMRR-ND
C._lectularius_X2                  -GPSNCVSCRHLYF-----RGKCVPECPDGYYSFNSRHCISKEDCINSSSTYHKMRR-ND
Cryptotermes_secundus              -GSDQCSACTSVVD----EHGVCVDSCPTDRFEFVGRRCVTEEECRNSTVPPPPYLQRDL
Miniopterus_natalensis             NDPTKCAACRHFYL-----DGRCVETCPPPYYHFQDWRCVNFSFCQDLHNRCKNSRR---
Callithrix_jacchus                 GDPTKCVACRNYYL-----DGRCVDSCPPPYYQFQDWRCVNFSFCQDLHYKCKNSQR---
Heterocephalus_glaber_X1           DDPTKCVACRNFYL-----DGRCVASCPPPYYHFQDWRCVNFSFCQELHNKCKNSRR---
Dasypus_novemcinctus               DDPTKCMACRNFYL-----DGRCVETCPPQYYQFQDWRCVNFSFCQDLHNKCKNSRR---
Tupaia_chinensis_X2                NDPTKCVACRNFYL-----DGRCVETCPPPYYHFQDWRCVNFSFCQDLHNKCKNSRK---
Homo_sapiens_X1                    DDPTKCVACRNFYL-----DGRCVETCPPPYYHFQDWRCVNFSFCQDLHHKCKNSRR---
Pongo_abelii_2                     DDPTKCVACRNFYL-----DGRCVETCPPPYYHFQDWRCVNFSFCQDLHHKCKNSRR---
Pan_troglodytes_X2                 DDPTKCVACRNFYL-----DGRCVETCPPPYYHFQDWRCVNFSFCQDLHHKCKNSRR---
Boleophthalmus_pectinirostris      NSVEHCVACRGLQH-----QDTCVEQCPPNYYLYRGWRCVPFSFCQDLHNKCKREKERSK
Larimichthys_crocea_X2             NSASHCVACRGLQH-----GDNCVERCPENHFTYKGWRCVSFAFCQDLHNRCKREKEHSK
Larimichthys_crocea                NSASHCVACRGLQH-----GDNCVERCPENHFTYKGWRCVSFAFCQDLHNRCKREKEHSK
Lates_calcarifer                   SSAAHCVACRGLQH-----EGNCVERCPENHFTYKGWRCVSFAFCQDLHNGCKREKERSK
Seriola_lalandi_dorsalis           NSASHCVACRGLQH-----DGNCVERCPENHFTYKGWRCVSFAFCQDLHNRCKREKERSK
Seriola_dumerili                   NSASHCVACRGLQH-----EGNCVERCPENHFTYKGWRCVSFAFCQDLHNRCKREKERSK
Zootermopsis_nevadensis            -RADQCYVCRGVLN-----DGNCIQKCPRDRVSYLGRRCVTEDECRLLNHSYMDAWTPEY
Parasteatoda_tepidariorum          -LSSDCTVCRHVVW-----EGKCMKKCPPNTYEFMNRRCVREDECRNFSISHKHSSG-GE
Onthophagus_nigriventris           -DPKQCSVCRNITFYTEFNKTVCLDKCPEGLFKYLDRRCVTREECINSPKH--IEYT-TS
Nicrophorus_vespilloides           -NPRVCHVCRNFTM---GEDGTCTSYCPADRYEYLGRRCVTKEECINTPRP--LRQYNTI
Tribolium_castaneum                -DTKLCTVCKNLSM-GFGAKKQCMSSCPPDYYQYLERRCILKNECKDMKRP--LNFQQQG
Nilaparvata_lugens                 -GPTNCIACRGYLF-----QGTCVATCPVQTYQYLQRRCVLEKECYDMPKP--REIMSEE
Blattella_germanica                -NSTDCMACRDVVF-----ENRCIKKCPTNHFKYLNRRCMSDIECRNITKP--RERN-YD
Nasonia_vitripennis_X1             -SNLECKVCRDVVV----NQNECVSSCPNGTYQFFNRRCITKRECQMMRRP--REAP-AE
Microplitis_demolitor              -TASDCKICRDVIL----PDNKCAKQCPHNMYEFLNRRCIDEHKCRKMRKP--LEAF-GN
Neodiprion_lecontei                -GSQDCAVCRDVIV----DGNRCANSCPENTYMFLNRRCVQENECRKMKKP--REAF-DI
Athalia_rosae                      -TSQDCRVCREVVV----AGNRCAEKCPPNTYEFLNRRCVQENECRKMKKP--REAF-DI
Orussus_abietinus                  -DSNSCIACRELVL----PSNVCVPQCPSGTYEYLNRRCVQADECRRMKRP--REAS--M
Dufourea_novaeangliae              -TSRDCMVCKDVMT----ADRDCRDSCPNATLEFMNYRCIEENMCYRMEKP--LEAF-NV
Megachile_rotundata_X1             -TNRDCAVCKDVLT----GNKECSEHCPNGTLEFMDYRCIDEDTCLRTRKP--LESA-YN
Melipona_quadrifasciata            -TNRDCAACKDVIS----LDGQCVDRCPNGTLEFMNHRCIEEDQCLRLEKP--REAA--N
Apis_mellifera_X1                  -TNRDCTVCKDVVT----GDSECRERCPNGSLEFMNHRCIDEARCLQMEKP--KEVL-KN
Eufriesea_mexicana_1               -TNRDCAVCKDLAT----DGFECRERCPNGTLEFMNHRCIDEERCLRMEKP--REVF--Y
Eufriesea_mexicana_2               -TNRDCAVCKDLAT----DGFECRERCPNGTLEFMNHRCIDEERCLRMEKP--REVF--Y
Bombus_terrestris                  -TNRDCVVCKDVVT----DNNECREQCPNGTLEFMNHRCINEERCLAMQKP--REVF--N
Bombus_impatiens                   -TNRDCVVCKDVVT----DNNECREQCPNGTLEFMNHRCINEERCLGMQKP--REVF--N
Polistes_dominula_X1               -TANDCKVCKNVLI----GPNQCGERCPEGTYEFMNRRCIGEDECRRMVKP--REAL-SI
Cephus_cinctus_X1                  -TAQNCTVCREMVV----DVNQCVKRCPIGTYEFMNRRCIQENECRTMPKP--KEAL-GT
Dinoponera_quadriceps_X2           -TARDCKVCKNVVA-----NDECMDRCPSDTYEFMNRRCVEEQECRRLLKP--REAL-DS
Harpegnathos_saltator_X1           -TAKDCKVCRNLLV-----NGECVDRCPNGTYEFMNRRCIGIQECRQMRKP--REAL-DS
Linepithema_humile_X2              -TVNDCIVCRNVVI----NGTECKDRCLNGTYEFMNRRCIGEQECRRMPKP--REAS-DT
Linepithema_humile_X1              -TVNDCIVCRNVVI----NGTECKDRCLNGTYEFMNRRCIGEQECRRMPKP--REAS-DT
Ooceraea_biroi                     -TARDCIVCKGVII----DGHECKNRCPEGTYEFMNRRCIGEQECRLMPKP--REAL-DT
Camponotus_floridanus              -SNANCTVCRNVMV-----GTECKDSCPNGTYEFMNRRCVSEKMCREIRKP--REAL-DS
Solenopsis_invicta                 -TANDCSVCRNVVI----NGKECKDRCPRGKYKFMNRRCIEEWECRQMPKSMYKNED-EM
Pogonomyrmex_barbatus_X1           -TVNDCVVCRNIID-----GEQCKDSCPNNTYVFMNRRCFTKKNCFEKKKP--QEAR-DT
Vollenhovia_emeryi_X1              -TANDCAVCRNVVV----TDKVCADHCPDGMYEFMNRRCIGEKECRQMLKP--RETS-NM
Wasmannia_auropunctata             --TTNCTVCRNVLV-----DGKCEDRCPAGKLEFMNRRCIEKEECHKMPKP--REAS-DA
Cyphomyrmex_costatus               -TANDCIVCKNVVI----NDRECKDRCPKGKYEFMNRRCIEEQECRQMQKP--REAP-DA
Trachymyrmex_septentrionalis       -TANDCIVCKNVII-----NRECKDRCPIGKYEFMNRRCIEEQECLQMQKP--REAP-DA
Atta_colombica                     -TANDCTVCKNVII-----NKECKDRCPKGKYEFMNRRCIEEQECLQMQKP--REAP-DA
Trachymyrmex_cornetzi              -TANDCIVCKNVII-----NKECKDRCPKGKYEFMNRRCIEEQECLQMQKP--REAP-DA
Acromyrmex_echinatior              -TANNCTVCKNVII-----NRECKDRCPKGKYEFMNRRCIEEQECLQMQKP--REAP-DA
Atta_cephalotes                    -TANDCTVCKNVII-----NKECKDRCPKGKYEFMNRRCIEEQECLQMQKP--REAP-DA
```

```
                                        *  *             *   *      :    *.    *

Drosophila_melanogaster            NSVYS---GIPY-NGQCITHCPTGYQKSENKRM------------CEPCPGGK-CDKEC-
Halyomorpha_halys                  TKEN----WFVW-DSLCVNSCPRGLERD-KQLG------------CKKC-PGR-CVLHC-
Rhodnius_prolixus                  VWSKPQE-WFVW-NGTCIQDCPTGLEKT-TMSS------------CERCKDGK-CKKEC-
Cimex_lectularius_X1               GAHLV---WFIW-GNSCVTECPPKFEKTPDGKS------------CQRC-SGM-CKKVC-
C._lectularius_X2                  GAHLV---WFIW-GNSCVTECPPKFEKTPDGKS------------CQRC-SGM-CKKVC-
Cryptotermes_secundus              RAGLPNKAWIPF-NGTCSLTCPQGYQTYTDSGG--KQL-------CEAC-VDH-CRKIC-
Miniopterus_natalensis             -PGCHQ--YVIH-NNKCIPECPSGY-TM-NSSN---LM-------CTPC-LGP-CPKVCH
Callithrix_jacchus                 QSCYQ---YVIH-NNKCLPECPSGY-TM-NSSN---LM-------CTPC-LGP-CPKVCH
Heterocephalus_glaber_X1           -QGCHQ--YVIH-NDRCIPECPSGY-TM-NSSN---LM-------CTPC-LGP-CPKVCH
Dasypus_novemcinctus               -QGCHQ--YVIH-NNKCIAECPSGY-TM-NSSN---LM-------CTPC-LGP-CPKVCH
Tupaia_chinensis_X2                -QGCHQ--YVIH-NNKCIPECPSGY-TM-NSSN---LM-------CTPC-LGP-CPKVCH
Homo_sapiens_X1                    -QGCHQ--YVIH-NNKCIPECPSGY-TM-NSSN---LL-------CTPC-LGP-CPKVCH
Pongo_abelii_2                     -QGCHQ--YVIH-NNKCIPECPSGY-TM-NSSN---LL-------CTPC-LGP-CPKVCH
Pan_troglodytes_X2                 -QGCHQ--YVIH-NNKCIPECPSGY-TM-NSSN---LL-------CTPC-LGP-CPKVCH
Boleophthalmus_pectinirostris      NIDCHE--YVIH-NGACIPECPSGYTTV-NSFA---LM-------CTLC-TGL-CPKIC-
Larimichthys_crocea_X2             SPDCHE--YVIH-NGACIQECPSGYTTV-NSSS---LN-------CTPC-AGL-CPKVC-
Larimichthys_crocea                SPDCHE--YVIH-NGACIQECPSGYTTV-NSSS---LN-------CTPC-AGL-CPKVC-
Lates_calcarifer                   SPDCHE--YVIH-NGACIPECPSGYTTV-NSSS---LN-------CTPC-AGL-CPKVC-
Seriola_lalandi_dorsalis           NPDCHE--YVIH-NGACISECPSGYTTV-NSSS---LN-------CTPC-AGL-CPKVC-
Seriola_dumerili                   NPDCHE--YVIH-NGACISECPSGYTTV-NSSS---LN-------CTPC-AGL-CPKVC-
Zootermopsis_nevadensis            GRRC----W-PF-NGSCVDKCPPGYEE--QSNGTITVG-------CQPC-QGR-CSHEC-
Parasteatoda_tepidariorum          YPKVPDVYYKPF-QSQCTTECPSGYIVD-DNNP---HV-------CKKC-RAK-CPKVC-
Onthophagus_nigriventris           KQEYE---FKTFNNTSCVYKCPPKYTDDPKKKA------------CVSC-DSR-CKKVC-
Nicrophorus_vespilloides           PQEYP---YKTL-NKSCLLECPSDYTENYDQHK------------CFPC-LGR-CKKEC-
Tribolium_castaneum                GPEKP---FKIF-NNSCILECPPNYMSN-DTH-------------CIPC-QGN-CIKKC-
Nilaparvata_lugens                 VRPQP---WKPF-MNQCLIDCPPNYAEA-PIDNFRGLDPDRTYYRCEPC-KGP-CKKEC-
Blattella_germanica                MRMKP---YKPF-NDSCVLECPPGYVET-EFEH-NRYN-------CTPC-KGP-CRKEC-
Nasonia_vitripennis_X1             TRAYP---FKPF-NNECLMDCPAGYEEV-AEKD--MWS-------CKKC-TGP-CLREC-
Microplitis_demolitor              VREFP---YKPF-NNSCVIECPAGYMDD-ENNG--KAS-------CKKC-DGA-CSKVC-
Neodiprion_lecontei                VKEHP---YKPF-NNTCVIECPPGYMDE-EVNG--KAT-------CKKC-DGL-CLKEC-
Athalia_rosae                      VKEFP---YKPF-NNTCVTECPAGYMDE-EIDG--KSS-------CKKC-DGL-CLKEC-
Orussus_abietinus                  VKEFP---YKPF-NNTCVMECPAGYMDD-GVDA--RAS-------CKKC-DGP-CLKKC-
Dufourea_novaeangliae              KKSLP---YKPF-NGSCVIECPPDYIEE-ELNG--RLL-------CKKC-EGP-CQKKC-
Megachile_rotundata_X1             IKNYP---YKPF-NGSCVIECPPGYMDE-ETNG--KGS-------CKKC-EGP-CQKEC-
Melipona_quadrifasciata            GKNYS---YKPF-NGSCVLECPPGYTDE-ESSN--KAS-------CKKC-EGP-CQKEC-
Apis_mellifera_X1                  VKNYP---YKPF-NGSCVMECPPGYMDD-ESNG--RVS-------CKKC-EGS-CQKEC-
Eufriesea_mexicana_1               VSNYP---HKPF-NGSCIMECPPGYMDE-ESNG--KIS-------CKKC-EGR-CQKEC-
Eufriesea_mexicana_2               VSNYP---HKPF-NGSCIMECPPGYMDE-ESNG--KIS-------CKKC-EGR-CQKEC-
Bombus_terrestris                  VKNYP---YKSF-NGSCVMECPPGYMDE-ESNG--KVS-------CKKC-EGP-CQKEC-
Bombus_impatiens                   VKNYP---YKSF-NGSCVMECPPGYMDE-ESNG--KVS-------CKKC-EGP-CQKEC-
Polistes_dominula_X1               VKDYP---YKPF-NGSCIIECPAGYMDE-EVNN--KTS-------CRKC-KGP-CLKEC-
Cephus_cinctus_X1                  VKNYP---YKLFNESSCVIECPAGYMEE-EVNG--KPS-------CKKC-DGS-CLKEC-
Dinoponera_quadriceps_X2           VPNYP---YKPF-NGSCVIECPPGYMDV-EVDK--KMF-------CKKC-EGP-CLKEC-
Harpegnathos_saltator_X1           MQNYP---YKPF-NGSCVIECPAGYMDY-EVGK--NVS-------CKKC-EGP-CLKEC-
Linepithema_humile_X2              VKKYP---YKLF-NNTCVIECPAGYMET-EGND---PS-------CRKC-NGQ-CLKEC-
Linepithema_humile_X1              VKKYP---YKLF-NNTCVIECPAGYMET-EGND---PS-------CRKC-NGQ-CLKEC-
Ooceraea_biroi                     VKDYP---YKPF-NGSCVIECPAGYMES-DDYS---VS-------CRQC-EGP-CLKEC-
Camponotus_floridanus              VKYHP---YKPF-NGTCVIECPAGYMESTDNDQ--NLT-------CRKC-EGS-CLKEC-
Solenopsis_invicta                 IKQHP---YKPF-NDSCVIECPAGYMES-EDHG--NVS-------CQKC-EGRQCLREC-
Pogonomyrmex_barbatus_X1           VKLYP---YKLF-NDSCVMECPAGYMEN-ETTR----T-------CQKC-NGQWCMKEC-
Vollenhovia_emeryi_X1              VKMRP---YKPF-NGSCVLECPAGYMES-EERG--SVS-------CQKC-EGRWCLKEC-
Wasmannia_auropunctata             VKNYP---YKPFLNTSCVIECPPGYMES-ARGD------------CEQC-KGRWCLKEC-
Cyphomyrmex_costatus               MKSRP---YKLF-NGTCVTECPPGYMES-DNER-EHAS-------CRKC-EGRWCLKEC-
Trachymyrmex_septentrionalis       MKSRP---YKPF-NGTCVIECPPGYMES-EEKKGKPPT-------CQKC-EGPSCLKEC-
Atta_colombica                     MKSRP---YKPF-NGTCVIECPPGYMES-EEKKGKPPT-------CQKC-EGPSCLKEC-
Trachymyrmex_cornetzi              MKSRP---YKPF-NGTCVIECPPGYMES-EEKKGKPPT-------CQKC-EGPSCLKEC-
Acromyrmex_echinatior              MKSRP---YKPF-NGTCVIECPPGYMES-EEKKGKPPT-------CQKC-EGPSCLKEC-
Atta_cephalotes                    MKSRP---YKPF-NGTCVIECPPGYMES-EEKKGKPPT-------CQKC-EGPSCLKEC-
                                                   *   **                       *  *     *   *

Drosophila_melanogaster            --SSGL---IDSLERAREFHGCTIITGTEPLTISIKRESGAHVM-DELKYGLAAVHKIQS
Halyomorpha_halys                  --KGAN---VESIQSAQQLRYCTHIEGPLVIQL---RSGNQSFIQKELEENLGHIEEIMG
Rhodnius_prolixus                  --YGSV---VDSLEKAERLRKCTHILGSLEIQI---KSGQQSVVAAELEDSLGMIEEIQG
Cimex_lectularius_X1               --SMGN---VDSLQAAQNLRDCTHIDGNLEIHI---GTGKPEVVAKELEENLGMIEEIEG
C._lectularius_X2                  --SMGN---VDSLQAAQNLRDCTHIDGNLEIHI---GTGKPEVVAKELEENLGMIEEIEG
Cryptotermes_secundus              --GNATRIPVENIYDIQSLNGCTYVNGSLAIHL---M--GNKVV-EELENNFDAIEEISG
Miniopterus_natalensis             LLEGEK--TIDSVTSAQELRGCTVINGSLIINI---R-GGNNLA-AELEANLGLIEEISG
Callithrix_jacchus                 LLDREK--IIDSVTSAQDLRGCTVINGSLIINI---R-GGNNLA-VELEANLGLIEEISG
Heterocephalus_glaber_X1           LLEGEK--TIDSVTSAQELRGCTVINGSLIINI---R-GGNNLA-AELEANLGLIEEISG
```

```
Dasypus_novemcinctus               LMEGEK--TIDSVTSAQELRGCTVVNGSLIINI---R-GGNNLA-AELEANLGLIEEISG
Tupaia_chinensis_X2                LLEGEK--TIDSVTSAQELRGCTVVNGSLIINI---R-GGNNLA-AELEANLGLIEEISG
Homo_sapiens_X1                    LLEGEK--TIDSVTSAQELRGCTVINGSLIINI---R-GGNNLA-AELEANLGLIEEISG
Pongo_abelii_2                     LLEGEK--TIDSVTSAQELRGCTVINGSLIINI---R-GGNNLA-AELEANLGLIEEISG
Pan_troglodytes_X2                 LLEGEK--TIDSVTSAQELRGCTVINGSLIINI---R-GGNNLA-AELEANLGLIEEISG
Boleophthalmus_pectinirostris      --MGQK--TIDSVTAAQALRGCTILNGSLLIKL---R-GGNNIA-AELEASLGQLEEITG
Larimichthys_crocea_X2             --MGLK--TVDSVTAAQALRGCTVLNGSLVINL---R-GGNNIA-AELEASLGQLEEITG
Larimichthys_crocea                --MGLK--TVDSVTAAQALRGCTVLNGSLVINL---R-GGNNIA-AELEASLGQLEEITG
Lates_calcarifer                   --MGLK--TVDSVTAAQALRGCTVLNGSLVINL---R-GGNNIA-AELEASLGQLEEITG
Seriola_lalandi_dorsalis           --MGLK--TVDSVTAAQALRGCTVLNGSLVINL---R-GGNNIA-AELEASLGQLEEITG
Seriola_dumerili                   --MGLK--TVDSVTAAQALRGCTVLNGSLVINL---R-GGNNIA-AELEASLGQLEEITG
Zootermopsis_nevadensis            --VGGF---VSSIEDAQKLRGCTTILGSLEIFI---RGYGNHIV-RELEENLAAIEEIQG
Parasteatoda_tepidariorum          --APMT---VDHVGAAQNYKGCTRINGSLEIHI---Q-GGSNVI-KELEENLNMVEQIDG
Onthophagus_nigriventris           --PGIS---VNNIETAESLKECTHITGSIEIEV-----SGKNIV-PALNANLNMIEEIDG
Nicrophorus_vespilloides           --QGMV---IDSVVTAKRLRGCTHINGSLEIQI---RSGGRSVV-SELEENLSMIEEIDG
Tribolium_castaneum                --AGVN---VDSINLARQLKGCTHITSSLEIQI---R-GGRNVV-NELEESLGMIEEIDG
Nilaparvata_lugens                 --EGLN---VDSISSSQRLRGCTYIKGSLEIQI---R-GGYNVV-KELENNLNMIEEING
Blattella_germanica                --PGVN---VDSIAAAQKMRGCTYIRGSLEIQI---R-GGKNVV-KELEENLNMLEEIEG
Nasonia_vitripennis_X1             --TGIV---VDSIATAQKLRGCSHIKGNLEIAV---R-EGQNIV-HELEESLSNIEVITG
Microplitis_demolitor              --SGAS---VDSIASAQKLRGCTRIEGSLEIQI---K-GGKNMV-KELEDNLNMIEIIDG
Neodiprion_lecontei                --SGTN---VDSIASAQKLRGCTHITGSLEIQI---R-GGKNIV-KELEDSLSMIEEIDG
Athalia_rosae                      --SGAN---VDSIASAQKLRGCTRIMGSLEIQI---R-GGKNIV-KELEDSLSMIEEIDG
Orussus_abietinus                  --AGTS---VDSIASAQRLRGCTHIVGSLEIQI---R-GGKNIV-KELEDSLSMIEEIDG
Dufourea_novaeangliae              --TGAN---VDSIASAQQLRGCTRITGSLVIQI---R-GGKNIV-KELEDSLSTIEEIDG
Megachile_rotundata_X1             --AGDF---VDSIATAQQLRGCTRITGSLVIQI---R-GGKNIV-KELEDSLSTIEEIDG
Melipona_quadrifasciata            --TGMN---VDSIATAQKLRGCTHIVGSLEIQI---R-GGKNIV-KELEESLSMIQVIDG
Apis_mellifera_X1                  --AGAN---VDSIASAQKLRGCTHITGSLEIQI---R-GGKNIV-KELEDGLSTIEEIDG
Eufriesea_mexicana_1               --AGTN---VDSIASAQKLRGCTHITGSLEIQI---R-GGKNIV-KELEDSLSAIEEIDG
Eufriesea_mexicana_2               --AGTN---VDSIASAQKLRGCTHITGSLEIQI---R-GGKNIV-KELEDSLSAIEEIDG
Bombus_terrestris                  --AGAN---VDSIASAQKLRGCTHITGSLEIQI---R-GGKNIV-KELEDSLSTIEEIDG
Bombus_impatiens                   --AGAN---VDSIASAQKLRGCTHITGSLEIQI---R-GGKNIV-KELEDSLSTIEEIDG
Polistes_dominula_X1               --RGTS---VDSIASAQKLRGCTHITGSLEIQI---R-GGKNIV-NELEDSLNMIEEIDG
Cephus_cinctus_X1                  --AGAS---VDSIASAQKLRGCTHITGSLEIQI---R-GGKNIV-KEFEDSLSMIEEIDG
Dinoponera_quadriceps_X2           --FATN---VDSIASAQKLRGCTHIKGSLEIQI---R-GGKNIV-KELEESLNMIEEIDG
Harpegnathos_saltator_X1           --PATN---VDSIASAQKLRGCTHIKGSLEIQI---R-GGKNIV-KELEESLSMIEEIDG
Linepithema_humile_X2              --PGSN---VDSIASAQKLRGCTHIQGSLEIQI---R-GGKNIV-KELEDSLSMIEEIDG
Linepithema_humile_X1              --PGSN---VDSIASAQKLRGCTHIQGSLEIQI---R-GGKNIV-KELEDSLSMIEEIDG
Ooceraea_biroi                     --AGTN---VDSIASAQMLRGCTRISGSLEIQI---R-GGKNIV-KELEDSLNMIEEIDG
Camponotus_floridanus              --TGAN---VDSIASAQKLRGCTHIAGSLEIQI---R-GGKNIV-KELEDNLSTIEEIDG
Solenopsis_invicta                 --SAAK---VMSIETAQKLRGCTHLTGSLEIEI---R-GGKNIV-KELEDNLGMIEEIDG
Pogonomyrmex_barbatus_X1           --SAAN---VDSIASAQKLRGCTNIAGSLEIQI---R-GGKNIV-KELEDNLNMIEEIDG
Vollenhovia_emeryi_X1              --SGSN---VDSIASAQKLRGCTHIAGSIEIQI---R-SGKNMV-KELENSLSMIEEIDG
Wasmannia_auropunctata             --EGAN---VESIASAQKLRGCTHIKGSLEIQI---R-GGKNIV-KELEDSLGTLEEIDG
Cyphomyrmex_costatus               --NGAN---VESIASAQLLRGCTHIAGSLEIQI---R-GGKNIV-KELEDSLSTIEEIDG
Trachymyrmex_septentrionalis       --AGAN---VDSIASAQKLRGCTHIAGSLEIQI---R-GGKNIV-KELEDSLSTIEEIDG
Atta_colombica                     --AGAN---VDSIASAQKLRGCTHIAGSLEIQI---R-GGKNIV-KELEDSLSTIEEIDG
Trachymyrmex_cornetzi              --AGAN---VDSIASAQKLRGCTHIAGSLEIQI---R-GGKNIV-KELEDSLSTIEEIDG
Acromyrmex_echinatior              --AGAN---VDSIASAQKLRGCTHIAGSLEIQI---R-GGKNIV-KELEDSLSTIEEIDG
Atta_cephalotes                    --AGAN---VDSIASAQKLRGCTHIAGSLEIQI---R-GGKNIV-KELEDSLSTIEEIDG
                                            :  :      . *: : .   : :         .    :: .:  :  * .

Drosophila_melanogaster            SLMVHLTYGLKSLKFFQSLTEISG----DPPMDAD--KYALYVLDNRDLDELWGPNQT--
Halyomorpha_halys                  YLKIVRSFPLANLNFLMNLRVIHG-----SKLFSDPMNLSFIMLENQNLQTIWDWDNRPA
Rhodnius_prolixus                  QLKITRSFPLVSLDFFKNLRIIQG---DRHFYFNS--NYSLFIKDNQNLMTIWNWDKRPA
Cimex_lectularius_X1               NLRIIHSFPLDSLGFFKNLKVIHGYKDKKGTNNLV--NHSLFVLDNQNLKEIWNWDTRPA
C._lectularius_X2                  NLRIIHSFPLDSLGFFKNLKVIHGYKDKKGTNNLV--NHSLFVLDNQNLKEIWNWDTRPA
Cryptotermes_secundus              YLKISHSYPLVTLNFFKKLKVIHG-----LRLESD--KFALVIVGNDNLQELWNWEARP-
Miniopterus_natalensis             YLKIRRSYALVSLSFFRKLRLIQG-----DTLEIG--NYSFYALDNQNLRQLWDWNKH--
Callithrix_jacchus                 YLKIRRSYALVSLAFFRKLRLIRG-----ETLEAG--NYSFYALDNQNLRQLWDWSKH--
Heterocephalus_glaber_X1           YLKIRRSYALVSLSFFRKLRLIRG-----ETLEIG--NYSFYALDNQNLRQLWDWSKH--
Dasypus_novemcinctus               YLKIRRSYALVSLSFFRKLRVIRG-----ETLEVG--NYSFYALDNQNLRQLWDWSKH--
Tupaia_chinensis_X2                YLKIRRSYALVSLSFFRKLRVIRG-----ETLEIG--NYSFYALDNQNLRQLWDWSKH--
Homo_sapiens_X1                    YLKIRRSYALVSLSFFRKLRLIRG-----ETLEIG--NYSFYALDNQNLRQLWDWSKH--
Pongo_abelii_2                     YLKIRRSYALVSLSFFRKLRLIRG-----ETLEIG--NYSFYALDNQNLRQLWDWSKH--
Pan_troglodytes_X2                 YLKIRRSYALVSLSFFRKLRLIRG-----ETLEIG--NYSFYALDNQNLRQLWDWSKH--
Boleophthalmus_pectinirostris      FLAVQRSYALVSLFFFRKLRVIRG-----EELYNG--NFSFYALDNQNLRQLWDWSKHK-
Larimichthys_crocea_X2             YLTVRRSYALVSLSFFRKLRVIRG-----EEQEIG--NYSFYALDNQNLRQLWDWSKHK-
Larimichthys_crocea                YLTVRRSYALVSLSFFRKLRVIRG-----EEQEIG--NYSFYALDNQNLRQLWDWSKHK-
Lates_calcarifer                   YLTVRRSYALVSLSFFRKLRVIRG-----EEQEIG--NYSFYALDNQNLRQLWDWSKHK-
Seriola_lalandi_dorsalis           YLTVRRSYALVSLSFFRKLRVIRG-----EEQEIG--NYSFYALDNQNLRQLWDWSKHK-
Seriola_dumerili                   YLTVRRSYALVSLSFFRKLRVIRG-----EEQEIG--NYSFYALDNQNLRQLWDWSKHK-
```

```
Zootermopsis_nevadensis            YLKVSRSFPLTSLNFLKNLRIIHG-----NKLEDN--WYSLVVLENQNLQELWDWSSRPQ
Parasteatoda_tepidariorum          YLKIARSFPLVSLNFLKSLTVIKG-----DTLDKK--DYSLLVLDNQNLQELWDWKLRT-
Onthophagus_nigriventris           YLKIVRSYPLISLSFFKSLKVIHG-----NAFENG--KYSFIVLDNQNLQNFWDWENRT-
Nicrophorus_vespilloides           YIKVVRSFPLVSLNFFKNLKVIRG-----NSLEFK--KYVVIVSDNQNLQDLWDFENRT-
Tribolium_castaneum                YLKVVRSFPLVSLNFLKNLKVIHG-----RQLESQ--KYVFVVLDNQNLQELWNWENNK-
Nilaparvata_lugens                 YLKIVRSFPLVSLNFLKKLKVIHG-----EKLESG--KYALVVLDNQNLVELWDFGPSR-
Blattella_germanica                YLKIVRSFPLISLNFLRKLRVVHG-----KKLDSN--KYAFVLPDNQNLQELWDWDTRP-
Nasonia_vitripennis_X1             YLKIFRSFPLISLSFLKNLVEIKG-----ETLDMK--DYSLVVLDNQNLQQLWNWTTRP-
Microplitis_demolitor              YLKIVRSFPLISLNFLKNLKLING-----SQLEND--KYTLAVLDNQNLQELWDWDTHP-
Neodiprion_lecontei                YLKIVRSFPLISLNFLKNLHTIHG-----KELESG--KYTFVVLDNQNLQELWDMESHK-
Athalia_rosae                      YLKIVRSFPLISLNFLKNLRTIHG-----KTLESG--KYSLVVLDNQNLQELWDLETHK-
Orussus_abietinus                  YLKIVRCFPLISLNFLKNLKIIRG-----NSLDNS--KYTLAVLDNQNLQELWDWDSSK-
Dufourea_novaeangliae              NLKIVRSFPLISLNFLKNLRLIRG-----NDVENS--KYSLLVMDNQNLQELWDWSWHR-
Megachile_rotundata_X1             YLKIVRSFPLISLNFLKNLRVIRG-----NEINNS--KYSLLVMDNQNLQELWDWSWHR-
Melipona_quadrifasciata            YLKIVRSFPLISLSFLKNLRAIRG-----NDIDNS--KYSLLVMDNQNLQELWDWDTHD-
Apis_mellifera_X1                  YLKIVRSFPLISLNFLKNLRVIRG-----NDIDNS--KYTLLVMDNQNLQELWDWSLHK-
Eufriesea_mexicana_1               YLKIVRSFPLISLNFLKNLRIIRG-----NDIDNS--KYSLLVMDNQNLQELWDWSLHK-
Eufriesea_mexicana_2               YLKIVRSFPLISLNFLKNLRIIRG-----NDIDNS--KYSLLVMDNQNLQELWDWSLHK-
Bombus_terrestris                  YLKIVRSFPLISLNFLKNLRVIRG-----NDIDNS--KYSLLVMDNQNLQELWDWSLHK-
Bombus_impatiens                   YLKIVRSFPLISLNFLKNLRVIRG-----NDIDNS--KYSLLVMDNQNLQELWDWSLHK-
Polistes_dominula_X1               YLKIVRSFPLISLNFLKSLRVIRG-----NSLDNN--KYTLAVLDNQNLQELWDWSTHP-
Cephus_cinctus_X1                  YLKIVRSFPLISLNFLKKLRLIKG-----NQLDNG--KYTLAVLDNQNLQELWDWDTHP-
Dinoponera_quadriceps_X2           YLKIVRSFPLISLNFLKSLRIIRG-----NVLENG--KYTLSVFDNQNLQELWDWSTHK-
Harpegnathos_saltator_X1           YLKIVRSFPLISFNFLHRLRVIRG-----KILDNA--KYTLSVFDNQNLQELWDWSTHK-
Linepithema_humile_X2              YLKIVRSFPLISLNFLKNLRVIRG-----NTLENH--KYSLAVFDNQNLQELWDWETHP-
Linepithema_humile_X1              YLKIVRSFPLISLNFLKNLRVIRG-----NTLENH--KYSLAVFDNQNLQELWDWETHP-
Ooceraea_biroi                     YLKIVRSFPLISLNFLKNLRVIRG-----NELDNG--KYSLAVLDNQNLQELWDWRTHR-
Camponotus_floridanus              YLKVIRSFPLISLNFLKNLRVIRG-----NTLENN--KYSLAVLDNQNLQELWDWSTHP-
Solenopsis_invicta                 SLRIVRSFPLISLNFLKNLRVIRG-----NDSES---KYSLSVLDNQNLQELWDWNTHK-
Pogonomyrmex_barbatus_X1           YLKIVRSFPLISLNFLKNLRVIHG-----NELENG--KYSLAVFDNQNLQELWDWNTHS-
Vollenhovia_emeryi_X1              YLKIVRSFPLISLNFLKNLRVIRG-----NQLENG--KYSLAVLDNQNLQELWDWNTHP-
Wasmannia_auropunctata             YLKIVRSFPLISLNFLKNLRVIRG-----KDLENG--KYSLMVLDNQNLQELWDWSTHP-
Cyphomyrmex_costatus               YLKIIRSFPLISLNFLKNLRVIRG-----KTLENG--KYSLAVLDNQNLQELWDWNTHP-
Trachymyrmex_septentrionalis       YLKIVRSFPLISLNFLKNLRVIRG-----QTLENG--KYSLAVLDNQNLQELWDWNTHP-
Atta_colombica                     YLKIVRSFPLISLNFLKNLRVIRG-----QTLENG--KYSLAVLDNQNLQELWDWNTHP-
Trachymyrmex_cornetzi              YLKIVRSFPLISLNFLKNLRVIRG-----QTLENS--KYSLAVLDNQNLQELWDWNTHP-
Acromyrmex_echinatior              YLKIVRSFPLISLNFLKNLRVIRG-----QTLENG--KYSLAVLDNQNLQELWDWNTHP-
Atta_cephalotes                    YLKIVRSFPLISLNFLKNLRVIRG-----QTLENG--KYSLAVLDNQNLQELWDWNTHP-
                                    : :   : * .: *:  *  : *                .    * :*  :*.

Drosophila_melanogaster            ---VFIRK------GGVFFHFNPKLCVSTINQLLPMLASKPKFFEKSDVGADSNGNRGSC
Halyomorpha_halys                  KRNFTILA------GRLFFHYNPSLCLKHIYELGKI-AGIEKIKNS-EVAKESNGNKFAC
Rhodnius_prolixus                  GRNFTINM------GRPLFNDNPKLCIKHIRELTTI-AGFKDVKDT-EVT-KQNGVKFAC
Cimex_lectularius_X1               GRNFKILN------GYPFFHYNPMLCYKHIEELTSI-AGIQDIKTI-EITRESNGGRFAC
C._lectularius_X2                  GRNFKILN------GYPFFHYNPMLCYKHIEELTSI-AGIQDIKTI-EITRESNGGRFAC
Cryptotermes_secundus              -RNFTIEK------GTISLHSNPKLCIAEIDELRDI-AGLHNNSAD-EISYESNGNEVTC
Miniopterus_natalensis             --NLTITQ------GKLFFHYNPKLCLSEIHKMEEI-SGTKGRQERNDIALKTNGDQASC
Callithrix_jacchus                 --NLTITQ------GKLFFHYNPKLCLSEIHKMEEI-SGTKGRQEKNDIALKTNGDQASC
Heterocephalus_glaber_X1           --NLTITQ------GKLFFHYNPKLCLSEIHKMEEV-SGTKGRQERNDIALKTNGDQASC
Dasypus_novemcinctus               --NLTITQ------GKLFFHYNPKLCLSEIHKMEEV-SGTKGRQERNDIALKTNGDQASC
Tupaia_chinensis_X2                --NLTITQ------GKLFFHYNPKLCLSEIHKMEEV-SGTKGRQERNDIAPKTNGDQASC
Homo_sapiens_X1                    --NLTITQ------GKLFFHYNPKLCLSEIHKMEEV-SGTKGRQERNDIALKTNGDQASC
Pongo_abelii_2                     --NLTITQ------GKLFFHYNPKLCLSEIHKMEEV-SGTKGRQERNDIALKTNGDQASC
Pan_troglodytes_X2                 --NLTITQ------GKLFFHYNPKLCLSEIHKMEEV-SGTKGRQERNDIALKTNGDQASC
Boleophthalmus_pectinirostris      ---LTIQK------GHIVFLYNSKLCISEILRMVEV-TGASIQKE--DIT-KTNGEQASC
Larimichthys_crocea_X2             ---LTILQ------GRMFFHYNSKLCMSEIRKMEGV-TGTKERQVKNDIASKTNGDQASC
Larimichthys_crocea                ---LTILQ------GRMFFHYNSKLCMSEIRKMEGV-TGTKERQVKNDIASKTNGDQASC
Lates_calcarifer                   ---LTILQ------GRMFFHYNSKLCMSEIRKMEEV-TGTKERLVKNDIASKTNGDQASC
Seriola_lalandi_dorsalis           ---LTILQ------GRMFFHYNSKLCMSEIRKMEEV-TGTKERHIKNDIASKTNGDQASC
Seriola_dumerili                   ---LTILQ------GRMFFHYNSKLCMSEIRKMEEV-TGTKERHIKNDIASKTNGDQASC
Zootermopsis_nevadensis            GLQLKIVK------GHLRFHYNPKLCMSEIDKLQNI-SGAPNYTNF-DVARESNGDKVAC
Parasteatoda_tepidariorum          -NKLKILN------GKIFFHFNSKLCPSKIQELKTY-AEVADWDER-DVSPSSNGDRVAC
Onthophagus_nigriventris           ---LQINH------GKLFFHFNPKLCYKLIKQLAEK-ANITKFDDW-DVAENSNGDKTAC
Nicrophorus_vespilloides           ---LKIDH------GGLFFHFNPKLCMYKIEALREM-ANLSEFEER-DVASNSNGDRIAC
Tribolium_castaneum                --TLKIDT------GRLFFHFNPKLCIREIEKLQNI-THIVDVTEL-EVAKNSNGDKIAC
Nilaparvata_lugens                 EERLRITR------GKLFFHFNPKLCYFKIDNLRKY-LGMPEFTDN-EVARNSNGDKVAC
Blattella_germanica                -EKLQILN------GRLFFHFNPKLCMYMIEKLKQV-ANLPDFTDL-EVAANSNGDKMAC
Nasonia_vitripennis_X1             --PLKIGSSNPV--PKVSFHYNPKLCLQTIEELRER-TGLNPFREV-EVSPTSNGDKVAC
Microplitis_demolitor              --PITIKSKDGP--AKIFFHFNPKLCIHNIEKLRDV-ANLSQFTDL-EVAPNSNGDKVAC
Neodiprion_lecontei                --SMRILANNGP--VKVFFHFNPKLCLDTIEKFRKG-AGLPEFSDL-EVAPNSNGDKVAC
Athalia_rosae                      --SIKILARDGP--AKVFFHFNPKLCLDTIEKFRKA-ADLPEFTEL-EVAPNSNGDKVAC
```

```
Orussus_abietinus                  --KIKILSKDGP--AKIFFHFNPKLCLKKIEKLREV-AGLQEFTDL-DVAPNSNGDKVAC
Dufourea_novaeangliae              --DIKIESKGEP--GKMFFHMNPKLCLYKIEALRER-AGLKKFTDY-EVAPNSNGDKVAC
Megachile_rotundata_X1             --DIKILSKDGP--GKIFFHLNPKLCLYKIEMLREK-AGVAPFTDY-EVAPNSNGDKVAC
Melipona_quadrifasciata            --GIKILSKDGP--GRIFFHLNPKLCLYKIETLRKK-AGLGPFTEY-DVAPNSNGDKVAC
Apis_mellifera_X1                  --EISIKSKDGP--GKIFFHLNPKLCLYKIEMLREK-AGLGPFTDY-EVAPNSNGDKVAC
Eufriesea_mexicana_1               --DIKIQSRDGP--GKIFFHLNPKLCLYKIEMLRKK-AGLGPFTDY-EVAPNSNGDKVAC
Eufriesea_mexicana_2               --DIKIQSRDGP--GKIFFHLNPKLCLYKIEMLRKK-AGLGPFTDY-EVAPNSNGDKVAC
Bombus_terrestris                  --DIKILSKGGI--GKIFFHLNPKLCLYKIEMLREK-AGLRPFTDY-EVAPNSNGDKVAC
Bombus_impatiens                   --DIKILSKGGI--GKIFFHLNPKLCLYKIEMLREK-AGLRPFTDY-EVAPNSNGDKVAC
Polistes_dominula_X1               --NIAIYSKDGP--AKVFFHFNPKLCFYKIEALRKK-AHLQEFTDL-EVAPNSNGDKVAC
Cephus_cinctus_X1                  --KIQIRSNGAP--GKVFFHFNPKLCLHKIEKLREV-ANLAEFSPL-EVALNSNGDKVAC
Dinoponera_quadriceps_X2           --NITILSKEGP--PKVFFHFNPKLCLSKIETLRQK-AKLNAFTDY-DVAPNSNGDKVAC
Harpegnathos_saltator_X1           --NITILSKEGH--AKIFFHYNPKLCLYKIEKLREV-ANLEYFTDY-DVAPNSNGDKVAC
Linepithema_humile_X2              --NLTILSPAGP--AKLFFHFNPKLCLQKIEILRDK-AKLEDFNDH-DVASSSNGDKVAC
Linepithema_humile_X1              --NLTILSPAGP--AKLFFHFNPKLCLQKIEILRDK-AKLEDFNDH-DVASSSNGDKVAC
Ooceraea_biroi                     --NISIMSKDGP--AKIFFHFNPKLCLYKIETLREK-ARLNEFTDL-EVAPNSNGDKVAC
Camponotus_floridanus              --NLTILSSAGS--PKVFFHFNPKLCMYKIDMLREK-AKLDNFTEH-DVAINSNGDKVAC
Solenopsis_invicta                 --NITILAKTGP--ARLFFHFNPKLCLHEIEKLRLK-ANLEEFTDH-DVASNSNGDKIAC
Pogonomyrmex_barbatus_X1           --NITILAHNGAAKAKLFFHFNPKLCLHEIEKLREK-AQLDEFTDH-DVASTSNGDKVAC
Vollenhovia_emeryi_X1              --NITILSEKGP--AKLFFHFNPKLCLHEIERLREK-ARLEEFTDH-DVASSSNGDKVAC
Wasmannia_auropunctata             --NITILSSVGP--AKLFFHFNPKLCLHEIEKLREK-AKLEEFNDH-DVASSSNGDKVAC
Cyphomyrmex_costatus               --NITILSSKGP--AKLFFHFNPKLCLHEIEKLREK-AKLDEFTDL-DVASSSNGDKVAC
Trachymyrmex_septentrionalis       --NITILSSGGP--AKLFFHFNPKLCLHEIEKLREK-AKLEEFTDH-DVASSSNGDKVAC
Atta_colombica                     --NITILSSGGP--AKLFFHFNPKLCLHEIEKLREK-AKLEEFTDH-DVASSSNGDKVAC
Trachymyrmex_cornetzi              --NITILSSGGP--AKLFFHFNPKLCLHEIEKLREK-AKLEEFTDH-DVASSSNGDKVAC
Acromyrmex_echinatior              --NITIMSSGGP--AKLFFHFNPKLCLHEIEKLREK-AKLEEFTDH-DVASSSNGDKVAC
Atta_cephalotes                    --NITILSSGGP--AKLFFHFNPKLCLHEIEKLREK-AKLEEFTDH-DVASSSNGDKVAC
                                      . *            :  *. **   *  :              ::    **   :*

Drosophila_melanogaster            GTAVLNVTLQS-VGANSAMLNVTTKVEIGEPQKPSNATIVFK--DPRA----FIGFVFYH
Halyomorpha_halys                  NIIDLNIKVHF-RNSTCITIFI--------------APPKFR--DAENQENYLLRYLAYY
Rhodnius_prolixus                  NLVELNISAHL-TFSQSIVIHI--------------HKPDFN--NT-S----LIKYIAYY
Cimex_lectularius_X1               NKLSLNVTVHL-VYENAIALAI--------------EQPEFT--NMAK-YYQMMRFVAYY
C._lectularius_X2                  NKLSLNVTVHL-VYENAIALAI--------------EQPEFT--NMAK-YYQMMRFVAYY
Cryptotermes_secundus              IVIKMNASSVV-INSTSVTIQW--------------DPFHVP--RGRG---TLLGYTVHY
Miniopterus_natalensis             ENELLKFSYIR-TSFDKILLRW--------------EPYWPP--DFRD----LLGFMLFY
Callithrix_jacchus                 ENELIKFSSIR-TSSDKILLKW--------------EPYWPP--DFRD----LLGFMLFY
Heterocephalus_glaber_X1           ENELLKFSSIR-TSSDKILLRW--------------EPYWPP--DFRD----LLGFMLFY
Dasypus_novemcinctus               ENELLKFSDIR-TSFDKILLRW--------------EPYWPP--DFRD----LLGFMLFY
Tupaia_chinensis_X2                ENELLKFSHIR-TSSDKILLKW--------------EPYWPP--DFRD----LLGFMLFY
Homo_sapiens_X1                    ENELLKFSYIR-TSFDKILLRW--------------EPYWPP--DFRD----LLGFMLFY
Pongo_abelii_2                     ENELLKFSYIR-TSFDKILLRW--------------EPYWPP--DFRD----LLGFMLFY
Pan_troglodytes_X2                 ENELLKFSYIR-TSFDKILLRW--------------EPYWPP--DFRD----LLGFMLFY
Boleophthalmus_pectinirostris      ETHSLTFSQIR-TSSDKIMVKW--------------KAFWPT--DFRD----LLGFMVLY
Larimichthys_crocea_X2             ENHVLKFTMIR-TMSDKIMVKW--------------EAFWPP--DFRD----LLGFMVLY
Larimichthys_crocea                ENHVLKFTMIR-TMSDKIMVKW--------------EAFWPP--DFRD----LLGFMVLY
Lates_calcarifer                   ENRVLKFTQIR-TMSDKIMVKW--------------EAFWPP--DFRD----LLGFMVLY
Seriola_lalandi_dorsalis           ENHVLRFTQIR-TMSDKIMVKW--------------EPFWPP--DFRD----LLGFMVLY
Seriola_dumerili                   ENHVLRFTQIR-TMSDKIMVKW--------------EPFWPP--DFRD----LLGFMVLY
Zootermopsis_nevadensis            NVVNLNVNSTV-TSSTSVLIEW--------------ERCVLP--DPRS----VLGYMVYY
Parasteatoda_tepidariorum          DVTNMNVTVRN-ASPSIVTVKW--------------DSFGKNLIDDRS----LLGYVIYY
Onthophagus_nigriventris           EIKDLIIEIQS-KSSTSVQLYW--------------VPFNIT--DSRK----LLSYQIYH
Nicrophorus_vespilloides           NVQNLVVNVTN-SGPRAIVLAW--------------APFEMK--DHRA----LLGYVVYS
Tribolium_castaneum                ELQVLKVDKPKVVNSKGVVLEW--------------EPFKID--DPRK----LLGYIVYS
Nilaparvata_lugens                 NISLLDARVET-RSSKGVIIKW--------------KEFEHY--DPRT----LLGYIIYF
Blattella_germanica                NVTKLRATVTK-RDHVAVLIEW--------------QQFEHY--DPRT----LLGYVVYY
Nasonia_vitripennis_X1             NVTEIRTIVFS-ISSKAAMINW--------------EPFEHH--DMRT----LLGYVLYS
Microplitis_demolitor              NVTELKVQVIK-KTAEAALIEW--------------KAFEHH--DPRS----LLGYVVYF
Neodiprion_lecontei                NVTKLHTRVQK-KTSNAALIAW--------------EPFDHH--DQRS----LLGYVVYF
Athalia_rosae                      NVTKLRTRVTK-KTSNAALIEW--------------EAFRHH--DPRS----LLGYVVYF
Orussus_abietinus                  DVTELVTRITR-INTAAALIEW--------------DAFEHH--DPRS----LLGYVVYV
Dufourea_novaeangliae              NVTELKTRVGN-KSPWGAVIEW--------------EPFIHH--DARS----LLSYVVYF
Megachile_rotundata_X1             NVTELKTRVGK-KSAYGAVIEW--------------EPFVHH--DVRS----LLGYVVYF
Melipona_quadrifasciata            NVTELMTMVGK-KSPWGAVIEW--------------EPFVHH--DARS----LLGYVVYY
Apis_mellifera_X1                  NVTELKTRVGK-KSPWGAVIEW--------------EPFVHH--DARS----LLGYVVYF
Eufriesea_mexicana_1               NVTELKTRVGK-KSPWGAVIEW--------------EPFVHH--DARS----LLGYVVYF
Eufriesea_mexicana_2               NVTELKTRVGK-KSPWGAVIEW--------------EPFVHH--DARS----LLGYVVYF
Bombus_terrestris                  NVTELRTRVGK-KSPWGAVIEW--------------EPFIHH--DARS----LLGYVVYF
Bombus_impatiens                   NVTELRTRVGK-KSPWGAVIEW--------------EPFIHH--DARS----LLGYVVYF
Polistes_dominula_X1               NVTELKTVVTK-TTSDAALIEW--------------EQFTHH--DPRS----LLGYVVYF
Cephus_cinctus_X1                  NVTDIITRVTK-KTSEAALIEW--------------QAFKHH--DPRS----LLGYVVYF
```

```
Dinoponera_quadriceps_X2           NVTELQTKVVK-ETSRGAIIEW--------------EAFMHH--DTRS----LLGYVVYF
Harpegnathos_saltator_X1           NVTNLYTKVIQ-KTSRGAIIEW--------------EAFMHH--DTRS----LLGYVVYF
Linepithema_humile_X2              NVTQLKTKVIK-KTSRGAIIEW--------------EAYTHH--DTRS----LLGYVVYF
Linepithema_humile_X1              NVTQLKTKVIK-KTSRGAIIEW--------------EAYTHH--DTRS----LLGYVVYF
Ooceraea_biroi                     NVTELKTKIIK-KTSRGAIIEW--------------ETFMHH--DTRS----LLGYIVYF
Camponotus_floridanus              NVTELKTKITK-KTSRGAIIEW--------------EPFSHH--DYRS----LLGYVVYF
Solenopsis_invicta                 NVTELETRVTW-RTPVGAIIKW--------------TAFKHH--DIRS----LLGYVVYF
Pogonomyrmex_barbatus_X1           NVTELKTKVTK-KTPRGAIIEW--------------RAFTHH--DTRS----LLGYVVYF
Vollenhovia_emeryi_X1              NVTELTPKVIK-RSSRAAIIEW--------------NIFTHH--DTRS----LLGYVVYF
Wasmannia_auropunctata             NVTELKTRITK-KTSRGVIIEW--------------KGFTHH--DTRS----LLGYVVYF
Cyphomyrmex_costatus               NVTELKTKVTR-KSSRGAIIQW--------------EAFTHH--DTRS----LLGYVLYF
Trachymyrmex_septentrionalis       NVTELKTKVTK-KTSRGAIIEW--------------KAFTHH--DTRS----LLGYVVYF
Atta_colombica                     NVTELKTKVTK-KSSRGAIIEW--------------KAFTHH--DTRS----LLGYVVYF
Trachymyrmex_cornetzi              NVTELKTKVTK-KSSRGAIIEW--------------KAFTHH--DTRS----LLGYVVYF
Acromyrmex_echinatior              NVTELKTKVTK-KSSRGAIIEW--------------KAFTHH--DTRS----LLGYVVYF
Atta_cephalotes                    NVTELKTKVTK-KSSRGAIIEW--------------KAFTHH--DTRS----LLGYVVYF
                                       :              :                                .: :

Drosophila_melanogaster            MIDPYGNSTK-SSDDPC-DDRWKVS-----------------------------SPEKSG
Halyomorpha_halys                  IKAPFRNVTADYEINECGSYRWTVDDVINYP-----------------------EKNYTK
Rhodnius_prolixus                  MEEPYGNLTTAIPSDDCEENAWKLNDVAISEEDK--------------------SMSNLK
Cimex_lectularius_X1               MEAPHQNVTELNTNE-CSESKWKMHDISGLNEPFD-------------------GGPPNG
C._lectularius_X2                  MEAPHQNVTELNTNE-CSESKWKMHDISGLNEPFD-------------------GGPPNG
Cryptotermes_secundus              METAVRNVTLETDNDACGVDKWKVAYVVLTTN----------------------QSATPT
Miniopterus_natalensis             KEAPYQNVTEFDGQDACGSNSWTVVDIDPPLRSN--------------------DPKSQN
Callithrix_jacchus                 KEAPYQNVTEFDGQDACGSNSWTVVDIDPPQRSS--------------------DPTSQS
Heterocephalus_glaber_X1           KEAPYQNVTEFDGQDACGSNSWTVVDIDPPLRSN--------------------DPKLQN
Dasypus_novemcinctus               KEAPYQNVTEFDGQDACGSNSWTVVDIDPPQRSN--------------------DPKSQS
Tupaia_chinensis_X2                KEAPYQNVTEFDGQDACGSNSWTVVDIDPPQRSN--------------------DPKSQN
Homo_sapiens_X1                    KEAPYQNVTEFDGQDACGSNSWTVVDIDPPLRSN--------------------DPKSQN
Pongo_abelii_2                     KEAPYQNVTEFDGQDACGSNSWTVVDIDPPLRSN--------------------DPKSQN
Pan_troglodytes_X2                 KEAPYQNVTEFDGQDACGSNSWTVVDIDPPLRSN--------------------DPKSQN
Boleophthalmus_pectinirostris      KEAPFQNVTEFDGQDACGSNSWVIADVEPPPRITD-------------------SEKEQM
Larimichthys_crocea_X2             KEAPFRNVTEFDGQDACGSNSWVIADVDPPRRSTE-------------------GDKEQS
Larimichthys_crocea                KEAPFRNVTEFDGQDACGSNSWVIADVDPPRRSTE-------------------GDKEQS
Lates_calcarifer                   KEAPFQNVTEFDGQDACGSNSWVIADVDPPRRATE-------------------GDKEQF
Seriola_lalandi_dorsalis           KEAPFQNVTEFDGQDACGSNSWVIADVDPPRRASE-------------------GDKEQF
Seriola_dumerili                   KEAPFQNVTEFDGQDACGSNSWVIADVDPPRRASE-------------------GDKEQF
Zootermopsis_nevadensis            IEAPEQNLTEHDGLDACGDCGWHVVDVPYDTDEKNTV-----------------DTSQYG
Parasteatoda_tepidariorum          RETLFRNISIFEGRDACGTDAWTVRDVEYVENTN-------------------------N
Onthophagus_nigriventris           IEAPVQNFSIFDSRDACGGDGWSIQDVPIPY-----------------------DHHNVS
Nicrophorus_vespilloides           IKAPEQNITLYDGRDACGGDGWRVDDIYIDNQKP--------------------------
Tribolium_castaneum                IEAPTQNVTLYDGRDACGGDGWRVDDVAIPENEM--------------------------
Nilaparvata_lugens                 IEAPYQNISLYDGRDACGGDGWRVIDVSPLEKDNETTTLNE-------------DKNKTK
Blattella_germanica                IEAPYQNLTLYDGRDACGGDGWHVTDMPVNQTIT--------------------------
Nasonia_vitripennis_X1             KEAPFQNVSMYESRDACGGDGWKVVDIGVNEEDKIKMKKIDTSISDSNTNNVTNNRSSTY
Microplitis_demolitor              IEAPNRTVTMYDGRDACGGDGWRVDDVASESS----------------------NESSNL
Neodiprion_lecontei                IEAPN-NVTMYDGRDACGGDGWRVDDVSAAESSDRTIHQNG-------------TDSETT
Athalia_rosae                      IEAPN-NVTMFDGRDACGGDGWRVDDVSAVDNPEAPPVLTNGT-----------LTESVS
Orussus_abietinus                  IEAPTQNVTMYDGRDACGGDGWRVDDVSSSSTSTSNQSVINNANKT--------TIETKS
Dufourea_novaeangliae              IEAPNRNVTMYDGRDACGGDGWKVDDVSVNKNYSLMDPSSV-------------LRPEKN
Megachile_rotundata_X1             IEAPNQNVTMYDGRDACGGDGWKVDDVPAGSNTNSNADSPPINGSKSI------GQQKRN
Melipona_quadrifasciata            IEAPHRNMTVYDARDACGGDGWKVDDVSATSNTTET------------------NKFGKK
Apis_mellifera_X1                  IEAPNRNVTMYDGRDACGGDGWKVDDVSASTNSTLMD-----------------SRQEKS
Eufriesea_mexicana_1               IEAPNKNVTMYDGRDACGGDGWKVDDVSASTNTTQVESGGKFGT----------GRQEKN
Eufriesea_mexicana_2               IEAPNKNVTMYDGRDACGGDGWKVDDVSASTNTTQVESGGKFGT----------GRQEKN
Bombus_terrestris                  IEAPNMNVTMYDGRDACGGDGWKVDDVSASTNTTLMEGSKFGS-----------GRQEKN
Bombus_impatiens                   IEAPNMNVTMYDGRDACGGDGWKVDDVSASTNTTLMEGSKFGS-----------GRQEKN
Polistes_dominula_X1               IEAPNENVTMYDGRDACGGDGWRVDDVSAIEGTTKSVTPNYT------------EPEKTT
Cephus_cinctus_X1                  IEAPNRNVTMYDGRDACGGDGWKVDDVGATESSDSSVPLGEE------------DSNHTE
Dinoponera_quadriceps_X2           IEAPKQNVKMYDRRDACGGDGWRVEDVSAGSPIPPSYNGT--------------ILQGPI
Harpegnathos_saltator_X1           IEAPNTNVKMYDGRDACGGDGWRVEDVSAEIPTSPSNNGT--------------IPQRPI
Linepithema_humile_X2              IEAPNRNVTMYDGRDACGGDGWRVEDISADSVMPQSPNS---------------TDRHPE
Linepithema_humile_X1              IEAPNRNVTMYDGRDACGGDGWRVEDISADSVMPQSPNS---------------TDRHPE
Ooceraea_biroi                     IEAPSQNVTMYDGRDACGGDGWRVEDVSAESSTQQSHNGT--------------EIQGPI
Camponotus_floridanus              IEAPYQNVTMYDGRDACGGDGWRVEDVSAESTSQQH------------------NDTQQP
Solenopsis_invicta                 IEAPNQNITMYDGRDACGGDGWRVEDVSADSTTLFANQTQNS------------THTDLQ
Pogonomyrmex_barbatus_X1           IEAPNQNITMYDGRDACGGDGWRVEDVSAESTTPQPQ-----------------NSSEPV
Vollenhovia_emeryi_X1              IEAPNQNVTMYDGRDACGGDGWRVEDVSAETAPSQSNKST--------------ELQETS
Wasmannia_auropunctata             IEAPNQNVTMYDGRDACGGDGWRVEDVSADSTTMQPYNGTL-------------DPQEPV
Cyphomyrmex_costatus               IEAPNQNVTMYDGRDACGGDGWKVEDVSAESTTMQPHNGT--------------ESQEPV
```

```
Trachymyrmex_septentrionalis       IEAPNQNITMYDGRDACGGDGWRVEDVSAESTTPQPHNGT--------------ESQEPV
Atta_colombica                     IEAPNQNITMYDGRDACGGDGWRVEDVSAESTTPQPHNGT--------------ESQEPV
Trachymyrmex_cornetzi              IEAPNQNITMYDGRDACGGDGWRVEDVSAESTTP--HNGT--------------ESQEPV
Acromyrmex_echinatior              IEAPNQNITMYDGRDACGGDGWRVEDVSAESTTPQPHNGT--------------ESQEPV
Atta_cephalotes                    IEAPNQNITMYDGRDACGGDGWRVEDVSAESTTPQPHNGT--------------ESQEPV
                                         . .     : *    * :

Drosophila_melanogaster            VM--VLSNLIPYTNYSYYVRTMAISSELT---NAESDVKNFRTNPGRPSKVTEVVATAI-
Halyomorpha_halys                  GVYHTLTQLEPNTQYAIFVKTYTIDST-----GGQSTTLYERTLPSKPTPPMEFHGASF-
Rhodnius_prolixus                  MYHHTITKLQPDTQYAIFVKTYTVDST-----GGQSPVLYVRTLPSRPSMPLYLLAHSN-
Cimex_lectularius_X1               SIYHIITRLEPYTQYAFYVKTYTVDST-----GIQSPIQYVRTLPSQPKMPAKLEGISN-
C._lectularius_X2                  SIYHIITRLEPYTQYAFYVKTYTVDST-----GIQSPIQYVRTLPSQPKMPAKLEGISN-
Cryptotermes_secundus              RMCHNVTHLKPFTLYAYYVKSYVTASATE---EGRSSIQYFRTYPDRPSIPRRIIAYSN-
Miniopterus_natalensis             HPGWLMRGLKPWTQYAIFVKTLVTFSDERRTYGAKSDIIYVQTDATNPSVPLDPISVSN-
Callithrix_jacchus                 HPGWLMRNLKPWTQYAIFVKTLVTFSDERRTYGAKSDIIYVQTAATNPSVPLDPISVSN-
Heterocephalus_glaber_X1           HPGWLMRGLKPWTQYAIFVKTLVTFSDERRTYGAKSDILYVQTDATNPSVPLDPISVSN-
Dasypus_novemcinctus               QPGWLMRNLKPWTQYAIFVKTLVTFSDERRTYGAKSDIIYVQTEATNPSVPLDPISVSN-
Tupaia_chinensis_X2                QPGWLMRGLKPWTQYAIFVKTLVTFSDERRTYGAKSDIIYVQTDATNPSVPLDPISVSN-
Homo_sapiens_X1                    HPGWLMRGLKPWTQYAIFVKTLVTFSDERRTYGAKSDIIYVQTDATNPSVPLDPISVSN-
Pongo_abelii_2                     HPGWLMRGLKPWTQYAIFVKTLVTFSDERRTYGAKSDIIYVQTDATNPSVPLDPISVSN-
Pan_troglodytes_X2                 HPGWLMRGLKPWTQYAIFVKTLVTFSDERRTYGAKSDIIYVQTDATNPSVPLDPISVSN-
Boleophthalmus_pectinirostris      DPGHLIMPLKPWTQYAIMVKTQLSASDEHQVQGAKSDIIYVRTNATKPSVPLDPRSSAK-
Larimichthys_crocea_X2             EPGHLILPLKPWTQYAIMVKTQLSASDEHQVHGAKSEIIYVRTNATKPSVPLDPISSSN-
Larimichthys_crocea                EPGHLILPLKPWTQYAIMVKTQLSASDEHQVHGAKSEIIYVRTNATKPSVPLDPISSSN-
Lates_calcarifer                   EPGHLILPLKPWTQYAIMVKTQLSASDEHQVHGAKSEIIYVRTNATKPSVPLDPISSSN-
Seriola_lalandi_dorsalis           EPGHLILPLKPWTQYAIMVKTQLSASDEHQIHGAKSEIIYVRTNATKPSVPLDPISSSN-
Seriola_dumerili                   EPGHLILPLKPWTQYAIMVKTQLSASDEHQIHGAKSEIIYVRTNATKPSVPLDPISSSN-
Zootermopsis_nevadensis            NLVQFLPHLEPFTQYAFYVKTYTKSNV-----GGQSTIKYFRTFPDRPSVPKEIHAFSN-
Parasteatoda_tepidariorum          FIIHNITGLKPFTQYALYIRTYTTASGRQ---GAQSSIVYFKTLSNTPSPPVNIKARST-
Onthophagus_nigriventris           KIEATLKDLKAYTQYAFFVKTYTIAQEKN---GGLSALKYFRTNPGQPSSPTFLNLRAN-
Nicrophorus_vespilloides           --THILTHLEPYTQYAFYVRTYTIATEKS---GAQSKIQYVRTMADRPSVPYNIIVKTDK
Tribolium_castaneum                NVTHPLTSLKPYTQYAFYVKTYTIATERR---GAQSMISYFTTLPDTPTAPVGLEVVSN-
Nilaparvata_lugens                 HMSQLITQLKPYTQYAFYVKTYTIASENS---GAQSPIIYFRTEPDIPSMPQNLVAKIE-
Blattella_germanica                ----LLPHLKPYTQYAFYVKTYTIATERT---GARSDIQYFRTLPYAPSPPMGLDVKAQ-
Nasonia_vitripennis_X1             TQYTILTQLKPFTQYAYYVKTYTISTERS---GAQSKLKYFKTLPGQPSIVRALTIYSN-
Microplitis_demolitor              IITALLTQLKPYTQYAFFVKTYTIATERS---GAQSKVQYFTTLPGEPSQPKAFSVWSN-
Neodiprion_lecontei                EYYSLLTQLKPYTQYAFYVKTYTIATERS---GAQSDLHYFTTDPDAPSVPRSLYAYSN-
Athalia_rosae                      YLTHILTQLKPYTQYAFYVKTYTIATERS---GAQSDLHYFTTFPDAPSIPRALSTWSN-
Orussus_abietinus                  IVTHILTLLNPYTQYAFYVKTYTIATERS---GALSKVKYFTTLPGTPSAPRALSTWSN-
Dufourea_novaeangliae              VHTHYLSQLQPYTQYAYYVKTYTIATERS---GAQSKLTYFRTLPEAPSVPRSFKTWST-
Megachile_rotundata_X1             IHAHYLSRLKPYTQYAYYVKTYSIATEKS---GAQSKLTYFRTMPDAPSSPLSLNTWSN-
Melipona_quadrifasciata            LHTHYLSQLKPYTQYAYYVKTYTIATERA---GAQSKVMYFRTMPEAPSQPRSLIIWSN-
Apis_mellifera_X1                  VHTHYLSQLKPYTQYAYYVKTYTIATERS---GAQSNLTYFRTMPEAPSSPRSLLTWSN-
Eufriesea_mexicana_1               VHTHYLSQLKPYTQYAYYVKTYTIATERS---GAQSNLTYFRTMPEAPSAPRSLTTRSN-
Eufriesea_mexicana_2               VHTHYLSQLKPYTQYAYYVKTYTIATERS---GAQSNLTYFRTMPEAPSAPRSLTTRSN-
Bombus_terrestris                  VHTHYLSQLKPYTQYAYYVKTYTIATERS---GAQSNLTYFRTMPEAPSSPRSLMTWSN-
Bombus_impatiens                   VHTHYLSQLKPYTQYAYYVKTYTIATERS---GAQSNLTYFRTMPEAPSSPRSLMTWSN-
Polistes_dominula_X1               YLSHILTQLKPFTQYAFYVKTYTIATERS---GAQSKVTYFTTKPHAPSAPRALTTRSN-
Cephus_cinctus_X1                  YLTHILTQLQPYTQYAYYVKTYTIATERS---GAQSNVSYFTTFPDAPSAPRALSTWSN-
Dinoponera_quadriceps_X2           VLTHILTQLKPYTQYAYYVKTYTIATERS---GAQSMVKYFMTLPDTPSSPRALSTYSN-
Harpegnathos_saltator_X1           VLTHILTQLKPYTQYAYYVKTYTIATERS---GAQSAVSYFVTLPDTPSSPRALSTYSN-
Linepithema_humile_X2              PFSHILTLLKPYTQYAYYVKTYTIATERS---GAQSKLEYFTTLPDAPSPPRALSIWSN-
Linepithema_humile_X1              PFSHILTLLKPYTQYAYYVKTYTIATERS---GAQSKLEYFTTLPDAPSPPRALSIWSN-
Ooceraea_biroi                     YLTHILTLLKPYTQYAYYVKTYTIATERS---GAQSQVKYFTTLPDAPSSPRALLTWSN-
Camponotus_floridanus              YLAHILTLLKPYTQYAYYVKTYTIATEES---GAQSKVMYFTTLPDAPSSPRALSIWSN-
Solenopsis_invicta                 EHLHILTQLKPYTQYAYYVKTYTIATERS---GAQSKITYFTTKPDAPGSPRALSTWSN-
Pogonomyrmex_barbatus_X1           YHTHILTLLKPYTQYAYYVKTYTIATERS---GAQSKVKYFTTLPDAPSPPRALSTWSN-
Vollenhovia_emeryi_X1              LLTHIMTLLKPYTQYAYYVKTYTIATERS---GAQSKVNYFTTMPDAPSPPRGLSTWSN-
Wasmannia_auropunctata             YHTHILTLLKPYTQYAYYVKTYTIATERS---GAQSKVKYFTTLPDAPSSPRALSTWSN-
Cyphomyrmex_costatus               DHTHILTLLKPYTQYAYYVKTYTIATERS---GAQSKVKYFTTLPDAPSSPRVLSTWSN-
Trachymyrmex_septentrionalis       YHTHILTLLKPYTQYAYYVKTYTIATERS---GAQSKVKYFTTLPDAPSSPTALSTWSN-
Atta_colombica                     YHTHILTLLKPYTQYAYYVKTYTIATERS---GAQSKVKYFTTLPDAPSSPRALSTWSN-
Trachymyrmex_cornetzi              YHTHILTLLKPYTQYAYYVKTYTIATERS---GAQSKVKYFTTLPDAPSSPRALSTWSN-
Acromyrmex_echinatior              YHTHILTLLKPYTQYAYYVKTYTIATERS---GAQSKVKYFTTLPDAPSPPRALSTWSN-
Atta_cephalotes                    YHTHILTLLKPYTQYAYYVKTYTIATERS---GAQSKVKYFTTLPDAPSSPRALSTWSN-
                                        :  * . * *:  :.:              *      * .  *

Drosophila_melanogaster            -SDSKINVTWSYLDKPYGVLTRYFIKAKLINRPTRN---NNRDYCTEPLVKAMEND-LPA
Halyomorpha_halys                  -SSDSILLEWEPPQRTNGHITEYIVTG-FSRVEESNML-NDMDFCLESFRDTLTNKKPEV
Rhodnius_prolixus                  -SSSEIVVTWEPPEKPNGKLSHYIVKA-TMHGDDPTYL-EARDYCKYPIKKEETTT--PA
Cimex_lectularius_X1               -SSSEILLHWSPPEKPNGKLVSYVVSG-FIQEYHPDFL-STRNFCKKPYVHIPPTT--PP
```

```
C._lectularius_X2                   -SSSEILLHWSPPEKPNGKLVSYVVSG-FIQEYHPDFL-STRNFCKKPYVHIPPTT--PP
Cryptotermes_secundus               -SSSEIILHWQPPLFPNGEISHYIIVG-WRHFDDAKLL-DQRDYCQLPLEYQKENG--EN
Miniopterus_natalensis              -SSSQIILKWKPPSDPNGNITHYLVFW-ERQAEDSELF--ELDYCLKGLKLP-SRT-WSP
Callithrix_jacchus                  -SSSQIILKWKPPTDPNGNITHYLVFW-QKQVEDSELF--ELDYCLKGLKLP-SRT-WSP
Heterocephalus_glaber_X1            -SSSQIILKWKPPSDPNGNITHYLVYW-QRQEEDSELY--ELDYCLKGLKLP-SRA-WSP
Dasypus_novemcinctus                -SSSQIILKWKPPSDPNGNITHYLVFW-ERQAEDSELF--ELDYCLKGLKLP-SRT-WSP
Tupaia_chinensis_X2                 -SSSQIILKWKPPSDPNGNITHYLVFW-ERQAEDSELY--ELDYCLKGLKLP-SRT-WSP
Homo_sapiens_X1                     -SSSQIILKWKPPSDPNGNITHYLVFW-ERQAEDSELF--ELDYCLKGLKLP-SRT-WSP
Pongo_abelii_2                      -SSSQIILKWKPPSDPNGNITHYLVFW-ERQAEDSELF--ELDYCLKGLKLP-SRT-WSP
Pan_troglodytes_X2                  -SSSQIILKWKPPSDPNGNITHYLVFW-ERQAEDSELF--ELDYCLKGLKLP-SRT-WSP
Boleophthalmus_pectinirostris       -SSSQILLTWKPPNDPNGNITHYLVFC-QRQAEASELY--KFDYCQKGMKLP-TRG--SA
Larimichthys_crocea_X2              -SSSQIILKWKPPNDPNGNVTHYLVFC-QRQPEAIELY--KFDYCQKGMKLP-SRV--PT
Larimichthys_crocea                 -SSSQIILKWKPPNDPNGNVTHYLVFC-QRQPEAIELY--KFDYCQKGMKLP-SRV--PT
Lates_calcarifer                    -SSSQIILKWKPPNDPNGNITHYLVFC-QRQPEASELY--KFDYCQKGMKLP-SRV--PT
Seriola_lalandi_dorsalis            -SSSQIILKWKPPNDPNGNITHYLVFC-QRQPEASELY--KFDYCQKGMKLP-SRV--PT
Seriola_dumerili                    -SSSQIILKWKPPNDPNGNITHYLVFC-QRQPEASELY--KFDYCQKGMKLP-SRV--PT
Zootermopsis_nevadensis             -STSNIILHWQPPEHPNGILTHYLVSG-IWERDDWLFL-EQRNYCHHPLDHTSDHDVLIV
Parasteatoda_tepidariorum           -VHNEIVVSWKPPKNPNGNVTYYIVEG-LYEEDTSYFMYQRRNYCLEPIAYPDYRK----
Onthophagus_nigriventris            -SSSSIEVDWSPPKSPNGNLTHYKVIVTLKETQDE--L-EYSNSC--------SVR-SSQ
Nicrophorus_vespilloides            SSSSSFDLTWKPPLQPNGNVTYYIITGIRRDQKREDIL-RNRNYCNPSMHRETVSK--PE
Tribolium_castaneum                 -SSNSLKLSWRPPKSPNGKLTHYIVSLKRHNSTTPDLD-EMEHFCKSPSSRPKHPI--PS
Nilaparvata_lugens                  -SSSEIKVAWQPPLNPNGNVTHYLITA-KWERDDSEFL-AQRNYCEEPLT--------SI
Blattella_germanica                 -SSSELIIKWLPPQYPNGNVTHYLITG-TLMKDNQQLL-EQRNYCREPPLLPGDAR-KPL
Nasonia_vitripennis_X1              -ASDTLVISWLPPTEANSNLTHYEITG-QMERYDLTFL-RQRNYCIEPMQVL-EYK--PI
Microplitis_demolitor               -SSSELVMSWLPPLRKNGNLTYYKITG-KREVYDPNIL-AKRDYCDKPLQFP-EKK--PI
Neodiprion_lecontei                 -ASNELVITWEPPLHKNGNLTHYRIIG-RWEKDDRAFL-DQRNYCNEPLNFR-ETK--PM
Athalia_rosae                       -ASNELVVTWEPPINKNGNLTHYRIVG-RWEADDRTFL-DQRNYCDEPLLLS-EKK--TI
Orussus_abietinus                   -SSSELVMSWSPPLHKNGNLTHYRIVG-RWEKDDSNFL-EQRDYCNEPMLLP-EKK--PM
Dufourea_novaeangliae               -SSNELRMSWLPPLHKNGNLSHYRIFG-RWEPDDPNFI-DQRNYCDEPILLT-DKK---A
Megachile_rotundata_X1              -SSNELHMSWLPPLHKNGNLTHYRIFG-RWEPDDPNFI-DQRNYCEEPMLLP-DKK---A
Melipona_quadrifasciata             -SSSELILSWLPPLHKNGNLTHYRIFG-RWEPDDPNFI-DQRNYCEEPMLLP-DKK----
Apis_mellifera_X1                   -SSSELIMSWLPPLHNNGNLTHYRIVG-RWEPDDPNFI-DQRNYCEEPMMLT-DKK---P
Eufriesea_mexicana_1                -SSSELIMSWLPPLHKNGNLTYYRIYG-RWERDDANFI-DQRNYCEEPMLLP-DKK---A
Eufriesea_mexicana_2                -SSSELIMSWLPPLHKNGNLTYYRIYG-RWERDDANFI-DQRNYCEEPMLLP-DKK---A
Bombus_terrestris                   -SSSELIMSWLPPLHKNGNLTHYRIFG-RWEPDDPNFI-DQRNYCEEPLMLT-DKK----
Bombus_impatiens                    -SSSELIMSWLPPLHKNGNLTHYRIFG-RWEPDDPNFI-DQRNYCEEPLMLT-DKK----
Polistes_dominula_X1                -SSSEIIISWSPPLKKNGNLTHYRIMG-RWVKDDSSFI-DQRNYCDEPMPLP-EKK--SI
Cephus_cinctus_X1                   -ASSELVMSWFPPLHKNGNLTHYRIVG-RWEPDDPSFL-AQRNYCDEPLLLP-EKK--SL
Dinoponera_quadriceps_X2            -SSSELVMSWLPPLHANGNLTHYRIIG-RWEPDDPNFI-DQRNYCDEPMLLP-EKK--PM
Harpegnathos_saltator_X1            -SSSELVMSWLPPLHKNGKLTHYRIVG-RWEPDDPNFI-DQRNYCDEPMLLP-EKK--PM
Linepithema_humile_X2               -SSSEMVMSWLPPLHKNGNLTEYRIIG-RLEPDDPNFI-DQRDYCEEPLTLP-EKK--PI
Linepithema_humile_X1               -SSSEMVMSWLPPLHKNGNLTEYRIIG-RLEPDDPNFI-DQRDYCEEPLTLP-EKK--PI
Ooceraea_biroi                      -SSSELIMSWFPPLHKNGNLTHYRIVG-RWEPDDPNFI-DQRNYCDEPMLLP-EKK--PI
Camponotus_floridanus               -SSSELVMSWFPPLHKNGNLTHYRIVG-RWEPDDPNFI-DQRNYCDEPMSLP-EKK-LPM
Solenopsis_invicta                  -SSNELVISWFPPVKKNGNLTHYRIVG-RWEPDDQSFI-DQRNYCDEPMPLL-ETK-SPE
Pogonomyrmex_barbatus_X1            -SSSELVISWFPPLHKNGNLTHYRIIG-RWEPDDANFI-DQRNYCDEPMPLP-KPK-LPG
Vollenhovia_emeryi_X1               -SSNELIISWFPPLHKNGNLTHYRIVG-RWEPDDPSFI-DQRNYCDEPMPLP-ETK--SP
Wasmannia_auropunctata              -SSSELIISWIPPLHKNGNLTHYRIVG-RWEPDDPNFI-DQRNYCDEPMLLP-ETK--PD
Cyphomyrmex_costatus                -SSNELVISWFPPLHKNGNLTHYRIVG-RWEPDDPNFI-DQRNYCDEPMPLP-ESK-PPE
Trachymyrmex_septentrionalis        -SSNELIISWYPPLHKNGKLTHYRIVG-RWEPDDPNFI-DQRNYCDEPMPLP-EAK-PPD
Atta_colombica                      -SSNELVISWFPPLHKNGNLTHYRIVG-RWEPDDPNFI-DQRNYCDEPMPLP-EAK-PPE
Trachymyrmex_cornetzi               -SSNELVISWFPPLHKNGNLTHYRIVG-RWEPDDPNFI-DQRNYCDEPMPLP-EAK-PPE
Acromyrmex_echinatior               -SSNELVISWFSPLHKNGNLTHYRIVG-RWEPDDPNFI-DQRNYCDEPMPLP-EAK-PPE
Atta_cephalotes                     -SSNELVISWFPPLHKNGNLTHYRIVG-RWEPDDPNFI-DQRNYCDEPMPLP-EAK-PPE
                                       . : : *       . :  * :                   *

Drosophila_melanogaster             TTPTKKI-------------------SDPLAGD----CKCVEG----SKKTSSQEYDDR-
Halyomorpha_halys                   TTILPEFKTQIGIEKHD--------------------C-CSDE--------EPKKLEHP-
Rhodnius_prolixus                   PRLSTDDCVDKKPEKK----------RPGDV------CESIDP------HLPPKLYDAP-
Cimex_lectularius_X1                PKFMTHVKTKDECVCNVSTIPIEDEFEPPLVTTEL--CETADE------LAKSPPLYEY-
C._lectularius_X2                   PKFMTHVKTKDECVCNVSTIPIEDEFEPPLVTTEL--CETADE------LAKSPPLYEY-
Cryptotermes_secundus               QLLYDESKPTDSCC------------KPVSRET----CISNDD------SSFNVIYTQES
Miniopterus_natalensis              PSELEGSQKHNQSEY-----------EESAGEC----CSCPKT--------DSQILKEL-
Callithrix_jacchus                  PVESEGFQKHNQSEE-----------EDLAGEC----CSCPKT--------DSQIQKEL-
Heterocephalus_glaber_X1            PFESEDAQKHNQSEY-----------EESAGEC----CSCPKT--------DSQILKEL-
Dasypus_novemcinctus                PFESEDAQKHNQSEY-----------EESAGEC----CSCPKT--------DSQILKEL-
Tupaia_chinensis_X2                 PFESEDSQKHNQSEY-----------EDSAGEC----CSCPKT--------DSQILKEL-
Homo_sapiens_X1                     PFESEDSQKHNQSEY-----------EDSAGEC----CSCPKT--------DSQILKEL-
Pongo_abelii_2                      PFESEDSQKHNQSEY-----------EDSAGEC----CSCPKT--------DSQILKEL-
Pan_troglodytes_X2                  PFESEDSQKHNQSEY-----------EDSAGEC----CSCPKT--------DSQILKEL-
Boleophthalmus_pectinirostris       QVDSNEEQKGNLTEE-----------HGPDKQC----CACPKT--------EKELKKEK-
```

```
Larimichthys_crocea_X2            QVDSDEEQKWNQTEE-----------QGPGTRC----CACPKT--------DKEIKKEI-
Larimichthys_crocea               QVDSDEEQKWNQTEE-----------QGPGTRC----CACPKT--------DKEIKKEI-
Lates_calcarifer                  QVDSDEEQKWNQTEE-----------QGQGTRC----CACPKT--------DKELKKEK-
Seriola_lalandi_dorsalis          QVDSDEEQKWNQTEE-----------QGQGTRC----CACPKT--------DKELKKEK-
Seriola_dumerili                  QVDSDEEQKWNQTEE-----------QGQGTRC----CACPKT--------DKELKKEK-
Zootermopsis_nevadensis           PSSTSDTVSSQNCCEKGKSEP-----IVLIEDEDDYNMLCVPK-----ATKKNKIFDVYG
Parasteatoda_tepidariorum         -SIYDDESTQIGYFENVTSSS-----ESTENEC----CSCSKD--------QHRLGDDD-
Onthophagus_nigriventris          TTPKTSDPAKPTDS------------PPSKNDT----CDCTTDMLRES---NALANKEA-
Nicrophorus_vespilloides          TKISTAVKQQNNT------------------------CECRPA-----PPNKPQSIRKQN
Tribolium_castaneum               VSVPPVTSTTTSNDT----------------------CQCLET------KPSTSSINE--
Nilaparvata_lugens                VDVTNQAGKWTLVNDDDVY-------TGGKPDD----CVCMDD------KKVNKKKQQE-
Blattella_germanica               PLDGSADKKEVTNK-----------------------CSCEDK------TDKTTKLREV-
Nasonia_vitripennis_X1            AEIAAEEKKRAEQELE-AS-------KIPEPST----CECKKR---------DPVQSES-
Microplitis_demolitor             TEIAEEERKRAEAEKER---------TKPDVAT----CECADR------E-SDQSIREK-
Neodiprion_lecontei               TTPDEAAHPKPTENKR-PSST-----NGDEIETDDSKCKCIDD------K-D---LREK-
Athalia_rosae                     SVLAEEARKKAQEEKM-VAAA-----KPGLTST----CQCAED------E-TDPLLREK-
Orussus_abietinus                 SVMVEEERKKAEAEKE-LI-------KIPETAT----CECSDS------K-SDPLIREK-
Dufourea_novaeangliae             ISAEEEERKRIEEEKEE---------TIIAEST----CECADR------E-NDQSLREK-
Megachile_rotundata_X1            ISLAEEERKRMEEEKEQS--------VTPETGT----CECADR------E-TDQSLREK-
Melipona_quadrifasciata           -ALAEEERKRMEEEKEQQQQM-----MLQEASN----CDCLDR-------ETDQSLREK-
Apis_mellifera_X1                 ITLAEEERKRMEEEKEQ---------MVPETGT----CECADR------E-TDQSLREK-
Eufriesea_mexicana_1              ISIAEEERKRIEEEKEQ---------MMPETGT----CDCADR------E-TDQSLREK-
Eufriesea_mexicana_2              ISIAEEERKRIEEEKEQ---------MMPETGT----CDCADR------E-TDQSLREK-
Bombus_terrestris                 -ALAEEERKRIEEEKEQ---------MIPETGT----CECPDR-------ETDQSLREK-
Bombus_impatiens                  -ALAEEERKRIEEEKEQ---------MIPETGT----CECPDR-------ETDQSLREK-
Polistes_dominula_X1              SVIAEEERKRAEEE---LV-------KIPDLAS----CDCADR------EL-DQSMREK-
Cephus_cinctus_X1                 AAMAEEERKKAESEME-RA-------RTPERTA----CDCADR-------VPDQSIREK-
Dinoponera_quadriceps_X2          SVMVEEEKKRAEEERDQST-------KLPDVAT----CDCADR------E-TDQSMREK-
Harpegnathos_saltator_X1          SVMVEEERKRAEEERDQST-------KQLEVAS----CDCADR------E-ADQSMREK-
Linepithema_humile_X2             SVMAEEERKRAEEEKE-LA-------KSPETTS----CICADR------EMADQSIREK-
Linepithema_humile_X1             SVMAEEERKRAEEEKE-LA-------KSPETTS----CICADR------EMADQSIREK-
Ooceraea_biroi                    SVIAEEERKRAEEEKE-LA-------KSPDAAS----CPCTDR------EMTDQSMREK-
Camponotus_floridanus             SVIAEEERKRAEEEKE-LV-------KSPETTS----CGCADR------ETTDQSMREK-
Solenopsis_invicta                EVVAEEEKKYFELEKE-FS-------K---TDS----CLCSDR------EVTDQSMLEK-
Pogonomyrmex_barbatus_X1          EVIAEEEKKHTELEKE-IA-------K---SDS----CACSDR------ETTDQSAREK-
Vollenhovia_emeryi_X1             EEVTMEEEKKPETEK-----------EFTKSDS----CSCTDR------EMTDQWMREK-
Wasmannia_auropunctata            DVIAEEEKKHTELEKE-FA-------K---SDS----CVCSDR------ETTDQSTREK-
Cyphomyrmex_costatus              EVIAEEEKKHNELEKE-FE-------K---TDS----CICSDR------ETTDQSMREK-
Trachymyrmex_septentrionalis      EVIAEEEKKHNELEKE-FA-------K---TDS----CSCSDR------ETIDQSMREK-
Atta_colombica                    EVIAEEEKKHNELEKE-FA-------K---TDS----CICSDR------ETTDQSMREK-
Trachymyrmex_cornetzi             EVIAEEEKKHNELEKE-FA-------K---TDS----CICSDR------ETTDQSMREK-
Acromyrmex_echinatior             EVIAEEEKKHNELEKE-FA-------K---TDS----CICSDR------ETTDQSMREK-
Atta_cephalotes                   EVIAEEEKKHNELEK-----------EFAKTDS----CICSDR------ETTDQSMREK-


Drosophila_melanogaster           -KVQA-GMEFENALQNFIFVPNIRKSKNGSSDKSDGAEGAALDSNAIPNGGATNPSRRRR
Halyomorpha_halys                 -ELICHNKNLKEAKVASSFINLK------------------------------EEASCEK
Rhodnius_prolixus                 --------TCEKYMYTLVDSTRL------------------------------TPTAEEH
Cimex_lectularius_X1              ---DGCHKYVYETLEQPPFLKIY---------------------------ENDNIGRYER
C._lectularius_X2                 --DGC-HKYVYETLEQPPFLKIY-------EN--------------------DNIGRYER
Cryptotermes_secundus             VNEED-SCDKENSIYSVLYSPRL--------------------------------YGNGG
Miniopterus_natalensis            -EESSFRKTFEDYLHNVVFVPRP--------------------------------SRKRR
Callithrix_jacchus                -EESSFRKTFEDYLHNVVFIPRRTSPGPGAEN--------------------SRPTRRRR
Heterocephalus_glaber_X1          -EESSFRKTFEDYLHNVVFVPRNPSAGSGAQD--------------------PRPSRKRR
Dasypus_novemcinctus              -EESSFRKTFEDYLHNVVFVPRKTSSGPGAED--------------------SRPSRKRR
Tupaia_chinensis_X2               -EESSFRKTFEDYLHNVVFVPRKTSPGTGAED--------------------TRPSRKRR
Homo_sapiens_X1                   -EESSFRKTFEDYLHNVVFVPRKTSSGTGAED--------------------PRPSRKRR
Pongo_abelii_2                    -EESSFRKTFEDYLHNVVFVPRKTSSGTGAED--------------------PRPSRKRR
Pan_troglodytes_X2                -EESSFRKTFEDYLHNVVFVPRKTSSGTGAED--------------------PRPSRKRR
Boleophthalmus_pectinirostris     -EDSEYRKTFENYLHNEVFEIKR--------------------------------SRMRR
Larimichthys_crocea_X2            -EDSEYRKTFENYLHNEVFEIKR--------------------------------SRQRR
Larimichthys_crocea               -EDSEYRKTFENYLHNEVFEIKR--------------------------------SRQRR
Lates_calcarifer                  -EDSEYRKTFENYLHNEVFEIKR--------------------------------SRQRR
Seriola_lalandi_dorsalis          -EDSEYRKTFENYLHNEVFEIKR--------------------------------SRQRR
Seriola_dumerili                  -EDSEYRKTFENYLHNEVFEIKR--------------------------------SRQRR
Zootermopsis_nevadensis           VEIDPCINYFYSFIHSGIPELKD--------------------------------EQDKL
Parasteatoda_tepidariorum         -EEAESQIHFEDFLQNKVYIRNP------------------------------NSYKVKR
Onthophagus_nigriventris          -ETES-RINFENELQNRVYIKRV-------------------------------APPPQP
Nicrophorus_vespilloides          AEDVSNQMMFEDNLINFVYVKRI------QKF--------------------INKDRSKR
Tribolium_castaneum               -DVEKSRIDFEDELHNAVYVRKP--------N--------------------FSESRKRR
Nilaparvata_lugens                -KEIQFQIQFEDTLQNLVYIKRS-------------------------------SLRDKR
```

```
Blattella_germanica                -EVQA-QIHFENSLHNLLYVKRP------------------------------TQSRRKR
Nasonia_vitripennis_X1             -EVYS-SITFEDALHNTVYVRRQ-------------------------------DNRRRR
Microplitis_demolitor              -EVSS-SIAFEDALHNQVYVKRV-----------------------------GGNNRRKR
Neodiprion_lecontei                -EVSS-SIAFEDALHNQVYVKRP------------------------------SSDRRRR
Athalia_rosae                      -EVSS-SIAFEDALHNQVYIKRP------------------------------TNERRRR
Orussus_abietinus                  -EVSS-SIAFEDALHNQVYVKRI------------------------------SSRRRRD
Dufourea_novaeangliae              -EASS-AIAFEDAVHNQVYVKRR--------------------------------SRRKR
Megachile_rotundata_X1             -EASS-SIAFEDALHNQVYVKRK------------------------------SSSRRKR
Melipona_quadrifasciata            -EASS-AIEFEDALHNQVYIKRR--------------------------------SRKKR
Apis_mellifera_X1                  -EASS-AIDFEDALHNQVYVKRK--------------------------------PRRKR
Eufriesea_mexicana_1               -EASS-AIAFEDALHNQVYVKRK--------------------------------PRRKR
Eufriesea_mexicana_2               -EASS-AIAFEDALHNQVYVKRK--------------------------------PRRKR
Bombus_terrestris                  -EASS-AIEFEDALHNQVYIKRK--------------------------------PKKKR
Bombus_impatiens                   -EASS-AIEFEDALHNQVYIKRK--------------------------------PKKKR
Polistes_dominula_X1               -EVSS-SIAFEDALHNQVYVKRI------------------------------D-ARRRR
Cephus_cinctus_X1                  -EVSS-SIAFEDALHNKVYVKKV------------------------------N-SRRRR
Dinoponera_quadriceps_X2           -EVSS-SIAFENALHNQVYVKRM------------------------------SSRRRRD
Harpegnathos_saltator_X1           -EVSS-SIAFENALHNQVYVKRN------------------------------VNVRRRR
Linepithema_humile_X2              -EVSS-SIAFENALHNQVYVKRM------------------------------MNARRRR
Linepithema_humile_X1              -EVSS-SIAFENALHNQVYVKRM------------------------------MNARRRR
Ooceraea_biroi                     -EVSS-SIAFENALHNQVYVKRM------------------------------T-TRRRR
Camponotus_floridanus              -EVSS-SIAFENALHNQVYVKRM------------------------------NARRRRD
Solenopsis_invicta                 -EVSS-SIAFENALHNQVYIKRA------------------------------QSRRRRH
Pogonomyrmex_barbatus_X1           -EVSS-SIAFENALHNQVYIKRI------------------------------N-IRRRR
Vollenhovia_emeryi_X1              -EVSS-SIAFENALHNQVYIKRI-------------------------------SSRRRR
Wasmannia_auropunctata             -EVSS-SIAFENALHNQVYIKRM------------------------------S-QRRRR
Cyphomyrmex_costatus               -EVSS-SIAFENALHNQVYVKRM------------------------------SQQRRRR
Trachymyrmex_septentrionalis       -EVSS-SIAFENALHNQVYVKRL------------------------------SQQRRRR
Atta_colombica                     -EVSS-SIAFENALHNQVYVKRL------------------------------SQQRRRR
Trachymyrmex_cornetzi              -EVSS-SIAFENALHNQVYVKRL------------------------------SQQRRRR
Acromyrmex_echinatior              -EVSS-SIAFENALHNQVYVKRL------------------------------SQQRRRR
Atta_cephalotes                    -EVSS-SIAFENALHNQVYVKRL------------------------------SQQRRRR
                                              .

Drosophila_melanogaster            DVALEPELDDVEGSVLLRHVRSITDDTDAFFEKDDENTYK--------------------
Halyomorpha_halys                  YFY---SLLENNILKTENQPSKRYVDYKKKFVNDLED-----------------------
Rhodnius_prolixus                  EEP---ADLIR----------------RNIKDEDEDDRLV--------------------
Cimex_lectularius_X1               GIS-----MIE---------------------EDDDLVLN--------------------
C._lectularius_X2                  GIS-----MIE---------------------EDDDLVLN--------------------
Cryptotermes_secundus              GIY---DSVDL-------------------------------------------------
Miniopterus_natalensis             ALG---EEGNV-TTVVPTAPGFPNTSSTIVPTSWEEQ-----------------------
Callithrix_jacchus                 SLD---DTGNM-TEAEPTVAVLPNTSSTMEPMSPEEP-----------------------
Heterocephalus_glaber_X1           SLG---EVNVT--TAVPLVPGFPTLSSVSVPTNAEEH-----------------------
Dasypus_novemcinctus               ALG---DSANV-TVAVPTVPGFPNTSSTSAPTSPEER-----------------------
Tupaia_chinensis_X2                SLG---DVGNV-TVAIPTVPGFPNTSSTSVPTSPEEH-----------------------
Homo_sapiens_X1                    SLG---DVGNV-TVAVPTVAAFPNTSSTSVPTSPEEH-----------------------
Pongo_abelii_2                     SLG---DVGNV-TAAVPTVAAFPNTSSTSVPTSPEEH-----------------------
Pan_troglodytes_X2                 SLG---DVGNV-TVAVPTVAAFPNTSSTSVPTSPEEH-----------------------
Boleophthalmus_pectinirostris      EVV---GIANH-TLPFLTTAMPPTTAPG----EDKE------------------------
Larimichthys_crocea_X2             AVM---SIANR-THPFLTTAPSPPHGTGIPEDEDEE------------------------
Larimichthys_crocea                AVM---SIANR-THPFLTTAPSPPHGTGIPEDEDEE------------------------
Lates_calcarifer                   SVM---GIANR-THPFHTTTPSPPEGTHSPDDEDEE------------------------
Seriola_lalandi_dorsalis           SVM---GIANR-TLPFHTTAPSPPEGTHIPNDEE--------------------------
Seriola_dumerili                   SVM---GIANR-THPFHTTAPSPPEGTHIPNDEE--------------------------
Zootermopsis_nevadensis            TLA---SRFPN-------FNSSGSSQQKVLATFNNHTLIN--------------------
Parasteatoda_tepidariorum          HIM---EFLSD--------GPESSFSSTSPASQSGDEV----------------------
Onthophagus_nigriventris           SMT---------------LSRKKRQMFPSHNDSNVSD-----------------------
Nicrophorus_vespilloides           DLDYNLQFPNN-------SLADDESKNRSFSTQNKTDMQG--------------------
Tribolium_castaneum                DVDS--EQLNA---------------NRVFNETDEAG-----------------------
Nilaparvata_lugens                 EISLRDGKTITENH----FAESDIRMKTVPVSTDLEV-----------------------
Blattella_germanica                DVSMWGDVSHN--SIEPPMPGDDDYPNMAHRYPDSVDPHM--------------------
Nasonia_vitripennis_X1             DVS---YTSVM-LEAVRMKRELTNYQEKDVISHSKKSLQNQSKTNPEQERSLEQQKILNP
Microplitis_demolitor              DIS---TDFKP---------------------NDSDSLKK--------------------
Neodiprion_lecontei                DVS---EIVKT-AKMW--QEQNVSYSYP----EDKDNALT--------------------
Athalia_rosae                      DLS---ETVRS-AMVM--RRD--LHSYP----EDKEN-LS--------------------
Orussus_abietinus                  TRE---SRALN-AV----THESNAHVNS----QDKKN-GM--------------------
Dufourea_novaeangliae              DVA---RVALV-GG----ADRSP-----------DQP-VL--------------------
Megachile_rotundata_X1             ELD---QISYS-SS----KTSKPS----------DQP-IV--------------------
Melipona_quadrifasciata            EIS---EMFVS------------------RKAKALEQLPM--------------------
Apis_mellifera_X1                  DVS---DILF--------RKTKSL----------DQP-VM--------------------
Eufriesea_mexicana_1               DVS---DMSFG-SS----RKTKAA--------DRDQP--M--------------------
```

```
Eufriesea_mexicana_2              DVS---DMSFG-SS----RKTKAA--------DRDQP--M--------------------
Bombus_terrestris                 DVS---EMSFI-------LSRKTKS-------LDRDQPVM--------------------
Bombus_impatiens                  DVS---EMSFI-------LSRKTKS-------LDRDQPVM--------------------
Polistes_dominula_X1              DTS---EILRA-VRM---MEQNITQAEA----LQKKY-PN--------------------
Cephus_cinctus_X1                 DVS---AMLSA-VR----KNPDTYYSYP----EDKDP--N--------------------
Dinoponera_quadriceps_X2          TKG---T-----------LGQSRDFYND----QNEES-SS--------------------
Harpegnathos_saltator_X1          ETL--------------------ERYKNSYDDQDEEN-SN--------------------
Linepithema_humile_X2             DTS---EMLVA-AS----LGQIS---------EEKEK-DA--------------------
Linepithema_humile_X1             DTS---EMLVA-AS----LGQIS---------EEKEK-DA--------------------
Ooceraea_biroi                    DTS---EMLIA-AD----LAKDSSYSYD----QDKES-AN--------------------
Camponotus_floridanus             VKS---EMLIT-AE----LAKDSNY-----NQQDKEG-IN--------------------
Solenopsis_invicta                TDS---EMLIA-AQ----LAKEPTF----KKVQNWES-IS--------------------
Pogonomyrmex_barbatus_X1          HTS---EMLIA-AE----EAKNFTH--NQVKFQDRGI-FN--------------------
Vollenhovia_emeryi_X1             YTN---EVAAS-------QAENSSYDPVKFQARESAN-----------------------
Wasmannia_auropunctata            GAT---EMLNM-VKKESVLNDDFSYVQD----RERES-TN--------------------
Cyphomyrmex_costatus              HIS---EMLIA-AD----LARESTY--E----QDKES-IN--------------------
Trachymyrmex_septentrionalis      HTS---EMLIA-AD----LAKEITY--D----QDRES-TN--------------------
Atta_colombica                    HTS---EMLIA-AD----LAREFTY--D----QDRES-TN--------------------
Trachymyrmex_cornetzi             HTS---EMVIA-AD----LAREFTY--D----QDRES-TN--------------------
Acromyrmex_echinatior             HTS---EMLIA-AD----LAKEFTY--D----QDRES-TN--------------------
Atta_cephalotes                   HTS---EMLIA-AD----LAREFTYD------QDRES-TN--------------------


Drosophila_melanogaster           --DEEDLSSNKQFYEVFA-KELPPNQTHFVFEKLRHFTRYAIFVVACREEIP---SEKLR
Halyomorpha_halys                 --VKKKKP----LFTIYE--IVPGNRTNVTIKNLDHFMLYTFEVKACRQIDP---AEEVV
Rhodnius_prolixus                 --EDLEQFNSDGTYASFT-ARYPHNVTLVTLSNLKHYTAYTVEVIACRERHP---RDSAT
Cimex_lectularius_X1              --EDVEMLGRDGSLVKFH-RIFPAEANVALIGGLRHFSTYTIEVKACRERVE---GELQR
C._lectularius_X2                 --EDVEMLGRDGSLVKFH-RIFPAEANVALIGGLRHFSTYTIEVKACRERVE---GELQR
Cryptotermes_secundus             ------------ADGHFM-QRSEGNTTTVVIKDLHHFSKYTMKVVVCRKNHS---GEVDF
Miniopterus_natalensis            --------------RPFE-KVV--NKESLVISGLRHFTGYRIELQACNQDSP---EER--
Callithrix_jacchus                --------------KPFE-KVV--NRESLVISGLRHFTGYRIELQACNQDTP---EER--
Heterocephalus_glaber_X1          --------------RPFE-KVV--NKESLVISGLRHFTGYRIELQACNQDAP---EER--
Dasypus_novemcinctus              --------------RPFE-KVV--NKESLVISGLRHFTGYRIELQACNQDVP---EER--
Tupaia_chinensis_X2               --------------RPFE-KVV--NKESLVISGLRHFTGYRIELQACNQDVP---EER--
Homo_sapiens_X1                   --------------RPFE-KVV--NKESLVISGLRHFTGYRIELQACNQDTP---EER--
Pongo_abelii_2                    --------------RPFE-KVV--NKESLVISGLRHFTGYRIELQACNQDTP---EER--
Pan_troglodytes_X2                --------------RPFE-KVV--NKESLVISGLRHFTGYRIELQACNQDTP---EER--
Boleophthalmus_pectinirostris     ------------SFESTRTVITVHDKESAVISNLRHFTSYQIEIHACNHPTD---QAR--
Larimichthys_crocea_X2            ------------TFESTKTVVTVHAKESTVISNLRHFTSYQIEIHACNHPTD---PAR--
Larimichthys_crocea               ------------TFESTKTVVTVHAKESTVISNLRHFTSYQIEIHACNHPTD---PAR--
Lates_calcarifer                  ------------TFESTKTVVTVHAKESTVISSLRHFTSYQIEIHACNHPTD---PSR--
Seriola_lalandi_dorsalis          ------------TFESTKSVVTVHAKESTVISNLRHFTSYQIEIHACNHPTD---PAR--
Seriola_dumerili                  ------------TFESTKSVVTVHAKESTVISNLRHFTSYQIEIHACNHPTD---PAR--
Zootermopsis_nevadensis           -----SYG----VYEKFL-KRINANTTQIVLNHMHHFAQYTIKVAACHEPHY---ADLQK
Parasteatoda_tepidariorum         --TQYKNG----NLLHFK-EVVRHNPF-LVVTNLKHFTEYTIEVRACQDKSE---KNI--
Onthophagus_nigriventris          --SYVGNK----TYV----ATYIVTGTKFYITNLQHHTSYEISVQACRKTEP---DKGDF
Nicrophorus_vespilloides          ------------KFIVFSYKVY--GKTELRIDNLKHFTAYEITIEACQNKDD---PTDMS
Tribolium_castaneum               ------------AYISFS-TVV--TGTEFYMPNLRHYTVYEINVQACREKTN---DKLDT
Nilaparvata_lugens                --QRLENE----PQV----KILLQGKTSYVIRGLHHFAQYTISVSACRQRDE---TDK-L
Blattella_germanica               --DMVSQEPIPPKYMDYDFQHVVYGNTQFVINNLSHFTEYTISVEACREIAPDEPANT--
Nasonia_vitripennis_X1            DEDYIEDG----AIVYFK-RKVPANELNFVMRGLRHYGKYNIKVTACRDKKA---NAH--
Microplitis_demolitor             --NETEED----SEQKFE-RLVPSSNLSFVMRNLRHFATYNIELQACREPVP---DDV--
Neodiprion_lecontei               --DDMVNG----SISLFN-RTV-YGQTNYAVKNLRHYAVYTIEVQACREPVP---NES-Q
Athalia_rosae                     --EKVENG----SYIVFN-RTV-YGQTIFSMKNLRHFAVYTIEVQACREPVP---NDT-L
Orussus_abietinus                 --DVIKNG----SYVIFE-RNVSSSSFTFVMKELRHFAAYNIEVLACRERIP---DDA-Q
Dufourea_novaeangliae             --DKMENN----SYIVFE-RNISSTNLSFTMKNLRHFAVYNIEVQVCRHQEM---NDT--
Megachile_rotundata_X1            --DKMENG----TYVVFE-RLVPSTNLSFVMKNLRHFAAYIIEVQACRAQEM---NDT--
Melipona_quadrifasciata           --EKIENG----TSTLFE-HVVPSTNLSFVMKNLRHFAAYNIEVQACRAQDL---NDT--
Apis_mellifera_X1                 --KKV-NG----TYVAFE-ENVPSTNRSFVMKDLRHFAAYNIEVQACRAQEM---NDT--
Eufriesea_mexicana_1              --EKIENG----VYTAFE-QMVPSTNLSFVMKNLHHFAAYNIEVQACRATEM---NDT--
Eufriesea_mexicana_2              --EKIENG----VYTAFE-QMVPSTNLSFVMKNLHHFAAYNIEVQACRATEM---NDT--
Bombus_terrestris                 --EKIENG----TYIVFE-QLVPSMNLSFVMKNLRHFAAYNIEVQACRAQEM---NDT--
Bombus_impatiens                  --EKIENG----TYIVFE-QLVPSMNLSFVMKNLRHFAAYNIEVQACRAQEM---NDT--
Polistes_dominula_X1              --EEMINN----TYITFD-RIVPTPNLSYVVKNLRHFAYYNIEVQACREVVK---NDT--
Cephus_cinctus_X1                 --DKLEDG----IYTTFE-RLVPSTNLSFVMRNLRHFAAYNIEVQACRERTV---NDS--
Dinoponera_quadriceps_X2          --NKIENG----TYVAFE-VLVPSTNLTYVMKNLRHFAAYDIEVQACRERV----NGT-N
Harpegnathos_saltator_X1          --NKIENG----TYIAFE-VLVPSTNLTYVMRNLRHFAVYDIEVQACRERV----NAT-S
Linepithema_humile_X2             --TKVVNG----TVIMFD-KRVPFTNLTLVMRNLRHFAAYNIEIQACRERVG---NET-K
Linepithema_humile_X1             --TKVVNG----TVIMFD-KRVPFTNLTLVMRNLRHFAAYNIEIQACRERVG---NET-K
Ooceraea_biroi                    --DKIENG----TYTVFE-RLVPSTNLTYVMKNLRHFAAYNIEVQACRERVS---NES-K
Camponotus_floridanus             --DKLDNG----TYTVFE-RLIPSTNLSVVMKNLRHFAAYNIEVQACRERVE---NED-K
```

```
Solenopsis_invicta                 --DKMENG----SVLVFE-RIIPSTNLTFVMRNLRHFTAYNIEVQACRELDASELNDT-K
Pogonomyrmex_barbatus_X1           --DKVVND----TYLVFD-RLVPSTSLTFVMRNLRHFAAYNIEVQACRERVA---NETNK
Vollenhovia_emeryi_X1              --NKAENG----TFIVFE-RSVPSTNHSFVMKNLQHFAAYNIEVQACREPVE---NDT--
Wasmannia_auropunctata             --DKIENG----TFIVFE-RLIPSTNHTFVMRNLRHFAAYNIEVQACRERVG---NDT-K
Cyphomyrmex_costatus               --DKVENG----TFTVFE-RLIPSTNLTFVMRNLRHFAAYNIEVQACRERVG---NDT-K
Trachymyrmex_septentrionalis       --DKMENG----TFTVFE-RLIPSTNRTFVMRNLRHFAAYNIEVQACRERVG---NDT-K
Atta_colombica                     --DKVENG----TFTVFE-RLIPSTNLTFVMRNLRHFAAYNIEVQACRERVG---NDT-K
Trachymyrmex_cornetzi              --DKVENG----TFTVFE-RLIPSTNLTFVMRNLRHFAAYNIEVQACRERVG---NDT-K
Acromyrmex_echinatior              --DKVENG----TFTIFE-RLISSTNLTFVMRNLRHFAAYNIEVQACRERVG---NDT-K
Atta_cephalotes                    --DKVENG----TFTVFE-RLIPSTNLTFVMRNLRHFAAYNIEVQACRERVG---NDT-K
                                                                 .  : *.  * . : .*.

Drosophila_melanogaster            DTSFKKSL-------CSDYDTVFQTTKRKKFADIV-MDLKVDLE----HANNTESPVRVR
Halyomorpha_halys                  TDKDR----------CSMSSTVTLRTLRKLDADDV-SWLTTEVN---------NRSVQLK
Rhodnius_prolixus                  T--KR----------CSLNAFTTLRTLPDPKADNIEGGIKESVV---------NRTVTIT
Cimex_lectularius_X1               S--AN----------CSISAFITLKTLSDSKADEIVGGLRVQVE---------NRTAILN
C._lectularius_X2                  S--AN----------CSISAFITLKTLSDSKADEIVGGLRVQVE---------NRTAILN
Cryptotermes_secundus              EWNDKQ---------CSPASLITARTRKLETADHI-DSSSVKVT-------VASGTVRVF
Miniopterus_natalensis             ---------------CSVAAYVSARTMPEAKADDIVGPVTHEIF--------ENNIVHLM
Callithrix_jacchus                 ---------------CSVAAYVSARTMPEAKADDIVSPVTHEIL--------ENNIVHLM
Heterocephalus_glaber_X1           ---------------CSVAAYVSARTMPEAKADDIVGPVTHEIF--------DNNAVHLM
Dasypus_novemcinctus               ---------------CSVSAYVSARTMPEAKADDIIGPVTHEIF--------ENNVVHLM
Tupaia_chinensis_X2                ---------------CSVAVYISARTMPEAKADDIVGPVTHEIF--------ENNVVHLM
Homo_sapiens_X1                    ---------------CSVAAYVSARTMPEAKADDIVGPVTHEIF--------ENNVVHLM
Pongo_abelii_2                     ---------------CSVAAYVSARTMPEAKADDIVGPVTHEIF--------ENNVVHLM
Pan_troglodytes_X2                 ---------------CSVAAYVSARTMPEAKADDIVGPVTHEIF--------ENNVVHLM
Boleophthalmus_pectinirostris      ---------------CSMAAYVSARTLPEDKADDIVTAIGYEVV---------DNTVHIS
Larimichthys_crocea_X2             ---------------CSMAAYVSARTMPEDKADDIASPINYEVT---------ENTVHIT
Larimichthys_crocea                ---------------CSMAAYVSARTMPEDKADDIASPINYEVT---------ENTVHIT
Lates_calcarifer                   ---------------CSMAAYVSARTMPEDKADDIVGPILYEVT---------ENTVHIT
Seriola_lalandi_dorsalis           ---------------CSMAAYVSARTMPEDKADDIVGPISYNVA---------ENTVHIT
Seriola_dumerili                   ---------------CSMAAYVSARTMPEDKADDIVGPISYDVA---------ENTVHIT
Zootermopsis_nevadensis            Y--NTNDITLAEYEWCSPAGLVTVRTKHLAIADTI-DSDHLAAQ-VS---NGTSGTVRLT
Parasteatoda_tepidariorum          ---EP----------CSIKAITSVRTLHLAGADDI-RSSSVH---ILTE-NVTAYSSFIR
Onthophagus_nigriventris           Q--PN----------CSDSTSKEIRTESKHDADNI--ASNISIV------SVTENSITIS
Nicrophorus_vespilloides           Y--------------CSQKALTTARTESKAGADKI---TEVRVA------NKSMSMVTIV
Tribolium_castaneum                E--NP----------CSSKNMKTHRTLKKKGADDI---KQIEVK------NQSLGMVSLT
Nilaparvata_lugens                 IMTNN----------CSTHSIISARTKKLANADVV-SEIWHEV--IENPVNHTQPIVKLY
Blattella_germanica                T--TN----------CSQQSFLTARTLRLPGADNV-LSSKLVVE-VLNE----SRIVKLK
Nasonia_vitripennis_X1             E--LP----------CSSESMKSLQTLRLDHADEI-PSGTLRLQ-KQAN-NDSTTSIKLT
Microplitis_demolitor              S--QR----------CSTKIIQNCRTLPFEHADDI-PTGSLKLEKIHG--NTSRPIVKLQ
Neodiprion_lecontei                S--NY----------CSTKSIKTARTEPLISADDI-PNNTFDLEVITG--NNSRSMVKLS
Athalia_rosae                      N--SC----------CSTKSIKTARTLPIDSADDI-PQNSFKYNVVVG--NNSESTVNLW
Orussus_abietinus                  D--KP----------CSTKSMRTYRTLPLESADDI-PPESFQMN-LLGG-NNSLTTVKLQ
Dufourea_novaeangliae              L--KK----------CSTKIMRTYRTLPLESADNI-PPDSFKMR-ISGE-NNSLTTVTLQ
Megachile_rotundata_X1             Y--KK----------CSTKSMRTYRTLPMKSADNI-PPNTFKMS-ISGE-NNSLTMVTLQ
Melipona_quadrifasciata            Y--RR----------CSTKSMRTYRTLPLDSADNI-PPNTFKIS-ISGE-NNSLTMVTLQ
Apis_mellifera_X1                  Y--KK----------CSTKSMRTFRTMSLDSADNI-PPNTFKMS-ISGE-NNSLTMVTLQ
Eufriesea_mexicana_1               Y--KN----------CSTKSMRTFRTLRLESADNI-PPNSFKIS-ISGE-NNSLTMVTLQ
Eufriesea_mexicana_2               Y--KN----------CSTKSMRTFRTLRLESADNI-PPNSFKIS-ISGE-NNSLTMVTLQ
Bombus_terrestris                  Y--KK----------CSTKSMRTYRTLPLESADNI-PPNTFKMS-ISGE-NNSLTMVTLQ
Bombus_impatiens                   Y--KK----------CSTKSMRTYRTLPLESADNI-PPNTFKMS-ISGE-NNSLTMVTLQ
Polistes_dominula_X1               N--DT----------CSTKSMKTYRTLPLKGADNI-PPNTLKWT-KIKT-NNTFD-IKLT
Cephus_cinctus_X1                  L--NN----------CSTKSMRTYRTLPLESADNI-PANTFKLS-TIGG-NNSLAMVRLQ
Dinoponera_quadriceps_X2           I--KN----------CSTKSMKTYRTLAMESADNI-PPNTFKLS-VSNE-NNSITVVMLF
Harpegnathos_saltator_X1           I--KN----------CSTKSMKTYRTLAMESADNI-PANTFRAT-VSGE-NNSVAVVTLN
Linepithema_humile_X2              K--KN----------CSTKSMKTYRTFAMESADNI-PLNTFRLS-ISGENNNSLTAVTLY
Linepithema_humile_X1              K--KN----------CSTKSMKTYRTFAMESADNI-PLNTFRLS-ISGENNNSLTAVTLY
Ooceraea_biroi                     K--KN----------CSTKSMKTYRTLAMESADNI-PPNTFKLKSISGE-NNSLTMVTLS
Camponotus_floridanus              SKKNN----------CSTKSMKTYRTLAMESADNI-PPNTFKFS-ISGG-NNSLTIVTLY
Solenopsis_invicta                 S--KN----------CSMKSMKTYRTLAMENADNI-PPNTFTLT-KSGE-NNSLTIITLS
Pogonomyrmex_barbatus_X1           S--KN----------CSTKSMKTHRTLAMESADNI-PLNTFDLK-VNGE-NSSLTIITLR
Vollenhovia_emeryi_X1              T--QN----------CSTKSMKTHRTLPMDTADNI-PPDTFKLK-YSGE-NNSLTVITLS
Wasmannia_auropunctata             S--KN----------CSTKSMKTFRTLSMDIADNI-PADTFQLK-TSGE-NNSLPIVTLS
Cyphomyrmex_costatus               S--KN----------CSTKSMKTYRTLSMESADNI-PPNTFELK-TSGE-NNSFTIVTLS
Trachymyrmex_septentrionalis       S--KN----------CSTKSMKTHRTLFMESADNI-PSNTFELK-TSGE-NNSFTIVTLS
Atta_colombica                     S--KN----------CSTKSMKTYRTLSMESADNI-PSNTFELK-TSGE-NNSFTIVTLS
Trachymyrmex_cornetzi              N--NN----------CSTKSMKTYRTLSMESADNI-PSNTFELK-TSGE-NNSFTIVTLS
Acromyrmex_echinatior              S--KN----------CSTKSMKTYRTLSMESADNI-PSNTFELK-TSGE-NNSFTIVTLS
Atta_cephalotes                    S--KN----------CSTKSMKTYRTLSMESADNI-PSNTFELK-TSGE-NNSFTIVTLS
                                                  **        *     ** :                       :
```

```
Drosophila_melanogaster             WTPPVDPNGEIVTYEVAYK-LQKP-DQVEEKKCIPAADF-NQTAGYLIK-LNEGLYSFRV
Halyomorpha_halys                   WDVPSKPNLMIFAFDLEYRRTDIP-NFRPTIDCIKAHDY-RSKGSYFLHGLDIGQYSIRV
Rhodnius_prolixus                   WT-PPLANGVIVAYMLERVREGSGADSKLMVECIPVA---MARGSFELRGLELGSYRIRL
Cimex_lectularius_X1                WV-PPKSNGMTVSYQIELTRTDRD-EMKPMPECIPVQD--AKNGAFMLEDLEFGEYKIRL
C._lectularius_X2                   WV-PPKSNGMTVSYQIELTRTDRD-EMKPMPECIPVQD--AKNGAFMLEDLEFGEYKIRL
Cryptotermes_secundus               WLEPSSPNGIVVTYHVEYRRTDII-NIKPVVVCVTKLQFERGKNWFVLSNLAPGKYALRI
Miniopterus_natalensis              WQEPKEPNGLIVLYEVSYRRYGDE----ELHLCVSRRHF-ALERGCRLRGLPPGNYSVRI
Callithrix_jacchus                  WQEPKEPNGLIVLYEVSYRRYGDE----ELHLCVSRKHF-TLERGCRLRGLSPGNYSVRV
Heterocephalus_glaber_X1            WQEPKEPNGLIVLYEVSYRRYGEE----ELHLCVSRKHY-ALEKGCRLRGLQPGNYSVRI
Dasypus_novemcinctus                WQEPKEPNGLIVLYEVSYRRYGDE----ELHFCVSRKHF-ALERGCRLRGLSPGNYSVRV
Tupaia_chinensis_X2                 WQGPKEPNGLIVLYEVSYRRYGDE----ELHLCVSRKHY-ALERGCRLRGLSPGNYSVRV
Homo_sapiens_X1                     WQEPKEPNGLIVLYEVSYRRYGDE----ELHLCVSRKHF-ALERGCRLRGLSPGNYSVRI
Pongo_abelii_2                      WQEPKEPNGLIVLYEVSYRRYGDE----ELHLCVSRKHF-ALERGCRLRGLSPGNYSVRV
Pan_troglodytes_X2                  WQEPKEPNGLIVLYEVSYRRYGDE----ELHLCVSRKHF-ALERGCRLRGLSPGNYSVRV
Boleophthalmus_pectinirostris       WKEPSAPNGMIILYEVNYKRLG---DSEELHYCVSRNMY-KQTQGAKLRVMHSGNYTVRI
Larimichthys_crocea_X2              WQEPVAPNGMIILYEVNYKRLG---DTEELHYCVSRNMY-KVTRGCKLKVMHPGNYTVRI
Larimichthys_crocea                 WQEPVAPNGMIILYEVNYKRLG---DTEELHYCVSRNMY-KVTRGCKLKVMHPGNYTVRI
Lates_calcarifer                    WQEPVAPNGMIILYEVNYKRLG---DSEELHYCVSRNMF-RETRGCKLKVMHPGNYTVRI
Seriola_lalandi_dorsalis            WLEPVAPNGMIILYEVNYKRLG---DSEELHYCVSRNMY-KVTRGCKLKVMHPGNYTVRI
Seriola_dumerili                    WLEPVAPNGMIILYEVNYKRLG---DSEELHYCVSRNMY-KVTRGCKLKVMHPGNYTVRI
Zootermopsis_nevadensis             WKEPKAPNGIIVAYRTEHHRTDSG-TLNPIVKCLTRQEYLKYGRGYVLNNLPPGNYSFRV
Parasteatoda_tepidariorum           WEDPKDSNGVIVAFTIEYKKVNGE-NHKAKRICVTKDQFHQHG-GYFLSELPPGNYSLRI
Onthophagus_nigriventris            WKPPINPNGRIRHYFIDVSKSGTP----IPERCIDNQDFKNSSYKYTIEPLSPGNYTIRV
Nicrophorus_vespilloides            WDEPLDPNGHILTYQIEYKRLDIE-NSQPILECITALDYLKHNKRYTLK-LNPGNYSLRV
Tribolium_castaneum                 WKEPEAPNGLIYCYTIEYKKLEIE-NSKANEEYITHARFINQSRIYTLKALSPGNYSVRV
Nilaparvata_lugens                  WKEPPAPNGLIVMYQISHWLVFEN-QQHKKTHCISRRQFLEEGNSHTLTNLAPGNYSYQV
Blattella_germanica                 WEEPAQPNGIIVSYQIGYKRVGNA-DYTPTVECITRTQFIQGSQSYTLNKLQPGDYSLRL
Nasonia_vitripennis_X1              WDEPPRPNGVIVTYQIEYKRVDVQ-NLKAEVACTTRDKFNEDGNSYLLQNLATGNYSVRV
Microplitis_demolitor               WKEPLKPNGLIVAYQVEYKRVGMP-NIQPIVVCISRQKFISDNKSCITKDLPAGNYSVIV
Neodiprion_lecontei                 WEEPPNPNGLIVTYQIEYKKIDFQ-NVKAMVICIPWREFVDDGHVYVLNALPPGNYSLRL
Athalia_rosae                       WKEPPDPNGLIVSYQIEYKNIDIQ-NVKSLVVCMTRQEFLQNGQMYALRGLPAGNYSLKL
Orussus_abietinus                   WKEPPQPNGLIVAYQIEYKRVDIK-NIKGTVVCITRRDFVTSENTYLLKDLSPGNYSVKV
Dufourea_novaeangliae               WDEPPQPNGLIVTYQIEYKRVDVQ-NYGPNVVCITRLNFTKEGKSYVLRDLPVGNYSVKI
Megachile_rotundata_X1              WDEPPQPNGLIITYQIEYKRVDIL-TYNPTVVCITRRDFTKEGNSYVLKELPSGNYSVKV
Melipona_quadrifasciata             WDEPPRPNGLIITYQIEYKRVDIQ-NYKPTVVCITRRDFTKAGNSYVLKELPVGNYSVKV
Apis_mellifera_X1                   WDEPPQPNGLIITYQIEYKRVDIQ-NYKPTVVCITRRDFTKAGNSYVLKELPAGNYSVKV
Eufriesea_mexicana_1                WDEPSEPNGLIITYQIEYKRVDIQ-NYKPTVVCTTRLDFAQAGNRYVLKELPAGNYSVKV
Eufriesea_mexicana_2                WDEPSEPNGLIITYQIEYKRVDIQ-NYKPTVVCTTRLDFAQAGNRYVLKELPAGNYSVKV
Bombus_terrestris                   WDEPPQPNGLIITYQIEYKRVDIQ-NYKPTVVCITRRDFTKAGNSYVLKELPAGNYSVKV
Bombus_impatiens                    WDEPPQPNGLIITYQIEYKRVDIQ-NYKPTVVCITRRDFTKAGNSYVLKELPAGNYSVKV
Polistes_dominula_X1                WAEPSEPNGLIVTYQIEYKRVDIL-NIKATVVCITRRDFNASGNVYYLKDLPPGNYSVKV
Cephus_cinctus_X1                   WEEPVKPNGLIVTYQIEYKRVDIQ-NIKATVVCITRRDFVNGGKSYLLKDLPPGNYSIKV
Dinoponera_quadriceps_X2            WDEPVQPNGLIVTYQIEYKRIDIK-NIKPTVVCITRRDFTNAGNSYLLKDLPAGNYSVKV
Harpegnathos_saltator_X1            WEEPLQPNGLIVTYQIEYKRINIK-NIKPTVVCITRRDFTNAGNSYVLKDLPAGNYSIKV
Linepithema_humile_X2               WDEPPQPNGLIVAYQIEYKRVDIK-NIKSMIICVTRREFTKRDNSYILKDLSAGNYSVKV
Linepithema_humile_X1               WDEPPQPNGLIVAYQIEYKRVDIK-NIKSMIICVTRREFTKRDNSYILKDLSAGNYSVKV
Ooceraea_biroi                      WDEPPQPNGLIVTYQIEYKRVDIK-NIQATVVCITRRDFTNAGNSYILKDLPAGNYSVKV
Camponotus_floridanus               WEEPPQPNGLIVTYQIEYKRVDIK-NIKSTVVCITRRDFTKAGNSYVLKDLPAGNYSVKV
Solenopsis_invicta                  WDEPPQPNGLIVTYQIEYKRIDIQ-NIQATVVCITRRDFVKLGNRYTLKELPAGNYSIKV
Pogonomyrmex_barbatus_X1            WEEPPQPNGLIVTYQIEYKRIDIK-NIKATVACITRRDFVNMGNSYVLKDLPAGNYSVKI
Vollenhovia_emeryi_X1               WDEPAQPNGLIVTYQIEYKRVDIK-NIKATVVCITRRDFAEMGNLYVLKELPTGNYSVKV
Wasmannia_auropunctata              WDEPPQPNGLIVTYQIEYKRIDIK-NIQATVVCITRRDFANMGNSYVLKNLPAGNYSVKV
Cyphomyrmex_costatus                WDEPPQPNGLIVTYQIEYKRIDIK-NIQATVVCITRRDFANMGNTYVLKELSSGNYSIKV
Trachymyrmex_septentrionalis        WDEPPQPNGLIVTYQIEYKRIDIK-NIQATVVCITRRDFTNMGNSYVLKELSPGNYSVKV
Atta_colombica                      WDEPPQPNGLIVTYQIEYKRIDIK-NIQATVVCITRRDFTNMGNSYVLKELSPGNYSVKV
Trachymyrmex_cornetzi               WDEPPQPNGLIVTYQIEYKRIDIK-NIQATVVCITRRDFTNMGNSYVLKELSSGNYSVKV
Acromyrmex_echinatior               WDEPPQPNGLIVTYQIEYKRIDIK-NIQATVVCITRRDFTSMGNSYVLKELSPGNYSVKV
Atta_cephalotes                     WDEPPQPNGLIVTYQIEYKRIDIK-NIQATVVCITRRDFTNMGNSYVLKELSPGNYSVKV
                                    *  *  .*     :                                    :  * *   :

Drosophila_melanogaster             RANSIAG--YGDFTEV-------EH--IKVEPPPS----YAKVFFWLLGIGLAFLIVSLF
Halyomorpha_halys                   RAISLAG--LGNFTEY-------KL--VSIYQFSS---KKIILIVVGVAICIIIVAALAA
Rhodnius_prolixus                   RALSLAG--AGEFTEP-------EH--FSISEYSS-----TNIIIITF-FIVITILLIGI
Cimex_lectularius_X1                RSQSLAG--PGKFTAP-------VY--FTISGQFA-K-DMMAVAVLTV-VILLVILVSVT
C._lectularius_X2                   RSQSLAG--PGKFTAP-------VY--FTISGQFA-K-DMMAVAVLTV-VILLVILVSVT
Cryptotermes_secundus               KTTSLAG--ESKYTNI-------AT--FTVNREIQ-TASRRPEIFVLL-SVIIISSILLF
Miniopterus_natalensis              RATSLAG--NGSWTDP-------TY--FYVANYLDVPSNIAKIVIGPL-V-FVFLFSVVI
Callithrix_jacchus                  RATSLAG--NGSWTEP-------TY--FYVSDYLDVPSNIAKIIIGPL-I-FVFLFSVVI
Heterocephalus_glaber_X1            RATSLAG--NGSWTEA-------TY--FYVTNYSDVSSNIAKMIIGPL-I-FVFLFSIVI
Dasypus_novemcinctus                RATSLAG--NGSWTEA-------TY--FYVTDYLDVPPNITKIIIGTL-I-FVFLFSIVI
```

```
Tupaia_chinensis_X2                RATSLAG--NGSWTEA-------TY--FYVTDYYV-PSNIAKIIIGPL--IFVFLFSVVI
Homo_sapiens_X1                    RATSLAG--NGSWTEP-------TY--FYVTDYYV-PSNIAKIIIGPL--IFVFLFSVVI
Pongo_abelii_2                     RATSLAG--NGSWTEP-------TY--FYVTDYLDVPSNIAKIIIGPL-I-FVFLFSVVI
Pan_troglodytes_X2                 RATSLAG--NGSWTEP-------TY--FYVTDYLDVPSNIAKIIIGPL-I-FVFLFSVVI
Boleophthalmus_pectinirostris      RATSLAG--NGSWTEP-------TY--FYVTDSDP-M-SIVKIAVAPV-ICFALLLIVA-
Larimichthys_crocea_X2             RATSLAG--NGSWTEP-------TY--FYVQDASD-PLYIVKIVIGPI-ICFVLLLFVS-
Larimichthys_crocea                RATSLAG--NGSWTEP-------TY--FYVQDASD-PLYIVKIVIGPI-ICFVLLLFVS-
Lates_calcarifer                   RATSLAG--NGSWTEP-------TY--FYVQDPSD-PLYVVKIVIGPV-ICFVLLLFVA-
Seriola_lalandi_dorsalis           RATSLAG--NGSWTEP-------TY--FYVQDASD-PLYVVKIVIGPV-ICFVLLLFVA-
Seriola_dumerili                   RATSLAG--NGSWTEP-------TY--FYVQDASD-PLYVVKIVIGPV-ICFVLLLFVA-
Zootermopsis_nevadensis            RAMSLAG--EGPFTKL-------FH--FTVEELPA---VSSWGSMIGVFVGCLLVLICFI
Parasteatoda_tepidariorum          RATSMAG--HGNWTSL-------VY--FHIVAASE---SSATETILVV-GIILFLCIAIG
Onthophagus_nigriventris           QAISDAN-TGGAFSLP-------VS--VYIDEPSD-----SWNIIAGI-IILVLIVVGI-
Nicrophorus_vespilloides           IGKSQAD--YGEYSKLYSELDDSKYSTFSIDEPNS---GISELAKIAIGLAVVFTFIVV-
Tribolium_castaneum                SATTSGD--YANFSPY-------AY--FYIEERPS---NTYVTLI----VCMLILVIILA
Nilaparvata_lugens                 CATSLAGLANCSQSPL-------RY--FEIQGYSS-V-TSLQIIFATF-MTAGMLILL--
Blattella_germanica                RATSLAG--VGDYTAV-------RY--FFIKEAAT-T-NNMEILIGAL-VGTAVVTTMLC
Nasonia_vitripennis_X1             RATSFAG--NGEYTEY-------KY--FDVPESNY-SMPAWKVILIVL-FSISFILGAFL
Microplitis_demolitor              RATSLAG--SGADSEV-------LY--VYIDEDSA-K-EVFWIVFWSV-SSIVILITSL-
Neodiprion_lecontei                RATSLYG--NGAYTVT-------KH--FYIEELNA---GGSHPYLWPL-LSVFILSVLIL
Athalia_rosae                      RATSLAG--NGAYTAT-------KY--FYIRELNA---GGSHPFLWPV-LSVLGLLILLL
Orussus_abietinus                  RATSLAG--NGAYTNV-------RY--FYIEEYNT-T-GKFWIMFWSI-GFIVIVILPL-
Dufourea_novaeangliae              RATSLAG--NGAYTEV-------KY--FYIQERNI-L-STFWLVFWLL-FCLLVLATSF-
Megachile_rotundata_X1             RATSLAG--YGDYTQV-------KY--FYIEERNT-I-GVFWLIFWLL-LCGTVMFLSF-
Melipona_quadrifasciata            RATSLAG--YGAYTHV-------RY--FYVDERNG-V-STFWLIFWVL-FCAISVIVG--
Apis_mellifera_X1                  RATSLAG--NGAYTHV-------KY--FYIEERNT-M-NTFWIVFWLL-FCAVLAALSL-
Eufriesea_mexicana_1               RATSLAG--YGAYTNV-------KY--FYIEERYT-I-ITFWVVFWLL-FCVFIAIASL-
Eufriesea_mexicana_2               RATSLAG--YGAYTNV-------KY--FYIEERYT-I-ITFWVVFWLL-FCVFIAIASL-
Bombus_terrestris                  RATSLAG--YGAYTHV-------KY--FYIEERNT-L-STFWVIFWLL-LCVFIAAFGF-
Bombus_impatiens                   RATSLAG--YGAYTHV-------KY--FYIEERNT-L-STFWVIFWLL-FCAVIAIVSF-
Polistes_dominula_X1               RATSLAG--NGNYTEP-------SY--FFIEESNI---GLYWVLFWSI-FCILVVILSP-
Cephus_cinctus_X1                  RATSLAG--NGAYTEV-------KY--FYIEEYST-S-GTFWILFWCI-LVLVLAILVC-
Dinoponera_quadriceps_X2           RATSLAG--NGAYTEL-------RY--FYVEEYGT-I-STFWILSLSF-FCIIMIPVLM-
Harpegnathos_saltator_X1           RATSLAG--NGAYSEL-------RI--FYIEEYGT-ISSTSWIL-LVV-FCIIFILVSM-
Linepithema_humile_X2              RATSLAG--NGAYTEV-------KY--FYIPESGT-I-SKFQIILFSI-LGCVLLIPMTW
Linepithema_humile_X1              RATSLAG--NGAYTEV-------KY--FYIPESGT-I-SKFQIILFSI-LGCVLLIPMTW
Ooceraea_biroi                     RATSLAG--YGAYTEV-------KY--FYIREYGG-----YATLLGSI-FGSIAFILVLG
Camponotus_floridanus              RATSLAG--YGAYTEV-------KY--FYIKEYGM-L-STFWIFFWLI-LTLIMLSSIL-
Solenopsis_invicta                 RATSLAG--NGAYTEV-------KY--FSIEESDT-L-SEFWIVICSI-IGVMTIVIIF-
Pogonomyrmex_barbatus_X1           RATSLAG--NGAYTEV-------QY--FYVREHGA-F-STTWIFFWSI-LGVVIIFTFL-
Vollenhovia_emeryi_X1              RATSLAG--NGAYTEI-------KY--FSIPEYGT-F-NTFWVFFWSI-LGIVIMFSSL-
Wasmannia_auropunctata             RATSLAG--NGAYTEV-------KY--FYISEHGT-L-STFWIFFWSI-LSIVIMSSSL-
Cyphomyrmex_costatus               RATSLAG--NGAYTEV-------KY--FYIPEYGT-F-GTFWIFFWSI-LGVVIMFSSL-
Trachymyrmex_septentrionalis       RATSLAG--NGAYTEV-------KY--FYIPEYGT-F-GTFWILFWSI-LGVVIMFSSL-
Atta_colombica                     RATSLAG--NGAYTEV-------KY--FYIPEYGT-F-GTFWILFWSI-LGIVITFSSL-
Trachymyrmex_cornetzi              RATSLAG--NGVYTEV-------KY--FYIPEYGT-F-STFWILFWSI-LGVVIMFSSL-
Acromyrmex_echinatior              RATSLAG--NGAYTEV-------KY--FYIPEYGT-L-GTFWILFWSI-LGVVITFTSL-
Atta_cephalotes                    RATSLAG--NGAYTEV-------KY--FYIPEYGT-F-GTFWILFWSI-LGIVITFSSL-
                                      :  .      :             . :

Drosophila_melanogaster            GYVCYLHKRKVPSNDL--HMNTEVNPFYASM-------Q-YIPDDWEVLRENIIQLAPLG
Halyomorpha_halys                  SVAYFWKRITMRNE----ILIASVNPEYLGL-------P-LVDEEWELPRERIQVIRELK
Rhodnius_prolixus                  VAGFVYYHRRKMNLQEV--LIASVNPEYFGL-------P-TVDEEWELPRDRVRLIRELK
Cimex_lectularius_X1               LAVWYYRKNLMINQP---VLIASVNPEYCGL-------L-HFDEEWELPRERVHIVKELK
C._lectularius_X2                  LAVWYYRKNLMINQP---VLIASVNPEYCGL-------L-HFDEEWELPRERVHIVKELK
Cryptotermes_secundus              CLSYFLWRKMKENAFR--TIVTSPNPEYVSL-------AIYEPDEWEIVRDKIELVRMLS
Miniopterus_natalensis             GSIYLFLRKRQPDGPMG-PLYASSNPEYLSASDVFPCSV-YVPDEWEVPRDKITLLRELG
Callithrix_jacchus                 GSIYIFLRKRQQDGPLG-PLYASSNPEYLSASDVFPCSV-YVPDEWEVPREKITLLRELG
Heterocephalus_glaber_X1           GSIYLFLRKRQPDGPMG-PLYASSNPEYLSASDVFPCSV-YVPDEWEVPREKITLLRELG
Dasypus_novemcinctus               GSIYLFLRKRQPDGPLG-PLYASSNPEYLSASDVFPCSV-YVPDEWEVPREKIALLRELG
Tupaia_chinensis_X2                GSIYLFLRKRQPDGPLG-PLYASSNPEYLSASDVFPCSV-YVPDEWEVPREKITLLRELG
Homo_sapiens_X1                    GSIYLFLRKRQPDGPLG-PLYASSNPEYLSASDVFPCSV-YVPDEWEVSREKITLLRELG
Pongo_abelii_2                     GSIYLFLRKRQPDGPLG-PLYASSNPEYLSASDVFPCSV-YVPDEWEVPREKITLLRELG
Pan_troglodytes_X2                 GSIYLFLRKRQPDGPLG-PLYASSNPEYLSASDVFPCSV-YVPDEWEVPREKITLLRELG
Boleophthalmus_pectinirostris      IGGFVVFRKNQTQGPSG-PIYASSNPEYLSAND-----V-YEEDEWEVPRDKINILRELG
Larimichthys_crocea_X2             VAGFVMFKKNQTQGPSG-PIYASSNPEYLSAND-----V-YEEDEWEVPRDKINILRELG
Larimichthys_crocea                VAGFVMFKKNQTQGPSG-PIYASSNPEYLSAND-----V-YEEDEWEVPRDKINILRELG
Lates_calcarifer                   VAGFVMFKKNQTQGPSG-PIYASSNPEYLSAND-----V-YEEDEWEVPREKINILRELG
Seriola_lalandi_dorsalis           VTGFVMFKKNQTQGPSG-PIYTSSNPEYLSAND-----V-YEEDEWEVAREKINILRELG
Seriola_dumerili                   VTGFVMFKKNQTQGPSG-PIYTSSNPEYLSAND-----V-YEEDEWEVAREKINILRELG
Zootermopsis_nevadensis            VLAMLWWKKRAKERSLM--IIASVNPDYISAGD-----VIYVEDDWEVAREELKLVRELG
```

```
Parasteatoda_tepidariorum           TIYGVYRFKVKQRIPDY-VDYASVNPEYMSSNL-----V-YVADEWEIARDKVKLLDELG
Onthophagus_nigriventris            IVYILFKKRQQEESP---RMNPSINPEYM-----------YVPDDWEIPRKKIELHKELG
Nicrophorus_vespilloides            LACVVYKNVYHKNDN---TLIKTVNPEYVST-------V-YEPDDWEVLREKISLIKELG
Tribolium_castaneum                 LCAFCFYKKKKADKESM-RLIPSVNPEYVPS-------V-YVPDEWEVPRKKIELIRELG
Nilaparvata_lugens                  GFVVFLRNKSNKQTP----LIAECNPEYVST-------V-YVPDDWEVPRSQIELDGELG
Blattella_germanica                 IAVVYLRRKYIPGIPNV-KLIASVNPEYVST-------L-YEPDEWEVPRKKVELLRELG
Nasonia_vitripennis_X1              VAAWFFKRKFMRNVPSM-RLIATVNPEYVST-------V-YIPDEWEVPRKKVHLLKELG
Microplitis_demolitor               TTVYVCKRKLLQNVPSR-RLIATVNPEYVST-------A-YVPDEWEVPRKKIRLSRELG
Neodiprion_lecontei                 GGGYALKRKFMPNSSEM-RLFATVNPEYVST-------V-YVPDDWEVPRKKIELLRELG
Athalia_rosae                       AGGYVLKRKFMPNVPNM-RLIATVNPEYVST-------V-YVPDEWEVPRKKIELLRELG
Orussus_abietinus                   IVFYVYKRKLMRNVPSM-RLIATVNPEYVST-------A-YVPDEWEVPRKKIQLLRELG
Dufourea_novaeangliae               VVYYFLKRKFMANVANA-TLIATVNPEYVSM-------P-YVLDEWEVPREKIVMTKCLG
Megachile_rotundata_X1              GSFYVYKRKYARNASNV-TLIATVNPEYVSP-------P-YVLDEWEVPREKIELLRSLG
Melipona_quadrifasciata             GLAYLCKRRYFSNGPNV-TLIATVNPEYIST-------P-YVLDEWEVPREKIEMLKSLG
Apis_mellifera_X1                   VAFYACKRKFLRNVPNV-TLIATVNPEYVST-------P-YVLDEWEVPREKIEMLRSLG
Eufriesea_mexicana_1                ISFYVCKRKLLRNAPNVSTLIATVNPEYVST-------P-YVLDEWEVPREKIEMLRSLG
Eufriesea_mexicana_2                ISFYVCKRKLLRNAPNVSTLIATVNPEYVST-------P-YVLDEWEVPREKIEMLRSLG
Bombus_terrestris                   VSFYFCKRKFLRNVPNV-TLIATVNPEYVST-------P-YVLDEWEVPREKIEMLKSLG
Bombus_impatiens                    VSFYVCKRKFLRNVPNV-TLIATVNPEYVST-------P-YVLDEWEVPREKIEMLKSLG
Polistes_dominula_X1                AVLYFCKKKYMRNVPSV-RLIATVNPEYVSS-------V-YVPDEWEVPRKKIQVLKELG
Cephus_cinctus_X1                   SVFYVCKKKNKRNVPNI-RLIATVNPEYAST-------A-YVADEWEVPREKIQLIRELG
Dinoponera_quadriceps_X2            GVFHVCRRKLSGNA-SM-RLIATVNPEYVST-------V-YVPDEWEVPRKKIELLRELG
Harpegnathos_saltator_X1            GVFQVCRRKLSRNA-SM-RLIATVNPEYVST-------V-YVPDEWEVPRKKIELLRELG
Linepithema_humile_X2               IAHCIYKKKSMRNVPSM-RLIATVNPEYVST-------S-YVPDEWEVPRKKIQLLRELG
Linepithema_humile_X1               IAHCIYKKKSMRNVPSM-RLIATVNPEYVST-------S-YVPDEWEVPRKKIQLLRELG
Ooceraea_biroi                      VVVCVYKRKSVRNVPSM-RLIATVNPEYVST-------N-YVPDEWEVPRKKIQLLRELG
Camponotus_floridanus               VIFYIFRRRSARNASSV-KIIATVNPEYVST-------G-YVPDEWEVARKKVQLIRELG
Solenopsis_invicta                  FISYVIKKKSMRNVPSM-RLIATVNPEYVST-------S-YVPDEWEVPRKKIQLLRELG
Pogonomyrmex_barbatus_X1            MAFYILKKRSMRNVPSM-RLIATVNPEYVST-------S-YVPDEWEVPRKKIQLLRELG
Vollenhovia_emeryi_X1               TIFYVFKKRSMRNVPSM-RLIATVNPEYVST-------S-YVPDEWEVPRKKIQLLRELG
Wasmannia_auropunctata              AMFYVFRKRAMRNVPSM-RLIATVNPEYVST-------S-YVPDEWEVARKNIQLLKELG
Cyphomyrmex_costatus                TIFYVFKKRSMRNVPSM-RLIATVNPEYVST-------S-YVPDEWEVPRKKIQLLRELG
Trachymyrmex_septentrionalis        TILYVFKKRSMRNVPSM-RLIATVNPEYVST-------S-YVPDEWEVPRKKIQLLRELG
Atta_colombica                      TILYVFKKRSMRNVPSM-RLIATVNPEYVST-------S-YVPDEWEVPRKKIQLLRELG
Trachymyrmex_cornetzi               TILYVFKKRSMRNVPSM-RLIATVNPEYVST-------S-YVPDEWEVPRKKIQLLRELG
Acromyrmex_echinatior               IILYVFKKRSMRNVPSM-RLIATVNPEYVST-------S-YVPDEWEVPRKKIQLLRELG
Atta_cephalotes                     TILYVFKKRSMRNVPSM-RLIATVNPEYVST-------S-YVPDEWEVPRKKIQLLRELG
                                                            ** *               ::**: *. :     *

Drosophila_melanogaster             QGSFGMVYEGILKSFPPNGVDRECAIKTVNENATDRERTNFLSEASVMKEF-DTYHVVRL
Halyomorpha_halys                   RGNFGIVCEGILLP-----EEKRVAVKKVVETASDRDCVEFLNEAAVMKRFTSAYHIVKL
Rhodnius_prolixus                   RGNFGVVCEGILSP----QGTTVAVKMSIDDEPSDRDAMQFLNEAVVMKQFTEAQHIVKL
Cimex_lectularius_X1                RGNFGVVCEGLLVP----EGRVVAVKKAINENCSPHEIAAFLNEAMVMKQFVDGPHIVKL
C._lectularius_X2                   RGNFGVVCEGLLVP----EGRVVAVKKAINENCSPHEIAAFLNEAMVMKQFVDGPHIVKL
Cryptotermes_secundus               KGSFGIVYEGILWP-----ANIRCAVKTVDEKATVRERIEFLTEASVMKSFTGGHHVVRL
Miniopterus_natalensis              QGSFGMVYEGNAKDIIKGEAETRVAVKTVNESASLRERIEFLNEASVMKGF-TCHHVVRL
Callithrix_jacchus                  QGSFGMVYEGNAKDVIKGEAETRVAVKTVNESASLRERIEFLNEASVMKGF-TCHHVVRL
Heterocephalus_glaber_X1            QGSFGMVYEGNAKDIVKGEAETRVAVKTVNEAASLRERIEFLNEASVMKGF-TCHHVVRL
Dasypus_novemcinctus                QGSFGMVYEGNARDIVKGEAETRVAVKTVNESASLRERIEFLNEASVMKGF-TCHHVVRL
Tupaia_chinensis_X2                 QGSFGMVYEGNARDIVKGEAETRVAVKTVNESASLRERIEFLNEASVMKGF-TCHHVVRL
Homo_sapiens_X1                     QGSFGMVYEGNARDIIKGEAETRVAVKTVNESASLRERIEFLNEASVMKGF-TCHHVVRL
Pongo_abelii_2                      QGSFGMVYEGNARDIIKGEAETRVAVKTVNESASLRERIEFLNEASVMKGF-TCHHVVRL
Pan_troglodytes_X2                  QGSFGMVYEGNARDIIKGEAETRVAVKTVNESASLRERIEFLNEASVMKGF-TCHHVVRL
Boleophthalmus_pectinirostris       QGSFGMVYEGIAKDIIKGESETRVAVKTVNESASLRERIEFLNEASVMKAF-SCHHVVRL
Larimichthys_crocea_X2              QGSFGMVYEGIAKDIIKGEGDTHVAVKTVNESASLRERIEFLNEASVMKAF-SCHHVVRL
Larimichthys_crocea                 QGSFGMVYEGIAKDIIKGEGDTHVAVKTVNESASLRERIEFLNEASVMKAF-SCHHVVRL
Lates_calcarifer                    QGSFGMVYEGIAKDIIKGESETHVAVKTVNESASLRERIEFLNEASVMKAF-SCHHVVRL
Seriola_lalandi_dorsalis            QGSFGMVYEGIAKDIIKGEPDTHVAVKTVNESASLRERIEFLNEASVMKAF-SCHHVVRL
Seriola_dumerili                    QGSFGMVYEGIAKDIIKGEPDTHVAVKTVNESASLRERIEFLNEASVMKAF-SCHHVVRL
Zootermopsis_nevadensis             QGSFGMVYEGLLSP-----GDIRCAVKMISENASVREKMEFLNEATMMKSFAGAHHVVNL
Parasteatoda_tepidariorum           QGSFGMVYKGEVRD-MGGKAFQPCAAKTVNESASLRERIEFLQEASVMKAF-NCHHVIKL
Onthophagus_nigriventris            QGSFGMVYEGIVRD-IKGKATIRCAIKTVNEHATNAERLNFLKEASVMKAF-DTAHVIKL
Nicrophorus_vespilloides            QGSFGMVYEGIALD-IKGKAQTRCAIKTVNEHATDRERIEFLNEASVMKAF-DTAYVVRL
Tribolium_castaneum                 QGSFGMVYEGIAQD-VRGKAQIKCAVKTVNEHATNRERLEFLNEASVMKAF-DTAHVVRL
Nilaparvata_lugens                  QGSFGMVYEGIATNVVPGEPRKKCAIKTVNENASLRERVEFLNEASVMKAS-NTHHVVRL
Blattella_germanica                 QGSFGMVYEGIVRDVVEGHPEMRCAIKTVNEHATDRERSEFLNEASVMKQF-NTHHVVRL
Nasonia_vitripennis_X1              NGSFGMVYEGIAKDVVKGKPEMRCAVKTVNENATDRERVEFLNEASVMKGF-NTHHVVRL
Microplitis_demolitor               NGSFGMVYEGVAKDISKLKPEIRCAVKTVNENATDRERIEFLNEASVMKAF-NTHHVVRL
Neodiprion_lecontei                 NGSFGMVYEGVAKDVVKGKPEVKCAVKTVNENATDRERIEFLNEASVMKAF-NTHHVVRL
Athalia_rosae                       NGSFGMVYEGMARDVLKGKPEVKCAVKTVNENATDRERIEFLNEASVMKAF-NTHHVVRL
Orussus_abietinus                   NGSFGMVYEGIAKDVVKGKPEVRCAVKTVNENATDRERIEFLNEASVMKAF-NTHHVVRL
```

```
Dufourea_novaeangliae              KGSFGMVFEGKAKDIVKDKPEVPCAIKTVNKNATDRERIEFLNEASVMKGF-DSHHVVKL
Megachile_rotundata_X1             NGSFGMVYEGIARDIVEGKPEVRCAVKTVNKDATDRERIEFLNEASVMKGF-DAHHVVKL
Melipona_quadrifasciata            TGTFGMVYEGIARDIVKGKPEVRCAVKTVNKDATDRERIEFLNEASVMKGF-DAHHVVKL
Apis_mellifera_X1                  TGTFGMVYEGIAKDIVKGKPEVRCAVKTVNKDATDRERIEFLNEASVMKGF-DAHHVVKL
Eufriesea_mexicana_1               TGTFGMVYEGIAKDIVKGKSEVRCAVKTVNKDATDRERIEFLNEASVMKGF-DAHHVVKL
Eufriesea_mexicana_2               TGTFGMVYEGIAKDIVKGKPEVRCAVKTVNKDATDRERIEFLNEASVMKGF-DAHHVVKL
Bombus_terrestris                  TGTFGMVYEGIAKDIVKGKPEVRCAVKTVNKDATDRERIEFLNEASVMKGF-DAHHVVKL
Bombus_impatiens                   TGTFGMVYEGIAKDIVKGKPEVRCAVKTVNKDATDRERIEFLNEASVMKGF-DAHHVVKL
Polistes_dominula_X1               NGSFGMVYEGLAKDVVKGKPEVRCAVKTVNENATDRERIEFLNEASVMKAF-NTHHVVRL
Cephus_cinctus_X1                  NGSFGMVYEGLAKDVVKGKPEVPCAVKTVNENATDRERIEFLNEASVMKAF-NTHHVVRL
Dinoponera_quadriceps_X2           NGSFGMVYEGVARDVVKGKPEVRCAVKTVNENATDRQRIEFLNEASVMKAF-DTHHVIKL
Harpegnathos_saltator_X1           NGSFGMVYEGVARDVVKGNPEVRCAVKTVNENATDRQRIEFLNEASVMKAF-NTHHVIKL
Linepithema_humile_X2              NGSFGMVYEGIAKD-VKGKSEVQCAVKTVNENATDRERIEFLNEASVMKAF-HTHHVVKL
Linepithema_humile_X1              NGSFGMVYEGIAKD-VKGKSEVQCAVKTVNENATDRERIEFLNEASVMKAF-HTHHVVKL
Ooceraea_biroi                     NGSFGMVYEGFAKAVVRGKPEVRCAVKTVNENATDRERIEFLNEASVMKAF-DTHHVVKL
Camponotus_floridanus              NGSFGMVYEGLAKDVVKDQPEVRCAVKTVNENATDRERIEFLNEASVMKAF-NTHHVVRL
Solenopsis_invicta                 NGSFGMVYEGLAKDVVKGKPEVRCAVKTVNENATDRERIEFLNEASVMKAF-NTHHVVRL
Pogonomyrmex_barbatus_X1           NGSFGMVYEGLAKDVVKGKPEVRCAVKTVNENATDRERIEFLNEASVMKAF-NTHHVVRL
Vollenhovia_emeryi_X1              NGSFGMVYEGLAKDVVKGTPEVRCAVKTVNENATDRERIEFLNEASVMKAF-NTHHVVRL
Wasmannia_auropunctata             NGSFGMVYEGLARDVVKGNPEVRCAVKTVNENATDRERIEFLNEASVMKAF-NTHHVVRL
Cyphomyrmex_costatus               NGSFGMVYEGLAKDVVKGKPEVRCAVKTVNENATDRERIEFLNEASVMKAF-NTHHVVRL
Trachymyrmex_septentrionalis       NGSFGMVYEGLARDVVKGKPEVRCAVKTVNENATDRERIEFLNEASVMKAF-NTHHVVRL
Atta_colombica                     NGSFGMVYEGLARDVVKGKPEVRCAVKTVNENATDRERIEFLNEASVMKAF-NTHHVVRL
Trachymyrmex_cornetzi              NGSFGMVYEGLARDVVKGKPEVRCAVKTVNENATDRERIEFLNEASVMKAF-NTHHVVRL
Acromyrmex_echinatior              NGSFGMVYEGLARDVVKGKPEVRCAVKTVNENATDRERIEFLNEASVMKAF-NTHHVVRL
Atta_cephalotes                    NGSFGMVYEGLARDVVKGKPEVRCAVKTVNENATDRERIEFLNEASVMKAF-NTHHVVRL
                                    *.**:* :*              .   : .  :  :   ** ** :**      :::.*

Drosophila_melanogaster            LGVCSRGQPALVVMELMKKGDLKSYLRAHRPEER---DEAMMTYLNRIGVTGNVQPPTYG
Halyomorpha_halys                  IGIVSKDWPQLVIMEMMDHGDLKTYLRECR----------------------NTKPPLPQ
Rhodnius_prolixus                  IGIVSRDRPFMVVMEMMAKGDLKSYLRECR----------------------NGIPPSPA
Cimex_lectularius_X1               IGVVSRDVPALVVMEMMANGDLKTYLRECR----------------------NKNVPSSQ
C._lectularius_X2                  IGVVSRDVPALVVMEMMANGDLKTYLRECR----------------------NKNVPSSQ
Cryptotermes_secundus              FGVVSRGQPPLVVMELMALGDLKTFLREAR--------NM------------VPSPINLS
Miniopterus_natalensis             LGVVSKGQPTLVVMELMTHGDLKSHLRSLRPDAE---NNP------------GRPPPTLQ
Callithrix_jacchus                 LGVVSKGQPTLVVMELMAHGDLKSYLRSLRPEAE---NNP------------GRPPPTLQ
Heterocephalus_glaber_X1           LGVVSKGQPTLVVMELMAHGDLKSYLRSLRPESE---NNP------------GRPPPTLQ
Dasypus_novemcinctus               LGVVSKGQPTLVVMELMAHGDLKSYLRSLRPEAE---NNP------------GRPPPTLQ
Tupaia_chinensis_X2                LGVVSRGQPTLVVMELMAHGDLKSYLRSLRPEAE---NNP------------GRPPPTLQ
Homo_sapiens_X1                    LGVVSKGQPTLVVMELMAHGDLKSYLRSLRPEAE---NNP------------GRPPPTLQ
Pongo_abelii_2                     LGVVSKGQPTLVVMELMAHGDLKSYLRSLRPEAE---NNP------------GRPPPTLQ
Pan_troglodytes_X2                 LGVVSKGQPTLVVMELMAHGDLKSYLRSLRPEAE---NNP------------GRPPPTLQ
Boleophthalmus_pectinirostris      LGVVSKGQPTLVVMELMTHGDLKSYLRSLRPDAE---NNP------------GRPPPTLK
Larimichthys_crocea_X2             LGVVSKGQPTLVVMELMTHGDLKSYLRSLRPDAE---NNP------------GRPPPTLK
Larimichthys_crocea                LGVVSKGQPTLVVMELMTHGDLKSYLRSLRPDAE---NNP------------GRPPPTLK
Lates_calcarifer                   LGVVSKGQPTLVVMELMTHGDLKSYLRSLRPDAE---NNP------------GRPPPTLK
Seriola_lalandi_dorsalis           LGVVSKGQPTLVVMELMTHGDLKSYLRSLRPDAE---NNP------------GRPPPTLK
Seriola_dumerili                   LGVVSKGQPTLVVMELMTHGDLKSYLRSLRPDAE---NNP------------GRPPPTLK
Zootermopsis_nevadensis            LGVVSREQPALVVMELMAQGDLKTYLRASR--------QP------------PPPPPDLC
Parasteatoda_tepidariorum          MGVVSKGQPTLVIMELMTNGDLKTFLRSRRPD-----ENP------------GQVAPAMP
Onthophagus_nigriventris           LGVVSQGQPTLVVMELMANGDLKSYLRLHRPDSDGYTHGI------------IGQPPSLR
Nicrophorus_vespilloides           LGVVSQGQPTLVVMELMANGDLKSYLRSHRPDVDV--KVK------------SKQPPTLK
Tribolium_castaneum                LGVVSQGQPTLVIMELMANGDLKTYLRSHRPDAEVYDPAT------------AKQPPTLK
Nilaparvata_lugens                 IGVVSQGQPPYVVMELMANGDLKSYLRLHRPDAT---VEP------------HKQPPTLK
Blattella_germanica                LGIVSQGQPVLVVMELMANGDLKTYLRSHRPDVS---DDP------------TKQPPTLK
Nasonia_vitripennis_X1             LGVVSQGQPTLVVMELMVNGDLKTYLRSHRPDVC---ENF------------SRQPPTLN
Microplitis_demolitor              LGVVSRGQPPYVIMELMVNGDLKTYLRSHRPDQC---ENM------------SRQPPTLK
Neodiprion_lecontei                LGVVSVGQPTLVVMELMVNGDLKTYLRSHRPDVC---ENF------------SKQPPTLK
Athalia_rosae                      LGVVSQGQPTLVVMELMVNGDLKTYLRSHRPDVC---ENF------------SKQPPTLK
Orussus_abietinus                  LGVVSQGQPTLVVMELMVNGDLKTYLRSHRPDVC---ENI------------LRQPPTLK
Dufourea_novaeangliae              LGVVTRGQPTLVIMELMGNGDLKTYLRGHRPDAA---DE-------------QHPPPKLE
Megachile_rotundata_X1             LGVVTRGQPTLVIMELMVNGDLKTYLRSHRPDTC---EN-------------ANVPPKLD
Melipona_quadrifasciata            LGVVTRGQPTLVIMELMVNGDLKRYLRSHRPDTC---ENM------------TSVSPKLD
Apis_mellifera_X1                  LGVVTRGQPTLVIMELMVNGDLKRYLRSHRP--C---ENM------------SNAPPKLD
Eufriesea_mexicana_1               LGVVTRGQPTLVIMELMVNGDLKRYLRSHRPDTC---ESL------------TNVSPKLD
Eufriesea_mexicana_2               LGVVTRGQPTLVIMELMVNGDLKRYLRSHRPDTC---ESL------------TNVSPKLD
Bombus_terrestris                  LGVVTRGQPTLVIMELMVNGDLKRYLRSHRPGTC---ENM------------INAPPKLD
Bombus_impatiens                   LGVVTRGQPTLVIMELMVNGDLKRYLRSHRPGTC---ENM------------INAPPKLD
Polistes_dominula_X1               LGVVSQGQPTLVVMELMVNGDLKTYLRSHRPDVC---ENF------------SRQPPTLK
Cephus_cinctus_X1                  LGVVSQGQPTLVVMELMVNGDLKTYLRSHRPDMC---ENF------------SRQPPTLK
Dinoponera_quadriceps_X2           LGVVSQGQPTLVVMELMVNGDLKTYLRSHRPDVC---ENF------------SRQPPTLK
```

```
Harpegnathos_saltator_X1            LGVVSQGQPTLVVMELMVNGDLKTYLRSHRPDVC---ENF------------SRQPPTLK
Linepithema_humile_X2               LGVVSQGQPTLVVMELMVNGDLKTYLRSHRPDVC---EN-------------SKTPPTLR
Linepithema_humile_X1               LGVVSQGQPTLVVMELMVNGDLKTYLRSHRPDVC---EN-------------SKTPPTLR
Ooceraea_biroi                      LGVVSQGQPTLVVMELMVNGDLKTYLRSHRPDVC---ENF------------SRQPPTLK
Camponotus_floridanus               LGVVSQGQPTLVVMELMVNGDLKTYLRSHRPDVC---EN-------------SKHPPTLR
Solenopsis_invicta                  LGVVSQGQPTLVVMELMVNGDLKTYLRSHRPDVC---EN-------------SKQPPTLR
Pogonomyrmex_barbatus_X1            LGVVSQGQPTLVVMELMVNGDLKTYLRSHRPDVC---EN-------------SKQPPTLR
Vollenhovia_emeryi_X1               LGVVSQGQPTLVVMELMVNGDLKTYLRSHRPDVC---EN-------------SKQPPTLR
Wasmannia_auropunctata              LGVVSQGQPTLVVMELMVNGDLKTYLRNHRPDVC---VN-------------SKQPPTLR
Cyphomyrmex_costatus                LGVVSQGQPTLVVMELMVNGDLKTYLRSHRPDVC---EN-------------SKQPPTLR
Trachymyrmex_septentrionalis        LGVVSQGQPTLVVMELMVNGDLKTYLRSHRPDVC---EN-------------SKQPPTLR
Atta_colombica                      LGVVSQGQPTLVVMELMVNGDLKTYLRSHRPDVC---EN-------------SKQPPTLR
Trachymyrmex_cornetzi               LGVVSQGQPTLVVMELMVNGDLKTYLRSHRPDVC---EN-------------SKQPPTLR
Acromyrmex_echinatior               LGVVSQGQPTLVVMELMVNGDLKTYLRSHRPDVC---EN-------------SKQPPTLR
Atta_cephalotes                     LGVVSQGQPTLVVMELMVNGDLKTYLRSHRPDVC---EN-------------SKQPPTLR
                                    :*: :   *  *:**:*  **** .**  *

Drosophila_melanogaster             RIYQMAIEIADGMAYLAAKKFVHRDLAARNCMVADDLTVKIGDFGMTRDIYETDYYRKGT
Halyomorpha_halys                   QMLLMAAQIADGMAYMEVTKHVHRDLAARNCMIREDLVVKIGDFGMARDIYETDYYRKGN
Rhodnius_prolixus                   GMILMAAQIADGMAYLESAKFVHRDLAARNCMVSDKLIVKIGDFGMTRDIYETDYYRKGN
Cimex_lectularius_X1                RMLLMAAQIADGMAYLEAAKFVHRDLAARNCMVSDNGVVKIGDFGMTRDIYETDYYRKGN
C._lectularius_X2                   RMLLMAAQIADGMAYLEAAKFVHRDLAARNCMVSDNGVVKIGDFGMTRDIYETDYYRKGN
Cryptotermes_secundus               RILLMAAQIADGMAYLEASKFVHRDLAARNCMLSENGTVKIGDFGMARDIYETDYYRKGN
Miniopterus_natalensis              EMIQMAAEIADGMAYLNAKKFVHRDLAARNCMVAHDFTVKIGDFGMTRDIYETDYYRKGG
Callithrix_jacchus                  EMIQMAAEIADGMAYLNAKKFVHRDLAARNCMVAHDFTVKIGDFGMTRDIYETDYYRKGG
Heterocephalus_glaber_X1            EMIQMAAEIADGMAYLNAKKFVHRDLAARNCMVAHDFTVKIGDFGMTRDIYETDYYRKGG
Dasypus_novemcinctus                EMIQMAAEIADGMAYLNAKKFVHRDLAARNCMVAHDFTVKIGDFGMTRDIYETDYYRKGG
Tupaia_chinensis_X2                 EMIQMAAEIADGMAYLNAKKFVHRDLAARNCMVAHDFTVKIGDFGMTRDIYETDYYRKGG
Homo_sapiens_X1                     EMIQMAAEIADGMAYLNAKKFVHRDLAARNCMVAHDFTVKIGDFGMTRDIYETDYYRKGG
Pongo_abelii_2                      EMIQMAAEIADGMAYLNAKKFVHRDLAARNCMVAHDFTVKIGDFGMTRDIYETDYYRKGG
Pan_troglodytes_X2                  EMIQMAAEIADGMAYLNAKKFVHRDLAARNCMVAHDFTVKIGDFGMTRDIYETDYYRKGG
Boleophthalmus_pectinirostris       EMVQMAAEIADGMAYLNAKKFVHRDLAARNCMVAHDLTVKIGDFGMTRDIYETDYYRKGG
Larimichthys_crocea_X2              EMIQMAAEIADGMAYLNAKKFVHRDLAARNCMVAHDLTVKIGDFGMTRDIYETDYYRKGG
Larimichthys_crocea                 EMIQMAAEIADGMAYLNAKKFVHRDLAARNCMVAHDLTVKIGDFGMTRDIYETDYYRKGG
Lates_calcarifer                    EMIQMAAEIADGMAYLNAKKFVHRDLAARNCMVAHDLTVKIGDFGMTRDIYETDYYRKGG
Seriola_lalandi_dorsalis            EMIQMAAEIADGMAYLNAKKFVHRDLAARNCMVAHDLTVKIGDFGMTRDIYETDYYRKGG
Seriola_dumerili                    EMIQMAAEIADGMAYLNAKKFVHRDLAARNCMVAHDLTVKIGDFGMTRDIYETDYYRKGG
Zootermopsis_nevadensis             RMLLMAAQIADGMAYLEAHKFVHRDLAARNCMVAEDLTIKIGDFGMTRDIYETDYYRNGS
Parasteatoda_tepidariorum           EICKMAAEIADGMAYLAAKKFVHRDLAARNCMVSTDLTVKIGDFGMTRDVYETDYYRKGG
Onthophagus_nigriventris            RILQMAIEIADGMAYLSAKKFVHRDLAARNCMVAEDLTVKIGDFGMTRDIYETDYYRKGT
Nicrophorus_vespilloides            RIMQMAIEIADGMAYLAAKKFVHRDLAARNCMVAEDLTVKIGDFGMTRDIYETDYYRKGT
Tribolium_castaneum                 QILQMAIEIADGMAYLSAKKFVHRDLAARNCMVAEDLTVKIGDFGMTRDIYETDYYRKGT
Nilaparvata_lugens                  RIRQMAIEIADGMAYLAAKKFVHRDLAARNCMVAEDLTVKIGDFGMTRDIYETDYYRKGT
Blattella_germanica                 RILQMAVEIADGMAYLAGKKYVHRDLAARNCMVAEDLTVKIGDFGMTRDIYETDYYRKGT
Nasonia_vitripennis_X1              RIMRMALEIADGMAYLAAKKFVHRDLAARNCMVSDDLTVKIGDFGMTRDVYETDYYRKGT
Microplitis_demolitor               RIIRMAAEISDGMAYLSAKKFVHRDLAARNCMVAEDLTVKIGDFGMTRDIYETDYYRKGT
Neodiprion_lecontei                 RILQMAIEIADGMSYLSAKKFVHRDLAARNCMVAEDMTVKIGDFGMTRDIYETDYYRKGT
Athalia_rosae                       RILQMAIEIADGMSYLAAKKFVHRDLAARNCMVAEDMTVKIGDFGMTRDIYETDYYRKGT
Orussus_abietinus                   RILQMAIEIADGMAYLAAKKFVHRDLAARNCMVAEDLTVKIGDFGMTRDIYETDYYRKGT
Dufourea_novaeangliae               RIIQMAIEIADGMAYLATKKFVHRDLAARNCMVAKDLTVKVGDFGMTRDIYERDYYRKGS
Megachile_rotundata_X1              RILQMAIEIADGMAYLSTKKFVHRDLAARNCMVAEDLTVKVGDFGMTRDIYETDYYRKGS
Melipona_quadrifasciata             RILQMAIEIADGMAYLSTKKFVHRDLAARNCMVAEDLTVKVGDFGMTRDIYERDYYRKGS
Apis_mellifera_X1                   RILQMAIEIADGMAYLSTKKFVHRDLAARNCMVAEDLTVKVGDFGMTRDIYERDYYRKGS
Eufriesea_mexicana_1                RILQMAIEIADGMAYLSTKKFVHRDLAARNCMVAEDLTVKVGDFGMTRDIYERDYYRKGS
Eufriesea_mexicana_2                RILQMAIEIADGMAYLSTKKFVHRDLAARNCMVAEDLTVKVGDFGMTRDIYERDYYRKGS
Bombus_terrestris                   RILQMAIEIADGMAYLSTKKFVHRDLAARNCMVAEDLTVKVGDFGMTRDIYERDYYRKGS
Bombus_impatiens                    RILQMAIEIADGMAYLSTKKFVHRDLAARNCMVAEDLTVKVGDFGMTRDIYERDYYRKGS
Polistes_dominula_X1                RILQMAIEIADGMAYLAAKKFVHRDLAARNCMVAEDLTVKIGDFGMTRDIYETDYYRKGT
Cephus_cinctus_X1                   RILQMAIEIADGMGYLAAKKFVHRDLAARNCMVAEDLTVKIGDFGMTRDIYETDYYRKGT
Dinoponera_quadriceps_X2            RILQMAIEIADGMAYLAAKKFVHRDLAARNCMVAEDLTVKIGDFGMTRDIYETDYYRKGT
Harpegnathos_saltator_X1            RIVQMAIEIADGMAYLAAKKFVHRDLAARNCMVAEDLTVKIGDFGMTRDIYETDYYRKGT
Linepithema_humile_X2               DILQMAIEIADGMSYLSAKKFVHRDLAARNCMVDDKLTVKIGDFGMTRDIYETDYYRKGS
Linepithema_humile_X1               DILQMAIEIADGMSYLSAKKFVHRDLAARNCMVDDKLTVKIGDFGMTRDIYETDYYRKGS
Ooceraea_biroi                      RILQMATEIADGMSYLSAKKFVHRDLAARNCMVAEDLTVKIGDFGMTRDIYETDYYRKGS
Camponotus_floridanus               DILQMAVEIADGMSYLAAKKFVHRDLAARNCMVAEDLTVKIGDFGMTRDIYETDYYRKGS
Solenopsis_invicta                  EILQMAVEIADGMSYLSAKKFVHRDLAARNCMVAEDLTVKIGDFGMTRDIYETDYYRKGS
Pogonomyrmex_barbatus_X1            DILQMAVEIADGMSYLAAKKFVHRDLAARNCMVAEDLTVKIGDFGMTRDIYETDYYRKGS
Vollenhovia_emeryi_X1               EILQMAVEIADGMSYLSAKKFVHRDLAARNCMVAEDLTVKIGDFGMTRDIYETDYYRKGS
Wasmannia_auropunctata              DILQMAVEIADGMSYLSAKKFVHRDLAARNCMVAEDLTVKIGDFGMTRDIYETDYYRKGS
Cyphomyrmex_costatus                DILQMAVEIADGMSYLAAKKFVHRDLAARNCMVAEDLTVKIGDFGMTRDIYETDYYRKGS
Trachymyrmex_septentrionalis        DILQMAVEIADGMSYLAAKKFVHRDLAARNCMVAEDLTVKIGDFGMTRDIYETDYYRKGS
```

```
Atta_colombica                     DILQMAVEIADGMSYLAAKKFVHRDLAARNCMVAEDLTVKIGDFGMTRDIYETDYYRKGS
Trachymyrmex_cornetzi              DILQMAVEIADGMSYLAAKKFVHRDLAARNCMVAEDLTVKIGDFGMTRDIYETDYYRKGS
Acromyrmex_echinatior              DILQMAVEIADGMSYLAAKKFVHRDLAARNCMVAEDLTVKIGDFGMTRDIYETDYYRKGS
Atta_cephalotes                    DILQMAVEIADGMSYLAAKKFVHRDLAARNCMVAEDLTVKIGDFGMTRDIYETDYYRKGS
                                    :  ** :*:***.*:   *.***********:  .  :*:*****:**:** ****:*

Drosophila_melanogaster            KGLLPVRWMPPESLRDGVYSSASDVFSFGVVLWEMATLAAQPYQGLSNEQVLRYVIDGGV
Halyomorpha_halys                  KGLLPIRWMAPESLNDGVFSSRSDAWSYGIVLWEMATLASQPYQGMSNEQVMAHVVSGNK
Rhodnius_prolixus                  KGLLPIRWMAPESLNDGVFTSKSDAWSYGVVLWEMATLAAQPYQGKSNEEVLQYVISGNK
Cimex_lectularius_X1               KGLLPIRWMAPESLNDGVFTSKSDAWSYGIVLWEMATLASQPYQGLSNEQVLQFVISGNK
C._lectularius_X2                  KGLLPIRWMAPESLNDGVFTSKSDAWSYGIVLWEMATLASQPYQGLSNEQVLQFVISGNK
Cryptotermes_secundus              KGLLPVRWMAPESLADGIFTNQSDVWSYGVVLWEISTLAEQPYQGLANEQVLQFVLRGEL
Miniopterus_natalensis             KGLLPVRWMAPESLKDGVFTTSSDMWSFGVVLWEITSLAEQPYQGLSNEQVLKFVMDGGY
Callithrix_jacchus                 KGLLPVRWMAPESLKDGIFTTSSDMWSFGVVLWEITSLAEQPYQGLSNEQVLKFVMEGGY
Heterocephalus_glaber_X1           KGLLPVRWMAPESLKDGVFTTSSDMWSFGVVLWEITSLAEQPYQGLSNEQVLKFVMDGGY
Dasypus_novemcinctus               KGLLPVRWMAPESLKDGVFTTSSDMWSFGVVLWEITSLAEQPYQGLSNEQVLKFVMDGGY
Tupaia_chinensis_X2                KGLLPVRWMAPESLKDGVFTTSSDMWSFGVVLWEITSLAEQPYQGLSNEQVLKFVMDGGY
Homo_sapiens_X1                    KGLLPVRWMAPESLKDGVFTTSSDMWSFGVVLWEITSLAEQPYQGLSNEQVLKFVMDGGY
Pongo_abelii_2                     KGLLPVRWMAPESLKDGVFTTSSDMWSFGVVLWEITSLAEQPYQGLSNEQVLKFVMDGGY
Pan_troglodytes_X2                 KGLLPVRWMAPESLKDGVFTTSSDMWSFGVVLWEITSLAEQPYQGLSNEQVLKFVMDGGY
Boleophthalmus_pectinirostris      KGLLPVRWMAPESLKDGVFTAHSDCWSFGVVLWEISTLAEQPYQGLSNEQVLKFVMDGGY
Larimichthys_crocea_X2             KGLLPVRWMAPESLKDGVFTAHSDCWSFGVVLWEISTLAEQPYQGLSNEQVLKFVMDGGY
Larimichthys_crocea                KGLLPVRWMAPESLKDGVFTAHSDCWSFGVVLWEISTLAEQPYQGLSNEQVLKFVMDGGY
Lates_calcarifer                   KGLLPVRWMAPESLKDGVFTAHSDCWSFGVVLWEISTLAEQPYQGLSNEQVLKFVMDGGY
Seriola_lalandi_dorsalis           KGLLPVRWMAPESLKDGVFTAHSDCWSFGVVLWEISTLAEQPYQGLSNEQVLKFVMDGGF
Seriola_dumerili                   KGLLPVRWMAPESLKDGVFTAHSDCWSFGVVLWEISTLAEQPYQGLSNEQVLKFVMDGGY
Zootermopsis_nevadensis            KGLLPIRWMSPESLADGVFTSQSDVWSFGVVLWEMATLAEQPYQGLANEQVLQFVLGGGI
Parasteatoda_tepidariorum          KGLLPVRWMSPESLKDGIFDSQTDVWSYGVVLWEMATLASQPYQGLSNEDVLKFVIAGNT
Onthophagus_nigriventris           KGLLPVRWMAPESLKDGVFSSSSDIWSYGVVLWEMATLASQPYQGLSNDQVLRYVIDSGI
Nicrophorus_vespilloides           KGLLPVRWMAPESLKDGVFTSNSDVWSYGVVLWEMATLASQPYQGLSNDQVLRYVIDGGI
Tribolium_castaneum                KGLLPVRWMAPESLKDGVFTSNSDVWSYGVVLWEMATLASQPYQGLSNDQVLRYVIDGGV
Nilaparvata_lugens                 KGLLPVRWMSPESLKDGVFTSSSDVWSYGVVIWEMATLASQPYQGLSNEQVLNYVKTGGC
Blattella_germanica                KGLLPVRWMAPESLKDGVFTSYSDVWSYGVVLWEMATLASQPYQGLSNDQVLRYVIDGGV
Nasonia_vitripennis_X1             KGLLPVRWMAPESLKDGVFTSFSDVWSYGVVLWEMVTLASQPYQGLSNDQVLRYVIDGGV
Microplitis_demolitor              KGLLPVRWMAPESLKDGVFTSYSDVWSYGIVLWEMVTLASQPYQGLSNDQVLRYVIDGGV
Neodiprion_lecontei                KGLLPVRWMAPESLKDGVFTSYSDVWSYGVVLWEMVTLASQPYQGLSNDQVLRYVIDGGV
Athalia_rosae                      KGLLPVRWMAPESLKDGVFTSFSDVWSYGVVLWEMVTLASQPYQGLSNDQVLRYVIDGGV
Orussus_abietinus                  KGLLPVRWMAPESLKDGLFTSYSDVWSYGVVLWEMVTLASQPYQGLSNDQVLRYVIDGGV
Dufourea_novaeangliae              KGLLPVRWMAPESLQDGVFSSSSDVWSYGVVLWEMVTFASQPYQGLSNDQVLRYVIDRGV
Megachile_rotundata_X1             KGFLPVRWMAPESLKDGMFSSRSDVWSYGVVLWEMVTFASQPYQGLSNDQVLRYVIEGGV
Melipona_quadrifasciata            KGLLPVRWMAPESLKDGVFSSYSDVWSYGVVLWEMVTFASQPYQGLSNEQVLRYVIEGGV
Apis_mellifera_X1                  KGLLPVRWMAPESLKDGVFSSYSDVWSYGVVLWEMVTFASQPYQGLSNEQVLRYVIEGGV
Eufriesea_mexicana_1               KGLLPVRWMAPESLKDGVFSSYSDVWSYGVVLWEMVTFASQPYQGLSNEQVLRYVIDGGV
Eufriesea_mexicana_2               KGLLPVRWMAPESLKDGVFSSYSDVWSYGVVLWEMVTFASQPYQGLSNEQVLRYVIDGGV
Bombus_terrestris                  KGLLPVRWMAPESLKDGVFSSYSDVWSYGVVLWEMVTFASQPYQGLSNEQVLRYVIEGGV
Bombus_impatiens                   KGLLPVRWMAPESLKDGVFSSYSDVWSYGVVLWEMVTFASQPYQGLSNEQVLRYVIEGGV
Polistes_dominula_X1               KGLLPVRWMAPESLKDGVFTSFSDVWSYGVVLWEMVTLASQPYQGLSNDQVLRYVIEGGV
Cephus_cinctus_X1                  KGLLPVRWMAPESLKDGVFTSFSDVWSYGVVLWEMVTLASQPYQGLSNDQVLRYVIDGGV
Dinoponera_quadriceps_X2           KGLLPVRWMAPESLKDGVFTSFSDVWSYGVVLWEMVTLASQPYQGLSNDQVLRYVIEGGV
Harpegnathos_saltator_X1           KGLLPVRWMAPESLKDGVFTSFSDVWSYGVVLWEMVTLASQPYQGLSNDQVLRYVIEGGV
Linepithema_humile_X2              KGLLPVRWMAPESLKDGVFTSFSDVWSYGVVLWEMVTLASQPYQGLSNDQVLRYVIEGGV
Linepithema_humile_X1              KGLLPVRWMAPESLKDGVFTSFSDVWSYGVVLWEMVTLASQPYQGLSNDQVLRYVIEGGV
Ooceraea_biroi                     KGLLPVRWMAPESLKDGVFTSFSDVWSYGVVLWEMVTLASQPYQGLSNDQVLRYVIEGGV
Camponotus_floridanus              KGLLPVRWMAPESLKDGVFTSFSDVWSYGVVLWEMVTLASQPYQGLSNDQVLRYVIEGGV
Solenopsis_invicta                 KGLLPVRWMAPESLKDGVFTSFSDVWSYGVVLWEMVTLASQPYQGLSNDQVLRYVIEGGV
Pogonomyrmex_barbatus_X1           KGLLPVRWMAPESLKDGVFTSFSDVWSYGVVLWEMVTLASQPYQGLSNDQVLRYVIEGGV
Vollenhovia_emeryi_X1              KGLLPVRWMAPESLKDGVFTSFSDVWSYGVVLWEMVTLASQPYQGLSNDQVLRYVIEGGV
Wasmannia_auropunctata             KGLLPVRWMAPESLKDGVFTSFSDVWSYGVVLWEMVTLASQPYQGLSNDQVLRYVIEGGV
Cyphomyrmex_costatus               KGLLPVRWMAPESLKDGVFTSFSDVWSYGVVLWEMVTLASQPYQGLSNDQVLRYVIEGGV
Trachymyrmex_septentrionalis       KGLLPVRWMAPESLKDGVFTSFSDVWSYGVVLWEMVTLASQPYQGLSNDQVLRYVIEGGV
Atta_colombica                     KGLLPVRWMAPESLKDGVFTSFSDVWSYGVVLWEMVTLASQPYQGLSNDQVLRYVIEGGV
Trachymyrmex_cornetzi              KGLLPVRWMAPESLKDGVFTSFSDVWSYGVVLWEMVTLASQPYQGLSNDQVLRYVIEGGV
Acromyrmex_echinatior              KGLLPVRWMAPESLKDGVFTSFSDVWSYGVVLWEMVTLASQPYQGLSNDQVLRYVIEGGV
Atta_cephalotes                    KGLLPVRWMAPESLKDGVFTSFSDVWSYGVVLWEMVTLASQPYQGLSNDQVLRYVIEGGV
                                   **:**:***.**** **::   :* :*:*:*:**: ::* ***** :*::*: .*

Drosophila_melanogaster            MERPENCPDFLHKLMQRCWHHRSSARPSFLDIIAYLEPQCPN-SQFKEVS-FYHSEAG--
Halyomorpha_halys                  LDLPPIYPKPFKTLMLWCWKWKPKFRPSFLQILDELHEHTT--KGFREVS-YYDSPEG--
Rhodnius_prolixus                  LELPPVYPRPFKTIMAWCWRWKPKFRPCFFQILSELEEHLT--VSFRTVC-FY-------
Cimex_lectularius_X1               LELPPVYPKPFKSLTAWCWRWKPKFRPSFIEILRELEEYMT--VSFRQVS-FFNSEAG--
C._lectularius_X2                  LELPPVYPKPFKSLTAWCWRWKPKFRPSFIEILRELEEYMT--VSFRQVS-FFNSEAG--
```

```
Cryptotermes_secundus              LEKPDSCPDILYRLMIACWQKRPINRPTFLRLVGIIESVTEVGDEFRQSS-FYHSQTG--
Miniopterus_natalensis             LEQPENSPERLNELMRMCWHFNPKMRPTFLEIVDLLKDDLH--PSFPEVS-FFYSDEN--
Callithrix_jacchus                 LDQPDNCPERVTDLMRMCWQFNPKMRPTFLEIVNLLKDDLH--PSFPEVS-FFHSEEN--
Heterocephalus_glaber_X1           LDQPDNCPERVTDLMRMCWQFNPKMRPTFLEIVSLLKDDLH--PSFPEVS-FFHSEEN--
Dasypus_novemcinctus               LDQPDNCPERVTDLMRMCWQFNPKLRPTFLEIVDLLKDDLH--PSFPEVS-FFHSEEN--
Tupaia_chinensis_X2                LDQPDNCPERVTDLMRMCWQFNPKMRPTFLEIVNLLKDDLH--PSFPEVS-FFHSDEN--
Homo_sapiens_X1                    LDQPDNCPERVTDLMRMCWQFNPKMRPTFLEIVNLLKDDLH--PSFPEVS-FFHSEEN--
Pongo_abelii_2                     LDQPDNCPERVTDLMRMCWQFNPKMRPTFLEIVNLLKDDLH--PSFPEVS-FFHSEEN--
Pan_troglodytes_X2                 LDQPDNCPERVTDLMRMCWQFNPKMRPTFLEIVNLLKDDLH--PSFPEVS-FFHSEEN--
Boleophthalmus_pectinirostris      LDRPDNCADRLHNLMQMCWQYNPKMRPMFQEIIEMLREDLH--PSFQDVS-FFYSEEN--
Larimichthys_crocea_X2             LDRPDNCADRLHNLMQMCWQYNPKMRPTFQEIIEMLREDLH--PTFQEVS-FFYSEEN--
Larimichthys_crocea                LDRPDNCADRLHNLMQMCWQYNPKMRPTFQEIIEMLREDLH--PTFQEVS-FFYSEEN--
Lates_calcarifer                   LDRPDNCADRLHNLMQMCWQYNPKMRPTFQEIIEMLREDLH--PTFQEVS-FFYSEEN--
Seriola_lalandi_dorsalis           LDRPDNCADRLHNLMQMCWQYNPKMRPTFQEIIEMLREDLH--PSFQEVS-FFYSEEN--
Seriola_dumerili                   LDRPDNCADRLHNLMQMCWQYNPKMRPTFQEIIEMLREDLH--PSFQEVS-FFYSEEN--
Zootermopsis_nevadensis            LDRPLCCPDVLHQFMVACWQRRPTHRPKFLQIVSDLETVSDLGQKFQHVS-FYHSTEGQV
Parasteatoda_tepidariorum          MDRPEDCPDLLFEVMTMCWNKNPKARPTFLELVEMFLPHVE--NIVQDVFHYFMYKAK--
Onthophagus_nigriventris           MERPENCPDKLYHLMRQCWKYKYAERPSFLDLVSMLLEDSS--PQFSRVS-FYHSAAG--
Nicrophorus_vespilloides           MERPENCPDKLYNLMRFCWKHKPSDRPTFINLCKMLLDDSS--QTFSQIS-FYHSAAG--
Tribolium_castaneum                MERPENCPDKLYTLMRYCWQHKPSARPSFLKLCSLLLEDAS--TSFAQVS-FYHSAAG--
Nilaparvata_lugens                 MERPDNCPDVLYNLMRQCWQHKPADRPTFMQLVRCLLPFAH--PSFASLS-FYHSDEG--
Blattella_germanica                MERPENCPDRLYQLMRLCWQHKPKARPPFMELVSLLLPDVS--PNFVQVS-FYHSNEG--
Nasonia_vitripennis_X1             MERPENCPDILYGMMRQTWCHKATRRPTFIDIERELMLEVDT-TEFEKVS-FYHSPDG--
Microplitis_demolitor              MERPENCPDSLYRLMRGTWNQKAVRRPTFIHITSILLKEASL-EGFEAVS-FYHSPEG--
Neodiprion_lecontei                MERPENCPDSLYNLMRRTWNHKPNQRPTFIDIATMLLTEVNV-ETFERVS-FYHSPEG--
Athalia_rosae                      MERPENCPDSLYTLMRRTWNHKPNQRPTFLDIATMLLSEVNV-ESFERVS-FYHSPEG--
Orussus_abietinus                  MERPENCPDSLYTLMRRTWNHKASKRPTFIDIATMLLQEINV-APFERVS-FYHSPDG--
Dufourea_novaeangliae              MERPENCPELLFDVMKRTWKYSASRRPSFLDIVTTLLNHIDV-ETFQKVS-FYHSKEG--
Megachile_rotundata_X1             MERPENCPELLYELMKRTWRHKAAERPTFMDIATMLLKHIDS-DSFKLVS-FYHSSKG--
Melipona_quadrifasciata            MERPENCPEMLYDLMKRTWKHKVTRRPTFMDIVTMLLKHIDS-KRFQEVS-FYHSAEG--
Apis_mellifera_X1                  MERPENCPELLYELMKRTWRHKATRRPTFMDIASMLLKHIDS-DSFQEVS-FYHSPEG--
Eufriesea_mexicana_1               MERPENCPELLYELMKRTWRHKATRRPTFMDIATMLLKHIDS-ESFQDVS-FYHSLEG--
Eufriesea_mexicana_2               MERPENCPELLYELMKRTWRHKATRRPTFMDIATMLLKHIDS-ESFQDVS-FYHSLEG--
Bombus_terrestris                  MERPENCPELLYELMKHTWRHKATRRPTFMDIASMLLKHIDS-DRFQNVS-FYHSVEG--
Bombus_impatiens                   MERPENCPELLYELMKHTWRHKATRRPTFMDIASMLLKHIDS-DRFQNVS-FYHSVEG--
Polistes_dominula_X1               MERPENCPDSLYNLMRRTWCHKATRRPTFIDIATILLPDVNL-EDFERVS-FYHSSEG--
Cephus_cinctus_X1                  MERPENCPDSLYKLMRQTWNHKATKRPTFIDIATMLLSEVNV-EAFERVS-FYHSSEG--
Dinoponera_quadriceps_X2           MERPENCPDSLYNLMRRTWSHKATRRPTFIDIATMLLQEVNI-EAFESVS-FYHSSAG--
Harpegnathos_saltator_X1           MERPENCPDSLYNLMRRTWSHKATRRPTFIDIATMLLQEVSI-EAFERVS-FYHSPEG--
Linepithema_humile_X2              MERPENCPDTLYNLMWRTWNHRSSKRPTFIDIETQLLQEMNI-ENFEKIS-FFHSPEG--
Linepithema_humile_X1              MERPENCPDTLYNLMWRTWNHRSSKRPTFIDIETQLLQEMNI-ENFEKIS-FFHSPEG--
Ooceraea_biroi                     MERPENCPDSLYNLMRRTWNHRAVRRPTFICIETMLLQEIDI-EDFEKVS-FYHSAEG--
Camponotus_floridanus              MERPENCPDSLYSLMRRTWNHRATRRPTFIDIETLLLQEVNI-EDFENVS-FYHSSEG--
Solenopsis_invicta                 MERPENCPDSLYNLMRRTWNHRATRRPTFIDIETLLLQEVSI-EGFENVS-FYHSPEG--
Pogonomyrmex_barbatus_X1           MERPENCPDSLYNLMRRTWNHRATRRPTFIDIETLLLQEVSI-EGFENVS-FYHSPEG--
Vollenhovia_emeryi_X1              MERPENCPDSLYNLMRRTWNHRATRRPTFIDIETLLLQEVSI-KDFENVS-FYHSLEG--
Wasmannia_auropunctata             MERPENCPDSLYNLMRRTWNHRATRRPTFIDIETLLMQEVSI-EDFENVS-FYHSSEG--
Cyphomyrmex_costatus               MERPENCPDSLYNLMRRTWNHRATRRPTFIDIETQLLQEVSI-EGFENVS-FYHSPEG--
Trachymyrmex_septentrionalis       MERPENCPDSLYNLMRRTWNHRATRRPTFIDIETQLLQEVSI-EDFENVS-FYHSPEG--
Atta_colombica                     MERPENCPDSLYNLMRRTWNHRATRRPTFIDIETQLLQEVSI-EDFENVS-FYHSPEG--
Trachymyrmex_cornetzi              MERPENCPDSLYNLMRRTWNHRATRRPTFIDIETQLLQEVSI-EDFENVS-FYHSPEG--
Acromyrmex_echinatior              MERPENCPDSLYNLMRRTWNHRATRRPTFIDIETQLLQEVNI-EDFENVS-FYHSPEG--
Atta_cephalotes                    MERPENCPDSLYNLMRRTWNHRATRRPTFIDIETQLLQEVSI-EDFENVS-FYHSPEG--
                                   :: *   .  .  .    *      ** *  :   :         .     ::

Drosophila_melanogaster            -----LQHREKERKERHQLDAFAAVPLDQDLQDREQQEDATTPLRMGDYEQNSSLDQPPE
Halyomorpha_halys                  ------------------------------------------------------REAREA
Rhodnius_prolixus                  ------------------------------------------------------------
Cimex_lectularius_X1               -----LQASRG-------------------------------------------AHSSET
C._lectularius_X2                  -----LQASRG-------------------------------------------AHSSET
Cryptotermes_secundus              --------HELRGTWYQQRTRSF-----------FEED----------------------
Miniopterus_natalensis             --------------KAPESEE-------------LEME----------------FEDMES
Callithrix_jacchus                 --------------KAPESEE-------------LEME----------------FEDMEN
Heterocephalus_glaber_X1           --------------KAPESEE-------------LEME----------------FEDMEN
Dasypus_novemcinctus               --------------KAPESDE-------------LEME----------------FEDMEN
Tupaia_chinensis_X2                --------------KAPESEE-------------LEME----------------FEDMES
Homo_sapiens_X1                    --------------KAPESEE-------------LEME----------------FEDMEN
Pongo_abelii_2                     --------------KAPESEE-------------LEME----------------FEDMEN
Pan_troglodytes_X2                 --------------KAPESEE-------------LEME----------------FEDMEN
Boleophthalmus_pectinirostris      --------------KPPETED-------------FDLD----------------MENMES
Larimichthys_crocea_X2             --------------KPPETED-------------FDLD----------------MENMES
```

```
Larimichthys_crocea               --------------KPPETED-------------FDLD----------------MENMES
Lates_calcarifer                  --------------KPPESED-------------FDLD----------------MENMES
Seriola_lalandi_dorsalis          --------------KPPESED-------------FDLD----------------MENMES
Seriola_dumerili                  --------------KPPESED-------------FDLD----------------MENMES
Zootermopsis_nevadensis           IKTNMAQQQDLDLSCELQFDQTFVSSPPVSFYQLLEEETI-----------P--SEENVV
Parasteatoda_tepidariorum         -----TENDDSVTPNTPLRSS-------------MREE----------------------
Onthophagus_nigriventris          -----MEARSTRNNQMI-------------------------------------IQDDVM
Nicrophorus_vespilloides          -----MEARNFRSTLTP-------------------------------------CQDDAR
Tribolium_castaneum               -----IEARSSRPTPSP-------------------------------------SQDDPS
Nilaparvata_lugens                ------GQERRASTSTPQ------------------------------------ATVEEE
Blattella_germanica               ----------REQRVPPRAE----------------------------------LTDDPS
Nasonia_vitripennis_X1            -----VEVRNQNPPSQYRTDKEL---EMV-MQE-SRED-------------E--AEGGED
Microplitis_demolitor             -----IEARNQNTSLSPLNDNDF---EMAPLED-LRED-------------E--QEGDED
Neodiprion_lecontei               -----IEARNQNASHSPQTDKDL---EMETLRN-LQDEEA-----------D--QEGSEE
Athalia_rosae                     -----IEARHQNASHSPQADKDM---EMATLHD-LREDDV-----------DGEGEGSEE
Orussus_abietinus                 -----VEARNQNSQHSPQTDKDF---EIVTLHE-LREDEA-----------E--AEGDED
Dufourea_novaeangliae             -----IEARSQNRSNSSQTDEHL---ELSTLQD-LQEE-------------E--VEGDED
Megachile_rotundata_X1            -----IDARNQNRSTSPQIDQHL---GLTALQD-SQDE-------------D--AEDEED
Melipona_quadrifasciata           -----VEARNQNRSNSPQINQHL---ELTTLQD-LQEE-------------E--AEGEED
Apis_mellifera_X1                 -----VEARNQNRSNSPQTNQHL---ELATLQD-LQEE-------------E--AEGEED
Eufriesea_mexicana_1              -----VEARNQNRSNSPQIDKHL---ELSSLQD-LQEE-------------E--AEGEED
Eufriesea_mexicana_2              -----VEARNQNRSNSPQIDKHL---ELSSLQD-LQEE-------------E--AEGEED
Bombus_terrestris                 -----VEARNQNKSNSPQIDQHL---ELATLQD-LQEE-------------E--VEGEED
Bombus_impatiens                  -----VEARNQNKSNSPQIDQHL---ELATLQD-LQEE-------------E--VEGEED
Polistes_dominula_X1              -----IEARNQNMSHSPQTDKDL---EMTTLQE-FREE-------------E--AEGEED
Cephus_cinctus_X1                 -----ADARHQNNSHSPHTDKDL---EISTLQD-LRED-------------E--GEGEED
Dinoponera_quadriceps_X2          -----IEARNQNSSHSPQNDKDL---EMAALQD-LREE-------------E--IEGEED
Harpegnathos_saltator_X1          -----IEARNQNSTHSPQNDKDL---EMAALQD-LREE-------------E--VEGEED
Linepithema_humile_X2             -----IEARNQNSSHSPQ-NEDL---EMVALQD-LREE-------------E--LEGGED
Linepithema_humile_X1             -----IEARNQNSSHSPQ-NEDL---EMVALQD-LREE-------------E--LEGGED
Ooceraea_biroi                    -----IEARNQNNSYSSD-NEDL---EMAALQD-LR-E-------------E--IEGDED
Camponotus_floridanus             -----IEARNQNISHSPQNDQDL---EMVALQN-LREE-------------E--IERGED
Solenopsis_invicta                -----IEARNQNNSHSPQNDQDL---EMVALQD-LREE-------------E--IEGEED
Pogonomyrmex_barbatus_X1          -----IEARNQNNSHSPQNDQDL---EMVALQD-LREE-------------E--TEGEED
Vollenhovia_emeryi_X1             -----IEARNQNSSHSPQNDQDL---EMVPLQD-LPEE-------------E--IEGEEN
Wasmannia_auropunctata            -----IEARNQNSSHSPQ-NEDL---EMVALQD-LREE-------------E--MQG-ED
Cyphomyrmex_costatus              -----IEARNQNNSHSPQTDQDL---EMVTLQD-LREE-------------E--MEGEED
Trachymyrmex_septentrionalis      -----IEARNQNNSHSPQTDQDL---EMVALQEHLREE-------------E--MEGEED
Atta_colombica                    -----IEARNQNNSHSPQTDQDL---EMVALQEHLREE-------------E--MEGEED
Trachymyrmex_cornetzi             -----IEARNQNNSHSPQTDQDL---EMVALQEHLREE-------------E--MEGGED
Acromyrmex_echinatior             -----IEARNQNNSHSPQTDQDL---EMVALQERLREE-------------E--MEGEED
Atta_cephalotes                   -----IEARNQNNSHSPQTDQDL---EMVALQEHLREE-------------E--MEGEED


Drosophila_melanogaster           SPIAMVDDQGSHLPFSLPSGFIASSTPDGQTVMATAFQNIPAAQGDISATYVVPDADALD
Halyomorpha_halys                 LSSEL--QHGRL------------------------------------------------
Rhodnius_prolixus                 ------------------------------------------------------------
Cimex_lectularius_X1              VPEEL--NCDTV------------------------------------------------
C._lectularius_X2                 VPEEL--NCDTV------------------------------------------------
Cryptotermes_secundus             -------IFEDT------------------------------------------------
Miniopterus_natalensis            VPL----DRSSH------------------------------------------------
Callithrix_jacchus                VPL----DRSSH------------------------------------------------
Heterocephalus_glaber_X1          VPL----DRSSH------------------------------------------------
Dasypus_novemcinctus              VPL----DRSSH------------------------------------------------
Tupaia_chinensis_X2               VPL----DQSSH------------------------------------------------
Homo_sapiens_X1                   VPL----DRSSH------------------------------------------------
Pongo_abelii_2                    VPL----DRSSH------------------------------------------------
Pan_troglodytes_X2                VPL----DRSSH------------------------------------------------
Boleophthalmus_pectinirostris     IPL----DPSSY------------------------------------------------
Larimichthys_crocea_X2            IPL----DPSSY------------------------------------------------
Larimichthys_crocea               IPL----DPSSY------------------------------------------------
Lates_calcarifer                  IPL----DPSSY------------------------------------------------
Seriola_lalandi_dorsalis          IPL----DPSSY------------------------------------------------
Seriola_dumerili                  IPL----DPSSY------------------------------------------------
Zootermopsis_nevadensis           QHQRAADESYVF------------------------------------------------
Parasteatoda_tepidariorum         -------ESNDH------------------------------------------------
Onthophagus_nigriventris          MPLRLTKELEEI------------------------------------------------
Nicrophorus_vespilloides          TPLRSNQDDENM------------------------------------------------
Tribolium_castaneum               TPLR---IAGDH------------------------------------------------
Nilaparvata_lugens                TPLRVTRDVEDF------------------------------------------------
Blattella_germanica               TPLRETRDVEDF------------------------------------------------
```

```
Nasonia_vitripennis_X1            SPLRQ--DFGDF------------------------------------------------
Microplitis_demolitor             LPLRE--DFGDF------------------------------------------------
Neodiprion_lecontei               SPLRE--DFGDF------------------------------------------------
Athalia_rosae                     SPLRQ--DFGDF------------------------------------------------
Orussus_abietinus                 SPLRR--DFGDF------------------------------------------------
Dufourea_novaeangliae             SPLRQ--DFGDF------------------------------------------------
Megachile_rotundata_X1            SPLRQ--HFGDF------------------------------------------------
Melipona_quadrifasciata           SPLRQ--DFGDF------------------------------------------------
Apis_mellifera_X1                 SPLRQ--DFGDF------------------------------------------------
Eufriesea_mexicana_1              SPLRQ--DFGDF------------------------------------------------
Eufriesea_mexicana_2              SPLRQ--DFGDF------------------------------------------------
Bombus_terrestris                 SPLRQ--DFGDF------------------------------------------------
Bombus_impatiens                  SPLRQ--DFGDF------------------------------------------------
Polistes_dominula_X1              SPLRQ--DFGDF------------------------------------------------
Cephus_cinctus_X1                 SPLRQ--DFGDF------------------------------------------------
Dinoponera_quadriceps_X2          SPLRQ--DFGDF------------------------------------------------
Harpegnathos_saltator_X1          SPLRQ--DFGDF------------------------------------------------
Linepithema_humile_X2             SPLRQ--DFGDF------------------------------------------------
Linepithema_humile_X1             SPLRQ--DFGDF------------------------------------------------
Ooceraea_biroi                    SPLRQ--DFGDF------------------------------------------------
Camponotus_floridanus             SPLRE--DFGDF------------------------------------------------
Solenopsis_invicta                SPLRQ--DFGDF------------------------------------------------
Pogonomyrmex_barbatus_X1          SPLRQ--DFGDF------------------------------------------------
Vollenhovia_emeryi_X1             SPLRQ--DFGDF------------------------------------------------
Wasmannia_auropunctata            SPLRQ--DFGDF------------------------------------------------
Cyphomyrmex_costatus              SPLRQ--DFGDF------------------------------------------------
Trachymyrmex_septentrionalis      SPLRQ--DFGDF------------------------------------------------
Atta_colombica                    SPLRQ--DFGDF------------------------------------------------
Trachymyrmex_cornetzi             SPLRQ--DFGDF------------------------------------------------
Acromyrmex_echinatior             SPLRQ--DFDDF------------------------------------------------
Atta_cephalotes                   SPLRQ--DFGDF------------------------------------------------


Drosophila_melanogaster           GDRGYEIYDPSPKCAELPTSRSGSTGGGKLSGEQHLLPRKGRQPTIMSSSMPDDVIGGSS
Halyomorpha_halys                 ---------------------------------------------------------ELT
Rhodnius_prolixus                 ------------------------------------------------------------
Cimex_lectularius_X1              ---------------------------------------------------------ASC
C._lectularius_X2                 ---------------------------------------------------------ASC
Cryptotermes_secundus             ---------------------------------------------------------S-C
Miniopterus_natalensis            ---------------------------------------------------------S--
Callithrix_jacchus                ---------------------------------------------------------C-Q
Heterocephalus_glaber_X1          ---------------------------------------------------------C-Q
Dasypus_novemcinctus              ---------------------------------------------------------S-Q
Tupaia_chinensis_X2               ---------------------------------------------------------C-Q
Homo_sapiens_X1                   ---------------------------------------------------------C-Q
Pongo_abelii_2                    ---------------------------------------------------------C-Q
Pan_troglodytes_X2                ---------------------------------------------------------C-Q
Boleophthalmus_pectinirostris     ---------------------------------------------------------S-H
Larimichthys_crocea_X2            ---------------------------------------------------------S-Q
Larimichthys_crocea               ---------------------------------------------------------S-Q
Lates_calcarifer                  ---------------------------------------------------------S-Q
Seriola_lalandi_dorsalis          ---------------------------------------------------------S-H
Seriola_dumerili                  ---------------------------------------------------------S-H
Zootermopsis_nevadensis           ---------------------------------------------------------PGN
Parasteatoda_tepidariorum         ---------------------------------------------------------DYR
Onthophagus_nigriventris          ---------------------------------------------------------Y-P
Nicrophorus_vespilloides          ---------------------------------------------------------S-S
Tribolium_castaneum               ---------------------------------------------------------DVN
Nilaparvata_lugens                ---------------------------------------------------------SLS
Blattella_germanica               ---------------------------------------------------------SLG
Nasonia_vitripennis_X1            ---------------------------------------------------------S-S
Microplitis_demolitor             ---------------------------------------------------------A-T
Neodiprion_lecontei               ---------------------------------------------------------A-S
Athalia_rosae                     ---------------------------------------------------------A-S
Orussus_abietinus                 ---------------------------------------------------------A-S
Dufourea_novaeangliae             ---------------------------------------------------------E-S
Megachile_rotundata_X1            ---------------------------------------------------------A-S
Melipona_quadrifasciata           ---------------------------------------------------------A-S
Apis_mellifera_X1                 ---------------------------------------------------------A-S
Eufriesea_mexicana_1              ---------------------------------------------------------APS
Eufriesea_mexicana_2              ---------------------------------------------------------APS
```

```
Bombus_terrestris                   ---------------------------------------------------------A-S
Bombus_impatiens                    ---------------------------------------------------------A-S
Polistes_dominula_X1                ---------------------------------------------------------A-S
Cephus_cinctus_X1                   ---------------------------------------------------------A-N
Dinoponera_quadriceps_X2            ---------------------------------------------------------A-S
Harpegnathos_saltator_X1            ---------------------------------------------------------A-S
Linepithema_humile_X2               ---------------------------------------------------------A-S
Linepithema_humile_X1               ---------------------------------------------------------A-S
Ooceraea_biroi                      ---------------------------------------------------------A-S
Camponotus_floridanus               ---------------------------------------------------------A-S
Solenopsis_invicta                  ---------------------------------------------------------A-S
Pogonomyrmex_barbatus_X1            ---------------------------------------------------------A-S
Vollenhovia_emeryi_X1               ---------------------------------------------------------A-S
Wasmannia_auropunctata              ---------------------------------------------------------A-S
Cyphomyrmex_costatus                ---------------------------------------------------------A-S
Trachymyrmex_septentrionalis        ---------------------------------------------------------A-S
Atta_colombica                      ---------------------------------------------------------A-S
Trachymyrmex_cornetzi               ---------------------------------------------------------A-S
Acromyrmex_echinatior               ---------------------------------------------------------A-S
Atta_cephalotes                     ---------------------------------------------------------A-S


Drosophila_melanogaster             LQPSTASAASSNASSHTGRPSLKKTVADSVRNKANFINRHLFNHKRTGSNASHKSNASNA
Halyomorpha_halys                   ANTA--------------------------------------------------------
Rhodnius_prolixus                   ------------------------------------------------------------
Cimex_lectularius_X1                PSPT--------------------------------------------------------
C._lectularius_X2                   PSPT--------------------------------------------------------
Cryptotermes_secundus               FQSC--------------------------------------------------------
Miniopterus_natalensis              ------------------------------------------------------------
Callithrix_jacchus                  REEA--------------------------------------------------------
Heterocephalus_glaber_X1            REEA--------------------------------------------------------
Dasypus_novemcinctus                RDEA--------------------------------------------------------
Tupaia_chinensis_X2                 REEV--------------------------------------------------------
Homo_sapiens_X1                     REEA--------------------------------------------------------
Pongo_abelii_2                      REEA--------------------------------------------------------
Pan_troglodytes_X2                  REEA--------------------------------------------------------
Boleophthalmus_pectinirostris       REGQ--------------------------------------------------------
Larimichthys_crocea_X2              REQC--------------------------------------------------------
Larimichthys_crocea                 REQC--------------------------------------------------------
Lates_calcarifer                    REQC--------------------------------------------------------
Seriola_lalandi_dorsalis            REQC--------------------------------------------------------
Seriola_dumerili                    REQC--------------------------------------------------------
Zootermopsis_nevadensis             HQGQ---SVRDPSNL------------------------------QLGMKHPSSVT--VP
Parasteatoda_tepidariorum           YFPS--------------------------------------------------------
Onthophagus_nigriventris            LNSC--------------------------------------------------------
Nicrophorus_vespilloides            IASS--------------------------------------------------------
Tribolium_castaneum                 FSLN--------------------------------------------------------
Nilaparvata_lugens                  DDED--------------------------------------------------------
Blattella_germanica                 GGSE--------------------------------------------------------
Nasonia_vitripennis_X1              FEPMT--RRKNGSSG------------------------------HFGMEPFTDNSKSPT
Microplitis_demolitor               VEPS---CFKNSPDI------------------------------RYDLKPYGDNSK-IP
Neodiprion_lecontei                 FEPSGPNSIKISSNP------------------------------IYGATTRNDNSK-VS
Athalia_rosae                       FEPAGPNSIKISSNP------------------------------IYGATTRNENSK-VS
Orussus_abietinus                   FEPT---SIKSNSTP------------------------------RYGSELYGENSK-IP
Dufourea_novaeangliae               FEPS---SIKNSFSP------------------------------HYRMDSYGENSK-TT
Megachile_rotundata_X1              FEPS---IVKNSFSP------------------------------RYRADSYGENSK-AT
Melipona_quadrifasciata             FEP----SIKNSFSP------------------------------RFRMDSYGENSK-TT
Apis_mellifera_X1                   FEPA---SIKNSFGS------------------------------RFRMDSYGENSK-TT
Eufriesea_mexicana_1                FEPS---SIKNSFSP------------------------------RYRMDSYGENSK-AT
Eufriesea_mexicana_2                FEPS---SIKNSFSP------------------------------RYRMDSYGENSK-AT
Bombus_terrestris                   FEPN---SIKNSFSP------------------------------RFRMDSYGENSK-TN
Bombus_impatiens                    FEPN---SIKNSFSP------------------------------RFRMDSYGENSK-TN
Polistes_dominula_X1                FEPT---SIKNSLSP------------------------------RY-TEPYGENSKTTT
Cephus_cinctus_X1                   FEPT---SLKNNSNS------------------------------RYVTEPYGENSK-VS
Dinoponera_quadriceps_X2            FEPT---SIKDDTSP------------------------------RY-INAYGENSK-AV
Harpegnathos_saltator_X1            FEPA---SIENNTSP------------------------------RY-TNTYGENSK-VA
Linepithema_humile_X2               FEPC---NIKNTLSP------------------------------RYGINSYGETSN-AT
Linepithema_humile_X1               FEPC---NIKNTLSP------------------------------RYGINSYGETSN-AT
Ooceraea_biroi                      FEPS---SIKNTLSP------------------------------RYGIDSYGETSK-TT
Camponotus_floridanus               FEPC---SIKNNLSP------------------------------HYGANSYGETS---T
Solenopsis_invicta                  FEPR---SIKNNLSP------------------------------QYEVDSFGETSK-AT
```

```
Pogonomyrmex_barbatus_X1         FEPC---SIKNNLSS------------------------------RYAIGSYGETSK-AT
Vollenhovia_emeryi_X1            FEPR---SIKNNLSP------------------------------RYGADSYGETSK-AI
Wasmannia_auropunctata           FEPR---SIRNNLSP------------------------------RYGADSYGESSK-AT
Cyphomyrmex_costatus             FEPR---SIKNTISP------------------------------RYGADSYGETSK-AT
Trachymyrmex_septentrionalis     FEPR---S-KNTLSP------------------------------RYGTDSYGETSK-AT
Atta_colombica                   FEPR---S-KNTLSP------------------------------RYGADSYGETSK-A-
Trachymyrmex_cornetzi            FEPR---S-KNTLSP------------------------------RYGADSYGETSK-AT
Acromyrmex_echinatior            FEPR---S-KNTLSP------------------------------RYGADSYGETSK-AT
Atta_cephalotes                  FEPR---S-KNTLSP------------------------------RYGADSYGETSK-A-


Drosophila_melanogaster          PSTSSNTNLTSHPVAMGNLGTIES-------GGSGSAGSYTGTPRFYTPSATPGGGSGMA
Halyomorpha_halys                --S--FHTANENTAL--------------YGPI---------------------------
Rhodnius_prolixus                ------------------------------------------------------------
Cimex_lectularius_X1             --N--------------------------------------LMD----------------
C._lectularius_X2                --------------------NLMD------------------------------------
Cryptotermes_secundus            ------ERTERFDSPL-------------------------LSGEFNSLVPEKRTSASVN
Miniopterus_natalensis           --Q--REDDGGSSLGRK--RSYED------------------------------------
Callithrix_jacchus               -----GGRDGGSSLGLK--RSYDE------------------------------------
Heterocephalus_glaber_X1         -----GGREGGSSLGIK--RNYEE------------------------------------
Dasypus_novemcinctus             -----GGRDGGSSLGLK--RNYEE------------------------------------
Tupaia_chinensis_X2              -----GGRDGGSSLGLK--RNYEE------------------------------------
Homo_sapiens_X1                  -----GGRDGGSSLGFK--RSYEE------------------------------------
Pongo_abelii_2                   -----GGRDGGSSLGFK--RSYEE------------------------------------
Pan_troglodytes_X2               -----GGRDGGSSLGFK--RSYEE------------------------------------
Boleophthalmus_pectinirostris    --S--LDRDEAASMGLL--GSYEE------------------------------------
Larimichthys_crocea_X2           -----LDRDEASSMGLR--GSYEE------------------------------------
Larimichthys_crocea              -----LDRDEASSMGLR--GSYEE------------------------------------
Lates_calcarifer                 -----LDRDEASSMGLR--GSYEE------------------------------------
Seriola_lalandi_dorsalis         -----LDRDEASSMGLR--GSYEE------------------------------------
Seriola_dumerili                 -----LDRDEASSMGLR--GSYEE------------------------------------
Zootermopsis_nevadensis          P-N--RHEISDSYVLPEHLPGFPETEHVLYGPGSDFTLQHNSFETAPLKRTSQTGSSVIR
Parasteatoda_tepidariorum        ----------ATMLPEE-----DEAEEIVNGGELVEATIS-SVDELRTVHVNSN------
Onthophagus_nigriventris         --------ADDSEV---------------------------EDDLVGSP-----------
Nicrophorus_vespilloides         -------------------DSSEE------------------------------------
Tribolium_castaneum              -----SDDSN---------DEFET------------------------------------
Nilaparvata_lugens               --D--YEPHLPAPAPAP---------------------------ATDTKVSNGSATAPTP
Blattella_germanica              -----EEEDDLPDADVE--VGYQP-----------------HYHTMSVP-----------
Nasonia_vitripennis_X1           SLALGFLDLNSSKAPLK--AGFDD-----FDGVSVGSLAS-SKDTLNLPFAEDSVKPAKN
Microplitis_demolitor            A-S--FHDLNSSKIPLK--AGFDD-----FDGVSVGSLCS-SKDTLNFPFAEESLKSVKN
Neodiprion_lecontei              P-N--FHEFNSSKVPLK--ADFDD-----FEGASMESLRS-SRDTLDLPFAEDSLKSVKN
Athalia_rosae                    P-N--FHEFNSSKVPLK--ADFED-----FEGASMESLRS-SRDTLDLPFAEESLKSVKN
Orussus_abietinus                S-A--FHDLPSSKIPLK--AGFED-----FDDVSAESMSS-SKDTLNLPFAEDSLKSVKS
Dufourea_novaeangliae            NMN--SHNMNSSNVPLK--AGFDD-----FDGVSAGSIAS-GKDTLNLPFVEDSLKSVKS
Megachile_rotundata_X1           A-T--CQDFNSSNVPLK--AVFDD-----FDGVSADSVIS-GKDALNLPFVEDSFKSVKS
Melipona_quadrifasciata          T-N--CHDMNSSNVPLK--AGFDD-----FDGVSADSIAS-GKDVLNLPFVEDSLKSVKS
Apis_mellifera_X1                P-N--CHDMNSSNVPLK--AGFDD-----FDGVSADSIASIAKDALNLPFVEDSLKSVKS
Eufriesea_mexicana_1             S-N--CHDMNSSNVPLK--AGFDD-----FDGVSADSVAS-GKDALNLPFVEDSLKSVKS
Eufriesea_mexicana_2             S-N--CHDMNSSNVPLK--AGFDD-----FDGVSADSVAS-GKDALNLPFVEDSLKSVKS
Bombus_terrestris                S-N--CHDMNSSNVPLK--AGFDD-----FDGVSADSIAS-GKDVLNLPFVEDSLKSVKS
Bombus_impatiens                 S-N--CHDMNSSNVPLK--AGFDD-----FDGVSADSIAS-GKDVLNLPFVEDSLKSVKS
Polistes_dominula_X1             T-N--FHDMNLSKIPLK--AGFDD-----FDGISGDSLAS-SKDTLNLPFVEDSLKSVKN
Cephus_cinctus_X1                R-T--FHDLNSSKVPLK--AGFDD-----FDGVSGDSLAS-SKDTLNLPFAEESLNSVRN
Dinoponera_quadriceps_X2         S-S--FHDMNSSKVPLK--AGFDD-----FDGVSGDSLAS-SKDTLNLPFVEDSLKSVKS
Harpegnathos_saltator_X1         S-T--FHDMNSSKVPLK--AGFDD-----FDGISGDSLAS-SKDTLNLPCVEDSLKSVKS
Linepithema_humile_X2            S-N--FHDMNSSEVP-K--AGFDE-----FGGISGDSLVS-N------------------
Linepithema_humile_X1            S-N--FHDMNSSEVP-K--AGFDE-----FGGISGDSLVS-SKDTLNSPYVEGSLKSVRS
Ooceraea_biroi                   P-D--FHDMNCSKIPLK--AGFED-----FDGVSGDSL-S-SKDTLNLPFVEDSLKSVKS
Camponotus_floridanus            S-N--FHDMNSSKVP-K--AGFDE-----FVGVSGDSLVS-SKDTLNSPFIEGSLKSVRS
Solenopsis_invicta               S-N--FHDMNSTKVP-K--AGFDE-----FGGISGDSLVS-SKDTLNSPFV-GSLKSV-S
Pogonomyrmex_barbatus_X1         S-N--FHDMNSIKVP-K--AGFDE-----FGGVSGDSLVS-SKDTLNSPFVEGSLKSVRS
Vollenhovia_emeryi_X1            S-N--FHDMNSTKVP-K--AGFDE-----FGGISGDSLVS-SKDTLNSPFVEGSLKSVRS
Wasmannia_auropunctata           S-N--FHDMSSIKVP-K--AGFDE-----FGGISGDSLVS-SKDTLNSPFVEGSLKSVRS
Cyphomyrmex_costatus             S-N--FHDMNSIKVP-K--AGFDE-----FGGISGDSLVS-SKDTLNLPFVEGSLKSIRS
Trachymyrmex_septentrionalis     S-N--FHDMNSIKVP-K--AGFDE-----FGGISGDSLVS-SKDTLNSPFVEGSLKSVRS
Atta_colombica                   S-N--FHDMNSIKVP-K--AGFDE-----FGGISGDSLVS-SKDTLNSPFVEGSLKSVRS
Trachymyrmex_cornetzi            S-N--FHDMNSIKVP-K--AGFDE-----FGGISGDSLVS-SKDTLNSPFVEGSLKSVRS
Acromyrmex_echinatior            S-N--FHDMNSIKVP-K--AGFDE-----FGGISGDSLVS-SKDTLNSPFVEGSLKSVRS
Atta_cephalotes                  S-N--FHDMNSIKVP-K--AGFDE-----FGGISGDSLVS-SKDTLNSPFVEGSLKSVRS
```

```
Drosophila_melanogaster           ISDNPNYRLLDESIASEQATILTTSSPNPNYEMMHPPTSLVSTNPNYMPMNETPVQMAGV
Halyomorpha_halys                 ------------------------------------------------------------
Rhodnius_prolixus                 ------------------------------------------------------------
Cimex_lectularius_X1              ------------------------------------------------------------
C._lectularius_X2                 ------------------------------------------------------------
Cryptotermes_secundus             SNMETLPNNSRSSIA---------------------------------------------
Miniopterus_natalensis            ------------------------------------------------------------
Callithrix_jacchus                ------------------------------------------------------------
Heterocephalus_glaber_X1          ------------------------------------------------------------
Dasypus_novemcinctus              ------------------------------------------------------------
Tupaia_chinensis_X2               ------------------------------------------------------------
Homo_sapiens_X1                   ------------------------------------------------------------
Pongo_abelii_2                    ------------------------------------------------------------
Pan_troglodytes_X2                ------------------------------------------------------------
Boleophthalmus_pectinirostris     ------------------------------------------------------------
Larimichthys_crocea_X2            ------------------------------------------------------------
Larimichthys_crocea               ------------------------------------------------------------
Lates_calcarifer                  ------------------------------------------------------------
Seriola_lalandi_dorsalis          ------------------------------------------------------------
Seriola_dumerili                  ------------------------------------------------------------
Zootermopsis_nevadensis           KASQLIPQTGYGLLDNEYSQPENIDPTIPSQVTSDSCSKILFAGLG--DGYVPDENILPK
Parasteatoda_tepidariorum         ----------------------------------------------------------DG
Onthophagus_nigriventris          ------------------------------------------------------------
Nicrophorus_vespilloides          ------------------------------------------------------------
Tribolium_castaneum               ------------------------------------------------------------
Nilaparvata_lugens                NGFIRRP-----------------------------------------------------
Blattella_germanica               -----------------------RKLSTPPNGIV--------------------------
Nasonia_vitripennis_X1            SPFAQKKSSSKGNVS--QSSAGKSSL-SPQSPSIIATTGNLGSKPS--PSLV------SS
Microplitis_demolitor             SPSIKNNSTNRRNIS----PLSSKNSLSPQ-NKR---NQIILRNRG--QNK-------SV
Neodiprion_lecontei               SPFVDKRNNSRVNSTGDNSVLGKNSI-NPN-NVV---KRNFSESPR--F---------SK
Athalia_rosae                     SPFVEKKNNSRGNLS--DGSIGK-TL-SPG-SIA---RKNFLETPR--F---------ST
Orussus_abietinus                 SPFSERKTPSRSNAS--LASTA-KST-SPR-SMT---KSAFPESPE--A---------PK
Dufourea_novaeangliae             SPFMNAKSSSRSNIS--QLSVDNKSI-SPRNAG----KRSYLNSPN--S---------SK
Megachile_rotundata_X1            SPFATAKSVSRSNLS--QLSTGNRSA-SPKSTG----KRSFLSSPN--S---------SR
Melipona_quadrifasciata           SPFINAKSISRSNLS--QLSISNRSA-SPKNHSG---RRSFLSSPN--S---------SK
Apis_mellifera_X1                 SPFINAKSASRSNLS--QLSTSNRSATSPKNPSG---KRSFLGSPN--S---------SK
Eufriesea_mexicana_1              SPFINAKSASRSNLS--QLSISNKSG-SPKNPS----KRSFLSSPN--S---------SK
Eufriesea_mexicana_2              SPFINAKSASRSNLS--QLSISNKSG-SPKNPS----KRSFLSSPN--S---------SK
Bombus_terrestris                 SPFINAKSTSRSNLS--QLSISNRSG-SPKNPS----KRSFLSSPN--S---------SK
Bombus_impatiens                  SPFINAKSTSRSNLS--QLSISNRSG-SPKNPS----KRSFLSSPN--S---------SK
Polistes_dominula_X1              SPFIHAKSTSKGIDN--QTSTG-RAT-SLQ-NLI---KQHIQQQQR--TQEDSPISIKSK
Cephus_cinctus_X1                 SPFAEKRSTSRGNIS--QISVG-KSL-TPQ-TGN---KRVFLESPG--L---------SR
Dinoponera_quadriceps_X2          SPFIHNKSTSRSN---------------SE-SLV--------------------------
Harpegnathos_saltator_X1          SPFIQNKSTSRSNVS--YVSLG-KSA-SPQ-SLV---KRNFLDSPN--P---------SC
Linepithema_humile_X2             ------------------------------------------------------------
Linepithema_humile_X1             SPFIYKKSTSRSNIS--QASLG-KSV-SPQ-GLV---RQNFLDSPN--S---------SK
Ooceraea_biroi                    SPFIHTKSASRSNVS--QASLA-KSA-SPQ-SKV---KQNFLDSPN--L---------SK
Camponotus_floridanus             SPFIYKKSTSRSNVS--QGSLV-KSG-SPQ-SLV---KRYYLDSPN--L---------PK
Solenopsis_invicta                SPFIYKKSTSRGNVS--QSSLG-KSA-SPQ-SLV---KRNFLDSPN--P---------SK
Pogonomyrmex_barbatus_X1          SPFIFKKSTSRSNIS--QSSLG-KPG-SPPGSLV---KRSFLDSPN--P---------SK
Vollenhovia_emeryi_X1             SPFIYKKSTSRSNVS--QGSLG-KPA-SPQ-SLV---KRHFFDSPN--L---------SK
Wasmannia_auropunctata            SPFIYKKSTSRSNVS--QGSLG-KPG-SPQ-SLV---KRNFLDSPN--P---------SK
Cyphomyrmex_costatus              SPFIYKKSTSRSNVS--QGSLG-KPG-SPQ-SLV---KRNFLDSPN--P---------SK
Trachymyrmex_septentrionalis      SPFIYKKSTSRSNVS--QGSLG-KSG-SPQ-SLV---KRNFLDSPN--P---------SK
Atta_colombica                    SPFIYKKSTSRSNVS--QGSLG-KSG-SPQ-SLV---KRNFLDSPN--P---------SK
Trachymyrmex_cornetzi             SPFIYKKSTSRSNVS--QGSLG-KSG-SPQ-SLV---KRNFLDSPN--P---------SK
Acromyrmex_echinatior             SPFIYKKSTSRSNVS--QGSLG-KSG-SPQ-SLV---KRNFLDSPN--P---------SK
Atta_cephalotes                   SPFIYKKSTSRSNVS--QGSLG-KSG-SPQ-SLV---KRNFLDSPN--P---------SK
```

```
Drosophila_melanogaster           TISHNPNYQPMQAPLNARQSQSSSDEDNEQEEDDEDEDDDVDDEHVEHIKMERMPLSRPR
Halyomorpha_halys                 ------------------------------------------------------------
Rhodnius_prolixus                 ------------------------------------------------------------
Cimex_lectularius_X1              ------------------------------------------------------------
C._lectularius_X2                 ------------------------------------------------------------
Cryptotermes_secundus             ----EPHYLNMCKRTS--------------------------------------------
Miniopterus_natalensis            --------------------------------------------HISYTHMN--------
Callithrix_jacchus                --------------------------------------------HIPYTHMN--------
Heterocephalus_glaber_X1          --------------------------------------------HIPYTHMN--------
Dasypus_novemcinctus              --------------------------------------------HIPYTHMN--------
Tupaia_chinensis_X2               --------------------------------------------HIPYTHMN--------
```

```
Homo_sapiens_X1                 --------------------------------------------HIPYTHMN--------
Pongo_abelii_2                  --------------------------------------------HIPYTHMN--------
Pan_troglodytes_X2              --------------------------------------------HIPYTHMN--------
Boleophthalmus_pectinirostris   -------------------------------------------HHMPFTHMN--------
Larimichthys_crocea_X2          -------------------------------------------HHIPFTHMN--------
Larimichthys_crocea             -------------------------------------------HHIPFTHMN--------
Lates_calcarifer                -------------------------------------------HHIPFTHMN--------
Seriola_lalandi_dorsalis        -------------------------------------------HHIPFTHMN--------
Seriola_dumerili                -------------------------------------------HHIPFTHMN--------
Zootermopsis_nevadensis         THSEECDLHSSVSAVRTQDCAP---------SENNSVEGDTR-LQAQGSKCPVATVSSGN
Parasteatoda_tepidariorum       SKGVSVNYSDGSKGSKISTLSN--------------------------------------
Onthophagus_nigriventris        --------------------------------------------HMEFSSYPKVQKDTT-
Nicrophorus_vespilloides        -------------------------------------EELEPDTNIEFITY---------
Tribolium_castaneum             ----------------------------------------DAETHIRFPSIPVENKDG--
Nilaparvata_lugens              ------------------------------------------------------------
Blattella_germanica             --------GNGSAATS--------------------------------------------
Nasonia_vitripennis_X1          TRS-DPEYENRSPEAV---DQL---------QQQPPTRDVAS-LRVSFPSMDAIDSDETV
Microplitis_demolitor           HNIDDLDYENQSTELM---SNA---------EM----KDINS-TQLDYSSLDNVDVTISV
Neodiprion_lecontei             IKSEDPDYENKSPEVFV-TSGD---------TI----KNTNV-HRVEFPSIDGID-DKNI
Athalia_rosae                   IKTEDPDYENKIPEVVLSTSGD---------QI----KDSNI-RRIEFPSIDDVE-DKNI
Orussus_abietinus               PRG-NSDYENRSPERV----SL---------NA----KNAST--RIEIVDINDVVVDDQK
Dufourea_novaeangliae           I-A-NPDYENGSPEIL---SAA---------EK----KETVP-LRVDFPSMDAIGIDHEI
Megachile_rotundata_X1          LAN-LPDYENGSPEIL---PAV---------NK----RETMP-LRVDFPSMDAIDVDNRL
Melipona_quadrifasciata         L-I-NPDYENGSPEIL---LAA---------EK----RDTVP-LRVDFSAID-IDV----
Apis_mellifera_X1               IAA-NPDYENGSPEIL--SAAA---------EK----RETVP-LRLDFPSIDAIDIDNGM
Eufriesea_mexicana_1            L-A-NPDYENGSPEIL---AVA---------EK----RETVP-LRVDFPSIDAIDIDNGM
Eufriesea_mexicana_2            L-A-NPDYENGSPEIL---AVA---------EK----RETVP-LRVDFPSIDAIDIDNGM
Bombus_terrestris               L-V-NPDYENGSPEIL---SAA---------EK----REAVP-LRVDFPSMDTINIDNGM
Bombus_impatiens                L-V-NPDYENGSPEIL---SAA---------EK----REAVP-LRVDFPSMDTINIDNGM
Polistes_dominula_X1            SKK-NPDYENSSPMIL---ANY---------ER----N-----FSFPSLSLNTLEIDVDI
Cephus_cinctus_X1               TRY-APDYENHSPEFI---NDE---------NN----KDAIP-LRIDFPSMEAIDDDEPE
Dinoponera_quadriceps_X2        ------------------------------------------------------------
Harpegnathos_saltator_X1        KPRGNPDYENRSPEIM---LGL---------EK----KETAP-MHVNFPSMDAIDIDING
Linepithema_humile_X2           ------------------------------------------------------------
Linepithema_humile_X1           PQS-DPDYEN-SPEIM---SGL---------EK----K-ISP-LHIEFASMDATDIEING
Ooceraea_biroi                  SRNLDPDYENKSSEII---SGS---------AK----KETAP-LHVKFPSVDEMDIEING
Camponotus_floridanus           SRG-DPDYENRSPETI---LAI---------EK----REITTPLHVEFPSVDVMDIQING
Solenopsis_invicta              LRD-DPDYENRSLEII---SGI---------EK----KEIIS-LHVEFPSVDVMDIQING
Pogonomyrmex_barbatus_X1        FRD-DPDYENRSPEIM---SSI---------EK----KEITS-LHVEFPSMDVIDIQING
Vollenhovia_emeryi_X1           FRD-DPDYENRSPEIM---SGI---------EK----KEITS-LHVEFPSVDVMDIQING
Wasmannia_auropunctata          RRD-DPDYENRSPEIM---SGV---------EK----KEITS-LHVEFPSVDVMDIQING
Cyphomyrmex_costatus            LRD-DPDYENRSPEIM---SGI---------EK----KEITS-LHVEFPSVDVMDIQING
Trachymyrmex_septentrionalis    LQD-DPDYENRSPEIM---SGI---------EK----REITS-LHVEFPSVDV---QING
Atta_colombica                  LQD-DPDYENRNPEIM---SGI---------EK----REITS-LHVEFPSVDV---QING
Trachymyrmex_cornetzi           LQD-DPDYENRSPEIM---SGI---------EK----REITS-LHVEFPSVDV---QING
Acromyrmex_echinatior           LQD-DPDYENRSPEIM---SGI---------EK----REITS-LHVEFPSMDV---QVNG
Atta_cephalotes                 LQD-DPDYENRSPEIM---SGI---------EK----REITS-LHVEFPSVDV---QING


Drosophila_melanogaster         QRALPSKTQPPRSRSVSQTRKSPTNPNSGIGATGAGNRSNLLKENWLRPASTPRPPPPNG
Halyomorpha_halys               ------------------------------------------------------------
Rhodnius_prolixus               ------------------------------------------------------------
Cimex_lectularius_X1            ------------------------------------------------------------
C._lectularius_X2               ------------------------------------------------------------
Cryptotermes_secundus           ------------------------------------------------------RFNADE
Miniopterus_natalensis          ------------------------------------------------------GGKKNG
Callithrix_jacchus              ------------------------------------------------------GGKKNG
Heterocephalus_glaber_X1        ------------------------------------------------------GGKKNG
Dasypus_novemcinctus            ------------------------------------------------------GGKKNG
Tupaia_chinensis_X2             ------------------------------------------------------GGKKNG
Homo_sapiens_X1                 ------------------------------------------------------GGKKNG
Pongo_abelii_2                  ------------------------------------------------------GGKKNG
Pan_troglodytes_X2              ------------------------------------------------------GGKKNG
Boleophthalmus_pectinirostris   ------------------------------------------------------GGKSNG
Larimichthys_crocea_X2          ------------------------------------------------------GGKTNG
Larimichthys_crocea             ------------------------------------------------------GGKTNG
Lates_calcarifer                ------------------------------------------------------GGKTNG
Seriola_lalandi_dorsalis        ------------------------------------------------------GGKTNG
Seriola_dumerili                ------------------------------------------------------GGKTNG
Zootermopsis_nevadensis         RAIP---------SSKDSPKGDDGN-----------------KSKPRPP-----NGMVNG
Parasteatoda_tepidariorum       ------------------------------------------------------GSIANG
```

```
Onthophagus_nigriventris         ------------------------------------------------------TGTANG
Nicrophorus_vespilloides         ------------------------------------------------------TSTANG
Tribolium_castaneum              ------------------------------------------------------ITTANG
Nilaparvata_lugens               ------------------------------------------------------PAANGN
Blattella_germanica              ------------------------------------------------------PTAANG
Nasonia_vitripennis_X1           IPETTSSIVVDSNSSSATLPSAKQQ-----------------YAN--KSGEQQAAPLNNG
Microplitis_demolitor            -------------QEAEPQS--QTD-----------------YLE--KC-----ESLNNG
Neodiprion_lecontei              -------------NDNGGNKS-KVD-----------------YKSNNKQ-----EGLNNG
Athalia_rosae                    -------------NEIGGNKS-KVD-----------------YKS--KP-----ETLNNG
Orussus_abietinus                -------------EKS------PVE-----------------YVN--RS-----ETLNNG
Dufourea_novaeangliae            -------------EKKDTI-P-ITD-----------------YVN--KP-----ETLNNG
Megachile_rotundata_X1           -------------EKKETF-S-DSD-----------------FTN--KS-----ENLNNG
Melipona_quadrifasciata          -------------ETNEPL-S-PVE-----------------YVN--KP-----EALNNG
Apis_mellifera_X1                -------------ERKEALSS-PVE-----------------YVN--KP-----ETLNNG
Eufriesea_mexicana_1             -------------EKKETL-S-PVE-----------------YVN--KP-----ETLNNG
Eufriesea_mexicana_2             -------------EKKETL-S-PVE-----------------YVN--KP-----ETLNNG
Bombus_terrestris                -------------EKKEMK-S-PVE-----------------YVN--KP-----ETLNNG
Bombus_impatiens                 -------------EKKEMK-S-PVE-----------------YVN--KP-----ETLNNG
Polistes_dominula_X1             -------------EGKDRRHSPRVE-----------------YMN--EP-----ETLNNG
Cephus_cinctus_X1                -------------VRKDIKKA-QAE-----------------YMN--KP-----ETLNNG
Dinoponera_quadriceps_X2         ------------------------------------------------------------
Harpegnathos_saltator_X1         -------------DKSESKS--MGG-----------------YVN--KP-----ETLNNG
Linepithema_humile_X2            ------------------------------------------------------------
Linepithema_humile_X1            -------------DKKDSKY--TAD-----------------YVN--KP-----ETLNNG
Ooceraea_biroi                   -------------EKNESKH--AGE-----------------YVN--KP-----ETLNNG
Camponotus_floridanus            -------------DKKETKC--TTD-----------------YVN--KS-----ETLNNG
Solenopsis_invicta               -------------NKTENNKKHTGD-----------------YMN--KS-----ETLNNG
Pogonomyrmex_barbatus_X1         -------------DKKESNKH-MSD-----------------YMN--KS-----ETLNNG
Vollenhovia_emeryi_X1            -------------DKTENNKR-TGD-----------------YMN--KS-----ETLNNG
Wasmannia_auropunctata           -------------DKTENNKRTTGD-----------------YMN--KS-----EILNNG
Cyphomyrmex_costatus             -------------DKTENNKR-TGD-----------------YIN--KS-----EILNNG
Trachymyrmex_septentrionalis     -------------DKTENNKR-MGD-----------------YMN--KS-----EMLNNG
Atta_colombica                   -------------DKTENNKR-TGD-----------------YMN--KS-----EMLNNG
Trachymyrmex_cornetzi            -------------DKTENNKR-TGD-----------------YMN--KS-----EMLNNG
Acromyrmex_echinatior            -------------DKTENNKR-TGD-----------------YMN--KS-----EMLNNG
Atta_cephalotes                  -------------DKTENNKR-TGD-----------------YMN--KS-----EMLNNG


Drosophila_melanogaster          FIGREA------------------
Halyomorpha_halys                ------------------------
Rhodnius_prolixus                ------------------------
Cimex_lectularius_X1             ------------------------
C._lectularius_X2                ------------------------
Cryptotermes_secundus            YILG--------------------
Miniopterus_natalensis           RVLTLPRSSPS-------------
Callithrix_jacchus               RILALPRSNPS-------------
Heterocephalus_glaber_X1         RILPLPRSSPS-------------
Dasypus_novemcinctus             RILTLPRSNPS-------------
Tupaia_chinensis_X2              RILTLPRSNPS-------------
Homo_sapiens_X1                  RILTLPRSNPS-------------
Pongo_abelii_2                   RILTLPRSNPS-------------
Pan_troglodytes_X2               RILTLPRSNPS-------------
Boleophthalmus_pectinirostris    RILALPRSTPS-------------
Larimichthys_crocea_X2           RILALPRSSPS-------------
Larimichthys_crocea              RILALPRSSPS-------------
Lates_calcarifer                 RILALPRSSPS-------------
Seriola_lalandi_dorsalis         RILALPRSSPS-------------
Seriola_dumerili                 RILALPRSSPS-------------
Zootermopsis_nevadensis          YISLPQEDGNECVPPLK-------
Parasteatoda_tepidariorum        HAYCAKSN----------------
Onthophagus_nigriventris         YVGTNPSNGTRA------------
Nicrophorus_vespilloides         FVGANK------------------
Tribolium_castaneum              YVSGCPTNGAATTQC---------
Nilaparvata_lugens               SVNVKTTKC---------------
Blattella_germanica              WVVGNGTGMKTTQC----------
Nasonia_vitripennis_X1           YIGGPAT-----------------
Microplitis_demolitor            YISRTTT-----------------
Neodiprion_lecontei              YISGKTTQC---------------
Athalia_rosae                    YISGKTTQC---------------
Orussus_abietinus                YIGGATT-----------------
Dufourea_novaeangliae            YIGGTTT-----------------
```

```
Megachile_rotundata_X1            YIGGATT-----------------
Melipona_quadrifasciata           YIGGTATKQLTLRPCASSYAYTAL
Apis_mellifera_X1                 YIGGTTT-----------------
Eufriesea_mexicana_1              YIGSTTT-----------------
Eufriesea_mexicana_2              YIGSTTT-----------------
Bombus_terrestris                 YISSTTT-----------------
Bombus_impatiens                  YISGTTT-----------------
Polistes_dominula_X1              YIGGNNKKT---------------
Cephus_cinctus_X1                 YIGGAAT-----------------
Dinoponera_quadriceps_X2          ------------------------
Harpegnathos_saltator_X1          YIGNTTT-----------------
Linepithema_humile_X2             ------------------------
Linepithema_humile_X1             YIGNTTT-----------------
Ooceraea_biroi                    YIGNTTT-----------------
Camponotus_floridanus             YIGNTAT-----------------
Solenopsis_invicta                YIGSTTT-----------------
Pogonomyrmex_barbatus_X1          YIGNTAT-----------------
Vollenhovia_emeryi_X1             YIGNTAT-----------------
Wasmannia_auropunctata            YIGNTTT-----------------
Cyphomyrmex_costatus              YISNTTT-----------------
Trachymyrmex_septentrionalis      YISNTTT-----------------
Atta_colombica                    YISNTTT-----------------
Trachymyrmex_cornetzi             YISNTTT-----------------
Acromyrmex_echinatior             YISNTTT-----------------
Atta_cephalotes                   YISNTTT-----------------
```

Supplementary Figure 2. Multiple alignment of insulin receptor sequences. Rhop-IR predicted aminoacid sequence (RPRC006251-PA) was aligned with 57 sequences of insulin receptors from 54 other species. Vertebrate (*Larimichthys crocea* XP_019121818.1 and KKF23242.1, *Boleophthalmus pectinirostris* XP_020784849.1, *Lates calcarifer* XP_018533362.1, *Seriola lalandi dorsalis* XP_023281132.1, *S. dumerili* XP_022614011.1, *Pongo abelii* PNJ04174.1, *Pan troglodytes* XP_016790340.1, *Dasypus novemcinctus* XP_004477219.1, *Miniopterus natalensis* XP_016072913.1, *H. sapiens* XP_011526290.2, *Tupaia chinensis* XP_014440404.1, *Callithrix jacchus* XP_017823137.1, *Heterocephalus glaber* XP_004865373.1) and invertebrate (*Cimex lectularius* XP_014242610.1 and XP_014242611.1, *Halyomorpha halys* XP_014273072.1, *Zootermopsis nevadensis* KDR21367.1, *Trachymyrmex septentrionalis* XP_018338965.1, *Atta colombica* KYM81501.1, *A. cephalotes* XP_012056411.1, *Trachymyrmex cornetzi* KYN13503.1, *Camponotus floridanus* XP_011251747.1, *Bombus terrestris* XP_003393794.1, *B. impatiens* XP_003490625.1, *Blattella germanica* CDI30232.1, *Cyphomyrmex costatus* KYN02366.1, *Acromyrmex echinatior* XP_011051139.1, *Wasmannia auropunctata* XP_011696537.1, *Vollenhovia emeryi* XP_011864734.1, *Ooceraea biroi* XP_011347281.1, *Cryptotermes secundus* PNF38775.1, *Dinoponera quadriceps* XP_014476872.1, *Microplitis demolitor* XP_014295647.1, *Linepithema humile* XP_012228696.1 and XP_012228695.1, *Neodiprion lecontei* XP_015510416.1, *Pogonomyrmex barbatus* XP_011648404.1, *Eufriesea mexicana* XP_017754627.1 and XP_017756280.1, *Orussus abietinus* XP_023289633.1, *Athalia rosae* XP_012260906.2, *Harpegnathos saltator* XP_011144146.1, *Solenopsis invicta* XP_011158641.1, *Melipona quadrifasciata* KOX70962.1, *Polistes dominula* XP_015177172.1, *Dufourea novaeangliae* XP_015431438.1, *Megachile rotundata* XP_012136521.1, *Nasonia vitripennis* XP_016838980.1, *Nicrophorus vespilloides* XP_017772616.1, *Parasteatoda tepidariorum* XP_015920989.1, *D. melanogaster* AAC47458.1, *O. nigriventris* AFQ20827.1, *Tribolium castaneum* AHF20214.1, *Nilaparvata lugens* AIY24638.1, *Apis mellifera* XP_016773490.1) sequences were used. The region highlighted in yellow represents the extracellular portion of the receptor comprising the leucine-and cysteine-rich domains. The region highlighted in red indicates the intracellular tyrosine kinase domain, which was used to construct the phylogenetic tree. An asterisk (*****) indicates positions that have a single, fully conserved aminoacid residue, a colon (**:**) indicates conservation between aminoacid groups of strongly similar properties, and a period (**.**) indicates conservation between aminoacid groups of weakly similar properties.

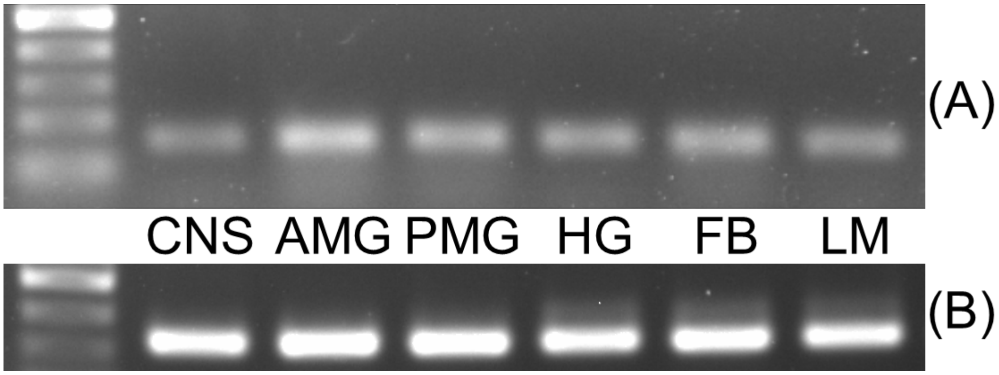

Supplementary Figure 3. Semi-quantitative analysis of Rhopr-IR transcript expression in 5th instars. Unfed 5th instar *R. prolixus* were dissected and tissues were pooled into 6 different groups (CNS – central nervous system, AMG – anterior midgut, PMG – posterior midgut, HG – hindgut, FB – fat body, LM – leg muscles). (A) indicates Rhopr-IR transcript amplification and (B) indicates actin transcript amplification, which was used as a reference control. The experiment was repeated 3 times.

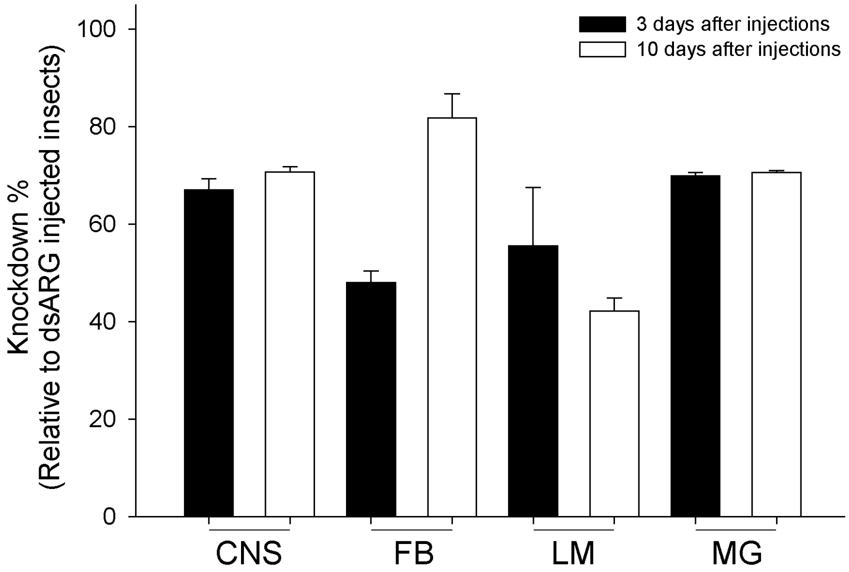

Supplementary Figure 4. Analysis of Rhopr-IR transcript expression in 5th instars post dsRNA injection. The double stranded RNAs for Rhopr-IR(dsIR) and Ampicilin Resistance Gene (dsARG) were injected into unfed 5th instar *R. prolixus* and insects were dissected at 3 and at 10 days post-injections. Tissues were pooled into 4 different groups (CNS – central nervous system, FB – fat body, LM – leg muscles, MG – midgut) and the expression of Rhopr-IR in dsIR-injected insects was quantified relative to the expression of Rhopr-IR in dsARG-injected insects by using qPCR and the ΔΔCt method (Pfaffl, 2001). The experiment was repeated 3 times and results are shown as means + std error.

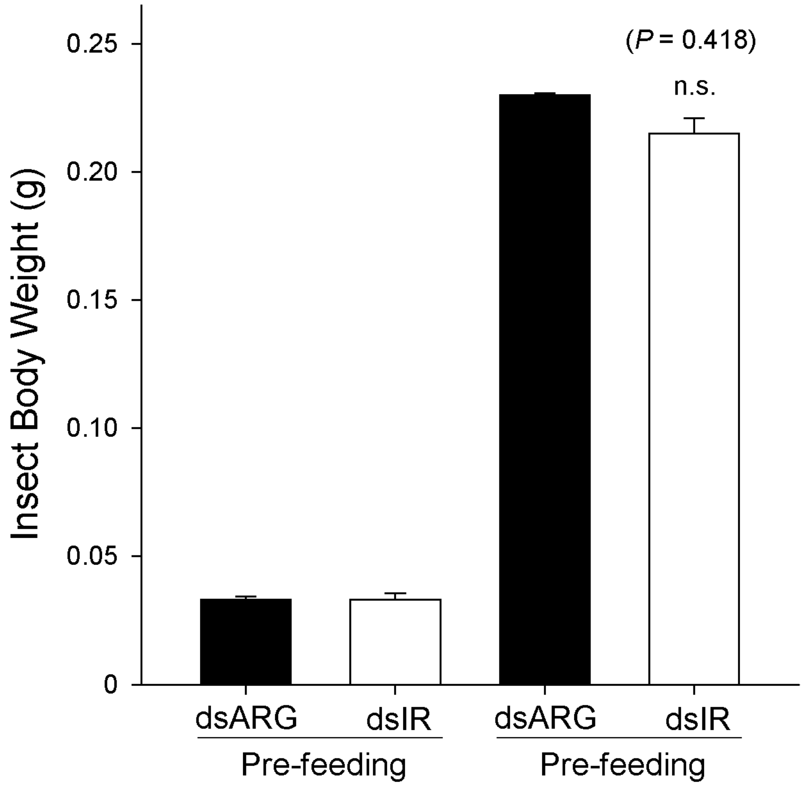

Supplementary Figure 5. Post-feeding body weight of Rhopr-IR knockdown insects. 5th instar *R. prolixus* were injected with 1 µg of dsARG or dsIR in 1 µL of ultrapure water and fed on defibrinated rabbit blood 3 days after injections. Insects were fed in groups of 40 individuals and allowed to feed for 25 minutes. Results are shown as means + std errors and the experiment was repeated for a total of 3 times. No statistical difference in post-feeding body weight was observed between dsARG- and dsIR-injected insects.

Supplemental Data – Full Western blots

i. *Figure 4, BpV vs. Saline – Western blots probing for pTyr (a), actin (b), Akt (c), and pAkt (d)*

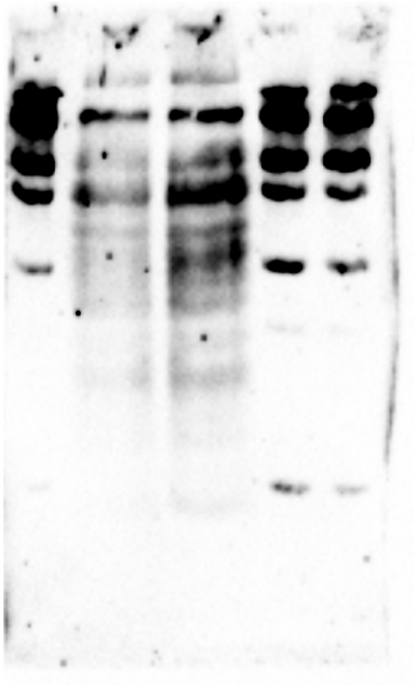

(a) Image displays chemiluminescent detection of anti-pTyr, using an anti-pTyr antibody, for blot shown in Figure 4. Some epitopes of proteins found within the ladders (lanes 1, 4 and 5) were also detected; it is possible that some of the proteins used in these ladders contain pTyr sites (information on proteins contained in protein ladders not disclosed by manufacturer).

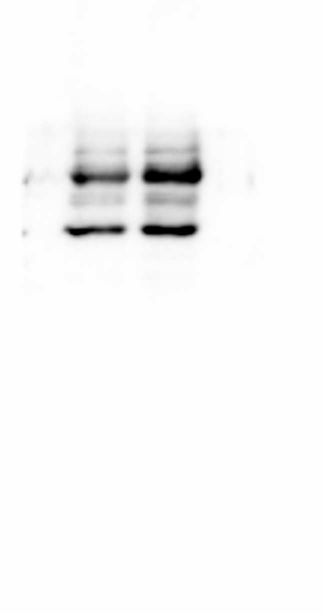

(b) Image displays chemiluminescent detection of anti-actin, using an anti-B-actin primary antibody, for blot shown in Figure 4. Multiple bands observed, potentially as a result of different actin homolog variants within insects, or due to the detection of similar epitopes within different insect proteins. Bottom set of bands used as actin controls in Figure 4, based on MW identification of typical actin protein size in mammals (~42 kDa). Ladder not shown.

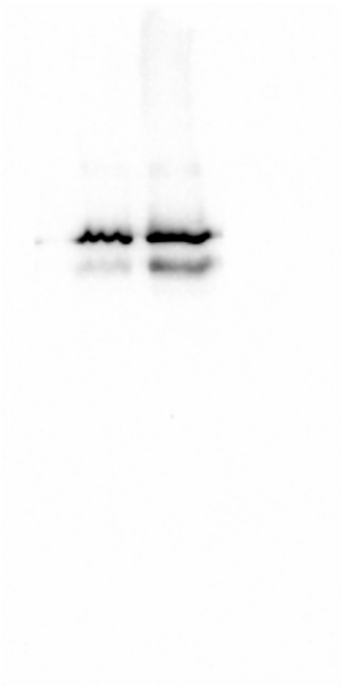

(c) Image displays chemiluminescent detection of anti-Akt, using an anti-Akt primary antibody, for blot shown in Figure 4. Multiple bands observed, potentially as a result of the detection of different Akt homologs that could be present in *R. prolixus*, or due to the detection of similar epitopes within different insect proteins. Top set of bands used as Akt protein signal, based on MW identification of typical Akt protein size in mammals (~60 kDa). Ladder not shown.

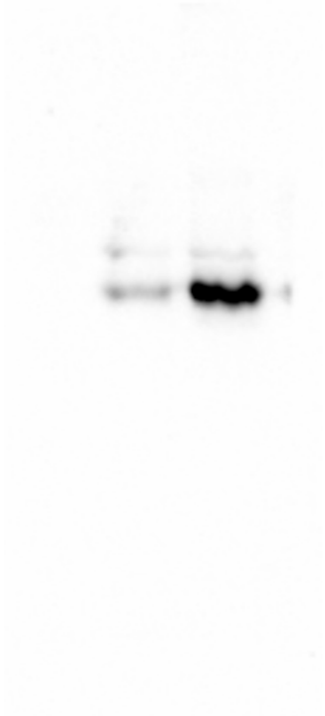

(d) Image displays chemiluminescent detection of anti-pAkt, using an anti-pAkt primary antibody, for blot shown in Figure 4. Multiple bands observed, potentially as a result of the detection of different pAkt homologs that could be present in *R. prolixus*, or due to the detection of similar epitopes within different insect proteins. Bottom set of bands used as pAkt protein signal, based on MW identification of typical pAkt protein size in mammals (~60 kDa). Ladder not shown.

ii. Figure 5A, weeklong feed experiment – pAkt (a), pGSK3B (b), pFOXO (c), and actin (d)

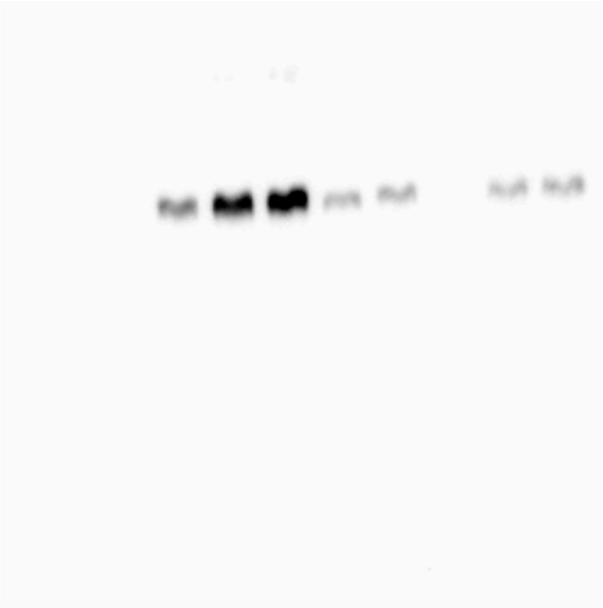

(a) Image displays chemiluminescent detection of anti-pAkt, using an anti-pAkt primary antibody, for blot shown in Figure 5A. Multiple bands observed, potentially as a result of the detection of different pAkt homologs that could be present in *R. prolixus*, or due to the detection of similar epitopes within different insect proteins. Bottom set of bands used as pAkt protein signal, based on MW identification of typical pAkt protein size in mammals (~60 kDa). Last two lanes not shown in Figure 5. Ladder not shown.

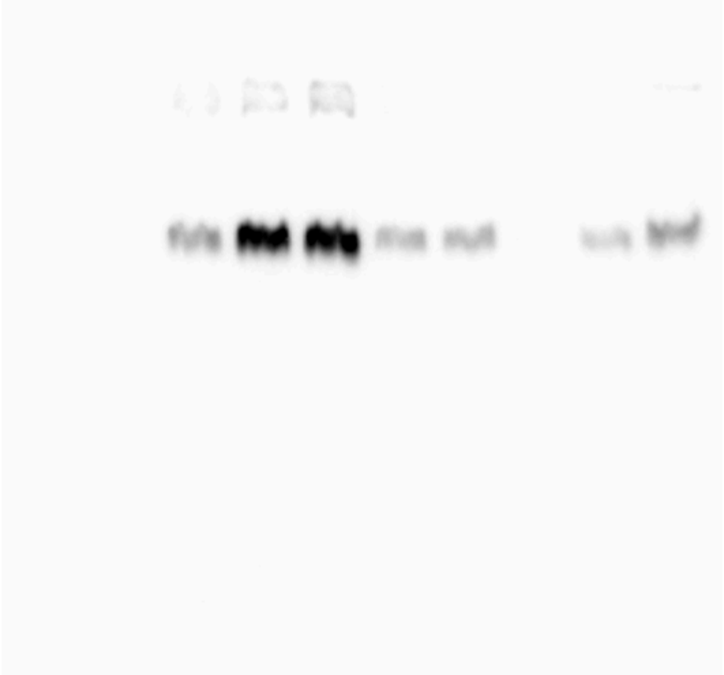

(b) Image displays chemiluminescent detection of anti-pGSK3B, using an anti-pGSK3B primary antibody, for blot shown in Figure 5A. Multiple bands observed, potentially as a result of the detection of different pGSK3B homologs that could be present in *R. prolixus*, or due to the detection of similar epitopes within different insect proteins. Bottom set of bands used as pGSK3B protein signal, based on MW identification of typical pGSK3B protein size in mammals (~46 kDa). Last two lanes not shown in Figure 5. Ladder not shown.

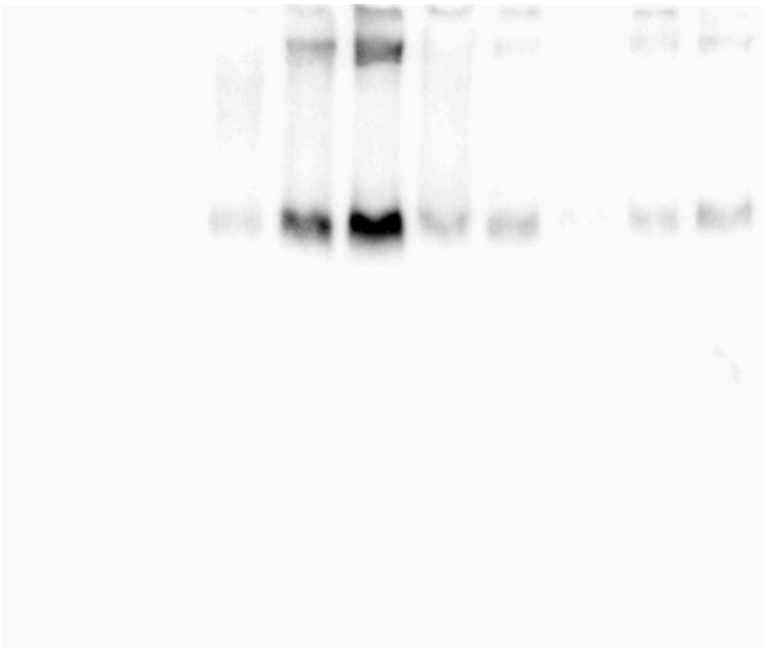

(c) Image displays chemiluminescent detection of anti-pFOXO, using an anti-pFOXO primary antibody, for blot shown in Figure 5A. Multiple bands observed, potentially as a result of the detection of different pFOXO homologs that could be present in *R. prolixus*, or due to the detection of similar epitopes within different insect proteins. Bottom set of bands used as pFOXO protein signal, based on MW identification of typical pFOXO protein size in mammals (~82 kDa). Last two lanes not shown in Figure 5. Ladder not shown.

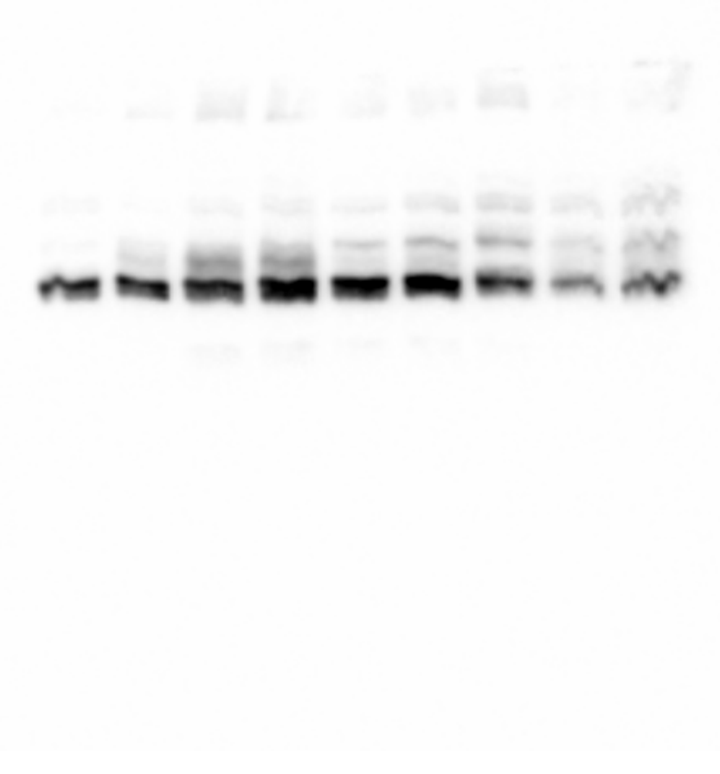

(d) Image displays chemiluminescent detection of anti-actin, using an anti-B-actin primary antibody, for blot shown in Figure 5A. Multiple bands observed, potentially as a result of the detection of different actin homologs that could be present in *R. prolixus*, or due to the detection of similar epitopes within different insect proteins. Bottom set of bands used as actin protein signal and control, based on MW identification of typical actin protein size in mammals (~42 kDa). Last two lanes not shown in Figure 5. Ladder not shown.

iii.   Figure 7A, saline vs. porcine insulin – pAkt (a), and Akt (b)

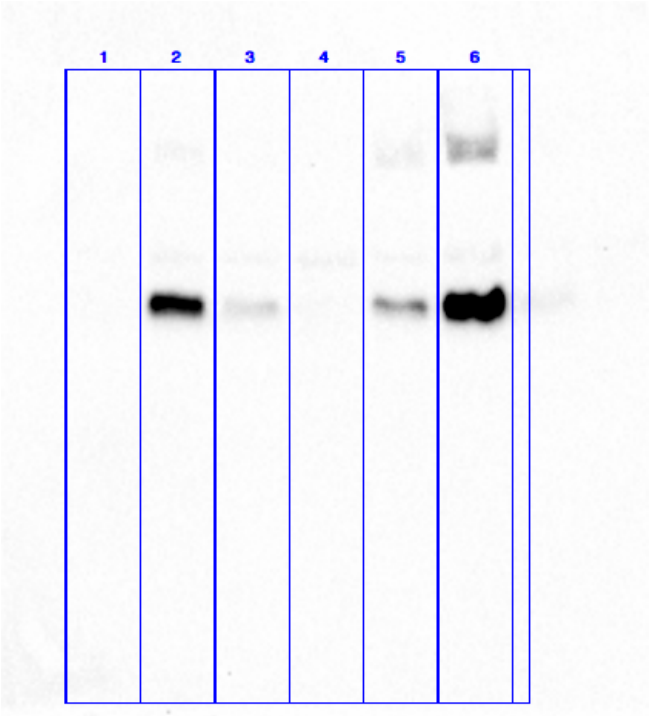

(a) Image displays chemiluminescent detection of anti-pAkt, using an anti-pAkt primary antibody, for blot shown in Figure 7A. Multiple bands observed, potentially as a result of the detection of different pAkt homologs that could be present in *R. prolixus*, or due to the detection of similar epitopes within different insect proteins. Bottom set of bands used as pAkt protein signal, based on MW identification of typical pAkt protein size in mammals (~60 kDa). Lanes correspond to the following conditions, as displayed in Figure 7A: Lane 1 = saline (- glucose); Lane 2 = saline (+ glucose); Lane 5 = porcine insulin (1 µg); Lane 6 = porcine insulin (0.1 µg). Ladder not shown. Lanes 3 and 4 not shown in Figure 7A.

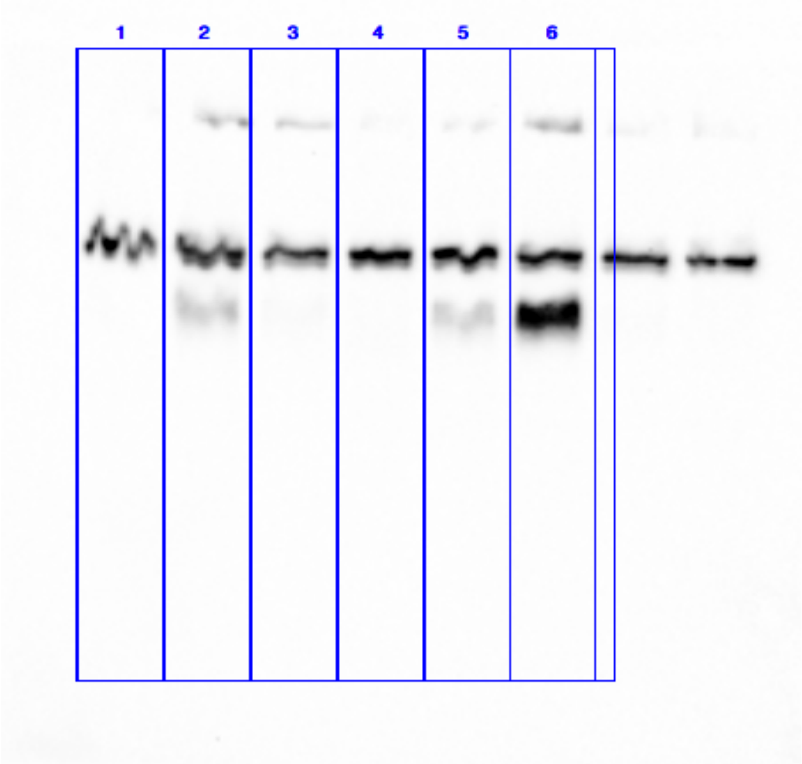

(b) Image displays chemiluminescent detection of anti-Akt, using an anti-Akt primary antibody, for blot shown in Figure 7A. Multiple bands observed, potentially as a result of the detection of different Akt homologs that could be present in *R. prolixus*, or due to the detection of similar epitopes within different insect proteins. Middle set of bands used as Akt protein signal, based on MW identification of typical Akt protein size in mammals (~60 kDa). Identified lanes correspond to the following conditions, as displayed in Figure 7A: Lane 1 = saline (- glucose); Lane 2 = saline (+ glucose); Lane 3 = human insulin (1 µg); Lane 4 = human insulin (0.1 µg); Lane 5 = porcine insulin (1 µg); Lane 6 = porcine insulin (0.1 µg). Ladder not shown. Lanes 3, 4, and last 2 lanes not shown in Figure 7A.

iv. *Figure 10, dsIR injections – pAkt (a), pGSK3B (b), pFOXO (c), and tubulin (d)*

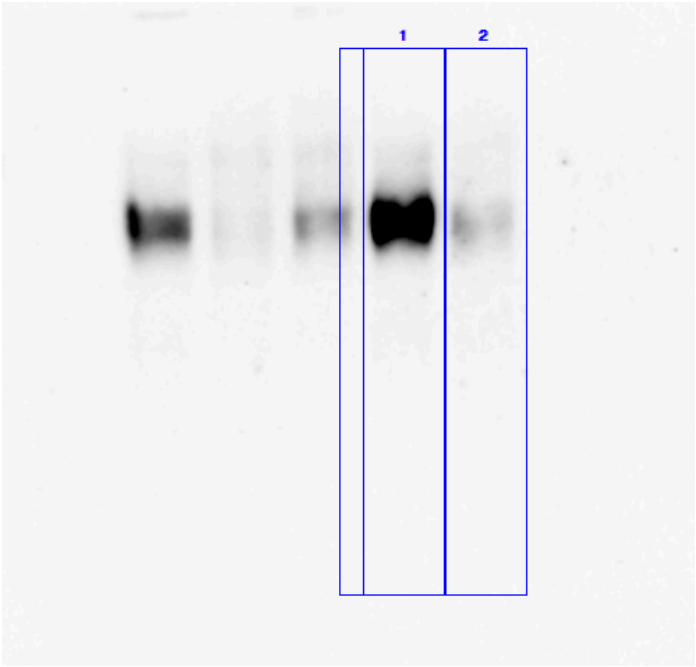

(a) Image displays chemiluminescent detection of anti-pAkt, using an anti-pAkt primary antibody, for blot shown in Figure 10. Multiple bands observed, potentially as a result of the detection of different pAkt homologs that could be present in *R. prolixus*, or due to the detection of similar epitopes within different insect proteins. Samples were obtained solely from fat bodies of unstimulated, starved 5th instar *R. prolixus*, unlike other blots shown in Figures 4, 5 and 7, and thus the propensity for detecting background noise is also higher since the signal intensity of pFOXO is inherently weaker without stimulation of the pathway by feeding or pharmacological intervention. Bottom set of bands used as pAkt protein signal, based on MW identification of typical pAkt protein size in mammals (~60 kDa). Identified lanes correspond to the following conditions, as displayed in Figure 10: Lane 1 = dsARG; Lane 2 = dsIR. Ladder not shown. Other lanes not used in Fig. 10.

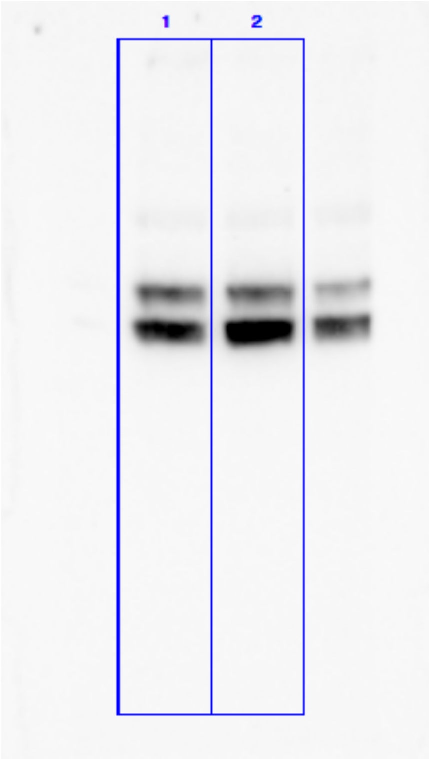

(b) Image displays chemiluminescent detection of anti-pGSK3B, using an anti-pGSK3B primary antibody, for blot shown in Figure 5A. Multiple bands observed, potentially as a result of the detection of different pGSK3B homologs that could be present in *R. prolixus*, or due to the detection of similar epitopes within different insect proteins. Samples were obtained solely from fat bodies of unstimulated, starved 5th instar *R. prolixus*, unlike other blots shownin Figures 4, 5 and 7, and thus the propensity for detecting background noise is also higher since the signal intensity of pGSK3B is inherently weaker without stimulation of the pathway by feeding or pharmacological intervention. Bottom set of bands used as pGSK3B protein signal, based on MW identification of typical pGSK3B protein size in mammals (~46 kDa). Identified lanes correspond to the following conditions, as displayed in Figure 10: Lane 1 = dsARG; Lane 2 = dsIR. Ladder not shown. Other lanes not used in Fig. 10.

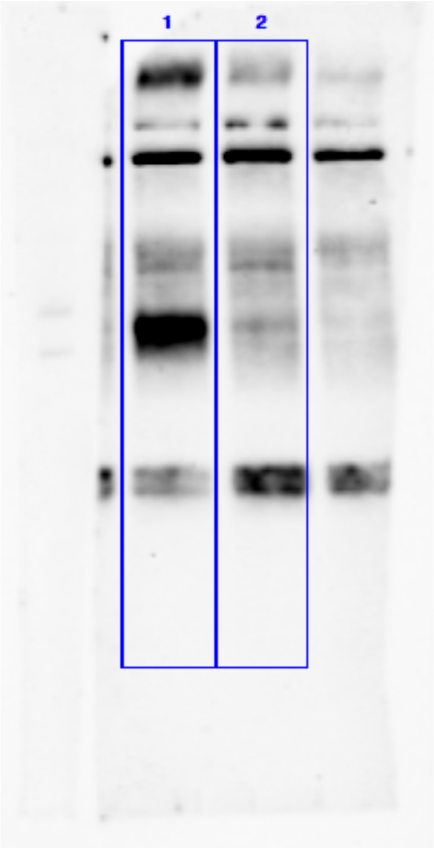

(c) Image displays chemiluminescent detection of anti-pFOXO, using an anti-pFOXO primary antibody, for blot shown in Figure 10. Multiple bands observed, potentially as a result of the detection of different pFOXO homologs that could be present in *R. prolixus*, or due to the detection of similar epitopes within different insect proteins. Samples were obtained solely from fat bodies of unstimulated, starved 5th instar *R. prolixus*, unlike other blots shownin Figures 4, 5 and 7, and thus the propensity for detecting background noise is also higher since the signal intensity of pFOXO is inherently weaker without stimulation of the pathway by feeding or pharmacological intervention. Middle set of bands used as pFOXO protein signal, based on MW identification of typical pFOXO protein size in mammals (~82 kDa). Identified lanes correspond to the following conditions, as displayed in Figure 10: Lane 1 = dsARG; Lane 2 = dsIR. Ladder not shown. Other lanes not used in Fig. 10.

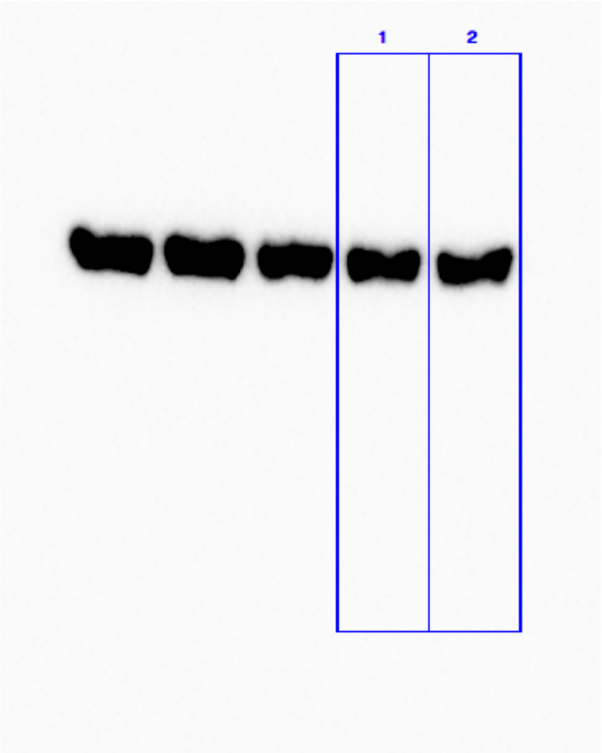

(d) Image displays chemiluminescent detection of anti-tubulin, using an anti-a-tubulin primary antibody, for blot shown in Figure 10. Single set of bands used as tubulin protein signal control, based on MW identification of typical a-tubulin protein size in mammals (~50 kDa). Identified lanes correspond to the following conditions, as displayed in Figure 10: Lane 1 = dsARG; Lane 2 = dsIR. Ladder not shown. Other lanes not used in Fig. 10.
